# Exploration of Long-Chain Vitamin E Metabolites for the Discovery of a Highly Potent, Orally Effective, and Metabolically Stable 5-LOX Inhibitor that Limits Inflammation

**DOI:** 10.1021/acs.jmedchem.1c00806

**Published:** 2021-07-19

**Authors:** Konstantin Neukirch, Khaled Alsabil, Chau-Phi Dinh, Rossella Bilancia, Martin Raasch, Alexia Ville, Ida Cerqua, Guillaume Viault, Dimitri Bréard, Simona Pace, Veronika Temml, Elena Brunner, Paul M. Jordan, Marta C. Marques, Konstantin Loeser, André Gollowitzer, Stephan Permann, Jana Gerstmeier, Stefan Lorkowski, Hermann Stuppner, Ulrike Garscha, Tiago Rodrigues, Gonçalo J. L. Bernardes, Daniela Schuster, Denis Séraphin, Pascal Richomme, Antonietta Rossi, Alexander S. Mosig, Fiorentina Roviezzo, Oliver Werz, Jean-Jacques Helesbeux, Andreas Koeberle

**Affiliations:** † Michael Popp Institute and Center for Molecular Biosciences Innsbruck (CMBI), University of Innsbruck, 6020 Innsbruck, Austria; ‡ Department of Pharmaceutical/Medicinal Chemistry, Institute of Pharmacy, Friedrich Schiller University Jena, 07743 Jena, Germany; § Univ Angers, SONAS, SFR QUASAV, F-49000 Angers, France; α Department of Pharmacy, School of Medicine and Surgery, University of Naples Federico II, 80131 Naples, Italy; ⊥ Institute of Biochemistry II, Jena University Hospital, 07747 Jena, Germany; # Department of Pharmaceutical and Medicinal Chemistry, Paracelsus Medical University Salzburg, 5020 Salzburg, Austria; ∇ Instituto de Medicina Molecular João Lobo Antunes, Faculdade de Medicina, Universidade de Lisboa, 1649-028 Lisboa, Portugal; ○ Department of Nutritional Biochemistry and Physiology, Institute of Nutritional Science and Competence Cluster for Nutrition and Cardiovascular Health (nutriCARD), Halle-Jena-Leipzig, Friedrich Schiller University Jena, 07743 Jena, Germany; ◆ Institute of Pharmacy/Pharmacognosy and Center for Molecular Biosciences Innsbruck (CMBI), University of Innsbruck, 6020 Innsbruck, Austria; ¶ Department of Pharmaceutical/Medicinal Chemistry, Institute of Pharmacy, University of Greifswald, 17489 Greifswald, Germany; †† Department of Chemistry, University of Cambridge, CB2 1EW Cambridge, U.K.

## Abstract

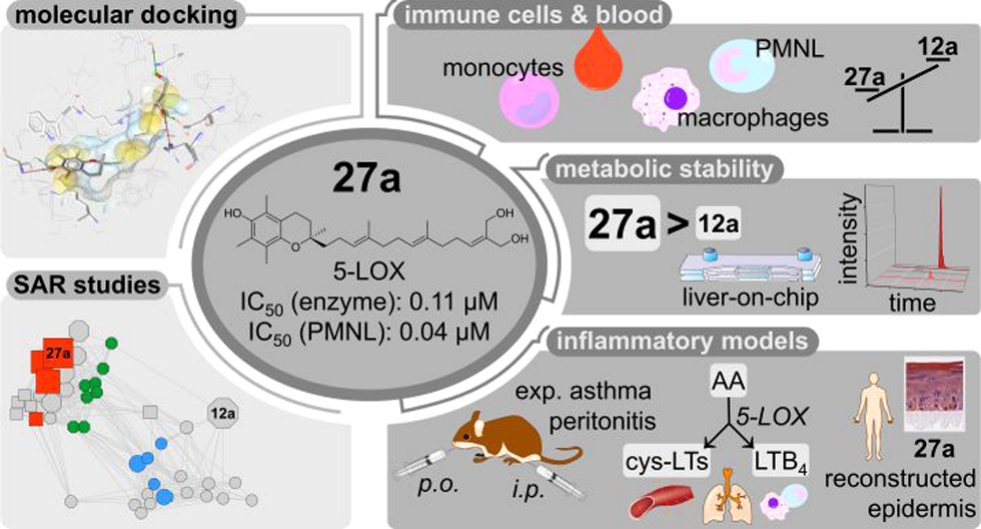

Endogenous long-chain metabolites of vitamin E (LCMs) mediate immune functions by targeting 5-lipoxygenase (5-LOX) and increasing the systemic concentrations of resolvin E3, a specialized proresolving lipid mediator. SAR studies on semisynthesized analogues highlight α-amplexichromanol (**27a**), which allosterically inhibits 5-LOX, being considerably more potent than endogenous LCMs in human primary immune cells and blood. Other enzymes within lipid mediator biosynthesis were not substantially inhibited, except for microsomal prostaglandin E_2_ synthase-1. Compound **27a** is metabolized by sulfation and β-oxidation in human liver-on-chips and exhibits superior metabolic stability in mice over LCMs. Pharmacokinetic studies show distribution of **27a** from plasma to the inflamed peritoneal cavity and lung. In parallel, 5-LOX-derived leukotriene levels decrease, and the inflammatory reaction is suppressed in reconstructed human epidermis, murine peritonitis, and experimental asthma in mice. Our study highlights **27a** as an orally active, LCM-inspired drug candidate that limits inflammation with superior potency and metabolic stability to the endogenous lead.

## Introduction

Deficiency of vitamin E causes a dysfunctional immune response, degenerative diseases, and potentially atherosclerosis and Alzheimer’s disease.^1−5^ The discovery that the vital antioxidant vitamin E mediates immune functions through endogenous long-chain metabolites (LCMs) recently revived research on this field.^6−8^ LCMs are produced from α-tocopherol (**1a**) and other vitamin E forms (**1b–d, 6a–d**) by hepatic ω-oxidation, yielding ω-alcohols and then ω-carboxylic acids, which are excreted via bile and feces, shortened by successive β-oxidations, or conjugated with sulfate or glucoronate for urinary elimination.^7,8^ The LCMs α-T-13′-CH_2_OH (**9a**) and α-T-13′-COOH (**12a**) were detected at low nanomolar concentrations in human plasma, albeit with strong variation between individuals.^6,8,9^ These differences in **1a** metabolism may provide an explanation for the mixed outcomes of human vitamin E intervention studies^8,10,11^ and open the door toward personalized pharmacotherapy. Notably, LCMs reach the highest concentration in the liver, which correlates with the recently confirmed clinical efficiency of **1a** in nonalcoholic fatty liver disease (NAFLD).^12^

We have shown that **12a** accumulates within immune cells at sites of inflammation, such as the inflamed peritoneal cavity of mice, limits the inflammatory reaction in murine peritonitis, and suppresses bronchial hyperreactivity in experimental asthma by targeting 5-lipoxygenase (5-LOX).^6^ LCMs bind to an allosteric site between the 5-LOX catalytic and regulatory domains and inhibit the enzyme at concentrations that are reached in plasma for **12a**.^6^

5-LOX initiates the biosynthesis of powerful immunomodulatory lipid mediators from polyunsaturated fatty acids that are released from membrane phospholipids by cytosolic phospholipase (cPL)A_2_.^13^ 5-Lipoxygenase-activating protein (FLAP) transfers arachidonic acid to 5-LOX at the nuclear membrane, where leukotriene (LT)A_4_ is synthesized via 5-hydro(pero)xyeicosatetraenoic acid (5-H(P)ETE) as an intermediate.^14^ LTA_4_ is converted to either the potent chemoattractant LTB_4_ or cysteinyl-LTs that elevate vascular permeability and trigger smooth-muscle contraction.^15−17^ LTs are central for asthma and allergic rhinitis and contribute to dermatitis, inflammatory liver disorders, neurodegenerative diseases, cardiovascular disease, and cancer.^14,15,18−21^

5-LOX is also involved in the biosynthesis of lipoxins, resolvins, and other specialized proresolving mediators that orchestrate resolution, pathogen clearance, and tissue regeneration,^22,23^ but the impact of 5-LOX in this process differs between immune cell populations.^14,24^ Remarkably, **12a** strongly increased systemic resolvin E3 levels in mice during the resolution phase, whereas the 5-LOX inhibitor zileuton, which is in clinical use for asthma therapy, did not show a comparable effect.^6^ These findings suggest that **12a**, besides suppressing acute inflammation, promotes resolution, which would be a major advantage in treating chronic inflammation.^22^

Additional targets besides 5-LOX were proposed to contribute to the anti-inflammatory effectiveness of LCMs. Garcinoic acid (δ-TE-13′-COOH, **13d**), a potential LCM with unknown physiological relevance in humans, has agonistic activity on specific nuclear receptors, such as pregnane X receptor and peroxisome proliferator-activated receptor γ, a mechanism that is partially shared by other LCMs.^25,26^ Of note, nuclear receptor activation by compound **13d** has been proposed to diminish Alzheimer’s disease progression by interfering with β-amyloid oligomerization and deposition.^27^ Further immunomodulatory targets were reported for LCMs at supraphysiological concentrations. 13′-Alcohols and 13′-carboxylic acids derived from **1a**, δ-tocopherol (**1d**), and δ-tocotrienol (**6d**) suppress lipopolysaccharide (LPS)-induced signal transduction in macrophages, thereby lowering cytokine release and the expression of proinflammatory enzymes, such as inducible nitric oxide synthase (iNOS) and cyclooxygenase (COX)-2.^28−30^ Moreover, the 13′-carboxylic acid of δ-tocopherol (**12b**), but not α-tocopherol (**12a**), reduces enzymatic COX-2 activity,^6,31^ and tocotrienol-derived 13′-carboxylic acids (**13a–d**) inhibit microsomal prostaglandin E_2_ synthase (mPGES)-1,^32^ an inducible enzyme that is functionally coupled to COX-2 and responsible for excessive prostaglandin (PG)E_2_ formation during inflammation.^33,34^ The *in vivo* relevance of COX-2 and mPGES-1 inhibition is unclear,^35^ but their moderate inhibition might be beneficial to buffer substrate redirection from LT to PG biosynthesis.^36^

The recent insights into bioactive vitamin E metabolites promise access to a new generation of 5-LOX-targeting drugs that suppress inflammation without impairing but instead triggering resolution. LCMs potently inhibit 5-LOX, beneficially adjust lipid mediator profiles, and are enriched in immune cells at inflammatory sites.^6^ As endogenous metabolites, they might be less afflicted with toxicity than zileuton and diverse clinical candidates targeting 5-LOX.^37^ Challenges for drug development were, however, the limited knowledge about pharmacophores, the elusive oral availability, and the rapid hepatic LCM metabolism. We here explored the structural requirements for 5-LOX inhibition, taking differences in cellular uptake into account, and identified α-amplexichromanol (α-TE-12a′,13′-diCH_2_OH, **27a**), a highly potent allosteric 5-LOX inhibitor, which combines a favorable pharmacological profile with oral availability and superior metabolic stability. Compound **27a** accumulates in immune cells, is efficiently distributed to inflamed regions, and shows anti-inflammatory effectiveness in experimental models of atopic dermatitis *in vitro* and murine peritonitis and bronchial hyperreactivity *in vivo*.

## Results

### Design and Semisynthesis of Chromanols Inspired from Bioactive Vitamin E Metabolites

**Scheme 1:**
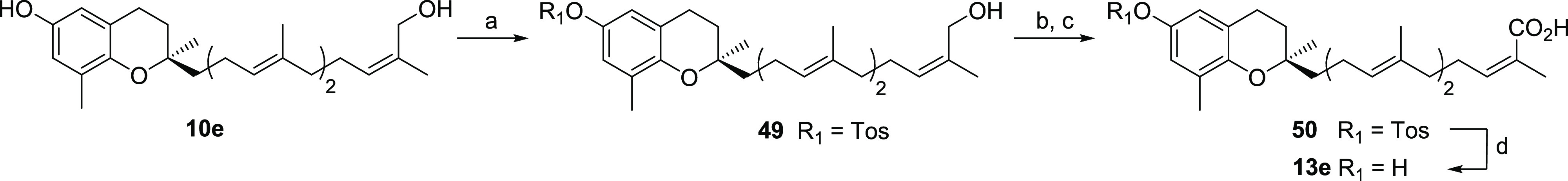
Preparation of δ-(*Z*)-Garcinoic Acid **13e** Reagents and conditions: (a) TosCl, Et_3_N, DCM, rt., 2.5 h, 72%; (b) 2-iodoxybenzoic acid, DCM/DMSO (9:1), 40 °C, 6 h; (c) NaClO_2_, H_2_NSO_3_H, 2-methyl-2-butene, 1,4-dioxane/H_2_O (1:1), 0 °C, 2 h, 72% (2 steps); (d) NaOH, MeOH, 70 °C, 3 h, 95%.

Compound **13d** was extracted and purified from *Garcinia kola* nuts according to a previously described method with an optimized yield compared to the literature.^39,40^ Chromanols from the amplexichromanol series (**10e**, **27c**, **27d**) were isolated from *Garcinia amplexicaulis* stem barks.^41^ Both α- and β-forms (**13a**, **13b**, **27a**, **27b**) were obtained from the corresponding δ-forms of the garcinoic acid (**13d**) and amplexichromanol (**27d**) series through a two-step strategy, which involves the preparation of the mono- or bis-Mannich bases and their reduction with sodium cyanoborohydride.^42,43^ δ-(*Z*)-Garcinoic acid **13e** has been previously isolated from *Clusia grandiflora*, more specifically from fruits, and subsequently characterized.^44^ In the current study, it was prepared from the corresponding alcohol **10e** extracted from the stem barks of this plant and also found in *G. amplexicaulis* (Scheme 1). Two oxidative steps, namely, 2-iodoxybenzoic acid-mediated and sodium chlorite-mediated, were applied to the tosyl-protected precursor **49**. Final hydrolysis led to **13e** with 51% overall yield over four steps.

**Scheme 2:**
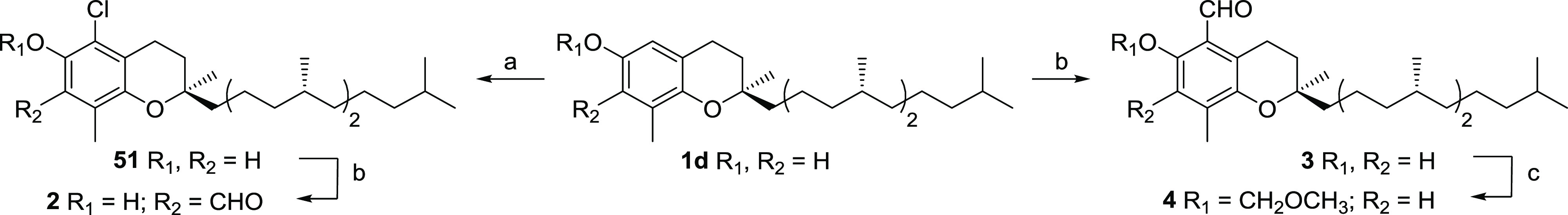
General Preparation of Formyltocopherols **2**–**4** Reagents and conditions: (a) NCS, MeOH, rt., 2 h, 95%; (b) MgCl_2_, Et_3_N, (CHO)*_n_*, THF, reflux, 75%; (c) MOMBr, *n*BuLi, THF, −5 °C, 2 h, 80%.

**Scheme 3:**
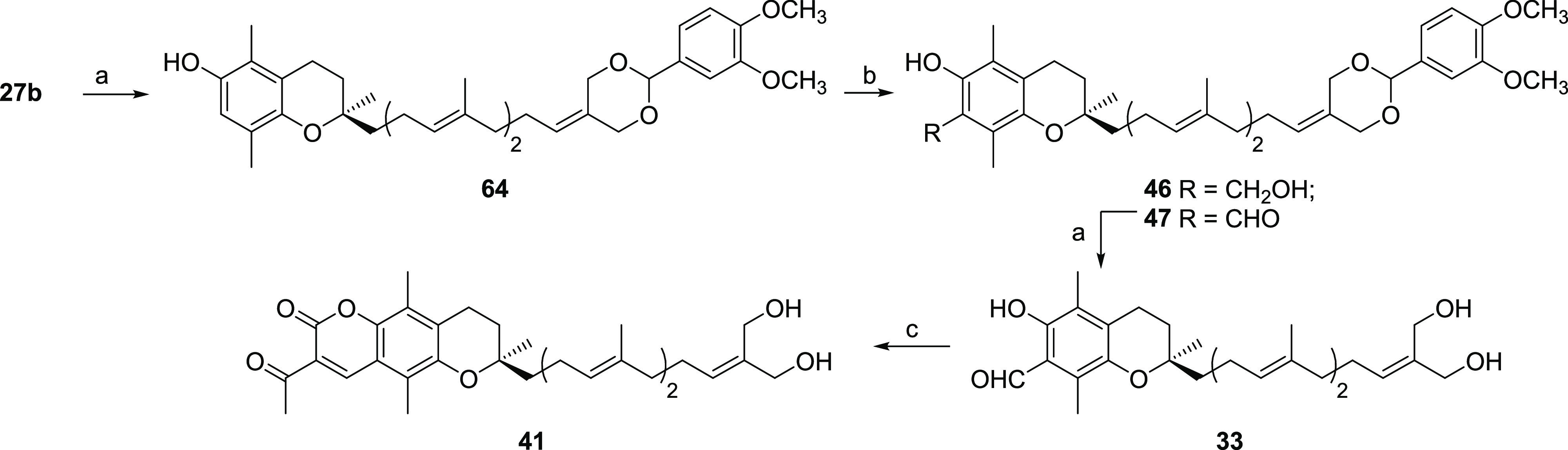
General Preparation of **33**, **41**, **46**, and **47** Reagents and conditions: (a) see Alsabil et al.;^38^ (b) MgCl_2_, Et_3_N, (CHO)*_n_*, THF, reflux, 33% for **46**, 36% for **47**; (c) ethyl acetoacetate, piperidine, EtOH, reflux, 89%.

**Scheme 4:**
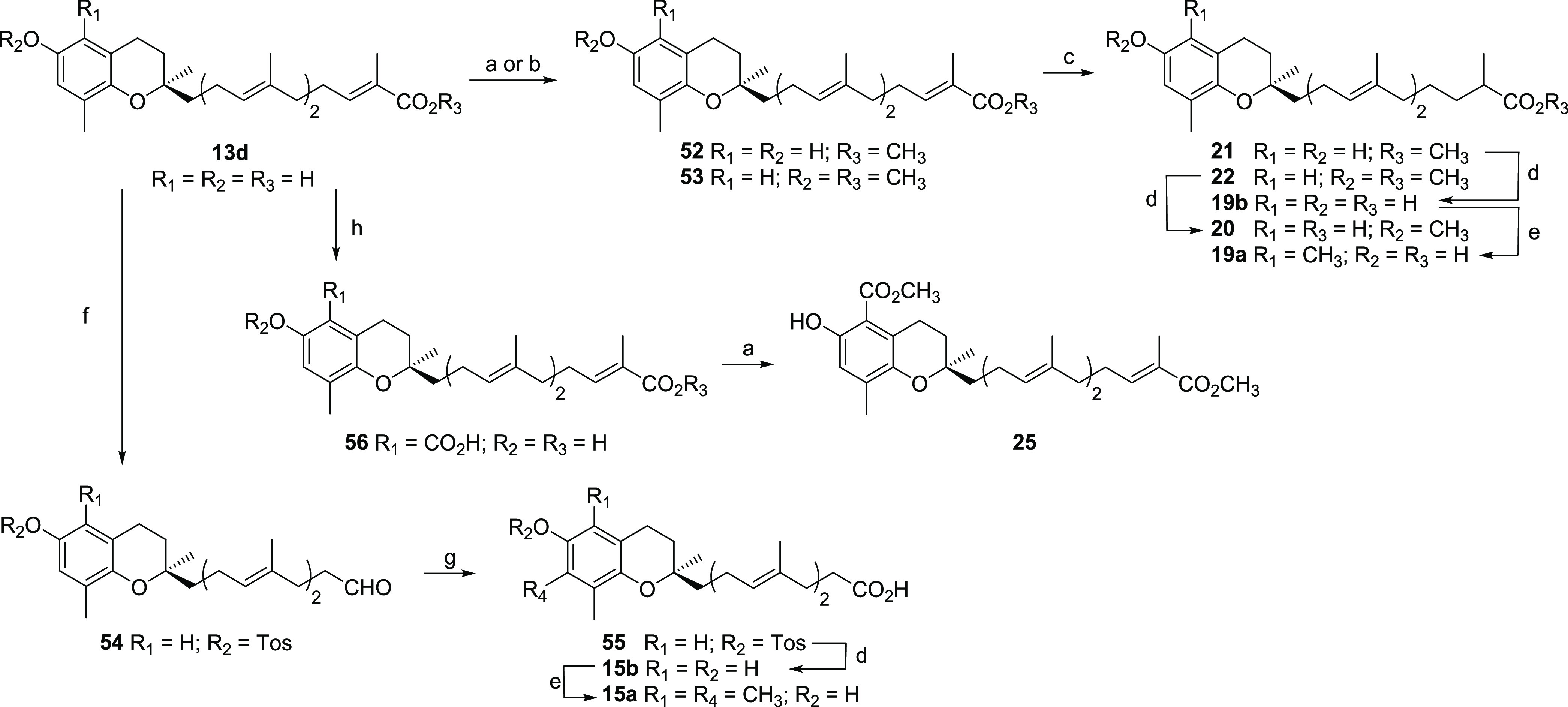
General Preparation of **15a**, **15b**, **19a**, **19b**, **20**–**22**, and **25** Reagents and conditions: (a) MeI, NaHCO_3_, DMF, MW 120 °C, 45 min, 93% for **52**, 85% for **25**; (b) NaH, MeI, DMF, 0 °C, 1 h, 67% for **53**; (c) Mg, MeOH, rt., 16 h, 66% for **21**, 72% for **22**; (d) NaOH, MeOH, 70 °C, 4 h, 91% for **19b**, 87% for **20**, 77% for **15b**; (e) TMDA, (CHO)*_n_*, 1,4-dioxane, MW 140 °C, 40 min and then NaBH_3_CN, EtOH, reflux, 14 h, 62% for **19a**, 75% for **15a**; (f) see Ville et al.;^39^ (g) NaClO_2_, H_2_NSO_3_H, 2-methyl-2-butene, 1,4-dioxane/H_2_O (1:1), 0 °C, 2 h, quant.; (h) see Viault et al.^47^

**Scheme 5:**
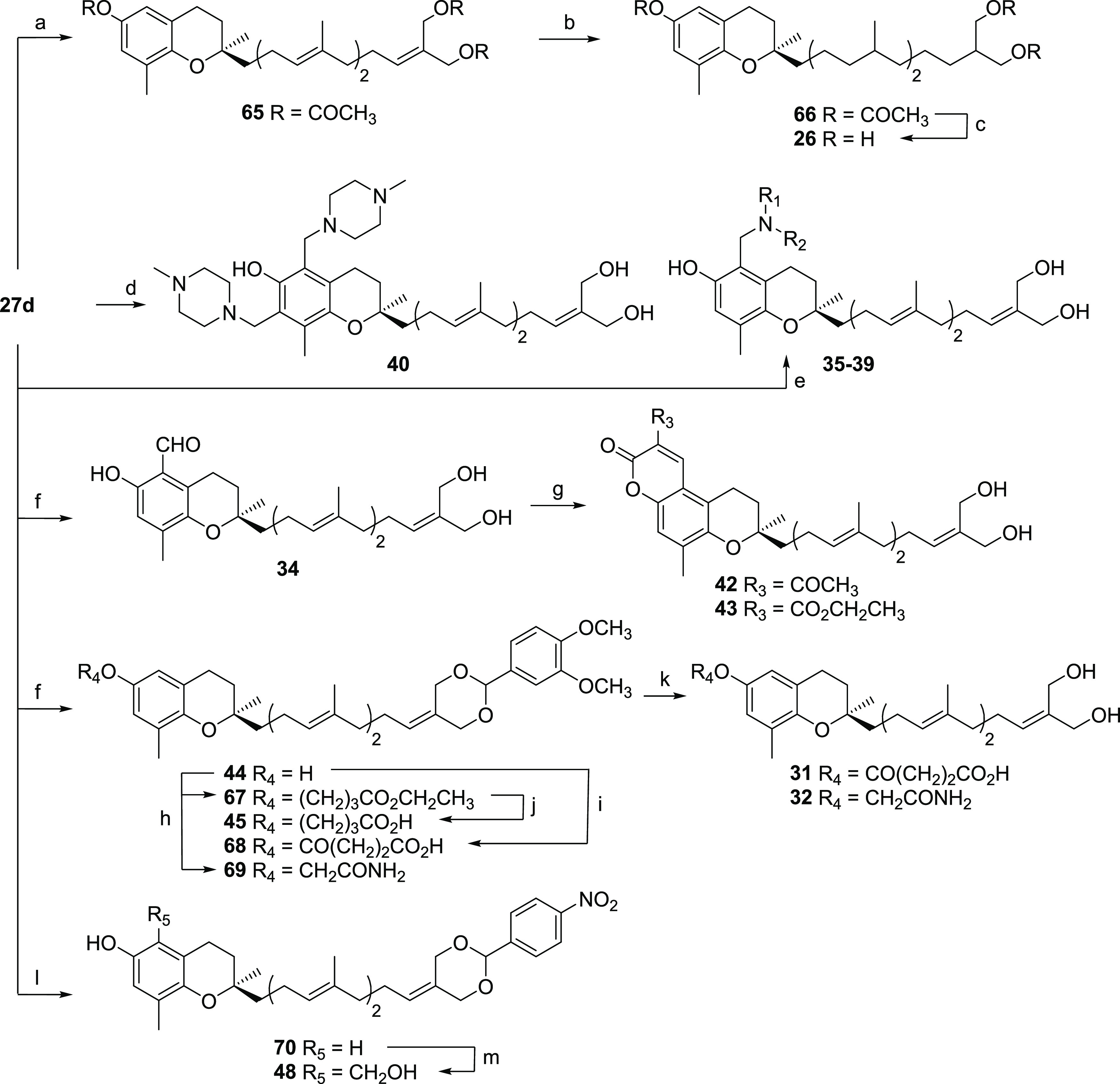
General Preparation of Amplexichromanol Analogues **26**, **31**, **32**, **35–40**, **42**–**45**, and **48** Reagents and conditions: (a) (CH_3_CO)_2_O, pyridine, rt., 24 h, 85%; (b) Pd/C 10% wt, EtOAc, rt., 40 min, 52%; (c) MeONa, MeOH, rt., 40 min, 99%; (d) (CHO)*_n_*, *N*-methylpiperazine, MeOH, reflux, 48 h, 92%; (e) (CHO)*_n_*, secondary amine [= pyrrolidine (**35**); dimethylamine (**36**); morpholine (**37**); piperidine (**38**); *N*-methylpiperazine (**39**)], MeOH, reflux, 5 h, 90% for **35**, 90% for **36**, 95% for **37**, 95% for **38**, 92% for **39**; (f) see Alsabil et al.;^38^ (g) ethyl acetoacetate (for **42**) or diethyl malonate (for **43**), piperidine, EtOH, reflux, 1–3 h, 93% for **42**, 90% for **43**; (h) ethyl bromobutyrate (for **67**) or bromoacetamide (for **69**), NaH, THF, 0 °C, 4 h, 70% for **67**, 66% for **70**; (i) succinic anhydride, Et_3_N, THF, rt., 20 h; (j) LiOH 10%, THF, rt., 3 h, 85%; (k) PTSA, THF, 1 h, 60% for **31**, 72% for **32**; (l) 4-nitrobenzaldehyde dimethyl acetal, PTSA, THF, reflux, 5 h, 78%; (m) H_3_BO_3_, (CHO)*_n_*, AcOH, toluene, reflux, 12 h, 55%.

Formyl derivatives are key intermediates to access a wide variety of functionalized polyphenols (*e.g*., coumarins, chalcones). Therefore, a MgCl_2_-mediated formylation was applied to vitamin E derivatives from different series (tocopherol, tocotrienol, garcinoic acid, amplexichromanol), leading to **2**, **3**, **7**, **8**, **33**, **34**, and **47**(38) (Scheme 2). Benzyl alcohol **46** was isolated due to an incomplete redox process (Scheme 3).^45^ C5-hydroxymethylation was also evaluated using reagents that prevent formylation such as paraformaldehyde and boric acid. Starting from **70** allowed the preparation of **48**.^46^ Compound **3** was either protected as a methoxymethyl acetal **4** or oxidized through a Pinnick oxidation leading to the corresponding carboxylic acid **5**.^47^ Similar conditions applied to aldehyde **54**(39) led to **15b** and then **15a** bearing a truncated 11-carbon long side-chain (Scheme 4). Aldehydes **33** and **34** were further involved in a condensation step with β-carbonylesters toward coumarin-tocotrienol hybrids **41**–**43** (Schemes 3 and 5).^48^

To increase the flexibility of the tail of the side chain and thus potentially improve the binding affinity of the corresponding ligands, reduction of the conjugated double bond of garcinoic acids was envisioned. Among different conditions (enzymatic, cobalt-, or copper hydride-catalyzed) reported in the literature and applied to conjugated esters or amides, the use of magnesium in dry methanol was the sole successful approach leading to **21** and **22** after a nonstereoselective three-step strategy from **13d** (Scheme 4).^49−53^ Hydrogenation of **10a**, **10c**, and **27d**, respectively, yielded **9a**, **9b**, and **26** as diastereoisomeric mixtures (Scheme 5).^54^ A similar conclusion can be drawn for **12a** and **12b**, previously prepared from **13a** and **13d**.^28^

**Scheme 6:**
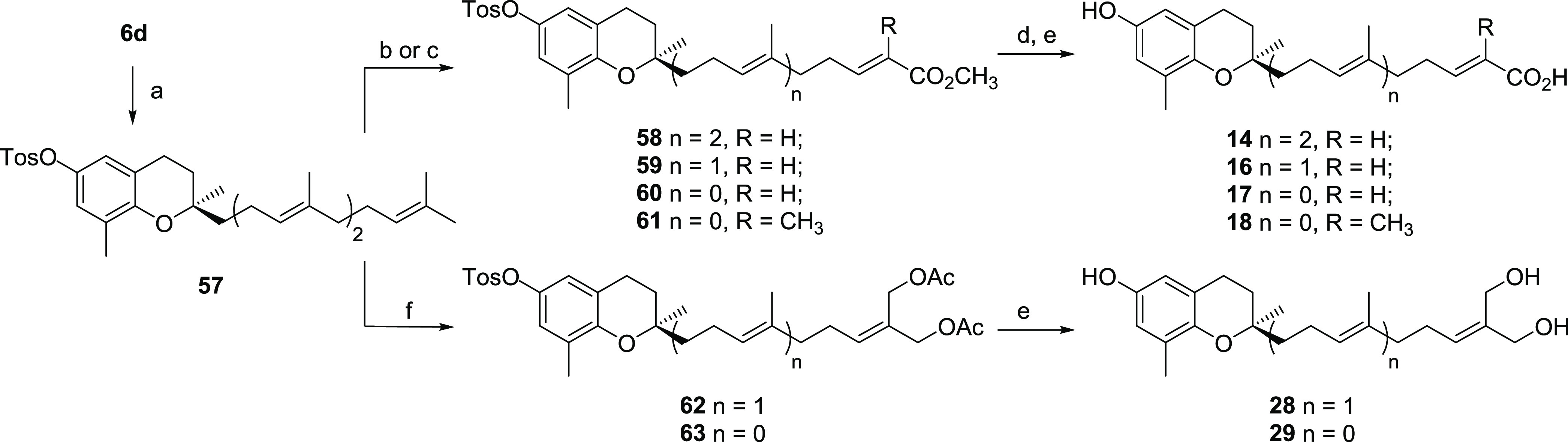
General Preparation of **14**, **16**–**18**, **28**, and **29** Reagents and conditions: (a) TosCl, Et_3_N, DCM, rt., 4 h, 60%; (b) for **58**–**60** methyl acrylate Grubbs-II catalyst, CDCl_3_, reflux, 3 days, 10% for **58**, 30% for **59**, 49% for **60**; (c) for **61** methyl methacrylate Hoveyda–Grubbs-II catalyst, CDCl_3_, MW 120 °C, 2.5 h, 21%; (d) LiOH, THF/MeOH/H_2_O (3:1:1), 40 °C, 16 h; (e) KOH, MeOH, reflux, 6 h, 16% for **14**, 31% for **16**, 17% for **17**, 20% for **18**, 53% for **28**, 57% for **29**; (f) 2-methylene-1,3-propanediacetate, Grubbs-II catalyst, CDCl_3_, MW 120 °C, 2 h, 27% for **62**, 38% for **63**.

Ruthenium-catalyzed cross-metathesis, applied to a tocotrienolic starting material bearing three double bonds along its side chain, could be considered a powerful combinatorial tool leading in one step to various tocotrienol analogues with shorter side chains. The efficiency of cross-metathesis is known to be dependent on the substitution and the electron density of the alkene reagents. In the present study, three ruthenium catalysts (Grubbs-II, Hoveyda–Grubbs-I, and Hoveyda–Grubbs-II), tolerant with electron-poor alkenes and mono- or disubstituted with various functional groups, were evaluated under different heating conditions and reaction times in deuterated chloroform.^55^ Yields were estimated through HPLC analysis of a crude aliquot from the reaction mixtures. This process confirmed that optimized conditions were both reagent- and catalyst-dependent. Therefore, experimental conditions vary for the metathesis reactions involving tosylated δ-tocotrienol **57** and methyl acrylate, methyl methacrylate, or 2-methylene-1,3-propanediacetate. Methyl methacrylate was employed to mimic the substitution pattern similar to the garcinoic acid series (Scheme 6). Methyl acrylate led to desmethyl analogues of **13d** with various side-chain lengths. The third aforementioned alkene reagent, 2-methylene-1,3-propanediacetate, helped prepare amplexichromanol analogues. Eventually, **14**, **16**–**18**, **28**, and **29** were synthesized in our group through a cross-metathesis approach^56^ similar to the one recently reported by Gujarathi et al.^57^ Tosyl ester was used as a protecting group of the phenol function rather than a silylated ether. This choice initially aimed at achieving one final deprotection step of all protecting groups. Practically such a strategy was successfully applied to the syntheses of **28** and **29** with the parallel removal of acetates and tosylate groups of **62** and **63**. On the other hand, a two-step deprotection sequence was employed to access **14**, **16**, and **17**.

Mannich bases, a chemical class with a wide structural variety, exhibit a broad spectrum of biological activities, including antitumoral, anti-inflammatory, antimicrobial, and antiviral properties.^58,59^ Besides obtaining structural insights from docking studies, we considered optimizing the physicochemical properties of pharmacologically relevant candidates. Water solubility may be further enhanced by adding a protonable group. Therefore, aminomethylation through the Mannich reaction was explored. This strategy has already been applied to **6d** to provide C5-aminomethylated analogues with an antitumoral potential.^46^ In the current study, several mono- and bis-Mannich bases have been prepared either in the garcinoic acid (**23**,^32^**24**^6^) or in the amplexichromanol (**35**–**40**) series following classical experimental conditions (Scheme 5).

Alkylation of the phenol function was potentially associated with a loss of 5-LOX binding affinity for the corresponding tocotrienolic ethers.^6^ Based on reported synthesis methods for the development of redox-silent antitumoral vitamin E analogues, two ethers (**32**, **45**)^60^ and one ester (**31**)^61^ bearing both a hydrogen-bond donor and acceptor with different linker lengths were semisynthesized and evaluated (Scheme 5).

### Structural Requirements for 5-LOX Inhibition

SAR studies on chromanols as 5-LOX inhibitors were performed both for the isolated human recombinant enzyme (cell-free assay) and in activated human polymorphonuclear leukocytes (PMNL, cell-based assay). ω-Oxidation of tocopherols (**1a**–**d**) and tocotrienols (**6a**–**d**) at the 16-carbon side chain led to potent 5-LOX inhibitors with a 13′-hydroxy or 12a′-hydroxymethyl group (**9a, 9b, 10a–e**),^6^ carboxylic acid (**12a, 13a–d**),^6^ carboxamide,^42^ or methoxycarbonyl substituent (**21**) (Tables 1 and S1, Schemes S1 and S2). The 5-LOX inhibitory potency was modulated by methyl substitution at the chromanol core, although not always in the same direction for tocopherols (**1a–d**), tocotrienols (**6a–d**), and derivatives with the modified side chain.^6^ Thus, **13d** is the most potent C13′-carboxylic acid to inhibit 5-LOX, whereas ω-alcohols (**9a, 9b, 10a–c**) are slightly less potent but also less restricted to side-chain unsaturation (**9a***vs***10a, 9b***vs***10c**), the site of ω-oxidation (C13′ or 12a′; **10d, 10e***vs***13e**), and chromanol methylation (*e.g*., **10a***vs***10c** in comparison to **13a***vs***13d** for the 5-LOX enzyme).^6^ Dihydroxylation of one double bond within the side chain was detrimental (**27d***vs***30**) (Tables 2 and S1, Schemes S1 and S2).

**Table 1:**
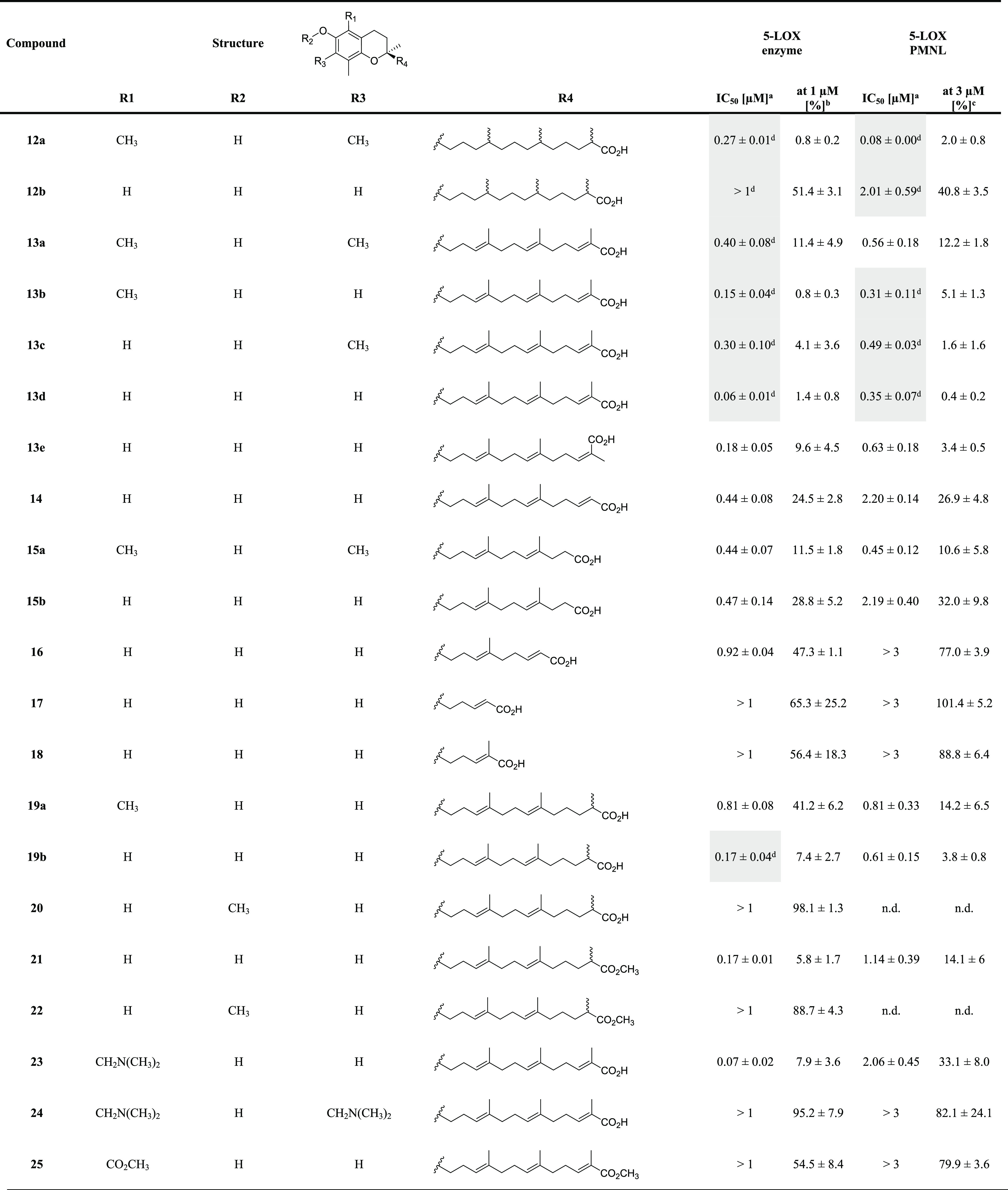
Inhibition of Human Isolated 5-LOX and 5-LOX Product Formation in Activated PMNL by Garcinoic Acid-Derived Compounds (**12a**–**25**)^e^

a IC_50_ values (μM).

b Residual activities (% control) at 1 μM compound concentration.

c Residual activities (% control) at 3 μM compound concentration.

d Highlighted data (gray) are from Pein et al.^6^ n.d., not determined.

e All values are given as mean ± standard error of the mean (SEM) of single determinations obtained in 3–6 independent experiments.

**Table 2:**
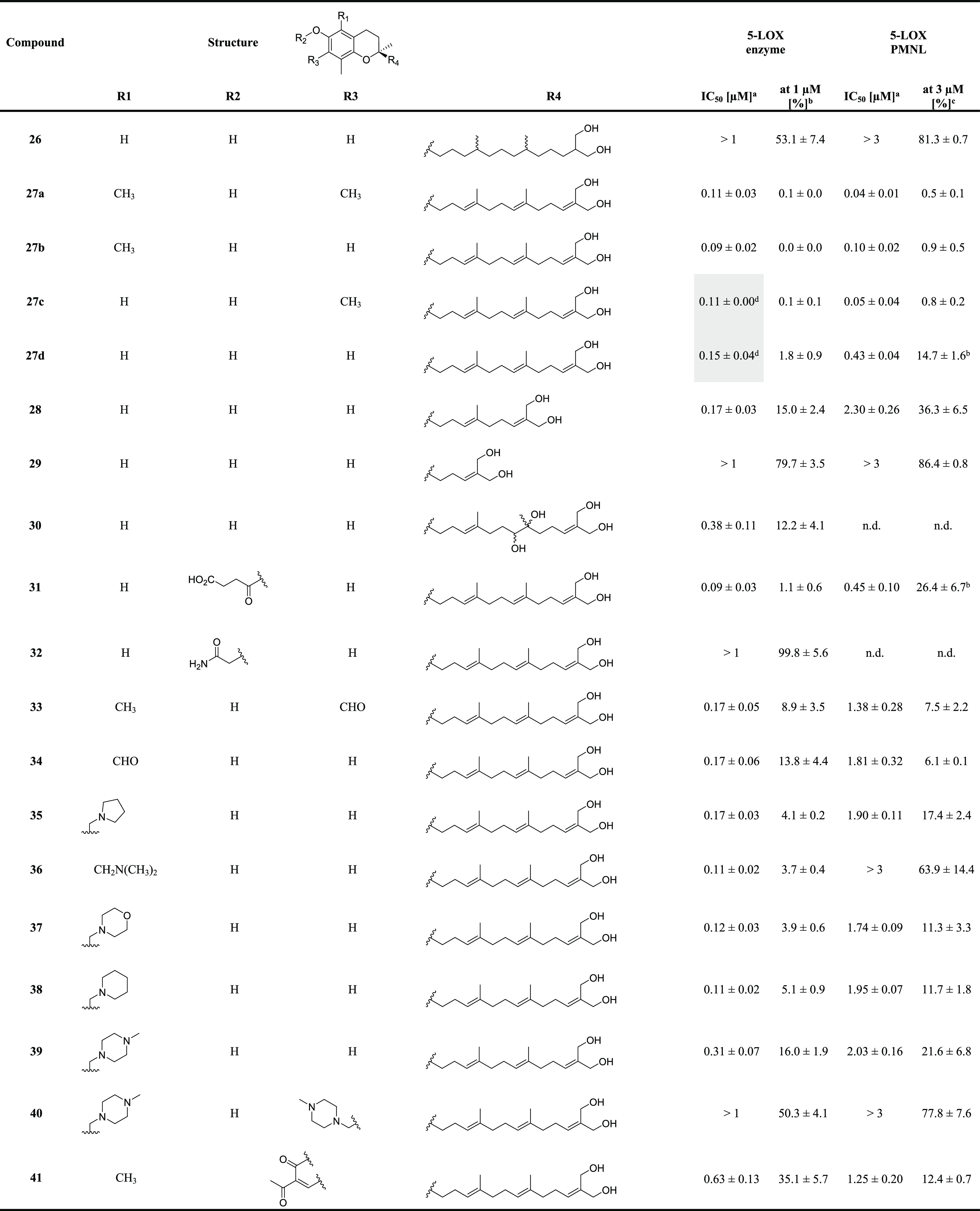

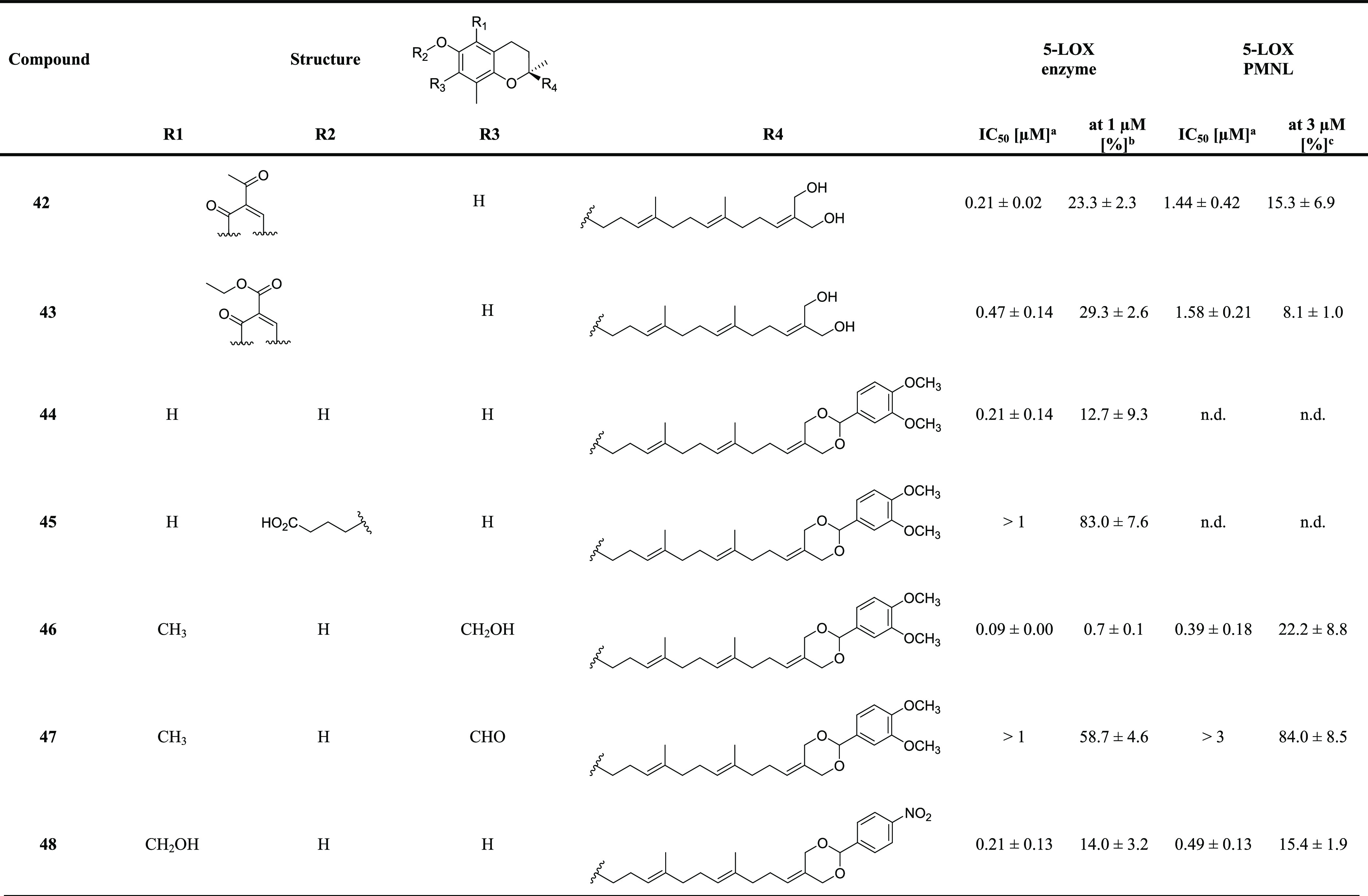
Inhibition of Human Isolated 5-LOX and 5-LOX Product Formation in Activated PMNL by Amplexichromanol-Derived Compounds (**26–48**)^e^

a IC_50_ values (μM).

b Residual activities (% control) at 1 μM compound concentration.

c Residual activities (% control) at 3 μM compound concentration.

d Highlighted data (gray) are from Pein et al.^6^ n.d., not determined.

e All values are given as mean ± SEM of single determinations obtained in 3–4 independent experiments.

We have shown that immune cells strongly accumulate LCMs, with the endogenous vitamin E metabolite **12a** being more efficiently enriched than **13d**.^6^ As a consequence, **12a** suppressed 5-LOX product biosynthesis in PMNL better than **13d**, which was the more potent enzymatic 5-LOX inhibitor (Table 1, Schemes S1 and S2). This gain of potency in PMNL was limited to side-chain saturated derivatives and not evident for the respective α-garcinoic acid **13a**. Together, substantial differences in the SARs of 5-LOX inhibition exist between cell-free and cell-based assays. The challenge was to enhance 5-LOX inhibition while maintaining an efficient uptake of the vitamin E-inspired compounds into immune cells.

Starting from **13a–d** as promising leads, we focused on the length and functionalization of the side chain and explored options for chromanol substitution. Both inhibition of cell-free 5-LOX activity and the biosynthesis of 5-LOX products in activated PMNL were addressed (Table 1, Schemes S1 and S2). In a first step, we varied the side-chain length (**14**, **15a**, **15b**, **16**) and found that inhibition of 5-LOX activity consistently decreased with shorter chain length within the δ-series (**14**, **15b**, **16**), both in cell-free and in cell-based assays. Side-chain shortening of the α-derivative **13a** (from C13 to C11) yielding **15a** was instead tolerated. We then addressed side-chain methylation, saturation, and chain length and found that 5-LOX inhibition was impaired for the desmethyl analogue at the C12 position (**14)** as well as by saturation of the Δ11′,12′ double bond (**19a, 19b**) and absent when truncated to C5 side chains (**17, 18**). Together, the natural LCM **13d** excels in 5-LOX inhibition within this series of side-chain scaffold modifications.

Next, we investigated whether ω-oxidation at both C13′ and C12a′ potentiates 5-LOX inhibition. In fact, 12a′/13′-dihydroxylated tocotrienol analogues (**27a–c**) belonging to the amplexichromanol series inhibited cell-free 5-LOX (**27a**: IC_50_ = 0.11 μM) better than ω-carboxylic acids (**13a–c**), except for the δ-derivative **27d**, which was less potent than **13d** (Tables 1 and 2, Schemes S1 and S2). We hypothesize that the methylation pattern of the chromanol core (as found in natural vitamin E forms) impacts cellular uptake and thus 5-LOX inhibition. In support of this hypothesis, the inhibitory activity of α-amplexichromanol **27a** further increased in PMNL (IC_50_ = 0.04 μM), thereby exceeding the potency of the endogenous metabolite **12a** (IC_50_ = 0.08 μM).^6^ The β- and γ-analogues **27b** and **27c** maintained their activity in the cell-based assay, whereas δ-amplexichromanol **27d** was less effective, which again highlights the strong influence of chromanol substitution on cell-based 5-LOX product formation. Shortening to C9 (**28**) or C5 (**29**) side chains was detrimental, especially in the cell-based assay, and also side-chain saturation of **27d** yielding **26** was not tolerated. Ring closure of the 12a′,13′-diol to substituted 1,3-dioxans (**44–48**) was explored because we previously found that bulky substituents, even without the hydrogen bridge donor, are tolerated in the ω-position.^6,42^ Some of the obtained 1,3-dioxanes potently inhibited cell-free 5-LOX although they were less effective in suppressing 5-LOX product formation in PMNL than **27a**. Together, compound **27a** carries an optimized side chain that qualifies for potent 5-LOX inhibition in cell-free and cell-based assays, thereby fulfilling the structural requirements for efficient 5-LOX binding and cellular accumulation.

We concentrated our further efforts on the chromanol moiety. Molecular docking studies suggest that the chromanol hydroxyl group is critical for the interaction with 5-LOX.^6^ In fact, *O*-methylation (**20, 22**) abolished 5-LOX inhibition by the ω-carboxylic acids **19b** and **21**, and ring closure as coumarin (a scaffold found in potent 5-LOX inhibitors^62^) under formation of an annulated 2-oxobenzopyran (**41**) was also disadvantageous (Tables 1 and 2, Schemes S1 and S2). Accordingly, replacement of the phenolic alcohol by an aminocarbonylmethoxy group (**32**) to increase the distance between the chromanol core and the hydrogen-bond donor/acceptor function was associated with a loss of inhibitory potency. On the other hand, 5-LOX inhibition was slightly enhanced in the cell-free assay by introducing a free carboxylic acid group as the *O*-succinyl ester (**31**). In activated PMNL, **31** inhibited 5-LOX comparably to **27d**, potentially because the ester in **31** is intracellularly cleaved to the free hydroxyl group of **27d**.

We then investigated whether also oxidative modifications at the chromanol core lead to potent 5-LOX inhibitors, as yielded by ω-oxidation of the unsaturated side chain. However, neither oxidation of the C5 or C7 methyl groups to an aldehyde (**3**, **4**, **7**) or carboxylic acid (**5**) nor additional halogenation (**2**, **8**) had any improving effect (Table S1, Schemes S1 and S2). The replacement of the C5 or C7 methyl groups by an aldehyde (**33**, 3**4**) or hydroxymethyl moiety (**46**, **48**) was, on the other hand, compatible with potent 5-LOX inhibition when combined with ω-oxidation of the side chain. Motivated by this finding, we explored a variety of structurally diverse substituents. Introduction of a methyl ester residue at the C5 methyl group of **13d** in combination with methylation of the 13′-carboxylic function was detrimental (**25**), whereas basic tertiary amines and N-heterocycles yielded inhibitors of cell-free 5-LOX that were comparably potent to the nonsubstituted derivative (for pyrrolidino, **35**; for dimethylamino, **23, 36**; for morpholino, **37**; or for piperidinyl substitution, **38**). An additional more distant nitrogen atom (*N*-methyl-piperazinyl, **39**) was detrimental, and substitution of both C5 and C7 positions with dialkylated aminomethyl groups (**24**, **40**) was not tolerated. While substituents at C5 and C7 of chromanol provide limited options to improve 5-LOX inhibition, all of these modifications substantially impaired the suppression of 5-LOX product biosynthesis in PMNL (Tables 1 and 2, Schemes S1 and S2), and we conclude that the natural chromanol moiety of vitamin E already represents an evolutionally optimized compromise between potent 5-LOX inhibition and access to the enzyme in innate immune cells. The 12a′,13′-dihydroxylated α-tocotrienol **27a** combines these features and was selected as a promising drug candidate for further pharmacological studies.

**Figure 1:**
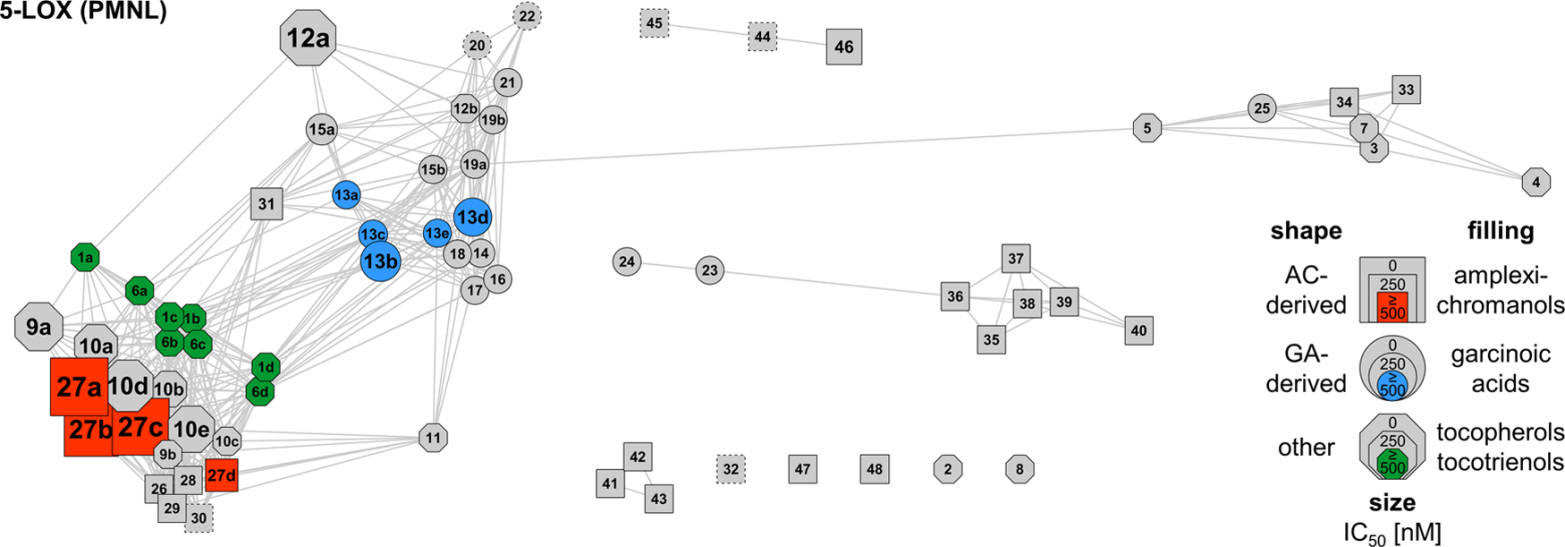
Correlation network of the compound library for inhibition of 5-LOX in PMNL. The network visualizes structural similarity between compounds calculated using Tanimoto similarity. Nodes represent individual compounds, and connecting edges represent Tanimoto coefficients > 0.9. The node shape differentiates between derivatives derived from amplexichromanols (AC), garcinoic acids (GA), or other leads, and the filling highlights the parental series, *i.e*., amplexichromanol (red), garcinoic acid (blue), tocopherol, and tocotrienol (green). The node size reflects the potency (IC_50_ values) of the compound to inhibit 5-LOX product formation in PMNL. For nodes with dotted lines, IC_50_ values were not determined.

Figures 1 and S1 summarize the SARs for 5-LOX inhibition in a network that was calculated based on the structural similarity of the derivatives, with the size of the symbols indicating their potency in cell-free (Figure S1) and cell-based assays (Figure 1).

### Mechanism of 5-LOX Inhibition and Binding Mode

**Figure 2:**
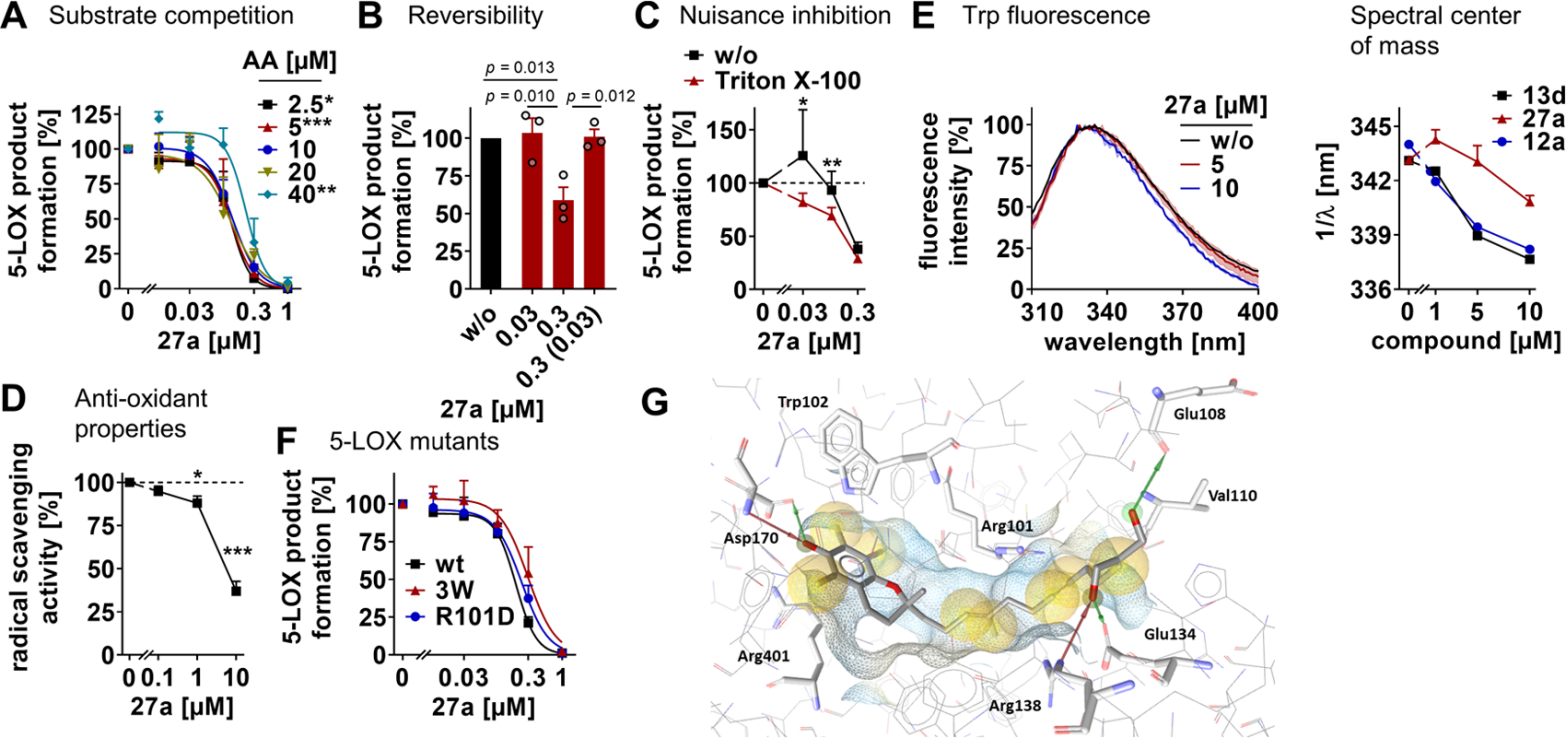
Molecular insights into 5-LOX inhibition by **27a**. (A) Effect of the arachidonic acid (AA) concentration on 5-LOX inhibition by **27a**. (B) Reversibility of 5-LOX inhibition by **27a**. Samples were preincubated with a vehicle or compound for 15 min, 10-fold diluted, and incubated for another 5 min before AA was added. The number in brackets indicates the diluted compound concentration after preincubation. (C) Effect of Triton X-100 (0.01%) on 5-LOX inhibition by **27a**. (D) Scavenging of DPPH radicals by **27a**. (E) Fluorescence excitation spectra as a percentage of maximum fluorescence intensity (left panel) and spectral center of mass of the fluorescence emission variations (right panel) shown for 5-LOX titrated with **13d**, **27a**, or **12a**. (F) Effect of **27a** on the inhibition of wild-type 5-LOX (wt), the triple mutant Trp13Ala, Trp75Ala, and Trp102Ala 5-LOX (3W), and the single mutant Arg101Asp 5-LOX (R101D). (G) Molecular docking pose of **27a** in the allosteric binding site of 5-LOX showing the interaction with Asp170, Arg138, Glu134, and Glu108. Hydrogen bonds are shown as red (HBA) and green (HBD) arrows and hydrophobic contacts as yellow spheres. Data are expressed as mean ± SEM (A–D, F) with single values (B) or mean ± SEM (transparent area) from *n* = 2 (E), *n* = 3 (A–C), and *n* = 4 (D, F) independent experiments. *p < 0.05, **p < 0.01, ***p < 0.001 *vs* 20 μM AA (A), control (D), absence of Triton X-100 (C), wt 5-LOX (F), or as indicated (B); two-way ANOVA + Tukey’s *post hoc* test (A, F), RM one-way ANOVA + Tukey’s *post hoc* test (B, D), two-tailed paired *t*-test of log data (C).

Compound **27a** inhibited human recombinant 5-LOX independent of the substrate concentration (Figure 2A) in a reversible manner (Figure 2B). Nonspecific inhibition through detergent-sensitive colloidlike aggregates was excluded by supplementing the detergent Triton X-100, which did not substantially impair 5-LOX inhibition by **27a** (Figure 2C). Moreover, the concentrations of **27a** that exhibit antioxidative properties exceed those that effectively inhibit 5-LOX, as measured by radical scavenging (Figure 2D).

We have recently shown for **13d** that it binds 5-LOX close to Trp102, Trp13, Trp75, and Arg101 at the interface of the catalytic and regulatory C2-like domain.^6^ To investigate whether **27a** targets the same site, we monitored the intrinsic Trp fluorescence of human 5-LOX. The spectral center of mass was substantially shifted to a shorter wavelength by both **13d** and the endogenous vitamin E metabolite **12a** (Figures 2E, S3A, and S3B), which indicates an altered chemical environment of Trp following ligand binding.^63^ This trend was also evident for **27a**, but the wavelength shift was less pronounced (Figure 2E). We conclude that the 5-LOX binding pose of **27a** is similar but not identical to the ω-carboxylic acids **12a** and **13d**. Site-directed mutagenesis studies strengthen our hypothesis. The inhibitory activity of **13d** is sensitive to Trp102Ala/Trp13Ala/Trp75Ala triple mutation and Arg101Asp replacement,^6^ whereas these mutations failed to affect 5-LOX inhibition by **27a** (Figure 2F).

Molecular docking studies confirm results from mutation experiments and point out poses where **13d** interacts with NH of Trp102 (H-bond with phenolic oxygen) and with Arg101 through an ionic interaction involving the ω-carboxylic acid function of **13d** (Figure S2A). Binding of **27d** within the 5-LOX allosteric site similarly involves an interaction of the phenol function with Trp102. However, at the tail of the side chain, the binding with Arg101 of the less polar allylic diol would be weaker than the one described above for the carboxylic acid group. Thus, the allylic diol exhibits multiple stabilizing interactions with various other amino acids, such as Val110 and Glu134 (Figure S2B). Site-directed mutagenesis studies showed that the 5-LOX binding affinity of **13d** depends on interactions with Trp102 and Arg101.^6^ Docking poses for **27a** suggest differences in the binding mode compared to the δ-form **27d** (Figures 2G and S2B). Despite the substitution of the chromanol by three methyl groups, the heterocycle of **27a**, as for **13d** and **27d**, still lies in the vicinity of Trp102 albeit with a shift downward. Consequently, the phenol function does not interact with Trp102 but it exhibits two hydrogen bonds with Asp170. This result supports the less pronounced wavelength shift observed in the fluorescence emission spectra (Figure 2E). Results from the docking studies tend to demonstrate that interactions of the phenol function are more efficient than the ones from the diol moiety to anchor amplexichromanols **27a** and **27d** in a close range of Trp102. Eventually, as highlighted by 5-LOX site-directed mutagenesis experiments (Figure 2F), a direct interaction with Trp102 is not fully required as long as the ligand strongly interacts with other spatially close amino acid residues, such as Asp170, through hydrogen bonds.

### 5-LOX Inhibition in Innate Immune Cells and Blood

**Figure 3:**
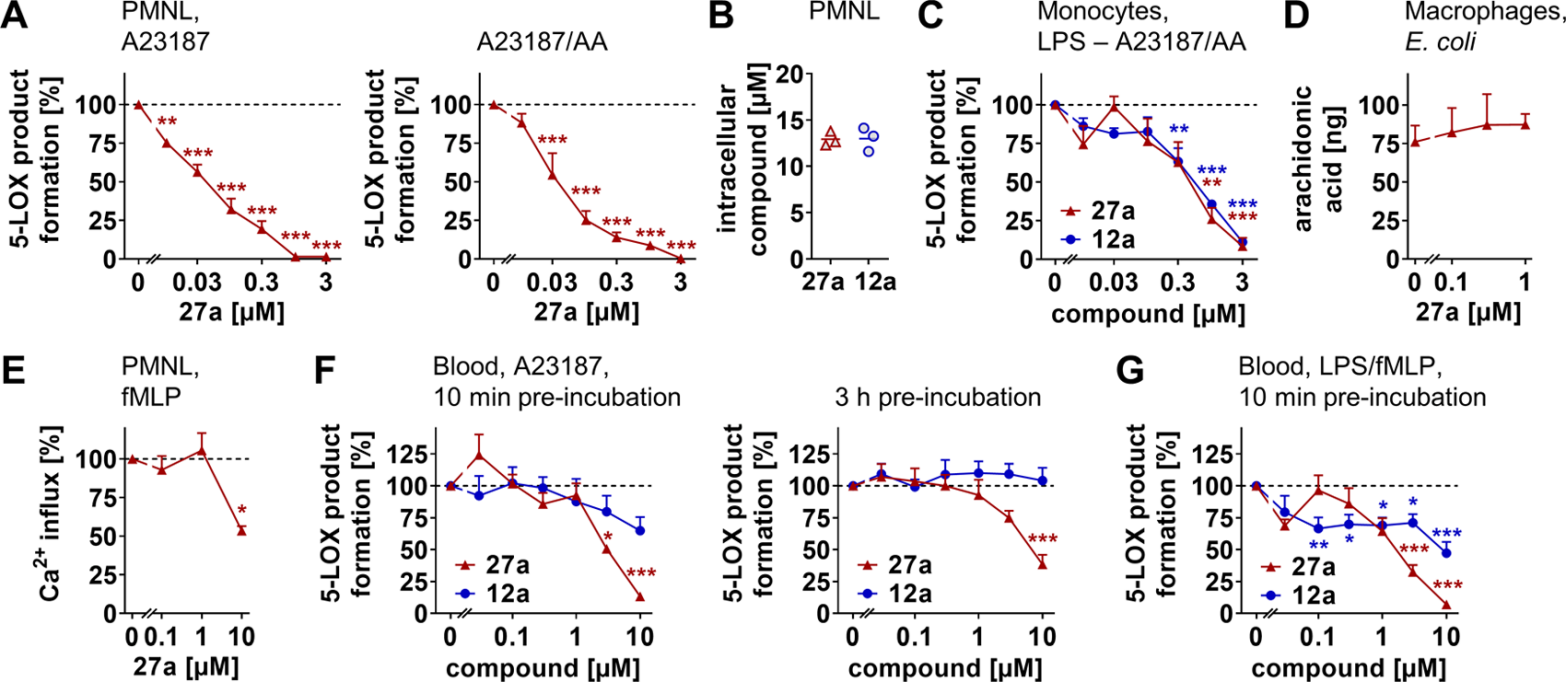
Compound **27a** accumulates in innate immune cells and inhibits 5-LOX product formation. (A) Effect of **27a** on 5-LOX product formation (LTB_4_, its isomers, and 5-H(P)ETE) in PMNL treated with Ca^2+^-ionophore A23187 or A23187 and arachidonic acid (AA). (B) Intracellular uptake of **27a** or **12a** by PMNL treated with 150 nM of the respective compound for 20 min. Average intracellular concentrations were calculated for spherical PMNL with a diameter of 13 μm. (C) Effect of **27a** or **12a** on 5-LOX product formation initiated by A23187 and AA in LPS-prestimulated monocytes. (D, E) Effect of **27a** on AA release from *Escherichia coli*-stimulated macrophages (D) and fMLP-induced Ca^2+^ influx in PMNL (E). (F, G) Effects of **27a** or **12a** on 5-LOX product formation in human whole blood stimulated with A23187 (F) or LPS and fMLP (G) upon preincubation with the compounds for 10 min (F, G) or 3 h (F). Data are expressed as mean ± SEM (A, C–G) or mean with single values (B) from *n* = 3 (A–E, F, left panel) and *n* = 4 (F, right panel, G) independent experiments. *p < 0.05, **p < 0.01, ***p < 0.001 *vs* control (A, C–G); RM one-way ANOVA + Tukey’s *post hoc* test (A, C–G).

Compound **27a** potently suppressed the biosynthesis of LTB_4_ and other 5-LOX products (*i.e*., 5-hydroxyeicosatetraenoic acid (5-HETE), LTB_4_ isomers) in PMNL (A23187 plus arachidonic acid: IC_50_ = 0.04 μM; A23187: IC_50_ = 0.05 μM) (Figure 3A), with superior potency as compared to the cell-free assay (Table 2). As a reason for this gain in 5-LOX inhibitory activity, we found that **27a** strongly accumulates in PMNL (Figure 3B), as previously described for the endogenous vitamin E metabolite **12a**, which is enriched in immune cells, both *in vitro* and in the inflamed peritoneal cavity *in vivo*.^6^ 5-LOX product formation in monocytes was instead comparably suppressed by **12a** and **27a** (IC_50_ = 0.5–0.6 μM) (Figure 3C), despite the higher potency of **27a** to inhibit 5-LOX (Tables 1 and 2).

Acute cytotoxic effects were excluded at effective concentrations of **27a** that inhibit 5-LOX (≤1 μM). Compound **27a** impaired neither mitochondrial dehydrogenase activity in peripheral blood mononuclear cells (PBMCs) (Figure S4A), an indicator of cell viability, nor membrane intactness in monocytes within 2 or 24 h (Figure S4B). At higher concentrations, **27a** induced a rapid release of lactate dehydrogenase (LDH), which was not evident for the endogenous vitamin E metabolite **12a** (Figure S4B).

Our data point toward a direct inhibition of 5-LOX in immune cells. On the one hand, the enzyme was potently inhibited in PMNL by **27a** in the presence of exogenous arachidonic acid (IC_50_ = 0.04 μM), which uncouples LT formation from the cPLA_2_-triggered release of arachidonic acid and its transfer to 5-LOX by FLAP (Figure 3A). On the other hand, **27a** neither reduced the availability of free arachidonic acid in *E. coli*-stimulated macrophages (Figure 3D) nor influenced intracellular levels of Ca^2+^, an essential cofactor for cPLA_2_α and 5-LOX activation, at submicromolar concentrations (≤1 μM) (Figure 3E). At higher concentrations of **27a** (10 μM), Ca^2+^ influx was substantially reduced, as previously reported for **13d**(6) and other ω-carboxylic acids.^64^

Lipophilic carboxylic acids like **12a** are often afflicted with strong plasma protein binding, leading to a loss of inhibitory activity in tissues and blood.^65^ In fact, the dialcohol **27a** suppressed 5-LOX product biosynthesis superior to **12a** in activated human blood, when treated with either A23187 (Figure 3F) or the physiological stimuli LPS and *N*-formyl-methionyl-leucyl-phenylalanine (fMLP) (Figure 3G). Together, compound **27a** targets 5-LOX and potently inhibits LT biosynthesis in innate immune cells and blood, thereby surpassing the endogenous vitamin E metabolite **12a**.

### mPGES-1 as a Subordinate Target within Lipid Mediator Biosynthesis

**Figure 4:**
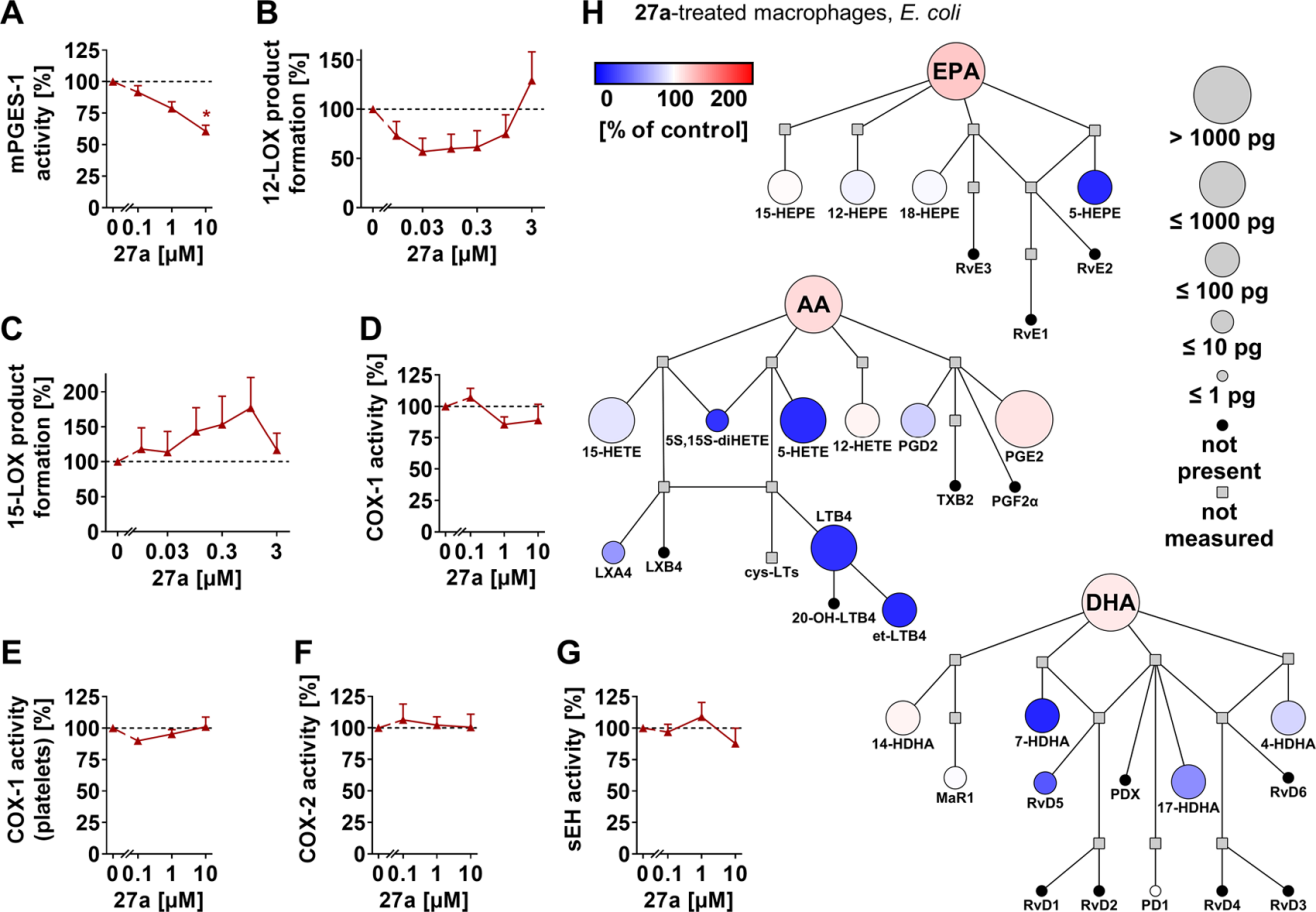
Compound **27a** moderately inhibits mPGES-1 within eicosanoid and docosanoid biosyntheses. (A–G) Effect of **27a** on mPGES-1 in microsomal preparations of IL-1β-treated A549 cells (A), 12-LOX (B), and 15-LOX product formation (C) in PMNL treated with A23187 and AA, isolated ovine COX-1 (D), COX-1-dependent 12-HHT formation in platelets (E), human recombinant COX-2 (F), and human recombinant sEH (G). (H) Quantitative illustration of the lipid mediator network of macrophages (M1) treated with **27a** (1 μM) and stimulated with pathogenic *E. coli* as compared to the vehicle control. The node size represents the mean concentration in pg, and the color intensity denotes the fold change for each lipid mediator. AA, arachidonic acid; DHA, docosahexaenoic acid; cys-LTs, cysteinyl-LTs; et-LTB_4_, LTB_4_ isomers; EPA, eicosapentaenoic acid; LX, lipoxine; MaR, maresin; 20-OH-LTB_4_, 20-hydroxy-LTB_4_; PD, protectin; Rv, resolvin; TX, thromboxane. Data are expressed as mean (H) + SEM (A-G) from *n* = 3 independent experiments. *p < 0.05 *vs* control; RM one-way ANOVA + Tukey’s *post hoc* test.

LOX, COX, and CYP_450_ monooxygenases drive the multistep conversion of polyunsaturated fatty acids into lipid mediators and work in concert with further metabolic enzymes to regulate inflammation.^13,22,66^ While some of these enzymes predominantly produce proinflammatory lipid mediators (*e.g*., mPGES-1), others additionally participate in homeostasis (*e.g*., COX-1, COX-2) or play important roles in terminating inflammation or triggering resolution (*e.g*., soluble epoxide hydrolase (sEH), 15-LOX). At micromolar concentrations (10 μM), compound **27a** decreased mPGES-1 activity (Figure 4A). Other enzymes in lipid mediator biosynthesis were not substantially inhibited: (i) 12-LOX (Figure 4B) and 15-LOX (Figure 4C) in activated human PMNL, (ii) COX-1 as the isolated ovine enzyme (Figure 4D) or within human platelets (Figure 4E), (iii) human recombinant COX-2 (Figure 4F), or (iv) sEH (Figure 4G). 15-LOX product formation was even increased by a trend at submicromolar **27a** concentrations (Figure 4C). In summary, compound **27a** exhibits superior selectivity to the endogenous vitamin E metabolite **12a**,^6^ with 5-LOX as the primary and mPGES-1 as the subordinate target.

To investigate the consequences on the lipid mediator network, we stimulated human macrophages (M1 subtype) with pathogenic *E. coli* and performed a comprehensive metabololipidomics analysis. At concentrations that do not effectively inhibit mPGES-1 (1 μM), **27a** preferentially suppressed the biosynthesis of 5-LOX-derived products, *i.e*., LTB_4_, its isomers, 5-HETE, 5,15-diHETE, 5-hydroxyeicosapentaenoic acid (HEPE), and 7-hydroxydocosahexaenoic acid (HDHA) (Figure 4H). On the other hand, PGE_2_ levels slightly increased, which we ascribed to the 5-LOX substrate arachidonic acid being channeled into PGE_2_ biosynthesis. Thus, our data indicate that the impact of **27a** on the lipid mediator profile is mainly determined by 5-LOX inhibition, as shown for macrophages (Figure 4H) and confirmed in monocytes (Figure S5).

### Superior Metabolic Stability against Side-Chain Truncation

**Figure 5:**
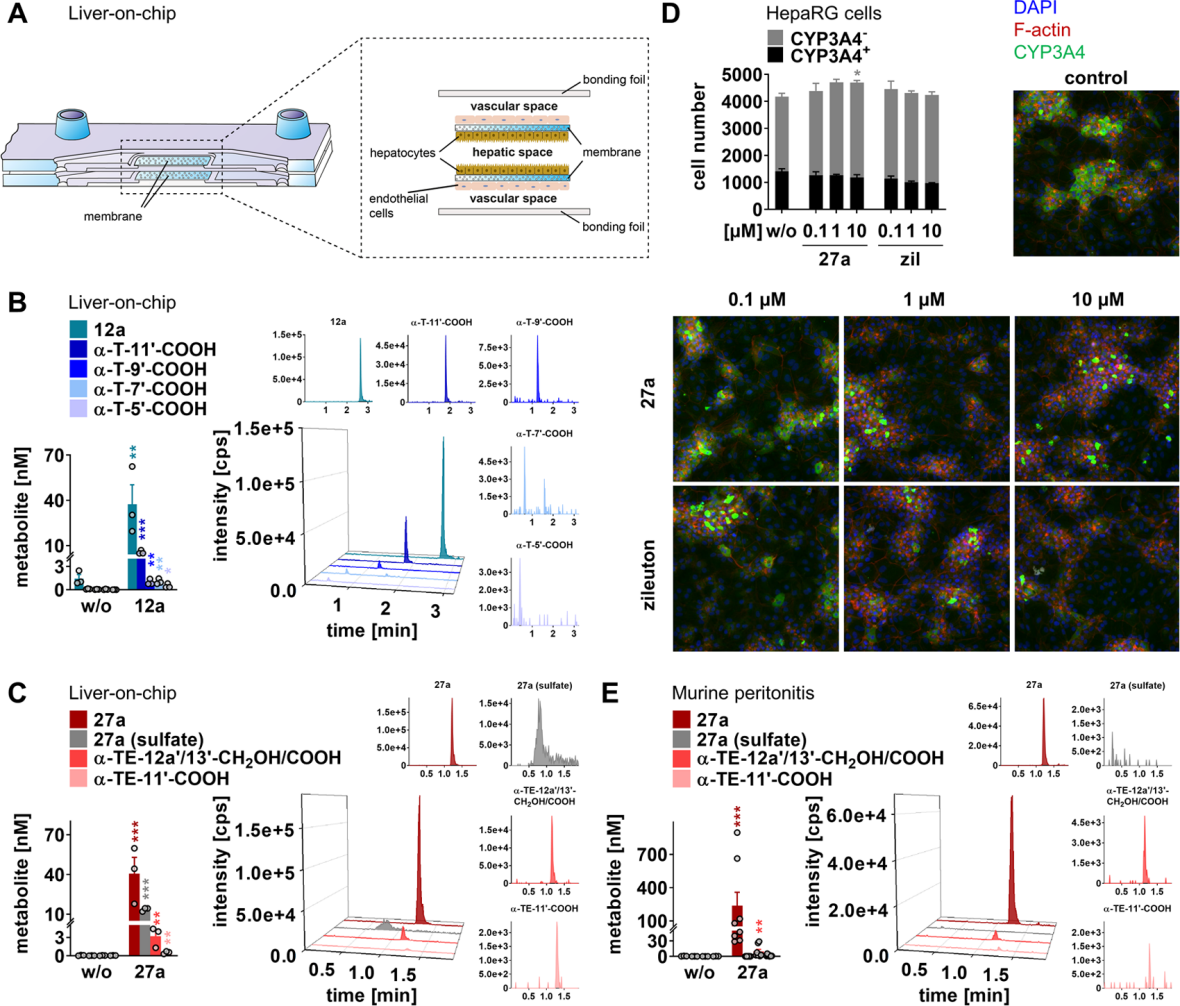
Metabolism of **27a** in a human liver-on-chip and in mice. (A) Scheme of the human liver-on-chip model. (B, C) The liver-on-chip was loaded with compound (1 μM) and incubated for 48 h. Extracted chromatograms (representative of three independent experiments) and concentrations of **12a** and metabolites (B) or **27a** and metabolites (C). (D) Effect of **27a** and zileuton (zil) on CYP3A4 expression in differentiated HepaRG cells after treatment for 48 h. Fluorescence images (20× magnification) visualize CYP3A4-expressing (CYP3A4^+^) and CYP3A4-nonexpressing cells (CYP3A4^–^) and are representative of nine (w/o) or three independent experiments. (E) Extracted chromatograms (representative of eight mice) and concentrations of **27a** and metabolites in plasma of mice with acute peritonitis 90 min post **27a** administration (10 mg/kg, p.o.). Data are expressed as mean ± SEM (D) and single values (B, C, E) from *n* = 3 (B, C, D, **27a**, and zil, E, w/o), *n* = 8 (E, **27a**), and *n* = 9 (D, w/o) independent experiments. *p < 0.05, **p < 0.01, ***p < 0.001 *vs* control; two-tailed unpaired *t*-test of log data (B, C, E), ordinary one-way ANOVA + Tukey’s *post hoc* test (D). The nomenclature used for LCMs is explained in Table S2.

We studied the hepatic metabolism of **12a** and **27a** using a human liver-on-chip.^6^ The biochip resembles a liver sinusoid and consists of a hepatic and vascular compartment, which are separated by two porous membranes that serve as cell scaffolds (Figure 5A). For the hepatic compartment, we selected HepaRG cells, which are considered to be the closest to human primary hepatocytes among liver cell lines, and differentiated them into hepatocyte-like and biliary-like cells. The endothelial layer mimics the endothelial lining of the liver sinusoid and consists of human umbilical vein endothelial cells (HUVECs).

The metabolism of **12a** and **27a** substantially differs. While **12a** is truncated to metabolites with varying side-chain lengths (Figure 5B), **27a** is preferentially conjugated with sulfate or oxidized at 12a′ or 13′ to the respective ω-carboxylic acid (Figure 5C). Truncation of the side chain was instead negligible. Despite being a substrate for hepatic ω-oxidation, **27a** did not induce CYP_450_ enzyme expression in HepaRG cells, as exemplarily studied for CYP3A4 (Figure 5D).

Next, we investigated the metabolism of **27a** after oral administration in mice. Compound **27a** was taken up within 1 h, reaching plasma concentrations (0.03–0.9 μM) that effectively inhibit 5-LOX *in vitro* (Figure 5E). We confirmed that the unsaturated chain of **27a** is resistant to degradation but did not detect sulfate conjugates (Figure 5E). We conclude that **27a** is less susceptible to side-chain degradation than the endogenous metabolite **12a** and speculate about species-specific differences in **27a** sulfation between mice and humans.

### Improved Epidermal Homeostasis in Experimental Atopic Dermatitis

**Figure 6:**
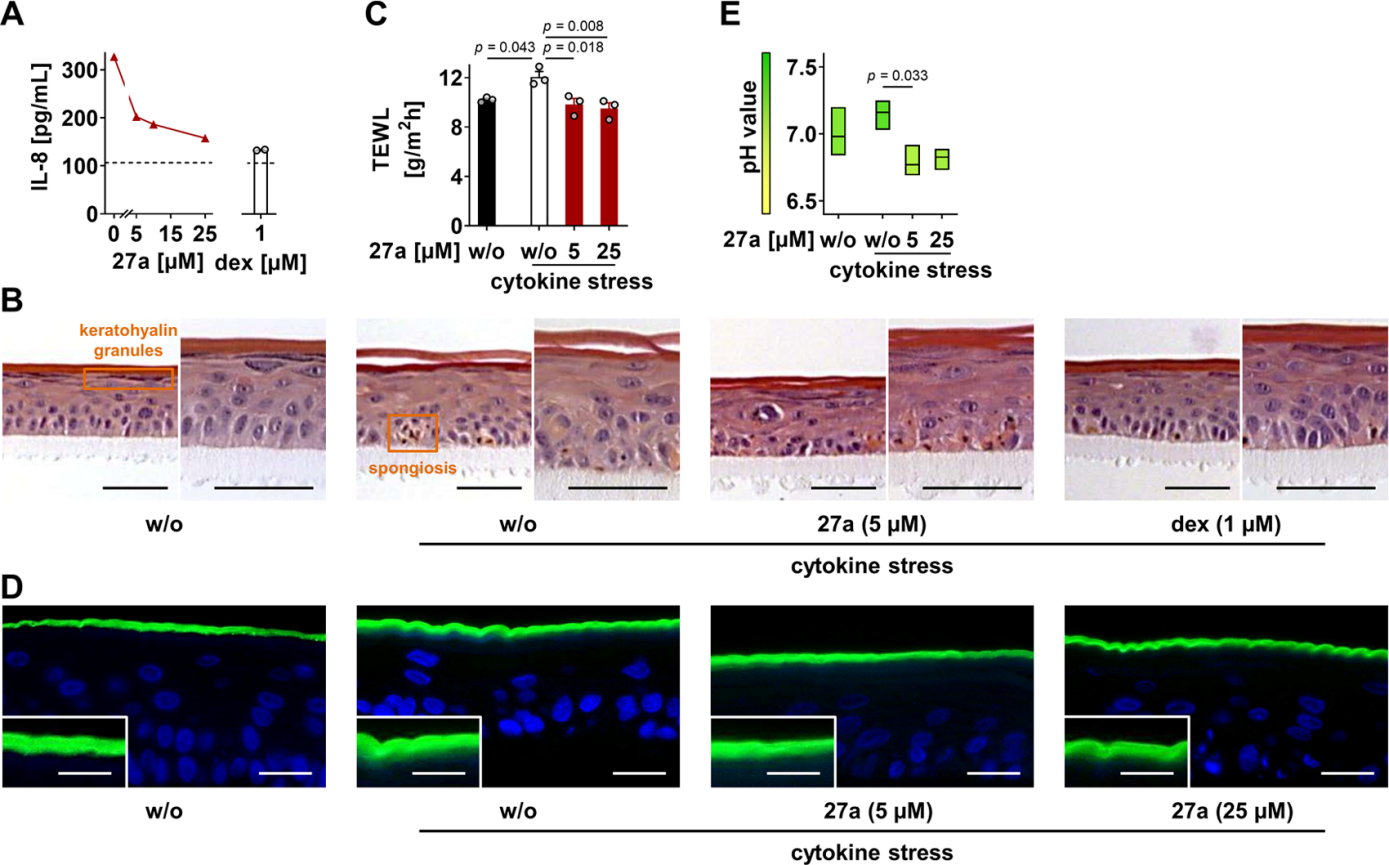
Compound **27a** relieves inflammation in reconstructed human epidermis (RHE). RHE exposed to **27a** or dexamethasone (dex) was treated with a cytokine cocktail for 4 days to trigger the inflammatory reaction. (A) Concentration of IL-8 in the growth medium. The dotted line indicates basal levels without cytokine stress. (B) Morphological changes visualized by hematoxylin and eosin staining (scale bar is 50 μm; representative of three independent experiments). (C) Transepidermal water loss (TEWL) at the RHE surface. (D) Impermeability of the stratum corneum. The stratum corneum of cytokine-stressed RHE becomes permeable for Lucifer yellow (green) that diffuses into the viable cell layers, as shown in the insets in higher magnification (scale bar for the outer box is 20 μm, and scale bar for the inset is 10 μm; representative of three independent experiments). Nuclei were stained with 4′,6′-diamidino-2-phenylindole (DAPI, blue). (E) pH value at the RHE surface. Data are expressed as mean ± SEM (A, **27a**) with single values (A, dex, C) or mean + floating bars from minimum to maximum values (E) from *n* = 2 (A) and *n* = 3 (C, E) independent experiments. Ordinary one-way ANOVA + Tukey’s *post hoc* test.

LT levels are substantially increased in relapsing inflammatory skin lesions in atopic dermatitis, as are other lipid mediators and cytokines.^67^ To study the effect of **27a** on the inflammatory processes leading to skin dysfunction, we used reconstructed human epidermis (RHE) and induced cytokine stress. Interleukin (IL)-8, an indicator of the severity of inflammation in atopic dermatitis,^68^ was upregulated (Figure 6A), as was thymic stromal lymphopoietin (TSLP) (Figure S6A), which is an IL-7-like cytokine that propagates skin lesions by mediating the recruitment and polarization of Th2-type CD4^+^ cells.^69^ Morphological and functional changes in the epidermal structure are characterized by spongiosis and the loss of keratohyalin granules (Figures 6B and S6B). The latter is associated with a diminished expression of filaggrin, one of the crucial proteins to maintain skin barrier function.^70^ Along these lines, cytokine stress triggers transepidermal water loss (Figure 6C) and allows the fluorescent dye Lucifer yellow to pass the stratum corneum (Figures 6D and S6C).

Moreover, the pH increased by trend (Figure 6E), which is believed to hamper skin barrier homeostasis, thereby interfering with the innate immune defense in atopic dermatitis.^71^ Dexamethasone, used as a control, suppressed these cytokine-induced responses, as expected.

Compound **27a** reduced IL-8 levels (Figure 6A), attenuated the epidermal disorganization induced by cytokine treatment (Figures 6B and S6B), and substantially improved skin barrier functions (Figure 6C, D and S6C). The pH was decreased even below the basal level (Figure 6E). Thus, compound **27a** limits the inflammatory reaction and supports epidermal homeostasis in experimental atopic dermatitis. On the other hand, **27a** did not influence TSLP expression in the RHE (Figure S6A), rather excluding a major impact on Th2-mediated tissue damage. Cytotoxic effects of **27a** on human keratinocytes were excluded under our experimental conditions (Figure S6D).

### Attenuated Inflammation in Murine Peritonitis

**Figure 7:**
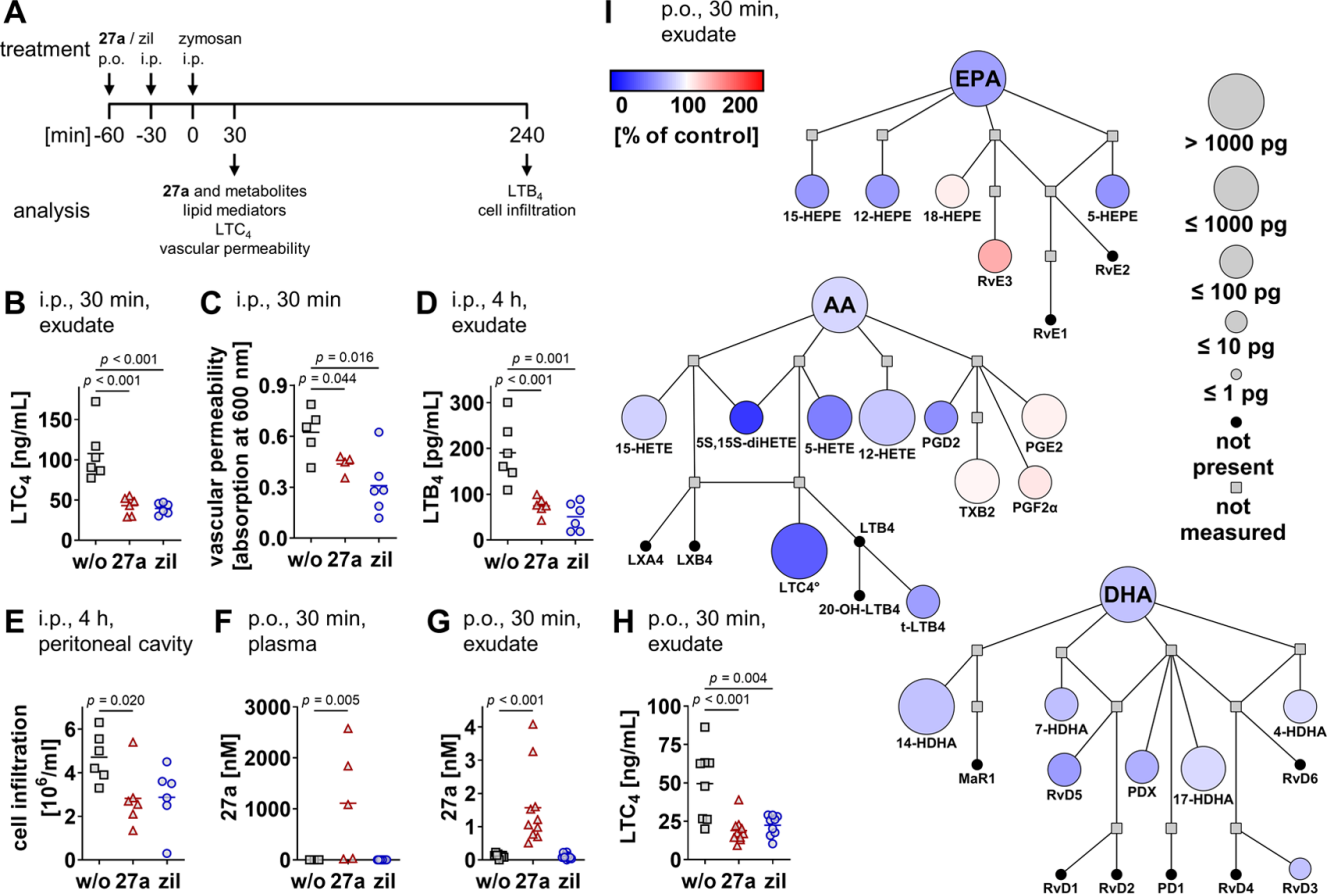
Compound **27a** inhibits 5-LOX product formation and limits inflammation in murine peritonitis. (A) Time scale for zymosan-induced murine peritonitis. (A–I) Mice received **27a** (B–E, I: 10 mg/kg; F, G: 30 mg/kg; H: 3 mg/kg; B–E: i.p.; F–I: p.o.) or zileuton (zil; B–E: 10 mg/kg; F, G: 30 mg/kg; H: 3 mg/kg; B–E: i.p.; F–H: p.o.) and were killed 30 min (B, C, F–I) or 4 h (D, E) post zymosan injection. (B, H) LTC_4_ levels in the exudate analyzed by ELISA. (C) Vascular permeability. (D) LTB_4_ levels in the exudate analyzed by ELISA. (E) Immune cell infiltration into the peritoneal cavity. (F, G) Concentration of **27a** in the plasma (F) and exudate (G). (I) Quantitative illustration of the lipid mediator network in the peritoneal exudate from mice given **27a** as compared to the vehicle. The node size represents the mean concentration in pg, and the color intensity denotes the fold change for each lipid mediator. °LTC_4_ was analyzed by ELISA, and all other lipid mediators and fatty acids were analyzed by UPLC-MS/MS. AA, arachidonic acid; DHA, docosahexaenoic acid; EPA, eicosapentaenoic acid; LX, lipoxin; MaR, maresin; 20-OH-LTB_4_, 20-hydroxy-LTB_4_; PD, protectin; Rv, resolvin; t-LTB_4_, LTB_4_ isomer; TX, thromboxane. Data are expressed as mean (I) with single values (B–H) from *n* = 3 (F, w/o), *n* = 4 (C, **27a**), *n* = 5 (C, w/o, F, **27a**), *n* = 6 (B, C, zil, D, E), *n* = 8 (H, w/o), *n* = 9 (G, w/o, H, zil, I, w/o), and *n* = 10 (F, zil, G, **27a** and zil, H, **27a**, I, **27a**) mice. Two-tailed unpaired *t*-test of log data (B–H).

The anti-inflammatory efficacy of **27a** was investigated for zymosan-induced murine peritonitis *in vivo*, which represents a model of acute inflammation that relies on LTs and other lipid mediators (Figure 7A).^72^ During the onset of inflammation, zymosan activates resident peritoneal macrophages that produce LTC_4_. This cysteinyl-LT increases vasopermeability in postcapillary venules. Compound **27a**, given i.p., strongly decreased LTC_4_ levels in the exudate (Figure 7B) and consequently reduced vascular permeability (Figure 7C). The progressive phase of inflammation is instead dominated by infiltrated neutrophils that generate the potent chemoattractant LTB_4_. Compound **27a** was also active at this stage and substantially lowered LTB_4_ levels, both in the exudate (Figure 7D) and by trend in plasma (Figure S7A). The influx of cells into the peritoneal cavity (Figure 7E), which is dominated by neutrophil infiltration,^72^ was accordingly reduced.

While oral administration of **27a** yielded plasma concentrations between 0.02 and 2.58 μM (Figure 7F), concentrations of **27a** in the peritoneal exudate were considerably lower (0.5–4 nM) (Figure 7G) but likely still in a range that inhibits 5-LOX, given the strong intracellular accumulation of **27a** in immune cells *in vitro* (Figure 3B). Cysteinyl-LTs were decreased to a basal level by **27a** at a dose of 3 mg/kg (Figure 7H). The clinically used 5-LOX inhibitor zileuton (at 3 mg/kg) did not evoke a stronger effect. Metabololipidomics profiling showed a preferential drop of arachidonic acid-derived 5-LOX products in exudates from **27a**-treated mice and a moderate reduction of various other LOX-derived metabolites (Figure 7I). The latter likely depends on the reduced availability of free polyunsaturated fatty acids. Conclusively, **27a** is an orally active 5-LOX inhibitor that potently suppresses LT formation and associated peritoneal inflammation *in vivo*.

We have recently shown that the endogenous metabolite **12a** augments the systemic concentration of resolvin E3, a specialized proresolving lipid mediator that actively terminates the resolution of inflammation.^6,22^ Although our study focused on acute inflammation and has only limited predictive power for the resolution phase, we found resolvin E3 levels already being moderately increased by **27a** at 30 min post zymosan injection in individual mice (Figure S7B). It is tempting to speculate that **27a** shares the putative proresolving potential of the endogenous lead compound.

### Diminished Airway Hyperreactivity in Experimental Asthma

**Figure 8:**
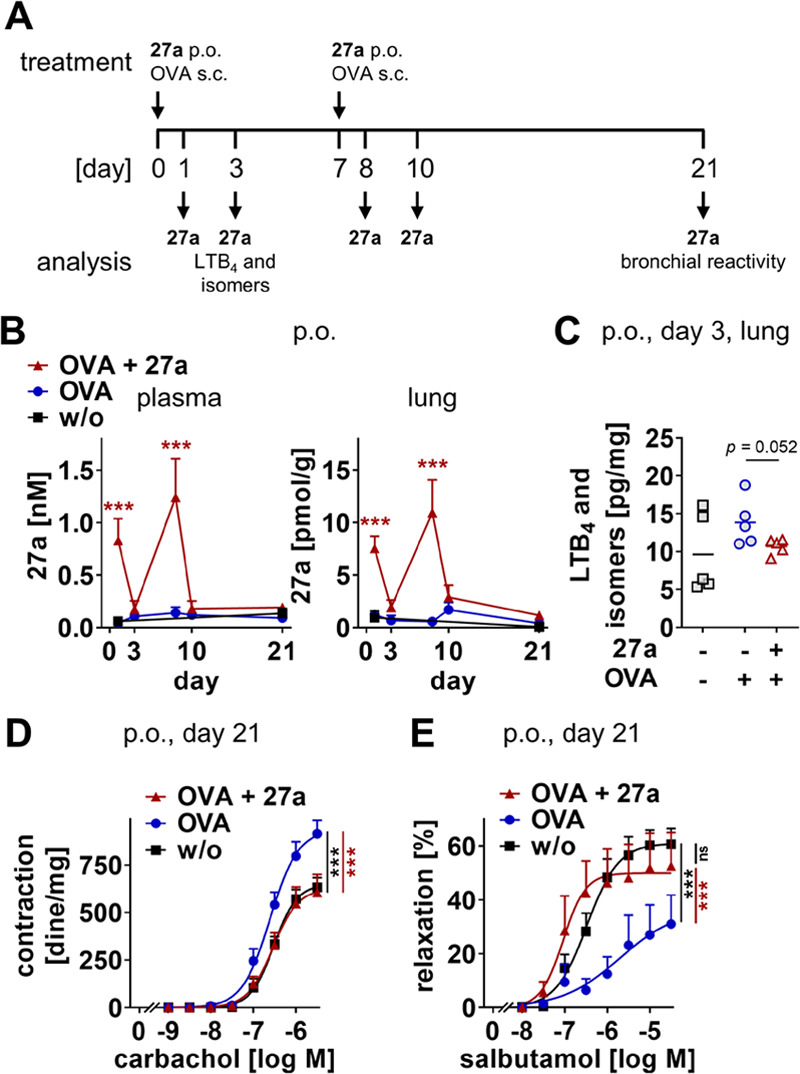
Compound **27a** suppresses bronchial hyperreactivity and pulmonary LT formation in mice sensitized to ovalbumin. (A) Time scale for the experimental asthma model. (B–E) Compound **27a** (10 mg/kg) was p.o. administered to mice prior to injection of ovalbumin (OVA) on days 0 and 7. (B) Concentrations of **27a** in plasma (nM) and lung (pmol/g lung tissue). (C) Pulmonary levels of LTB_4_ and its isomers on day 3 in pg/mg lung tissue. (D, E) Bronchial reactivity to carbachol (D) or salbutamol (E) on day 21. Data are expressed as mean with single values (C) or mean ± SEM (B, D, E) from *n* = 5 (B, days 1–10, C), *n* = 6 (B, day 21), *n* = 7 (E, OVA, OVA + **27a**), *n* = 8 (D, OVA + **27a**), *n* = 11 (D, OVA, E, control), and *n* = 12 (D, control) mice. ns, not significant; ***p < 0.001 *vs* control or as indicated; two-tailed unpaired *t*-test of log data (C), two-way ANOVA + Bonferroni’s *post hoc* test (B, D, E).

Since LTs play a pivotal role in the pathogenesis of asthma by propagating lung inflammation, immune cell infiltration, and bronchoconstriction,^15,73^ we investigated the effects of **27a** in an experimental model of asthma. Mice were treated with **27a** p.o. 60 min before being sensitized with ovalbumin on days 0 and 7 and monitored up to 21 days (Figure 8A). Systemic concentrations of **27a** peaked at the days after administration (days 1 and 8) and then rapidly declined again in the plasma and lung (Figure 8B). Gavage of **27a** lowered lung levels of LTB_4_ and its isomers (Figure 8C), blocked ovalbumin-induced bronchial hyperreactivity to carbachol (Figure 8D), and fully restored the adrenergic bronchial relaxation induced by salbutamol (Figure 8E). Together, compound **27a** is orally available and distributed to the lung, thereby effectively suppressing pulmonary LT levels and asthmatic airway contraction.

## Discussion and Conclusions

Inspired by the endogenous vitamin E metabolite **12a**, which may mediate immunomodulatory effects of vitamin E,^6,7^ we designed a novel class of potent 5-LOX inhibitors that limit inflammation. We previously explored 13′-garcinamides, which impress through their potent 5-LOX inhibitory activity,^42^ but were substantially less active in innate immune cells. Extended SAR studies revealed oxidative ω-modifications of the side chain as a superior strategy toward potent 5-LOX inhibitors that maintain their privileged access to innate immune cells. These two criteria were best realized by the C12a′-/C13′-dihydroxylated α-tocotrienol **27a**, which inhibits 5-LOX in a substrate-independent manner at concentrations that do not allow efficient radical scavenging. Compound **27a** seems to bind 5-LOX slightly displaced from **12a** and **13d** in the vicinity of Trp102, as suggested by molecular docking studies, Trp fluorescence spectroscopy, and site-directed mutagenesis. In contrast to ω-oxidation, the chromanol core and the unsaturated side chain of **27a** offer little space for structural optimization. Although we identified modifications that turn **27a** in an even more potent 5-LOX inhibitor, the pharmacologically relevant inhibition of 5-LOX product formation in PMNL was not enhanced.

Compound **27a** strongly accumulates in PMNL, reaching a comparable intracellular concentration as **12a**. Accordingly, **27a** superiorly inhibits 5-LOX product biosynthesis in activated PMNL and human blood but not in monocytes.

These cell-type-specific differences are in favor of our hypothesis that LCMs are efficiently taken up by cells through specific transport systems. Within cellular lipid mediator biosynthesis, 5-LOX is the direct and primary target of **27a**, and its inhibition shapes the lipid mediator profile of activated human macrophages and monocytes. Although **27a** is considerably more selective than **12a**,^6^ both compounds additionally inhibit mPGES-1 at high concentrations, which might be beneficial to damp the redirection of the 5-LOX substrate arachidonic acid toward proinflammatory PGE_2_ biosynthesis.

Compound **27a** is orally available and reaches plasma and lung concentrations that effectively inhibit 5-LOX *in vitro*. Rapid clearance of **27a** from the plasma is accompanied by substantial retardation in tissues, including inflamed lungs. Along these lines, **27a** is more stable against side-chain truncation than **12a**. While we did not detect **27a** metabolites in mice *in vivo* (within 90 min), **27a** was efficiently sulfated or ω-oxidized to a carboxylic acid in a human liver-on-chip (within 48 h), which might be related to the kinetics or species-specific differences in **27a** metabolism. It is tempting to speculate that both metabolites of **27a**, the sulfate and ω-carboxylic acid, possess 5-LOX inhibitory activity or, in the case of the **27a** sulfate, might be hydrolyzed to the active compound in tissues that highly express sulfatases, such as the lung and liver.^74^

We demonstrate anti-inflammatory efficacy of **27a** in murine models of peritonitis and asthma *in vivo*, for both i.p. and p.o. administration, and in experimental atopic dermatitis *in vitro* using RHE. Compound **27a** effectively suppresses LT levels along with LT-driven hallmarks of inflammation, *i.e*., immune cell infiltration, vasopermeability, and bronchial hyperreactivity.

In conclusion, we here present compound **27a**, a potent and orally active LCM-inspired 5-LOX inhibitor that shares the favorable pharmacological profile of the endogenous vitamin E metabolite **12a** but is even more potent, selective, and metabolically stable, thereby allowing an efficient suppression of inflammation *in vitro* and *in vivo*. Whether **27a** shares the proposed proresolving activities of **12a** needs further investigation.

## Experimental Section

### Isolation and Semisynthesis of Vitamin E Derivatives

^1^H and ^13^C NMR along with 2D NMR data were obtained on a Bruker Avance DRX 500 MHz spectrometer (500 and 125 MHz, respectively; BRUKER, Bremen, Germany) or a JEOL JNM-ECZS 400 MHz spectrometer (400 and 100 MHz, respectively; JEOL Ltd., Akishima, Tokyo, Japan) in deuterated chloroform, methanol, or acetone and calibrated using the residual undeuterated solvent resonance as an internal reference. IR spectra were recorded on a Thermo Scientific Nicolet iS5 FT-IR spectrometer (Thermo Scientific). Mass spectrometry analyses were performed on a JEOL JMS-700 (JEOL Ltd.) double-focusing mass spectrometer with reversed geometry, equipped with a pneumatically assisted EI or FAB source and on a BRUKER ESQUIRE 3000+ spectrometer (BRUKER) for ESI analyses in both positive and negative modes. Chromatographic analysis was performed on a Prominence-i LC-2030C (Shimadzu, Noisiel, France) equipped with a refrigerated autosampler and a column oven. The HPLC system was coupled to an evaporative light-scattering detector (ELSD SEDEX 90 LT, SEDERE). Then, 5 μL samples refrigerated at 10 °C were injected onto a Phenomenex Luna C18 column (150 mm × 4.6 mm, 5 μm) heated at 20 °C. A gradient of water with 0.1% formic acid (A) and acetonitrile with 0.1% formic acid (B) was applied (65% B for 1 min, 65–77% B within 4 min, 77% B for 8 min, 77–100% B within 5 min, 100% B for 6 min) at a flow rate of 1 mL/min. ELSD experiments were performed at 50 °C, and nitrogen was used as the nebulization gas (3.5 bar). Data were acquired and processed with LabSolutions Software (Shimadzu, Noisiel, France). Spectra for selected vitamin E analogues (**13a**, **13d**, **27a**, **27d**) are shown in Figures S57–S60. Purity determined using ELSD experiments was ≥95%. Chromatographic separations, such as flash chromatography, were performed on IntelliFlash 310 (Analogix) using a silica gel column Chromabond flash RS column (Macherey-Nagel). Reactions under microwave irradiations were performed using an Anton Paar Monowave 300 microwave reactor (Anton Paar). All reactions under microwave irradiations were performed using the stirring option in borosilicate glass vials of 10 or 30 mL (G10 or G30) sealed with PTFE-coated silicone septa (at the end of the irradiation, cooling of reaction mixtures was realized by compressed air). The microwave instrument consists of a continuous focused microwave power output from 0 to 600 W. The target temperature was reached with a ramp of 3 min, and the chosen microwave power was maintained at a constant to hold the mixture at this temperature. The reaction temperature was monitored using a calibrated infrared sensor, and the reaction time included the ramp period. The microwave irradiation parameters (power, temperature, and time) were monitored by the Monowave software package.

#### (*R*)-2-((3*E*,7*E*,11*Z*)-13-Hydroxy-4,8,12-trimethyltrideca-3,7,11-trien-1-yl)-2,8-dimethylchroman-6-yl-4-methylbenzenesulfonate (**49**)

To a solution of δ-(*Z*)-deoxyamplexichromanol **10e** (152 mg, 0.36 mmol, 1 equiv) in dichloromethane (5 mL) were added 4-toluenesulfonyl chloride (75 mg, 0.39 mmol, 1.1 equiv) and triethylamine (60 μL, 0.43 mmol, 1.2 equiv). The reaction mixture was stirred at room temperature for 2.5 h. Then, the reaction was quenched with a saturated aqueous solution of sodium bicarbonate. The resulting mixture was extracted three times with diethyl ether. The combined organic layers were washed with water and brine, dried over anhydrous sodium sulfate, filtered, and concentrated under reduced pressure. The residue was purified by column chromatography on silica gel eluted with a petroleum ether/diethyl ether mixture 6:4 to afford the desired product **49** with 72% yield. Brown oil; *R_f_* = 0.62 (petroleum ether/acetone/dichloromethane 7:2:1). ^1^H NMR (400 MHz, CDCl_3_) δ_H_ 7.71 (d, *J* = 8.4 Hz, 2H), 7.30 (d, *J* = 7.9 Hz, 2H), 6.55 (d, *J* = 2.6 Hz, 1H), 6.53 (d, *J* = 2.4 Hz, 1H), 5.26 (t, *J* = 7.4 Hz, 1H), 5.14–5.07 (m, 2H), 4.09 (s, 2H), 2.67–2.63 (m, 2H), 2.44 (s, 3H), 2.15-2.04 (m, 6H), 2.05 (s, 3H), 1.99–1.95 (m, 4H), 1.81–1.69 (m, 2H), 1.78 (s, 3H), 1.65–1.50 (m, 2H), 1.58 (s, 6H), 1.25 (s, 3H). ^13^C NMR (100 MHz, CDCl_3_) δ_C_ 150.7, 145.0, 141.5, 135.3, 134.6, 134.5, 132.9, 129.6 (2C), 128.6 (2C), 128.2, 127.5, 124.8, 124.2, 121.9, 121.2, 120.2, 76.2, 61.6, 39.9 (2C), 39.7, 30.9, 26.6, 26.3, 24.1, 22.4, 22.2, 21.8, 21.3, 16.1 (2C), 15.9.

#### (2*Z*,6*E*,10*E*)-13-((*R*)-2,8-Dimethyl-6-(tosyloxy)chroman-2-yl)-2,6,10-trimethyltrideca-2,6,10-trienoic Acid (**50**)

To a solution of **49** (152 mg, 0.27 mmol, 1 equiv) in a mixture of 1:9 dimethyl sulfoxide/dichloromethane (5 mL) was added 2-iodoxybenzoic acid (280 mg, 0.54 mmol, 2 equiv). The reaction mixture was stirred at room temperature for 12 h. Then, the reaction was quenched with a saturated aqueous solution of sodium bicarbonate. The resulting mixture was extracted three times with dichloromethane. The combined organic layers were washed with water and brine, dried over anhydrous sodium sulfate, filtered, and concentrated under reduced pressure. The crude aldehyde was used for the next step without further purification. To a solution of aldehyde (145 mg, 0.26 mmol, 1 equiv) in 1,4-dioxane (5 mL) at 0 °C were added sulfamic acid (40 mg, 0.42 mmol, 1.6 equiv), 2-methyl-2-butene (1.7 mL, 13 mmol, 50 equiv), and a solution of sodium chlorite (33 mg, 0.36 mmol, 1.4 equiv) in water (1.8 mL). The reaction mixture was stirred at 0 °C for 2 h. Then, the reaction was quenched with sodium sulfite and diluted with water. The resulting mixture was extracted three times with diethyl ether. The combined organic layers were washed with water and brine, dried over anhydrous sodium sulfate, filtered, and concentrated under reduced pressure. The residue was purified by column chromatography on silica gel using a 7:3 mixture of petroleum ether/diethyl ether as the eluent to afford the expected product **50** with 75% yield over the two-step oxidation. Brown oil; *R_f_* = 0.45 (petroleum ether/ethyl acetate 75:25). ^1^H NMR (400 MHz, CDCl_3_) δ_H_ 7.72 (d, *J* = 8.4 Hz, 2H), 7.30 (d, *J =* 8.0 Hz, 2H), 6.55 (d, *J* = 2.6 Hz, 1H), 6.52 (d, *J* = 2.8 Hz, 1H), 6.05 (td, *J* = 1.4, 7.9 Hz, 1H), 5.13–5.10 (m, 2H), 2.67–2.58 (m, 4H), 2.45 (s, 3H), 2.12–2.04 (m, 6H), 2.05 (s, 3H), 1.98–1.94 (m, 2H), 1.90 (s, 3H), 1.83–1.69 (m, 2H), 1.64–1.50 (m, 2H), 1.58 (s, 6H), 1.25 (s, 3H). ^13^C NMR (100 MHz, CDCl_3_) δ_C_ 171.1, 150.8, 145.1, 141.5, 135.4, 134.8 (2C), 132.9, 129.7 (2C), 128.7 (2C), 127.6, 126.3, 124.5, 124.2, 122.0, 121.2, 120.3, 76.3, 39.9, 39.8, 39.4, 31.0, 26.7, 26.4, 24.2, 22.4, 22.2, 21.9, 16.2, 16.1, 16.0, 13.8. IR (ATR) ν_max_ 2923, 2852, 1686, 1473, 1454, 1372, 1093, 984 cm^–1^. MS (EI) *m*/*z* calcd for C_34_H_44_O_6_S [M^+•^] 580.3, found 580.2.

#### (2*Z*,6*E*,10*E*)-13-((*R*)-6-Hydroxy-2,8-dimethylchroman-2-yl)-2,6,10-trimethyltrideca-2,6,10-trienoic Acid (**13e**)

To a solution of **50** (141 mg, 0.24 mmol, 1 equiv) in methanol (8 mL) was added sodium hydroxide (16 mmol, 640 mg). The mixture was stirred at 70 °C for 3 h. Then, the reaction was quenched with an aqueous solution of hydrochloric acid (1 M). The resulting mixture was extracted three times with dichloromethane. The combined organic layers were washed with water and brine, dried over anhydrous sodium sulfate, filtered, and concentrated under reduced pressure. The residue was purified by column chromatography eluted with a petroleum ether/acetone mixture, 8:2, to afford the desired product **13e** with 95% yield. Pale-yellow oil; *R_f_* = 0.29 (petroleum ether/acetone 8:2). ^1^H NMR (400 MHz, CDCl_3_) δ_H_ 6.47 (d, *J* = 3.5 Hz, 1H), 6.38 (d, *J* = 2.8 Hz, 1H), 6.24 (bs, 1H), 6.06 (td, *J* = 1.4, 7.2 Hz, 1H), 5.14–5.09 (m, 2H), 2.69 (t, *J* = 6.8 Hz, 2H), 2.60 (dd, *J* = 7.3, 13.7 Hz, 2H), 2.19–2.16 (m, 2H), 2.13–2.10 (m, 2H), 2.12 (s, 3H), 2.09–2.04 (m, 6H), 1.98–1.95 (m, 2H), 1.90 (s, 3H), 1.83–1.71 (m, 2H), 1.67–1.49 (m, 2H), 1.58 (s, 3H), 1.57 (s, 3H), 1.26 (s, 3H). ^13^C NMR (100 MHz, CDCl_3_) δ_C_ 172.3, 147.8, 146.4, 146.1, 135.2, 134.2, 127.5, 126.0, 125.1, 124.5, 121.4, 115.8, 112.7, 75.5, 39.7, 39.6, 39.1, 31.5, 28.3, 26.7, 24.2, 22.6, 22.3, 20.7, 16.2, 16.0 (2C). IR (ATR) ν_max_ 3335, 2924, 2852, 1686, 1471, 1378, 1220, 1095, 939 cm^–1^. HRMS (EI) *m*/*z* calcd for C_27_H_38_O_4_ [M^+•^] 426.2770, found 426.2768.

#### (*R*)-5-Chloro-2,8-dimethyl-2-((4*R*,8*R*)-4,8,12-trimethyltridecyl)chroman-6-ol (**51**)

To a solution of **1d** (1 g, 2.5 mmol, 1 equiv) in methanol (15 mL) was added *N*-chlorosuccinimide (400 mg, 1.2 equiv). The reaction was stirred at room temperature for 2 h until completion monitored by TLC (petroleum ether/acetone 8:2). After removal of the solvent under reduced pressure, the crude residue was taken into hexane and filtered on a Celite pad. The filtrate was concentrated under reduced pressure and purified on silica gel flash chromatography using a 95:5 mixture of petroleum ether/acetone as the mobile phase, leading to 5-chloro-δ-tocopherol **51** with 95% yield. Pale-yellow oil. *R_f_* = 0.74 (petroleum ether/acetone 8:2). ^1^H NMR (400 MHz, CDCl_3_) δ_H_ 6.70 (d, *J* = 0.7 Hz, 1H), 5.04 (s, 1 H, OH), 2.71 (q, *J* = 6.7 Hz, 2H), 2.12 (d, *J* = 0.7 Hz, 3H), 1.86–1.72 (m, 2H), 1.54–1.47 (m, 4H), 1.44–1.32 (m, 4H), 1.31–1.17 (m, 11H), 1.15–1.00 (m, 5H), 0.88–0.82 (m, 12H). ^13^C NMR (100 MHz, CDCl_3_) δ_C_ 146.2, 143.9, 126.2, 118.8, 116.5, 115.3, 75.5, 39.6, 39.5, 37.6, 37.5, 37.4, 32.9, 32.8, 31.0, 29.8, 28.1, 24.9, 24.6, 23.9, 22.9, 22.8, 21.4, 21.1, 19.9, 19.8, 16.1. HRMS (ESI) *m*/*z* calcd for C_27_H_44_ClO_2_ [M – H]^−^ 435.3030, found 435.3033.

#### (*R*)-5-Chloro-6-hydroxy-2,8-dimethyl-2-((4*R*,8*R*)-4,8,12-trimethyltridecyl)chromane-7-carbaldehyde (**2**)

To a stirred solution of **51** (500 mg, 1.14 mmol) in dry THF (20 mL) were added magnesium chloride (1.085 g, 10 equiv), paraformaldehyde (1.094 g, 32 equiv), and triethylamine (4.8 mL, 32 equiv) at room temperature. The mixture was stirred under reflux until the completion of the reaction was observed by TLC. The heterogeneous mixture was cooled down to room temperature, and 1 N HCl (10 mL) and Et_2_O (30 mL) were added dropwise. The organic layer was separated, and the aqueous layer was extracted with Et_2_O (2 × 10 mL). The combined organic extracts were washed with brine (20 mL), dried over Na_2_SO_4_, and evaporated to dryness. Purification by column chromatography on silica gel, using a petroleum ether/diethyl ether (PE/Et_2_O) mixture as the mobile phase (100:0 to 95:5), afforded the desired formylated product **2** with 75% yield. *R_f_* = 0.50 (Et_2_O/petroleum ether 1:9). ^1^H NMR (500 MHz, acetone-*d*_6_) δ_H_ 12.02 (s, 1H), 10.39 (s, 1H), 2.82–2.85 (m, 2H), 2.49 (s, 3H), 1.85–1.95 (m, 2H), 1.29 (s, 3H), 1.05–1.66 (m, 21H), 0.85–0.88 (m, 12H). ^13^C NMR (125 MHz, acetone-*d*_6_) δ_C_ 197.2, 153.3, 145.6, 131.4, 128.6, 118.8, 118.5, 76.9, 40.1, 39.9, 38.0 (2C), 38.1 (2C), 33.5, 33.3, 31.1, 28.7, 25.5, 25.1, 22.8, 24.0, 23.0, 22.9, 21.6, 20.0, 20.1, 9.7. HRMS (FAB) *m*/*z* calcd for C_28_H_45_ClO_3_ [M^+•^] 464.3052, found 464.3050.

#### (*R*)-6-(Methoxymethoxy)-2,8-dimethyl-2-((4*R*,8*R*)-4,8,12-trimethyltridecyl)chromane-5-carbaldehyde (**4**)

To a solution of aldehyde **3**(38) (66 mg, 0,16 mmol, 1.0 equiv) in 3 mL of distilled THF at −5 °C under a nitrogen atmosphere was added dropwise *n*-BuLi 1.6 M in hexane (200 μL, 0.30 mmol, 1.95 equiv). The reaction mixture was stirred for 2 h at −5 °C. Then, bromomethyl methyl ether (30 μL, 0.37 mmol, 2.4 equiv) was added, and the resulting mixture was stirred for 20 min. After dilution with EtOAc (15 mL), the organic layer was washed with a saturated aqueous NH_4_Cl solution, water, and brine (15 mL each), dried over sodium sulfate, and filtered. The solvent was evaporated under reduced pressure. The crude product was purified by preparative TLC using an 8:2 mixture of ether/petroleum ether to lead to 58 mg of **4** (80% yield). *R_f_* = 0.30 (Et_2_O/petroleum ether 1:9). ^1^H NMR (500 MHz, acetone-*d*_6_) δ_H_ 10.57 (s, 1H), 7.00 (s, 1H), 5.25 (s, 2H), 3.48 (s, 3H), 3.01–3.11 (m, 2H), 2.19 (s, 3H), 1.72–1.82 (m, 2H), 1.26 (s, 3H), 1.09–1.59 (m, 21H), 0.85–0.88 (m, 12H). ^13^C NMR (125 MHz, acetone-*d*_6_) δ_C_ 191.8, 155.6, 147.8, 135.4, 123.0, 122.6, 117.0, 96.0, 76.0, 56.0, 40.2, 40.1, 38.1 (2C), 38.0 (2C), 33.5, 33.3, 31.6, 28.7, 25.5, 25.1, 24.1, 23.0, 22.9, 22.0, 21.6, 20.1, 20.0, 17.4. HRMS (FAB) *m*/*z* calcd for C_30_H_50_O_4_ [M^+•^] 474.3704, found 474.3706.

#### (2*E*,6*E*,10*E*)-Methyl-13-((*R*)-6-methoxy-2,8-dimethylchroman-2-yl)-2,6,10-trimethyltrideca-2,6,10-trienoate (**53**)

To a solution of sodium hydride (140 mg, 3.5 mmol, 5 equiv) in dimethyformamide (5 mL) were added at 0 °C a solution of **13d** (300 mg, 0.70 mmol, 1 equiv) in dimethyformamide (5 mL) and iodomethane (174 μL, 2.8 mmol, 4 equiv). After 15 min at 0 °C, the reaction was stirred at room temperature for 1 h. Then, the reaction was quenched with water. The resulting mixture was extracted three times with diethyl ether. The combined organic layers were washed with water and brine, dried over anhydrous sodium sulfate, filtered, and concentrated under reduced pressure. The residue was purified by column chromatography on silica gel eluted with a petroleum ether/acetone (98:2) mixture to afford **53** with 67% yield. Yellow oil; *R_f_* = 0.76 (petroleum ether/acetone 9:1). ^1^H NMR (400 MHz, CDCl_3_) δ_H_ 6.74 (td, *J* = 1.4, 7.3 Hz, 1H), 6.56 (d, *J* = 2.7 Hz, 1H), 6.44 (d, *J* = 3.0 Hz, 1H), 5.15–5.12 (m, 2H), 3.73 (s, 6H), 2.75–2.71 (m, 2H), 2.26 (dd, *J =* 7.5, 15.0 Hz, 2H), 2.15 (s, 3H), 2.13–2.04 (m, 6H), 1.99–1.95 (m, 2H), 1.83 (s, 3H), 1.81–1.71 (m, 2H), 1.68–1.51 (m, 2H), 1.59 (s, 6H), 1.26 (s, 3H). ^13^C NMR (100 MHz, CDCl_3_) δ_C_ 168.8, 152.2, 146.2, 142.5, 135.1, 134.0, 127.5, 127.4, 125.2, 124.5, 121.0, 114.9, 111.1, 75.4, 55.8, 51.8, 39.8, 39.7, 38.3, 31.5, 27.5, 26.7, 24.2, 22.8, 22.3, 16.4, 16.1, 16.0, 12.6. IR (ATR) ν_max_ 2924, 2849, 1713, 1481, 1435, 1274, 1219, 1192, 1150, 1122, 1094, 1060 cm^–1^. MS (EI) *m*/*z* calcd for C_29_H_42_O_4_ [M^+•^] 454.3, found 454.4.

#### (6*E*,10*E*)-Methyl-13-((*R*)-6-hydroxy-2,8-dimethylchroman-2-yl)-2,6,10-trimethyltrideca-6,10-dienoate (**21**)

To a solution of methyl δ-garcinoate **52**(6) (110 mg, 0.25 mmol, 1 equiv) in dry methanol (10 mL), magnesium turnings were added (122 mg, 5 mmol, 20 equiv). The reaction mixture was stirred at room temperature for 16 h. Then, the reaction was quenched with water. The resulting mixture was extracted 3 times with diethyl ether. The combined organic layers were washed successively with water and brine, dried over anhydrous sodium sulfate, filtered, and concentrated under reduced pressure. The crude residue was purified by column chromatography on silica gel eluted with a petroleum ether/acetone (95:5) mixture to afford the desired reduced product **21** with 66% yield. Brown oil; *R_f_* = 0.45 (petroleum ether/acetone/dichloromethane 7:2:1). ^1^H NMR (400 MHz, CDCl_3_) δ_H_ 6.48 (d, *J* = 3.0 Hz, 1H), 6.39 (d, *J* = 3.0 Hz, 1H), 5.14–5.06 (m, 2H), 3.67 (s, 3H), 2.69 (t, *J* = 6.9 Hz, 2H), 2.44 (dd, *J* = 7.0, 13.0 Hz, 1H), 2.12 (s, 3H), 2.09–2.02 (m, 4H), 1.98–1.92 (m, 4H), 1.81–1.71 (m, 2H), 1.67–1.49 (m, 4H), 1.58 (s, 3H), 1.55 (s, 3H), 1.39–1.33 (m, 2H), 1.26 (s, 3H), 1.14 (d, *J* = 7.0 Hz, 3H). ^13^C NMR (100 MHz, CDCl_3_) δ_C_ 177.7, 148.0, 146.0, 135.2, 134.8, 127.4, 124.6, 124.4, 121.3, 115.8, 112.7, 75.4, 51.7, 39.8, 39.6, 39.5 (2C), 33.4, 31.5, 26.6, 25.6, 24.2, 22.6, 22.3, 17.2, 16.2, 16.0, 15.9. IR (ATR) ν_max_ 2926, 2854, 1735, 1714, 1464, 1218, 1159, 854 cm^–1^. HRMS (FAB) *m*/*z* calcd for C_28_H_42_O_4_ [M^+•^] 442.3083, found 442.3078.

#### (6*E*,10*E*)-Methyl-13-((*R*)-6-methoxy-2,8-dimethylchroman-2-yl)-2,6,10-trimethyltrideca-6,10-dienoate (**22**)

**22** was obtained from **53** with 72% yield using the same method as described above for **21**. Brown oil; *R_f_* = 0.68 (petroleum ether/acetone/dichloromethane 7:2:1). ^1^H NMR (400 MHz, CDCl_3_) δ_H_ 6.56 (d, *J* = 2.9 Hz, 1H), 6.44 (d, *J* = 2.9 Hz, 1H), 5.13 (td, *J* = 1.1 Hz, 7.1 Hz, 1H), 5.08 (td, *J* = 1.2 Hz, 6.9 Hz, 1H), 3.73 (s, 3H), 3.66 (s, 3H), 2.75–2.71 (m, 2H), 2.48–2.39 (m, 1H), 2.15 (s, 3H), 2.13–2.03 (m, 6H), 1.98–1.93 (m, 4H), 1.85–1.71 (m, 2H), 1.68–1.51 (m, 2H), 1.59 (s, 3H), 1.55 (s, 3H), 1.41–1.34 (m, 2H), 1.26 (s, 3H), 1.14 (d, *J* = 7.0 Hz, 3H). ^13^C NMR (100 MHz, CDCl_3_) δ_C_ 177.5, 152.2, 146.2, 135.2, 134.8, 127.4, 124.6, 124.4, 121.0, 114.8, 111.1, 75.4, 55.8, 51.6, 39.8, 39.5 (2C), 33.4, 31.5, 29.8, 26.7, 25.6, 24.2, 22.8, 22.3, 17.2, 16.3, 16.0, 15.9. IR (ATR) ν_max_ 2923, 2853, 1738, 1481, 1463, 1377, 1219, 1196, 1151, 1061, 855 cm^–1^. HRMS (ESI) *m*/*z* calcd for C_29_H_45_O_4_ [M + H]^+^ 457.3318, found 457.3320

#### (6*E*,10*E*)-13-((*R*)-6-Hydroxy-2,8-dimethylchroman-2-yl)-2,6,10-trimethyltrideca-6,10-dienoic Acid (**19b**)

To a solution of **21** (60 mg, 1 equiv) in methanol, crushed sodium hydroxide (2 M) was added. The mixture was stirred at 70 °C for 4 h, and then, the reaction was quenched with an aqueous solution of hydrochloric acid (1 M). The resulting mixture was extracted three times with diethyl ether, and the combined organic layers were washed with water and brine, dried over anhydrous sodium sulfate, filtered, and concentrated under reduced pressure. The residue was purified by column chromatography on silica gel eluted with a petroleum ether/acetone (8:2) mixture to afford the corresponding carboxylic acid **19b** with 61% yield from **13d** (3 steps). Brown oil; *R_f_* = 0.26 (petroleum ether/acetone/dichloromethane 7:2:1). ^1^H NMR (400 MHz, CDCl_3_) δ_H_ 6.48 (d, *J* = 3.0 Hz, 1H), 6.38 (d, *J* = 2.9 Hz, 1H), 5.14–5.10 (m, 2H), 2.69 (t, *J* = 6.8 Hz, 2H), 2.48–2.43 (m, 1H), 2.12 (s, 3H), 2.10–2.03 (m, 4H), 1.99–1.94 (m, 4H), 1.83–1.71 (m, 2H), 1.67–1.50 (m, 4H), 1.59 (s, 3H), 1.56 (s, 3H), 1.45–1.36 (m, 2H), 1.26 (s, 3H), 1.17 (d, *J* = 7.0 Hz, 3H). ^13^C NMR (100 MHz, CDCl_3_) δ_C_ 182.9, 147.8, 146.1, 135.2, 134.7, 127.5, 124.6, 124.5, 121.4, 115.8, 112.7, 75.5, 39.8, 39.6, 39.5, 39.3, 33.2, 31.4, 26.6, 25.4, 24.2, 22.6, 22.3, 17.0, 16.2, 16.0, 15.9. IR (ATR) ν_max_ 2927, 1703, 1609, 1465, 1378, 1219, 1097, 853, 737 cm^–1^. HRMS (FAB) *m*/*z* calcd for C_27_H_40_O_4_ [M^+•^] 428.2927, found 428.2930.

#### (6*E*,10*E*)-13-((*R*)-6-Methoxy-2,8-dimethylchroman-2-yl)-2,6,10-trimethyltrideca-6,10-dienoic Acid (**20**)

**20** was obtained from **22** with 42% yield from **13d** (3 steps) using the same method as described above for **19b**. Brown oil; *R_f_* = 0.55 (petroleum ether/acetone/dichloromethane 7:2:1). ^1^H NMR (400 MHz, CDCl_3_) δ_H_ 6.57 (d, *J* = 2.8 Hz, 1H), 6.44 (d, *J* = 3.0 H*z*, 1H), 5.15–5.08 (m, 2H), 3.73 (s, 3H), 2.75–2.71 (m, 2H), 2.48–2.43 (m, 1H), 2.16 (s, 3H), 2.13–2.04 (m, 6H), 1.99–1.95 (m, 4H), 1.85–1.72 (m, 2H), 1.68–1.51 (m, 2H), 1.59 (s, 3H), 1.57 (s, 3H), 1.46–1.36 (m, 2H), 1.27 (s, 3H), 1.17 (d, *J* = 7.0 H***z***, 3H). ^13^C NMR (100 MHz, CDCl_3_) δ_C_ 182.9, 152.2, 146.2, 135.2, 134.7, 127.4, 124.7, 124.4, 121.0, 114.9, 111.1, 75.5, 55.8, 39.8 (2C), 39.5, 39.3, 33.2, 31.5, 26.7, 25.4, 24.1, 22.8, 22.3, 17.0, 16.4, 16.0, 15.9. IR (ATR) ν_max_ 2922, 2852, 1705, 1481, 1465, 1377, 1280, 1220, 1150, 1061, 950 cm^–1^. HRMS (ESI) *m*/*z* calcd for C_28_H_41_O_4_ [M – H]^−^ 441.3005, found 441.3008.

#### (6*E*,10*E*)-13-((*R*)-6-Hydroxy-2,5,8-trimethylchroman-2-yl)-2,6,10-trimethyltrideca-6,10-dienoic Acid (**19a**)

To a solution of **19b** (1 equiv) in ethanol (0.08 M) were added *N,N,N′*,*N*′-tetramethyldiaminomethane (3 equiv) and paraformaldehyde (3 equiv). The reaction mixture was stirred at 120 °C under microwave irradiation for 1 h. Then, the reaction was quenched with water. The resulting mixture was extracted three times with dichloromethane. The combined organic layers were washed with water and brine, dried over anhydrous sodium sulfate, filtered, and concentrated under reduced pressure. The brown oily crude product was used without further purification. To a solution of 5-dimethylaminomethylchromanol derivative (1 equiv) in ethanol (0.07 M) was added sodium cyanoborohydride (5 equiv). The reaction mixture was stirred at 120 °C under microwave irradiation for 45 min. Then, the reaction was quenched with an aqueous solution of hydrochloric acid (1 M). The resulting mixture was extracted three times with diethyl ether. The combined organic layers were washed with water and brine, dried over anhydrous sodium sulfate, filtered, and concentrated under reduced pressure. The residue was purified by column chromatography on silica gel eluted with a petroleum ether/acetone (9:1) mixture to afford **19a** with 62% yield. ^1^H NMR (400 MHz, CDCl_3_) δ_H_ 6.48 (s, 1H), 5.10 (m, 2H), 2.60 (q, *J* = 6.8 Hz, 2H), 2.46 (m, 1H), 2.11 (s, 3H), 2.08 (s, 3H), 2.08–2.01 (m, 3H), 2.01–1.89 (m, 4H), 1.91–1.69 (m, 2H), 1.69–1.60 (m, 2H), 1.59 (s, 3H), 1.56 (s, 3H), 1.50–1.45 (m, 1H), 1.43–1.38 (m, 3H), 1.25 (s, 3H), 1.18 (s, 3H), 1.16 (s, 3H). ^13^C NMR (100 MHz, CDCl_3_) δ_C_ 182.5, 146.0, 145.8, 135.1, 134.7, 124.7, 124.5, 124.2, 120.5, 119.3, 115.4, 74.4, 39.8, 39.5, 39.4, 39.3, 33.2, 31.6, 26.7, 25.4, 23.9 (2C), 22.3, 20.9, 17.0, 16.0, 15.9, 11.1. HRMS (ESI) *m*/*z* calcd for C_28_H_41_O_4_ [M – H]^−^ 441.3005, found 441.3004.

#### (4*E*,8*E*)-11-((*R*)-2,8-Dimethyl-6-(tosyloxy)chroman-2-yl)-4,8-dimethylundeca-4,8-dienoic Acid (**55**)

To a solution of **54**(39) (198 mg, 0.38 mmol, 1 equiv) in 1,4-dioxane (7.5 mL) at 0 °C were added sulfamic acid (59 mg, 0.60 mmol, 1.6 equiv), 2-methyl-2-butene (2.4 mL, 18.9 mmol, 50 equiv), and a solution of sodium chlorite (60 mg, 0.53 mmol, 1.4 equiv) in water (2.6 mL). The reaction mixture was stirred at 0 °C for 2 h. Then, the reaction was quenched with sodium sulfite and diluted with water. The resulting mixture was extracted three times with diethyl ether. The combined organic layers were washed with water and brine, dried over anhydrous sodium sulfate, filtered, and concentrated under reduced pressure. The crude product **55** can be used in the next step without further purification. *R_f_* = 0.31 (petroleum ether/acetone 8:2). ^1^H NMR (400 MHz, CDCl_3_) δ_H_ 7.72 (d, *J* = 8.0 Hz, 2H), 7.31 (d, *J* = 7.9 Hz, 2H), 6.55 (s, 1H), 6.52 (s, 1H), 5.14–5.09 (m, 2H), 2.67–2.63 (m, 2H), 2.45 (s, 3H), 2.45–2.42 (m, 2H), 2.31–2.27 (m, 2H), 2.05 (s, 3H), 2.10–2.03 (m, 4H), 1.98–1.94 (m, 2H), 1.81–1.69 (m, 2H), 1.68–1.50 (m, 2H), 1.60 (s, 3H), 1.57 (s, 3H), 1.25 (s, 3H). ^13^C NMR (100 MHz, CDCl_3_) δ_C_ 179.3, 150.8, 145.1, 131.5, 135.2, 133.1, 132.9, 129.7 (2C), 128.7 (2C), 127.6, 125.3, 124.3, 122.0, 121.2, 120.3, 76.3, 39.9, 39.6, 34.4, 33.0, 31.0, 26.6, 24.2, 22.4, 22.2, 21.9, 16.2, 16.1, 16.0. IR (ATR) ν_max_ 2923, 1707, 1598, 1473, 1449, 1372, 1225, 1094, 1049, 814, 696, 552 cm^–1^. HRMS (FAB) *m*/*z* calcd for C_31_H_40_O_6_S [M^+•^] 540.2546, found 540.2544.

#### (4*E*,8*E*)-11-((*R*)-6-Hydroxy-2,8-dimethylchroman-2-yl)-4,8-dimethylundeca-4,8-dienoic Acid (**15b**)

To a solution of **55** (175 mg, 0.32 mmol, 1 equiv) in methanol (6 mL) was added sodium hydroxide (467 mg). The mixture was stirred at 70 °C for 1.5 h. Then, the reaction was quenched with an aqueous solution of hydrochloric acid (1 M). The resulting mixture was extracted three times with ethyl acetate. The combined organic layers were washed with water and brine, dried over anhydrous sodium sulfate, filtered, and concentrated under reduced pressure. The residue was purified by column chromatography on silica gel eluted with a petroleum ether/acetone (8:2) mixture to afford the desired product **15b** with 77% yield over two steps from **54**. Yellow oil; *R_f_* = 0.25 (petroleum ether/acetone 8:2). ^1^H NMR (400 MHz, CDCl_3_) δ_H_ 6.48 (d, *J* = 2.6 Hz, 1H), 6.38 (d, *J* = 2.6 Hz, 1H), 5.16–5.10 (m, 2H), 2.69 (t, *J* = 7.0 Hz, 2H), 2.44 (t, *J* = 7.8 Hz, 2H), 2.29 (t, *J* = 7.8 Hz, 2H), 2.13 (s, 3H), 2.13–2.04 (m, 4H), 1.98–1.95 (m, 2H), 1.83–1.71 (m, 2H), 1.70–1.50 (m, 2H), 1.60 (s, 3H), 1.58 (s, 3H), 1.26 (s, 3H). ^13^C NMR (100 MHz, CDCl_3_) δ_C_ 179.6, 147.9, 146.0, 134.9, 133.1, 127.5, 125.4, 124.6, 121.4, 115.8, 112.8, 75.4, 39.6 (2C), 34.4, 33.1, 31.5, 26.6, 24.2, 22.6, 22.3, 16.2, 16.1, 16.0. IR (ATR) ν_max_ 2921, 1707, 1469, 1218, 1146, 1095, 933, 853 cm^–1^. HRMS (EI) *m*/*z* calcd for C_24_H_34_O_4_ [M^+•^] 386.2457, found 386.2455.

#### (4*E*,8*E*)-11-((*R*)-6-Hydroxy-2,5,7,8-tetramethylchroman-2-yl)-4,8-dimethylundeca-4,8-dienoic Acid (**15a**)

To a solution of **15b** (1 equiv) in 1,4-dioxane (0.08 M) were added *N*,*N*,*N*′,*N*′-tetramethyldiaminomethane (20 equiv) and paraformaldehyde (20 equiv). The reaction mixture was stirred at 140 °C under microwave irradiation. Completion of the reaction was monitored by TLC. Then, the reaction was quenched with water. The resulting mixture was extracted three times with dichloromethane. The combined organic layers were washed with water and brine, dried over anhydrous sodium sulfate, filtered, and concentrated under reduced pressure. The brown oily crude product was used without further purification. To a solution of α-5,7-bis(dimethylaminomethyl)-tocodienol derivative (1 equiv) in ethanol (0.06 M) was added sodium cyanoborohydride (20 equiv). The reaction mixture was stirred at 150 °C under microwave irradiation for 0.75–1.5 h. Then, the reaction was quenched with an aqueous solution of hydrochloric acid (1 M). The resulting mixture was extracted three times with diethyl ether. The combined organic layers were washed with water and brine, dried over anhydrous sodium sulfate, filtered, and concentrated under reduced pressure. The residue was purified by column chromatography on silica gel eluted with a petroleum ether/acetone mixture to afford **15a** with 75% yield. Yellow oil; *R_f_* = 0.22 (petroleum ether/acetone 8:2). ^1^H NMR (400 MHz, CDCl_3_) δ_H_ 5.16–5.11 (m, 2H), 2.62 (t, *J* = 6.8 Hz, 2H), 2.46–2.42 (m, 2H), 2.31–2.27 (m, 2H), 2.16 (s, 3H), 2.12 (s, 3H), 2.11 (s, 3H), 2.09–2.04 (m, 4H), 1.98–1.94 (m, 2H), 1.87–1.74 (m, 2H), 1.68–1.50 (m, 2H), 1.60 (s, 3H), 1.59 (s, 3H), 1.25 (s, 3H). ^13^C NMR (100 MHz, CDCl_3_) δ_C_ 179.5, 145.6, 144.7, 134.9, 133.1, 125.4, 124.7, 122.8, 121.2, 118.7, 117.5, 74.4, 39.6 (2C), 34.4, 33.0, 31.7, 26.6, 23.8, 22.3, 20.9, 16.1, 16.0, 12.4, 11.9, 11.4. IR (ATR) ν_max_ 3459, 2922, 2852, 1708, 1453, 1378, 1249, 1087 cm^–1^. HRMS (ESI) *m*/*z* calcd for C_26_H_37_O_4_ [M – H]^−^ 413.2692, found 413.2689.

#### (*R*)-Methyl-6-hydroxy-2-((3*E*,7*E*,11*E*)-13-methoxy-4,8,12-trimethyl-13-oxotrideca-3,7,11-trien-1-yl)-2,8-dimethylchroman-5-carboxylate (**25**)

To a solution of diacid **56**(47) (1 equiv) in dimethylformamide (0.13 M) were added sodium bicarbonate (4 equiv) and iodomethane (4 equiv). The reaction mixture was stirred at 120 °C under microwave irradiation for 2 h. Then, the reaction was quenched with water. The resulting mixture was extracted three times with diethyl ether. The combined organic layers were washed with water and brine, dried over anhydrous sodium sulfate, filtered, and concentrated under reduced pressure. The residue was purified by column chromatography on silica gel eluted with a petroleum ether/acetone mixture 95:5 to afford **25** with 85% yield. Colorless oil; *R_f_* = 0.3 (petroleum ether/ethyl acetate 9:1). ^1^H NMR (400 MHz, CDCl_3_) δ_H_ 10.83 (s, 1H), 6.73 (dd, *J* = 7.3, 5.9 Hz, 1H), 6.68 (s, 1H), 5.17–5.08 (m, 2H), 3.93 (s, 3H), 3.72 (s, 3H), 2.98 (t, *J* = 6.9 Hz, 2H), 2.26 (s, 2H), 2.19–2.15 (m, 3H), 2.14–2.03 (m, 6H), 1.97 (d, *J* = 7.9 Hz, 2H), 1.82 (d, *J* = 1.2 Hz, 3H), 1.74 (d, *J* = 6.8 Hz, 2H), 1.64–1.53 (m, 8H), 1.25 (s, 3H). ^13^C NMR (100 MHz, CDCl_3_) δ_C_ 172.2, 168.8, 156.3, 145.3, 142.5, 136.3, 135.2, 134.0, 127.5, 125.2, 124.4, 120.8, 117.6, 109.0, 74.5, 52.0, 51.8, 39.7, 39.5, 38.3, 31.6, 27.5, 26.7, 23.7, 23.0, 22.2, 17.2, 16.1, 16.0, 12.5. HRMS (ESI) *m*/*z* calcd for C_30_H_42_O_6_Na [M + Na]^+^ 521.2879, found 521.2878.

#### (2R)-2,8,-Dimethyl-2-[(3*E*,7*E*)-4,8,12-trimethyldeca-3,7,11-trien-1-yl]-3,4-dihydro-2H-1-benzopyran-6-yl-4-methylbenzene-1-sulfonate (**57**)

To a solution of **6d** (600 mg; 1.51 mmol; 1 equiv) in dichloromethane (33 mL) were added 4-toluenesulfonyl chloride (316.5 mg; 1.66 mmol; 1.1 equiv) and trimethylamine (252.2 μL; 1.81 mmol; 1.2 equiv). The reaction mixture was stirred at room temperature for 4 h. Then, the reaction was quenched with a saturated aqueous solution of sodium bicarbonate after being extracted 3 times with diethyl ether. The combined organic layers were washed twice with water and once with brine and afterward dried over anhydrous sodium sulfate, filtrated through cotton, and evaporated under reduced pressure. The residue was purified by automated flash chromatography on silica gel eluted with a mixture of petroleum ether/acetone (9:1). The desired product was obtained in 60% yield. Light-brown oil, *R_f_* = 0.43 (petroleum ether/acetone 8:2) ^1^H NMR (400 MHz, CDCl_3_) δ_H_ 7.72 (d, *J* = 8.4 Hz, 2H), 7.31 (d, *J* = 7.9 Hz, 2H), 6.56 (d, *J* = 2.6 Hz, 1H), 6.52 (d, *J* = 2.4 Hz, 1H), 5.14–5.07 (m, 3H), 2.67–2.63 (m, 2H), 2.45 (s, 3H), 2.13–2.02 (m, 6H), 2.05 (s, 3H), 1.99–1.94 (m, 4H), 1.82–1.70 (m, 2H), 1.67 (s, 3H), 1.64–1.52 (m, 2H), 1.59 (s, 3H), 1.58 (s, 6H), 1.25 (s, 3H). ^13^C NMR (100 MHz, CDCl_3_) δ_C_ 150.8, 145.1, 141.5, 135.5, 135.2, 132.9, 131.4, 129.7 (2C), 128.7 (2C), 127.6, 124.5, 124.2, 124.1, 122.0, 121.2, 120.3, 76.3, 39.9, 39.8 (2C), 31.0, 26.9, 26.7, 25.9, 24.2, 22.4, 22.2, 21.8, 17.8, 16.2, 16.1, 16.0. IR (ATR) ν_max_ 2919, 2853, 1473, 1449, 1373, 1189, 1093, 984, 813, 581 cm^–1^. HRMS (FAB) *m*/*z* calcd for C_34_H_46_O_4_S [M^+**]**^ 550.3117, found 550.3116.

### Cross-Metathesis Procedure for the Preparation of **58**–**60**

To a solution of tosylated δ-tocotrienol **57** (30 mg, 0.06 mmol, 1 equiv) in degassed deuterated chloroform (0.7 mL) were added methyl acrylate (10.8 μL, 0.12 mmol, 2.4 equiv) and Grubbs-II catalyst (0.1 equiv). The reaction mixture was refluxed for 3 days. Then, the solvent was removed under reduced pressure. The residue was purified by preparative TLC on silica gel eluted with a cyclohexane/ethyl acetate mixture (7:2).

#### (2*E*,6*E*,10*E*)-Methyl-13-((*R*)-2,8-dimethyl-6-(tosyloxy)chroman-2-yl)-6,10-dimethyltrideca-2,6,10-trienoate (**58**)

**58** was obtained from **57** and methyl acrylate with 10% yield. Yellow oil; *R_f_* = 0.58 (cyclohexane/ethyl acetate 7:2). ^1^H NMR (400 MHz, CDCl_3_) δ_H_ 7.72 (d, *J* = 7.8 Hz, 2H), 7.31 (d, *J* = 8.0 Hz, 2H), 6.98–6.91 (m, 1H), 6.56 (s, 1H), 6.52 (s, 1H), 5.81 (d, *J* = 15.5 Hz, 1H), 5.14–5.10 (m, 2H), 3.72 (s, 3H), 2.69–2.61 (m, 2H), 2.45 (s, 3H), 2.31–2.26 (m, 2H), 2.12–2.08 (m, 6H), 2.05 (s, 3H), 1.98–1.94 (m, 2H), 1.82–1.70 (m, 2H), 1.67–1.50 (m, 2H), 1.58 (s, 6H), 1.25 (s, 3H). ^13^C NMR (100 MHz, CDCl_3_) δ_C_ 167.3, 150.8, 149.5, 145.1, 141.5, 136.3, 133.6, 133.0, 129.7 (2C), 128.7 (2C), 127.6, 125.3, 124.2, 122.0, 121.2, 121.0, 120.3, 76.3, 51.6, 39.9, 39.7, 38.0, 31.0, 30.9, 26.6, 24.2, 22.4, 22.2, 21.9, 16.2, 16.1, 16.0. IR (ATR) ν_max_ 2925, 2852, 1722, 1473, 1450, 1371, 1189, 1174, 1093, 984 cm^–1^. MS (EI) *m*/*z* calcd for C_34_H_44_O_6_S [M^+•^] 580.3, found 580.3.

#### (2*E*,6*E*)-Methyl-9-((*R*)-2,8-dimethyl-6-(tosyloxy)chroman-2-yl)-6-methylnona-2,6-dienoate (**59**)

**59** was obtained from **57** and methyl acrylate with 30% yield. Yellow oil; *R_f_* = 0.53 (cyclohexane/ethyl acetate 7:2). ^1^H NMR (400 MHz, CDCl_3_) δ_H_ 7.73 (d, *J* = 8.0 Hz, 2H), 7.31 (d, *J* = 7.9 Hz, 2H), 6.98–6.90 (m, 1H), 6.56 (s, 1H), 6.53 (s, 1H), 5.81 (d, *J* = 15.7 Hz, 1H), 5.17–5.13 (m, 1H), 3.71 (s, 3H), 2.68–2.61 (m, 2H), 2.45 (s, 3H), 2.32–2.26 (m, 2H), 2.12–2.09 (m, 2H), 2.05 (s, 3H), 1.81–1.67 (m, 4H), 1.62–1.49 (m, 2H), 1.58 (s, 3H), 1.25 (s, 3H). ^13^C NMR (100 MHz, CDCl_3_) δ_C_ 167.3, 150.8, 149.4, 145.1, 141.6, 133.9, 133.0, 129.7 (2C), 128.7 (2C), 127.6, 125.3, 122.0, 121.2, 121.1, 120.3, 76.2, 51.6, 39.9, 38.0, 31.0, 30.8, 24.1, 22.4, 22.2, 21.8, 16.2, 15.9. IR (ATR) ν_max_ 2925, 2852, 1722, 1473, 1450, 1371, 1189, 1176, 1093, 984 cm^–1^. MS (EI) *m*/*z* calcd for C_29_H_36_O_6_S [M^+•^] 512.2, found 512.3.

#### (*R*,*E*)-Methyl-5-(2,8-dimethyl-6-(tosyloxy)chroman-2-yl)pent-2-enoate (**60**)

**60** was obtained from **57** and methyl acrylate with 49% yield. Yellow oil; *R_f_* = 0.44 (cyclohexane/ethyl acetate 7:2). ^1^H NMR (400 MHz, CDCl_3_) δ_H_ 7.73 (d, *J* = 8.1 Hz, 2H), 7.31 (d, *J* = 8.0 Hz, 2H), 7.03–6.96 (m, 1H), 6.57 (s, 1H), 6.53 (s, 1H), 5.83 (d, *J* = 15.6 Hz, 1H), 3.72 (s, 3H), 2.73–2.61 (m, 2H), 2.45 (s, 3H), 2.39–2.33 (m, 2H), 2.04 (s, 3H), 1.82–1.68 (m, 4H), 1.25 (s, 3H). ^13^C NMR (100 MHz, CDCl_3_) δ_C_ 167.2, 150.4, 149.4, 145.1, 141.7, 132.9, 129.7 (2C), 128.7 (2C), 127.6, 122.2, 121.1, 121.0, 120.4, 75.8, 51.6, 38.3, 31.1, 26.6, 23.9, 22.3, 21.9, 16.2. IR (ATR) ν_max_ 2926, 2851, 1722, 1473, 1371, 1189, 1177, 1094, 984 cm^–1^. MS (EI) *m*/*z* calcd for C_24_H_28_O_6_S [M^+•^] 444.2, found 444.2.

#### (*R*,*E*)-Methyl-5-(2,8-dimethyl-6-(tosyloxy)chroman-2-yl)-2-methylpent-2-enoate (**61**)

To a solution of **57** (30 mg, 0.05 mmol, 1 equiv) in degassed deuterated chloroform (0.7 mL) were added methyl methacrylate (6.4 μL, 0.06 mmol, 1.2 equiv) and Hoveyda–Grubbs-II catalyst (3.55 mg, 0.005 mmol, 0.1 equiv). The reaction mixture was stirred under microwave irradiation at 120 °C for 2.5 h. Then, the solvent was removed under reduced pressure. The residue was purified by preparative TLC on silica gel eluted with a cyclohexane/ethyl acetate mixture 7:2 to afford **61** as an oil with 21% yield. Yellow oil; *R_f_* = 0.41 (cyclohexane/ethyl acetate 7:2). ^1^H NMR (400 MHz, CDCl_3_) δ_H_ 7.73 (d, *J* = 8.0 Hz, 2H), 7.31 (d, *J* = 7.8 Hz, 2H), 6.77 (t, *J* = 7.3 Hz, 1H), 6.57 (s, 1H), 6.53 (s, 1H), 3.72 (s, 3H), 2.73–2.62 (m, 2H), 2.45 (s, 3H), 2.34–2.28 (m, 2H), 2.05 (s, 3H), 1.82 (s, 3H), 1.79–1.69 (m, 2H), 1.67–1.56 (m, 2H), 1.26 (s, 3H). ^13^C NMR (100 MHz, CDCl_3_) δ_C_ 168.7, 150.5, 145.1, 142.2, 141.7, 132.9, 129.7 (2C), 128.7 (2C), 127.8, 127.6, 122.1, 121.0, 120.4, 75.9, 51.9, 38.6, 31.1, 24.0, 23.0, 22.3, 21.9, 16.2, 12.4. IR (ATR) ν_max_ 2924, 2852, 1711, 1471, 1369, 1189, 1176, 1091, 982, 813 cm^–1^. MS (EI) *m*/*z* calcd for C_25_H_30_O_6_S [M^+•^] 458.2, found 458.1.

### Cross-Metathesis Procedure for the Preparation of **62** and **63**

To a solution of **57** (30 mg, 0.05 mmol, 1 equiv) in degassed deuterated chloroform (0.7 mL) were added 2-methylene-1,3-propanediacetate (10 mg, 0.05 mmol, 1.2 equiv) and Grubbs-II catalyst (4.2 mg, 0.005 mmol, 0.1 equiv). The reaction mixture was stirred under microwave irradiation at 120 °C for 2 h. Then, the solvent was removed under reduced pressure. The residue was purified by preparative TLC on silica gel eluted with a cyclohexane/ethyl acetate mixture 7:2 to afford the desired products as oil.

#### (*R*,*E*)-2-(7-(2,8-Dimethyl-6-(tosyloxy)chroman-2-yl)-4-methylhept-4-en-1-ylidene)propane-1,3-diyl Diacetate (**62**)

**62** was obtained from **57** and 2-methylene-1,3-propanediacetate with 27% yield. Yellow oil; *R_f_* = 0.37 (cyclohexane/ethyl acetate 7:2). ^1^H NMR (400 MHz, CDCl_3_) δ_H_ 7.72 (d, *J* = 8.3 Hz, 2H), 7.31 (d, *J* = 8.0 Hz, 2H), 6.56 (d, *J* = 2.8 Hz, 1H), 6.52 (d, *J* = 2.8 Hz, 1H), 5.74 (t, *J* = 7.3 Hz, 1H), 5.13 (td, *J* = 1.1, 7.1 Hz, 1H), 4.64 (s, 2H), 4.55 (s, 2H), 2.68–2.64 (m, 2H), 2.45 (s, 3H), 2.24 (dd, *J* = 7.4 Hz, 15.0 Hz, 2H), 2.13–2.01 (m, 4H), 2.05 (s, 9H), 1.80–1.71 (m, 2H), 1.67–1.50 (m, 2H), 1.58 (s, 3H), 1.25 (s, 3H). ^13^C NMR (100 MHz, CDCl_3_) δ_C_ 171.1, 170.9, 150.7, 145.1, 141.5, 136.5, 135.4, 134.3, 132.9, 129.7 (2C), 129.1, 128.7 (2C), 127.5, 125.1, 122.0, 121.2, 120.3, 76.2, 66.8, 59.9, 39.9, 39.1, 31.0, 29.8, 26.3, 24.1, 22.4, 22.2, 21.8, 21.1, 16.2, 15.9. IR (ATR) ν_max_ 2925, 2853, 1711, 1361, 1221, 1178, 735 cm^–1^. HRMS (FAB) *m*/*z* calcd for C_33_H_42_O_8_S [M]^+^ 598.2600, found 598.2603.

#### (*R*)-2-(3-(2,8-Dimethyl-6-(tosyloxy)chroman-2-yl)propylidene)propane-1,3-diyl Diacetate (**63**)

**63** was obtained from **57** and 2-methylene-1,3-propanediacetate with 38% yield. Yellow oil; *R_f_* = 0.22 (cyclohexane/ethyl acetate 7:2). ^1^H NMR (400 MHz, CDCl_3_) δ_H_ 7.72 (d, *J* = 8.3 Hz, 2H), 7.31 (d, *J* = 8.4 Hz, 2H), 6.56 (d, *J* = 2.8 Hz, 1H), 6.52 (d, *J* = 2.8 Hz, 1H), 5.77 (t, *J* = 7.4 Hz, 1H), 4.68–4.60 (m, 2H), 4.55 (s, 2H), 2.69–2.64 (m, 2H), 2.45 (s, 3H), 2.33–2.27 (m, 2H), 2.05 (s, 3H), 2.03 (s, 6H), 1.81–1.57 (m, 4H), 1.25 (s, 3H). ^13^C NMR (100 MHz, CDCl_3_) δ_C_ 171.1, 170.9, 150.5, 145.1, 141.7, 136.3, 132.9, 129.7 (2C), 129.4, 128.7 (2C), 127.6, 122.1, 121.0, 120.4, 75.9, 66.7, 59.8, 39.5, 31.0, 24.0, 22.3, 22.1, 21.8, 21.1, 21.0, 16.2. IR (ATR) ν_max_ 2917, 2849, 1734, 1471, 1370, 1224, 1178, 734 cm^–1^. HRMS (FAB) *m*/*z* calcd for C_28_H_34_O_8_S [M^+•^] 530.1974, found 530.1977.

#### (*R*,*E*)-2-(7-(6-Hydroxy-2,8-dimethylchroman-2-yl)-4-methylhept-4-en-1-ylidene)propane-1,3-diol (**28**)

To a solution of **62** (1 equiv) in methanol (0.03 M), potassium hydroxide (400 mg) was added. The mixture was stirred at 70 °C until completion of the reaction, monitored by TLC. Then, the reaction was quenched with an aqueous solution of hydrochloric acid (1 M). The resulting mixture was extracted three times with dichloromethane. The combined organic layers were washed with water and brine, dried over anhydrous sodium sulfate, filtered, and concentrated under reduced pressure. The residue was purified by column chromatography on silica gel eluted with a petroleum ether/acetone (9:1 to 8:2) mixture to afford **28** with 53% yield. Pale-yellow oil; *R_f_* = 0.26 (petroleum ether/acetone 7:3). ^1^H NMR (400 MHz, CDCl_3_) δ_H_ 6.48 (d, *J* = 2.4 Hz, 1H), 6.39 (d, *J* = 2.6 Hz, 1H), 5.52 (t, *J* = 7.3 Hz, 1H), 5.13 (td, *J* = 1.1, 7.1 Hz, 1H), 4.29 (s, 2H), 4.20 (s, 2H), 2.69 (t, *J* = 6.7 Hz, 2H), 2.52 (bs, 3H), 2.20–2.16 (m,2H), 2.14–2.08 (m, 2H), 2.12 (s, 3H), 2.03–2.00 (m, 2H), 1.83–1.70 (m, 2H), 1.66–1.48 (m, 2H), 1.58 (s, 3H), 1.26 (s, 3H). ^13^C NMR (100 MHz, CDCl_3_) δ_C_ 147.9, 146.0, 137.1, 134.3, 131.0, 127.5, 125.3, 121.4, 115.8, 112.7, 75.4, 67.9, 60.3, 39.5, 39.4, 31.5, 26.0, 24.2, 22.6, 22.2, 16.2, 16.0. IR (ATR) ν_max_ 3335, 2926, 1608, 1472, 1220, 1147, 994, 854 cm^–1^. HRMS (EI) *m*/*z* calcd for C_22_H_32_O_4_ [M^+•^] 360.2301, found 360.2299.

#### (*R*)-2-(3-(6-Hydroxy-2,8-dimethylchroman-2-yl)propylidene)propane-1,3-diol (**29**)

**29** was obtained from **63** with 57% yield using the method described above for the preparation of **28**. Pale-yellow oil; *R_f_* = 0.21 (petroleum ether/acetone 7:3). ^1^H NMR (400 MHz, CDCl_3_) δ_H_ 6.48 (d, *J* = 2.4 Hz, 1H), 6.38 (d, *J* = 2.8 Hz, 1H), 5.58 (t, *J* = 7.5 Hz, 1H), 4.31 (m, 2H), 4.20 (s, 2H), 2.70 (dd, *J* = 6.6 Hz, 12.9 Hz, 2H), 2.24 (dd, *J* = 7.8, 16.5 Hz, 2H), 2.12 (s, 3H), 1.82–1.54 (m, 4H), 1.25 (s, 3H). ^13^C NMR (100 MHz, CDCl_3_) δ_C_ 148.0, 141.4, 137.1, 131.3, 127.5, 121.3, 115.8, 112.8, 75.1, 67.8, 60.2, 39.6, 31.6, 24.1, 22.5, 21.8, 16.2. IR (ATR) ν_max_ 3334, 2921, 1608, 1466, 1219, 1147, 994, 854 cm^–1^. HRMS (FAB) *m*/*z* calcd for C_17_H_24_O_4_ [M^+•^] 292.1675, found 292.1671.

#### (2*E*,6*E*,10*E*)-13-[(2*R*)-6-Hydroxy-2,8-dimethyl-3,4-dihydro-2*H*-1-benzopyran-2-yl]-6,10-dimethyltrideca-2,6,10-trienoic Acid (**14**)

1st step: A 0.02 M solution of **58** (26.3 mg, 1 equiv) in 4 mL of a mixture of THF/MeOH/H_2_O (3:1:1) was treated with LiOH (5.4 mg, 4.3 equiv). The reaction mixture was stirred at 40 °C overnight. Completion of the reaction was monitored by TLC using a mixture of petroleum ether/acetone/dichloromethane (7:2:1) as the mobile phase. The reaction was quenched with 10% hydrochloric acid and extracted three times with ethyl acetate. The combined organic layers were washed twice with water and once with brine, dried over anhydrous sodium sulfate, and filtered. Removal of the solvent under reduced pressure yielded the corresponding carboxylic acid that was used without further purification. 2nd step: Removal of the tosyl group was achieved in a mixture of methanol (0.01 M) and 2 M aqueous potassium hydroxide solution (1:1). The reaction mixture was refluxed for 6 h. Completion of the reaction was monitored by TLC using a mixture of petroleum ether/acetone/dichloromethane (7:2:1) as the mobile phase. The reaction was quenched with 10% hydrochloric acid and extracted three times with ethyl acetate. The combined organic layers were washed twice with water and once with brine, dried over anhydrous sodium sulfate, and filtered. Removal of the solvent under reduced pressure yielded the crude phenol derivative. Purification was achieved by silica gel preparative TLC, leading to **14** with 16% yield from **58**. Light-yellow oil; *R_f_* = 0.53 (petroleum ether/acetone/dichloromethane 7:2:1). ^1^H NMR (400 MHz, CDCl_3_) δ_H_ 7.09–7.02 (m, 1H), 6.48 (s, 1H), 6.38 (s, 1H), 5.86–5.77 (m, 1H), 5.16–5.09 (m, 2H), 2.72–2.66 (m, 2H), 2.35–2.23 (m, 2H), 2.16–2.01 (m, 10H), 1.98–1.93 (m, 2H), 1.82–1.71 (m, 2H), 1.64–1.52 (m, 8H), 1.26 (s, 3H). ^13^C NMR (100 MHz, CDCl_3_) δ_C_ 171.6, 152.3, 147.8, 146.1, 134.9, 133.4, 127.5, 125.5, 124.6, 121.3, 120.6, 115.8, 112.7, 75.4, 39.6, 39.5, 37.8, 31.5, 31.0, 26.5, 24.3, 22.6, 22.3, 16.2, 16.1, 16.0. HRMS (ESI) *m*/*z* calcd for C_26_H_35_O_4_ [M – H]^−^ 411.2535, found 411.2531.

#### (2*E*,6*E*)-9-[(2*R*)-6-Hydroxy-2,8-dimethyl-3,4-dihydro-2*H*-1-benzopyran-2-yl]-6-methylnona-2,6-dienoic Acid (**16**)

**16** was obtained from **59** with 31% yield using the two-step procedure described above for **58**. Light-yellow oil; *R_f_* = 0.47 (petroleum ether/acetone/dichloromethane 7:2:1). ^1^H NMR (400 MHz, CDCl_3_) δ_H_ 7.08–6.96 (m, 1H), 6.48 (s, 1H), 6.38 (s, 1H), 5.84–5.78 (m, 1H), 5.19–5.13 (m, 1H), 2.71–2.65 (m, 2H), 2.35–2.29 (m, 2H), 2.17–2.07 (m, 7H), 1.79–1.72 (m, 2H), 1.69–1.47 (m, 6H), 1.26 (s, 3H). ^13^C NMR (100 MHz, CDCl_3_) δ_C_ 171.4, 152.1, 147.8, 146.1, 133.5, 127.5, 125.7, 121.4, 120.6, 115.8, 112.7, 75.4, 39.6, 37.8, 31.5, 30.9, 24.2, 22.6, 22.3, 16.2, 15.9. HRMS (ESI) *m*/*z* calcd for C_21_H_27_O_4_ [M – H]^−^ 343.1909, found 343.1906.

#### (2*E*)-5-[(2*R*)-6-Hydroxy-2,8-dimethyl-3,4-dihydro-2*H*-1-benzopyran-2-yl]pent-2-enoic Acid (**17**)

**17** was obtained with 17% yield from **60** using the two-step procedure described above for **58**. Light-yellow oil; 17% total yield; *R_f_* = 0.36 (petroleum ether/acetone/dichloromethane 7:2:1). ^1^H NMR (400 MHz, CDCl_3_) δ_H_ 7.17–7.08 (m, 1H), 6.48 (s, 1H), 6.39 (s, 1H), 5.84 (d, *J* = 15.6 Hz, 1H), 2.77–2.64 (m, 2H), 2.44–2.38 (m, 2H), 2.11 (s, 3H), 1.87–1.60 (m, 4H), 1.26 (s, 3H). ^13^C NMR (100 MHz, CDCl_3_) δ_C_ 171.5, 152.5, 148.0, 145.7, 127.6, 121.1, 120.5, 115.9, 112.7, 74.9, 37.9, 31.6, 26.8, 23.9, 22.5, 16.2. HRMS (ESI) *m*/*z* calcd for C_16_H_19_O_4_ [M – H]^−^ 275.1283, found 275.1281.

#### (*R*,*E*)-5-(6-Hydroxy-2,8-dimethylchroman-2-yl)-2-methylpent-2-enoic Acid (**18**)

To a solution of **61** (48 mg, 0.1 mmol, 1 equiv) in a mixture of water/THF (2:1; 3 mL) was added potassium hydroxide (337 mg). The mixture was stirred at 70 °C for 18 h. Then, the reaction was quenched with an aqueous solution of hydrochloric acid (1 M). The resulting mixture was extracted three times with diethyl ether. The combined organic layers were washed with water and brine, dried over anhydrous sodium sulfate, filtered, and concentrated under reduced pressure. The residue was purified by preparative TLC eluted with a petroleum ether/acetone (75:25) mixture to afford the desired product **18** with 20% yield. Yellow oil; *R_f_* = 0.4 (petroleum ether/acetone 7:3). ^1^H NMR (400 MHz, CDCl_3_) δ_H_ 6.92 (td, *J* = 1.4 Hz, 7.5 Hz, 1H), 6.48 (d, *J* = 2.5 Hz, 1H), 6.38 (d, *J* = 3.0 Hz, 1H), 2.74–2.69 (m, 2H), 2.36 (dd, *J* = 7.8 Hz, 15.6 Hz, 2H), 2.12 (s, 3H), 1.83 (s, 3H), 1.81–1.73 (m, 3H), 1.68–1.61 (m, 1H), 1.27 (s, 3H). ^13^C NMR (100 MHz, CDCl_3_) δ_C_ 172.9, 148.0, 145.8, 145.2, 127.5, 121.2, 115.9, 112.7, 75.0, 38.4, 31.6, 29.4, 23.9, 23.4, 22.5, 16.2, 12.0. IR (ATR) ν_max_ 3365, 2925, 1682, 1639, 1607, 1470, 1426, 1378, 1291, 1216, 1145, 1096, 853 cm^–1^. HRMS (ESI) *m*/*z* calcd for C_17_H_21_O_4_ [M – H]^−^ 289.1440, found 289.1435.

#### 2-((4*E*,8*E*)-11-((*R*)-6-Acetoxy-2,8-dimethylchroman-2-yl)-4,8-dimethylundeca-4,8-dien-1-ylidene)propane-1,3-diyl Diacetate (**65**)

To a solution of δ-AC (**27d**) (289 mg, 0.67 mmol, 1 equiv) in pyridine (15 mL) was added acetic anhydride (431 μL, 3.37 mmol, 5 equiv). The reaction mixture was stirred at room temperature for 24 h. Then, the reaction was quenched with a saturated aqueous solution of sodium bicarbonate. The resulting mixture was extracted three times with ethyl acetate. The combined organic layers were washed with water and brine, dried over anhydrous sodium sulfate, filtered, and concentrated under reduced pressure. The residue was purified by column chromatography on silica gel eluted with a petroleum ether/acetone (9:1) mixture to afford the desired product **65** with 85% yield. Pale-yellow oil; *R_f_* = 0.48 (petroleum ether/acetone 85:15). ^1^H NMR (400 MHz, CDCl_3_) δ_H_ 6.66 (s, 1H), 6.61 (s, 1H), 5.75 (t, *J* = 7.4 Hz, 1H), 5.14–5.09 (m, 2H), 4.64 (s, 2H), 4.55 (s, 2H), 2.75–2.71 (m, 2H), 2.26–2.21 (m, 2H), 2.25 (s, 3H), 2.14–2.01 (m, 7H), 2.12 (s, 3H), 2.05 (s, 6H), 1.98–1.94 (m, 2H), 1.83–1.70 (m, 4H), 1.66–1.53 (m, 2H), 1.58 (s, 3H), 1.27 (s, 3H). ^13^C NMR (100 MHz, CDCl_3_) δ_C_ 171.6, 170.9, 170.5, 149.8, 142.6, 136.5, 135.3, 133.9, 129.1, 127.5, 125.3, 124.3, 121.3, 121.0, 119.2, 76.0, 66.8, 59.9, 40.0, 39.7, 39.1, 31.0, 26.7, 26.3, 24.2, 22.5, 22.2, 21.2, 21.1 (2C), 16.3, 16.0 (2C). IR (ATR) ν_max_ 2924, 2853, 1739, 1475, 1368, 1204, 1020, 801 cm^–1^. MS (EI) *m*/*z* calcd for C_33_H_46_O_7_ [M^+•^] 554.3, found 554.5.

#### 2-(11-((*R*)-6-Acetoxy-2,8-dimethylchroman-2-yl)-4,8-dimethylundecyl)propane-1,3-diyl Diacetate (**66**)

To a solution of **65** (140 mg, 0.25 mmol, 1 equiv) in ethyl acetate (10 mL) was added under a nitrogen atmosphere palladium over charcoal (loading 10 wt %), and then, hydrogen was bubbled in the organic phase. The reaction mixture was stirred at room temperature for 40 min under a hydrogen atmosphere. Completion of the reaction was monitored by ^1^H NMR. The resulting mixture was filtered on Celite, washed with ethyl acetate, and concentrated under reduced pressure. The residue was purified by column chromatography on silica gel eluted with a petroleum ether/acetone (95:5) mixture to afford the desired product **66** as a diastereomeric mixture with 52% yield. Pale-yellow oil; *R_f_* = 0.50 (petroleum ether/acetone 8:2). ^1^H NMR (400 MHz, CDCl_3_) δ_H_ 6.66 (s, 1H), 6.60 (s, 1H), 4.04 (qd, *J* = 5.8, 11.1 Hz, 4H), 2.74–2.67 (m, 2H), 2.25 (s, 3H), 2.13 (s, 3H), 2.05 (s, 6H), 2.01–1.97 (m, 1H), 1.83–1.68 (m, 2H), 1.60–1.50 (m, 2H), 1.44–1.28 (m, 10H), 1.25 (s, 6H), 1.16–0.99 (m, 5H), 0.85–0.83 (m, 6H). ^13^C NMR (100 MHz, CDCl_3_) δ_C_ 171.3 (2C), 170.5, 149.9, 142.5, 127.4, 121.2, 121.0, 119.2, 76.2, 64.5, 64.4, 40.3 (2C), 37.6–37.2 (7C), 32.8 (2C), 31.0 (2C), 28.6, 24.6, 24.4, 22.6, 21.2, 21.1, 21.0 (2C), 19.8–19.7 (4C), 16.3. MS (EI) *m*/*z* calcd for C_33_H_52_O_7_ [M^+•^] 560.4, found 560.5.

#### 2-(11-((*R*)-6-Hydroxy-2,8-dimethylchroman-2-yl)-4,8-dimethylundecyl)propane-1,3-diol (**26**)

To a solution of **66** (43 mg, 0.08 mmol, 1 equiv) in methanol (3 mL) was added under a nitrogen atmosphere a solution of MeONa 0.1 M (2.33 mL, 0.23 mmol, 3 equiv). The reaction mixture was stirred at room temperature for 40 min under a nitrogen atmosphere. Then, the reaction was quenched with an aqueous solution of hydrochloric acid (1 M). The resulting mixture was extracted three times with dichloromethane. The combined organic layers were washed with water and brine, dried over anhydrous sodium sulfate, filtered, and concentrated under reduced pressure to afford the desired product **26** with 99% yield. Pale-yellow oil; *R_f_* = 0.15 (petroleum ether/acetone 85:15). ^1^H NMR (400 MHz, CDCl_3_) δ_H_ 6.47 (s, 1H), 6.38 (s, 1H), 3.85–3.83 (m, 2H), 3.69–3.65 (m, 2H), 2.94 (bs, 2H, OH), 2.70–2.65 (m, 2H), 2.11 (s, 3H), 1.80–1.70 (m, 3H), 1.56–1.52 (m, 1H), 1.45–1.15 (m, 19H), 1.14–1.00 (m, 4H), 0.84–0.83 (m, 6H). ^13^C NMR (100 MHz, CDCl_3_) δ_C_ 148.1, 145.9, 127.3, 121.4, 115.8, 112.7, 75.6 (3C), 66.7 (2C), 41.9, 39.5 (4C), 37.5–37.1 (9C), 32.8–32.5 (6C), 31.5–31.4 (4C), 29.8, 28.1, 24.8 (2C), 24.6–24.3 (6C), 22.6, 21.1–20.8 (4C), 20.0–19.8 (5C), 16.2. IR (ATR) ν_max_ 3361, 2925, 2855, 1466, 1377, 1220, 1032, 855 cm^–1^. HRMS (ESI) *m*/*z* calcd for C_27_H_45_O_4_ [M – H]^−^ 433.3318, found 433.3313.

#### 2-((4*E*,8*E*)-11-((*R*)-6-Hydroxy-2,8-dimethyl-5,7-bis((4-methylpiperazin-1-yl)methyl)chroman-2-yl)-4,8-dimethylundeca-4,8-dien-1-ylidene)propane-1,3-diol (**40**)

To a solution of **27d** (100 mg, 0.24 mmol, 1 equiv) in methanol (5 mL) under a nitrogen atmosphere was added the Mannich alkylating reagent (4.8 mmol, 20 equiv), freshly prepared from paraformaldehyde and N-methylpiperazine (547 mg, 4.8 mmol, 20 equiv). The reaction mixture was refluxed for 48 h. Then, the solvent was evaporated under reduced pressure. The crude residue was diluted into methyl-tert-butyl ether (MTBE). The organic layer was stirred for 30 min with an aqueous saturated solution of sodium phosphate and washed with water (3 × 20 mL) and brine. It was then dried over anhydrous sodium sulfate and filtered. Removal of the solvent under reduced pressure led to the final bis-Mannich base characterized without further purification (92% yield). *R_f_* (neutral Al_2_O_3_) = 0.35 (DCM/MeOH 9.5/0.5). ^1^H NMR (500 MHz, methanol-*d*_4_) δ_H_ 5.55 (t, *J* = 7.2 Hz, 1H), 5.14 (t, *J* = 7.2 Hz, 1H), 5.12 (t, *J* = 7.2 Hz, 1H), 4.15 (s, 2H), 4.08 (s, 2H), 3.65 (s, 2H), 3.60 (s, 2H), 2.74 (t, *J* = 5.9 Hz, 2H), 2.67–2.35 (m, 16H), 2.29 (s, 6H), 2.24–2.19 (m, 2H), 2.13 (s, 3H), 2.12–2.09 (m, 2H), 2.07–2.05 (m, 2H), 2.04–1.96 (m, 4H), 1.83–1.72 (m, 2H), 1.59 (s, 3H), 1.52 (s, 3H), 1.62–1.47 (m, 2H), 1.24 (s, 3H). ^13^C NMR (125 MHz, methanol-*d*_4_) δ_C_ 151.7, 145.8, 139.3, 135.9, 135.5, 130.7, 126.5, 125.8, 125.7, 121.3, 121.1, 118.5, 75.5, 65.6, 58.3, 55.8, 55.9 (2C), 55.5, 54.8, 53.0, 53.1 (2C), 45.9 (2C), 40.5, 40.3, 40.2, 32.7, 27.6, 27.1, 24.3, 22.3, 21.2, 16.0, 16.1, 12.0. HRMS (FAB) *m*/*z* calcd for C_33_H_44_O_6_ [M + H]^+^ 653.5000, found 653.4992.

#### 2-((4*E*,8*E*)-11-((*R*)-6-Hydroxy-2,8-dimethyl-5-(pyrrolidin-1-ylmethyl)chroman-2-yl)-4,8-dimethylundeca-4,8-dien-1-ylidene)propane-1,3-diol (**35**)

To a solution of **27d** (100 mg, 0.24 mmol, 1 equiv) in methanol (5 mL) under a nitrogen atmosphere, we added pyrrolidine (41 mg, 0.48 mmol, 2 equiv), either *N,N,N,N*-tetramethylmethylene diamine (TMMDA) or the iminium freshly prepared from paraformaldehyde and the corresponding secondary amine. The reaction mixture was refluxed for 5 h. Then, the solvent was evaporated under reduced pressure. The crude residue was diluted into methyl-*tert*-butyl ether (MTBE). The organic layer was stirred for 30 min with an aqueous saturated solution of sodium phosphate and washed with water (3 × 20 mL) and brine. It was then dried over anhydrous sodium sulfate and filtered. Removal of the solvent under reduced pressure led to the final Mannich base **35** with 90% yield characterized without further purification. *R_f_* (neutral Al_2_O_3_) = 0.35 (DCM/petroleum ether/MeOH 5:5:0.3). ^1^H NMR (500 MHz, acetone-*d*_6_) δ_H_ 6.39 (s, 1H), 5.47 (t, *J* = 7.2 Hz, 1H), 5.16 (t, *J* = 7.2 Hz, 1H), 5.12 (t, *J* = 7.2 Hz, 1H), 4.17 (s, 2H), 4.10 (s, 2H), 3.66 (t, *J* = 3.9 Hz, 4H), 3.78 (s, 2H), 2.67 (t, *J* = 6.8 Hz, 2H), 2.63 (t, *J* = 5.0 Hz, 4H), 2.22 (s, 3H), 2.20–2.11 (m, 4H), 2.09–2.06 (m, 2H), 2.07 (s, 3H), 2.01–1.96 (m, 4H), 1.85–1.71 (m, 4H), 1.63–1.49 (m, 8H), 1.23 (s, 3H).^13^C NMR (125 MHz, acetone-*d*_6_) δ_C_ 151.7, 145.1, 140.2, 135.5, 135.3, 127.8, 126.2, 125.4, 125.2, 119.2, 118.0, 117.0, 74.7, 65.9, 58.9, 54.2 (2C), 54.1, 46.1, 40.5, 40.3, 39.7, 32.3, 27.2, 26.7, 24.2, 24.1, 22.8, 21.1, 16.3, 16.1, 15.9. HRMS (FAB) *m*/*z* calcd for C_32_H_50_NO_4_ [M + H]^+^ 512.3735, found 512.3758.

#### 2-((4*E*,8*E*)-11-((*R*)-5-((Dimethylamino)methyl)-6-hydroxy-2,8-dimethylchroman-2-yl)-4,8-dimethylundeca-4,8-dien-1-ylidene)propane-1,3-diol (**36**)

**36** was obtained from **27d** (100 mg, 0.24 mmol, 1 equiv), paraformaldehyde, and TMMDA (68 μL, 0.48 mmol, 2 equiv) with 90% yield using the same method as described above for **35**. *R_f_* (neutral Al_2_O_3_) = 0.40 (DCM/petroleum ether/MeOH 5:5:0.3). ^1^H NMR (500 MHz, acetone-*d*_6_) δ_H_ 6.39 (s, 1H), 5.47 (t, *J* = 7.2 Hz, 1H), 5.16 (t, *J* = 7.2 Hz, 1H), 5.13 (t, *J* = 7.2 Hz, 1H), 4.17 (s, 2H), 4.10 (s, 2H), 3.57 (s, 2H), 2.67 (t, *J* = 6.8 Hz, 2H), 2.29 (s, 6H), 2.22 (s, 3H), 2.20–2.11 (m, 4H), 2.09–2.06 (m, 2H), 2.07 (s, 3H), 2.01–1.96 (m, 4H), 1.85–1.71 (m, 2H), 1.63–1.49 (m, 2H), 1.59 (s, 6H), 1.23 (s, 3H). ^13^C NMR (125 MHz, acetone-*d*_6_) δ_C_ 151.7, 145.1, 140.2, 135.5, 135.3, 127.8, 126.3, 125.4, 125.2, 119.7, 117.7, 117.0, 74.7, 66.9, 58.9, 58.2, 44.6 (2C), 40.5, 40.3, 39.7, 32.3, 27.2, 26.7, 24.1, 22.8, 21.1, 16.3, 16.1, 15.9. HRMS (FAB) *m*/*z* calcd for C_30_H_48_NO_4_ [M + H]^+^ 486.3577, found 486.3567.

#### 2-((4*E*,8*E*)-11-((*R*)-6-Hydroxy-2,8-dimethyl-5-(morpholinomethyl)chroman-2-yl)-4,8-dimethylundeca-4,8-dien-1-ylidene)propane-1,3-diol (**37**)

**37** was obtained from **27d** (100 mg, 0.24 mmol, 1 equiv), paraformaldehyde, and morpholine (49 mg, 0.48 mmol, 2 equiv) with 95% yield using the same method as described above for **35**. *R_f_* (neutral Al_2_O_3_) = 0.40 (DCM/petroleum ether/MeOH 5:4:1). ^1^H NMR (500 MHz, acetone-*d*_6_) δ_H_ 6.41 (s, 1H), 5.47 (t, *J* = 7.2 Hz, 1H), 5.16 (t, *J* = 7.2 Hz, 1H), 5.12 (t, *J* = 7.2 Hz, 1H), 4.17 (s, 2H), 4.10 (s, 2H), 3.66 (t, *J* = 3.9 Hz, 4H), 3.65 (s, 2H), 2.67 (t, *J* = 6.8 Hz, 2H), 2.52 (s, 4H), 2.22 (s, 3H), 2.20–2.11 (m, 4H), 2.09–2.06 (m, 2H), 2.07 (s, 3H), 2.01–1.96 (m, 4H), 1.85–1.71 (m, 2H), 1.63–1.49 (m, 2H), 1.59 (s, 6H), 1.23 (s, 3H). ^13^C NMR (125 MHz, acetone-*d*_6_) δ_C_ 151.3, 145.4, 140.2, 135.5, 135.3, 127.8, 126.4, 125.5, 125.2, 120.0, 117.0, 116.7, 74.7, 67.3 (2C), 65.9, 58.9, 56.9, 53.6 (2C), 46.1, 40.5, 40.3, 39.7, 32.3, 27.2, 26.7, 24.2, 22.8, 21.1, 16.3, 16.1, 15.9. HRMS (FAB) *m*/*z* calcd for C_32_H_50_NO_5_ [M + H]^+^ 528.3684, found 528.3675.

#### 2-((4*E*,8*E*)-11-((*R*)-6-Hydroxy-2,8-dimethyl-5-(piperidin-1-ylmethyl)chroman-2-yl)-4,8-dimethylundeca-4,8-dien-1-ylidene)propane-1,3-diol (**38**)

**38** was obtained from **27d** (100 mg, 0.24 mmol, 1 equiv), paraformaldehyde, and piperidine (47 mg, 0.48 mmol, 2 equiv) with 95% yield using the same method as described above for **35**. *R_f_* (neutral Al_2_O_3_) = 0.35 (DCM/petroleum ether/MeOH 5:4:1). ^1^H NMR (500 MHz, acetone-*d*_6_) δ_H_ 6.38 (s, 1H), 5.47 (t, *J* = 7.2 Hz, 1H), 5.16 (t, *J* = 7.2 Hz, 1H), 5.12 (t, *J* = 7.2 Hz, 1H), 4.17 (s, 2H), 4.10 (s, 2H), 3.60 (s, 2H), 2.64 (t, *J* = 6.8 Hz, 2H), 2.58–2.39 (m, 4H), 2.22 (s, 3H), 2.20–2.11 (m, 4H), 2.09–2.06 (m, 2H), 2.07 (s, 3H), 2.01–1.96 (m, 4H), 1.85–1.71 (m, 2H), 1.63–1.43 (m, 14H), 1.23 (s, 3H). ^13^C NMR (125 MHz, acetone-*d*_6_) δ_C_ 151.8, 145.2, 140.2, 135.5, 135.3, 127.8, 126.2, 125.5, 125.2, 119.7, 117.0, 117.2, 74.7, 66.0, 58.9, 57.5, 54.4 (2C), 46.1, 40.5, 40.3, 39.7, 32.3, 27.2, 26.8, 26.7 (2C), 24.8, 24.2, 22.8, 21.0, 16.3, 16.1, 15.9. HRMS (FAB) *m*/*z* calcd for C_33_H_51_NO_4_ [M^+•^] 525.3818, found 525.3817.

#### 2-((4*E*,8*E*)-11-((*R*)-6-Hydroxy-2,8-dimethyl-5-((4-methylpiperazin-1-yl)methyl)chroman-2-yl)-4,8-dimethylundeca-4,8-dien-1-ylidene)propane-1,3-diol (**39**)

**39** was obtained from **27d** (100 mg, 0.24 mmol, 1 equiv), paraformaldehyde, and N-methylpiperazine (55 mg, 0.48 mmol, 2 equiv) with 92% yield using the same method as described above for **35**. *R_f_* (neutral Al_2_O_3_) = 0.35 (DCM/petroleum ether/MeOH 5:4.5:0.5). ^1^H NMR (500 MHz, acetone-*d*_6_) δ_H_ 6.39 (s, 1H), 5.47 (t, *J* = 7.2 Hz, 1H), 5.15 (t, *J* = 7.2 Hz, 1H), 5.12 (t, *J* = 7.2 Hz, 1H), 4.17 (s, 2H), 4.10 (s, 2H), 3.63 (s, 2H), 2.65 (t, *J* = 6.8 Hz, 2H), 2.55–2.25 (m, 8H), 2.22 (s, 3H), 2.20–2.11 (m, 4H), 2.09–2.06 (m, 2H), 2.07 (s, 3H), 2.01–1.96 (m, 4H), 1.85–1.71 (m, 2H), 1.63–1.49 (m, 2H), 1.59 (s, 6H), 1.23 (s, 3H). ^13^C NMR (125 MHz, acetone-*d*_6_) δ_C_ 151.5, 145.2, 140.2, 135.4, 135.3, 127.6, 126.4, 125.5, 125.2, 119.8, 117.0 (2C), 74.7, 65.9, 58.9, 56.7, 55.8 (2C), 53.1 (2C), 46.1, 40.5, 40.3, 39.7, 32.3, 27.2, 26.7, 24.1, 22.8, 21.1, 16.3, 16.1, 15.9. HRMS (FAB) *m*/*z* calcd for C_33_H_52_N_2_O_4_ [M + H]^+^ 541.3999, found 541.3992.

#### (*R*)-3-Acetyl-7-((3*E*,7*E*)-13-hydroxy-12-(hydroxymethyl)-4,8-dimethyltrideca-3,7,11-trien-1-yl)-5,7,10-trimethyl-8,9-dihydropyrano[2,3-*g*]chromen-2(7*H*)-one (**41**)

To a solution of aldehyde **33** (0.11 mmol, 1 equiv) in ethanol (3 mL), piperidine (11 μL, 0.11 mmol, 1 equiv) and ethyl acetoacetate (17 μL, 0.132 mmol, 1.2 equiv) were added. The reaction mixture was refluxed until completion of the reaction monitored by TLC. After cooling to room temperature, the reaction mixture was diluted with ether. The organic layer was washed with water to pH 7 and brine, dried over anhydrous sodium sulfate, filtered, and concentrated under reduced pressure. The crude residue was purified by silica preparative TLC to yield the corresponding coumarine **41** with 89% yield. *R_f_* = 0.35 (petroleum ether/DCM/acetone 5:3:2). ^1^H NMR (500 MHz, acetone-*d*_6_) δ_H_ 8.64 (s, 1H), 5.47 (t, *J* = 7.2 Hz,1H), 5.18 (t, *J* = 7.2 Hz,1H), 5.13 (t, *J* = 7.2 Hz,1H), 4.16 (d, *J* = 4.8 Hz, 2H), 4.09 (d, *J* = 4.8 Hz, 2H), 3.67 (t, *J* = 5.3 Hz, 1H), 3.59 (t, *J* = 5.3 Hz, 1H), 2.85 (t, *J* = 7.0 Hz, 2H), 2.61 (s, 3H), 2.41 (s, 3H), 2.29 (s, 3H), 2.23–2.14 (m, 4H), 2.11–2.06 (m, 2H), 2.01–1.96 (m, 4H), 1.96–1.81 (m, 2H), 1.73–1.63 (m, 2H), 1.58 (s, 3H), 1.61 (s, 3H), 1.33 (s, 3H). ^13^C NMR (125 MHz, acetone-*d*_6_) δ_C_ 195.7, 159.7, 149.0, 148.2, 145.1, 140.2, 135.7, 135.3, 129.6, 127.7, 125.2, 125.1, 123.6, 122.9, 122.0, 116.7, 76.9, 65.9, 58.9, 40.4, 40.3, 39.9, 31.5, 30.4, 27.2, 26.6, 24.1, 22.8, 22.1, 16.1, 16.0, 10.8, 10.6. IR (ATR) ν_max_ 3364, 2925, 2855, 1726, 1550, 1232 cm^–1^. HRMS (FAB) *m*/*z* calcd for C_33_H_44_O_6_ [M^+•^] 536.3138, found 536.3133.

#### (*R*)-2-Acetyl-8-((3*E*,7*E*)-13-hydroxy-12-(hydroxymethyl)-4,8-dimethyltrideca-3,7,11-trien-1-yl)-6,8-dimethyl-9,10-dihydropyrano[3,2-*f*]chromen-3(8*H*)-one (**42**)

**42** was obtained from **34** with 93% yield using the same method as described above for **41**. *R_f_* = 0.35 (petroleum ether/DCM/acetone 5:3:2). ^1^H NMR (500 MHz, acetone-*d*_6_) δ_H_ 8.57 (s, 1H), 7.08 (s, 1H), 5.47 (t, *J* = 7.2 Hz, 1H), 5.18 (t, *J* = 7.2 Hz, 1H), 5.13 (t, *J* = 7.2 Hz, 1H), 4.16 (d, *J* = 4.8 Hz, 2H), 4.09 (d, *J* = 4.8 Hz, 2H), 3.66 (t, *J* = 5.3 Hz, 1H), 3.57 (t, *J* = 5.3 Hz, 1H), 3.09–3.06 (m, 2H), 2.60. (s, 3H), 2.30 (s, 3H), 2.23–2.14 (m, 4H), 2.11–2.06 (m, 2H), 2.01–1.96 (m, 4H), 1.96–1.81 (m, 2H), 1.73–1.63 (m, 2H), 1.61 (s, 3H), 1.58 (s, 3H), 1.35 (s, 3H). ^13^C NMR (125 MHz, acetone-*d*_6_) δ_C_ 196.7, 159.8, 150.7, 149.4, 144.0, 140.2, 136.0, 135.7, 135.3, 127.7, 125.2, 125.1, 123.6, 119.1, 116.6, 116.0, 77.0, 65.9, 58.9, 40.5, 40.4, 39.9, 31.5, 30.4, 27.2, 26.6, 23.9, 22.8, 19.5, 17.2, 16.1, 16.0. HRMS (ESI) *m*/*z* calcd for C_32_H_42_NaO_6_ [M + Na]^+^ 545.2874, found 545.2870.

#### Ethyl-(*R*)-8-((3*E*,7*E*)-13-hydroxy-12-(hydroxymethyl)-4,8-dimethyltrideca-3,7,11-trien-1-yl)-6,8-dimethyl-3-oxo-3,8,9,10-tetrahydropyrano[3,2-*f*]chromene-2-carboxylate (**43**)

**43** was obtained from **34** and diethyl malonate with 90% yield using the same method as described above for **41**. *R_f_* = 0.30 (petroleum ether/DCM/acetone, 5:3:2). ^1^H NMR (500 MHz, acetone-*d*_6_) δ_H_ 8.63 (s, 1H), 7.05 (s, 1H), 5.45 (t, *J* = 7.2 Hz,1H), 5.18 (t, *J* = 7.2 Hz,1H), 5.13 (t, *J* = 7.2 Hz, 1H), 4.31 (q, *J* = 7.1 Hz, 2H), 4.16 (d, *J* = 4.8 Hz, 2H), 4.09 (d, *J* = 4.8 Hz, 2H), 3.71 (t, *J* = 5.3 Hz, 1H), 3.63 (t, *J* = 5.3 Hz, 1H), 3.06 (t, *J* = 6.3 Hz, 2H), 2.30 (s, 3H), 2.23–2.14 (m, 4H), 2.11–2.06 (m, 2H), 2.01–1.96 (m, 4H), 1.96–1.81 (m, 2H), 1.73–1.63 (m, 2H), 1.61 (s, 3H), 1.58 (s, 3H), 1.36 (t, *J* = 7.1 Hz, 3H), 1.35 (s, 3H). ^13^C NMR (125 MHz, acetone-*d*_6_) δ_C_ 164.2, 156.9, 150.7, 149.4, 145.2, 140.2, 136.5, 135.7, 135.3, 127.7, 125.2, 125.1, 118.6, 117.4, 116.6, 115.4, 76.9, 65.9, 61.9, 58.9, 40.5, 40.4, 39.9, 31.0, 27.2, 26.6, 23.9, 22.8, 19.4, 17.2, 16.1, 15.9, 14.5. HRMS (ESI) *m*/*z* calcd for C_33_H_44_NaO_7_ [M + Na]^+^ 575.2979, found 575.2969.

#### Ethyl-4-(((2*R*)-2-((3*E*,7*E*)-11-(2-(3,4-dimethoxyphenyl)-1,3-dioxan-5-ylidene)-4,8-dimethylundeca-3,7-dien-1-yl)-2,8-dimethylchroman-6-yl)oxy)butanoate (**67**)

Under a nitrogen atmosphere, a solution of **44** (1 equiv) in dry THF (0.05 M) was added dropwise to a suspension of sodium hydride (60% wt, 3 equiv) in 2 mL of dry THF cooled at 0 °C. After 1 h of stirring at this temperature, 2.4 equiv of ethyl bromobutyrate was added. The reaction mixture was stirred further for 3 h. Then, it was diluted with ethyl acetate (15 mL). The organic layer was washed with water (4 × 15 mL) and brine (15 mL) and dried over anhydrous sodium sulfate. After filtration and removal of the solvent under reduced pressure, the crude residue was purified by silica gel column chromatography eluted with cyclohexane/EtOAc, leading to **67** with 70% yield. *R_f_* = 0.35 (petroleum ether/DCM 7:3). ^1^H NMR (500 MHz, acetone-*d*_6_) δ_H_ 7.02 (d, *J* = 1.8 Hz, 1H), 6.97 (d, *J* = 8.2 Hz, 1H), 6.89 (d, *J* = 8.2 Hz, 1H), 6.56 (d, *J* = 3.2 Hz, 1H), 6.46 (d, *J* = 3.2 Hz, 1H), 5.58 (s, 1H), 5.37 (t, *J* = 7.2 Hz, 1H), 5.17 (t, *J* = 7.2 Hz, 1H), 5.15 (t, *J* = 7.2 Hz, 1H), 4.79 (d, *J* = 13.0 Hz, 1H), 4.58 (s, 2H), 4.49 (d, *J* = 13.0 Hz, 1H), 4.34 (d, *J* = 13.0 Hz, 1H), 4.30 (dd, *J* = 1.6 Hz, 13.0 Hz, 1H), 4.09 (t, *J* = 7.2 Hz, 3H), 3.91 (t, *J* = 6.2 Hz, 2H), 3.79 (s, 6H), 2.72 (t, *J* = 6.8 Hz, 2H), 2.46 (t, *J* = 7.2 Hz, 2H), 2.26–2.05 (m, 11H), 2.03–1.96 (m, 4H), 1.86–1.72 (m, 2H), 1.66–1.52 (m, 2H), 1.60 (s, 6H), 1.27 (s, 3H), 1.24 (t, *J* = 7.2 Hz, 3H), 1.22 (t, *J* = 7.2 Hz, 3H). ^13^C NMR (125 MHz, acetone-*d*_6_) δ_C_ 173.4, 152.5, 150.5, 149.9, 146.7, 136.4, 134.8, 132.7, 131.0, 127.3, 125.8, 125.6, 125.4, 121.7, 119.6, 116.2, 112.6, 111.9, 110.8, 102.2, 75.9, 72.7, 67.7, 66.6, 60.6, 56.0 (2C), 40.3, 40.2, 40.0, 32.1, 31.2, 27.1, 25.8, 25.6, 24.4, 23.1, 22.8, 16.4, 16.1, 15.9, 14.5. HRMS (ESI) *m*/*z* calcd for C_40_H_58_NaO_8_ [M + Na]^+^ 713.4024, found 713.4023.

#### 2-(((2*R*)-2-((3*E*,7*E*)-11-(2-(3,4-Dimethoxyphenyl)-1,3-dioxan-5-ylidene)-4,8-dimethylundeca-3,7-dien-1-yl)-2,8-dimethylchroman-6-yl)oxy)acetamide (**69**)

**69** was obtained from **44** and bromoacetamide with 66% yield after purification through column chromatography eluted with petroleum ether/DCM. *R_f_* = 0.30 (ethyl acetate/cyclohexane 5:5). ^1^H NMR (500 MHz, acetone-*d*_6_) δ_H_ 7.08 (bs, 2H), 7.01 (d, *J* = 1.6 Hz, 1H), 6.96 (dd, *J* = 1.6 Hz, 8.3 Hz, 1H), 6.89 (d, *J* = 8.3 Hz, 1H), 6.63 (d, *J* = 2.8 Hz, 1H), 6.53 (d, *J* = 2.8 Hz, 1H), 5.58 (s, 1H), 5.37 (t, *J* = 7.2 Hz, 1H), 5.17 (t, *J* = 7.2 Hz, 1H), 5.14 (t, *J* = 7.2 Hz, 1H), 4.79 (d, *J* = 13.0 Hz, 1H), 4.48 (d, *J* = 13.0 Hz, 1H), 4.34 (d, *J* = 13.0 Hz, 1H), 4.30 (d, *J* = 13.0 Hz, 1H), 4.31 (s, 2H), 3.79 (s, 6H), 2.73 (t, *J* = 6.8 Hz, 2H), 2.12 (s, 3H), 2.24–1.96 (m, 10H), 1.88–1.69 (m, 2H), 1.67–1.52 (m, 2H), 1.60 (s, 6H), 1.26 (s, 3H). ^13^C NMR (125 MHz, acetone-*d*_6_) δ_C_ 171.2, 151.4, 150.4, 149.9 (2C), 147.4, 135.4, 134.8, 132.7, 131.0, 127.6, 125.8, 125.6, 125.4, 121.9, 119.6, 116.4, 113.4, 111.9, 110.8, 102.2, 76.1, 72.7, 68.5, 66.6, 56.0 (2C), 40.3, 40.2, 40.0, 32.2, 27.2, 25.8, 24.4, 23.1, 22.9, 16.4, 16.1, 15.9. HRMS (ESI) *m*/*z* calcd for C_38_H_51_NNaO_7_ [M + Na]^+^ 656.3558, found 656.3551.

#### 4-(((2*R*)-2-((3*E*,7*E*)-11-(2-(3,4-Dimethoxyphenyl)-1,3-dioxan-5-ylidene)-4,8-dimethylundeca-3,7-dien-1-yl)-2,8-dimethylchroman-6-yl)oxy)butanoic Acid (**45**)

LiOH (93 μL of a 10% aqueous solution, 3 equiv) was added to a solution of **67** (90 mg, 0.13 mmol, 1 equiv) in THF (3 mL). The reaction mixture was stirred at room temperature for 3 h. It was then diluted with water and acidified with 1 N HCl down to pH 4–5. The aqueous layer was extracted with 3 × 10 mL of EtOAc. The combined organic extracts were washed with brine, dried over anhydrous sodium sulfate, filtered, and concentrated to dryness under reduced pressure. **45** was obtained with 85% yield. *R_f_* = 0.30 (acetone/petroleum ether 4:6). ^1^H NMR (500 MHz, acetone-*d*_6_) δ_H_ 7.02 (d, *J* = 1.8 Hz, 1H), 6.97 (d, *J* = 8.2 Hz, 1H), 6.89 (d, *J* = 8.2 Hz, 1H), 6.56 (d, *J* = 3.2 Hz, 1H), 6.46 (d, *J* = 3.2 Hz, 1H), 5.58 (s, 1H), 5.37 (t, *J* = 7.2 Hz, 1H), 5.17 (t, *J* = 7.2 Hz, 1H), 5.15 (t, *J* = 7.2 Hz, 1H), 4.79 (d, *J* = 13.0 Hz, 1H), 4.58 (s, 2H), 4.49 (d, *J* = 13.0 Hz, 1H), 4.34 (d, *J* = 13.0 Hz, 1H), 4.30 (dd, *J* = 13.0 Hz, 1.6 Hz, 1H), 4.09 (q, *J* = 7.2 Hz, 2H), 3.92 (t, *J* = 6.2 Hz, 2H), 3.79 (s, 6H), 2.72 (t, *J* = 6.8 Hz, 2H), 2.48 (t, *J* = 7.2 Hz, 2H), 2.26–2.05 (m, 8H), 2.11 (s, 3H), 2.03–1.96 (m, 4H), 1.84–1.66 (m, 2H), 1.66–1.52 (m, 2H), 1.60 (s, 6H), 1.27 (s, 3H). ^13^C NMR (125 MHz, acetone-*d*_6_) δ_C_ 174.4, 152.5, 150.4, 149.8, 146.7, 135.4, 134.8, 132.7, 131.0, 127.3, 125.7, 125.6, 125.4, 121.7, 119.6, 116.2, 112.7, 111.8, 110.7, 102.2, 75.9, 72.7, 67.7, 66.5, 56.0, 55.9, 40.3, 40.2, 40.0, 32.1, 30.8, 27.5, 27.1, 25.8, 25.6, 24.4, 23.1, 22.8, 16.4, 16.1, 15.9. HRMS (ESI) *m*/*z* calcd for C_40_H_54_NaO_8_ [M + Na]^+^ 685.3711, found 685.3710

#### (2*R*)-2-((3*E*,7*E*)-4,8-Dimethyl-11-(2-(4-nitrophenyl)-1,3-dioxan-5-ylidene)undeca-3,7-dien-1-yl)-2,8-dimethylchroman-6-ol (**70**)

To a stirred solution of **27d** (216 mg, 0.5 mmol) in dry THF (5 mL) were added pTSA (22 mg, 0.12 mmol, 0.23 equiv) and a solution of 4-nitrobenzaldehyde dimethyl acetal (198 mg, 1 mmol, 2 equiv) in dry THF (5 mL). The reaction mixture was refluxed for 5 h. The reaction mixture was cooled down to room temperature, and EtOAc (30 mL) was added. The organic layer was washed with a saturated aqueous solution of NaHCO_3_ (20 mL) and brine (20 mL), dried over Na_2_SO_4_, and evaporated to dryness. Purification by column chromatography on silica gel and eluting with a petroleum ether/acetone (PE/acetone) mixture afforded the desired protected product **70** with 78% yield. *R_f_* = 0.3 (petroleum ether/acetone 85:15). ^1^H NMR (500 MHz, acetone-*d*_6_) δ_H_ 8.24 (d, *J* = 8.9 Hz, 2H), 7.73 (d, *J* = 8.9 Hz, 2H), 7.44 (s, 1H), 6.46 (d, *J* = 2.8 Hz, 1H), 6.36 (d, *J* = 2.8 Hz, 1H), 5.82 (s, 1H), 5.43 (t, *J* = 7.2 Hz,1H), 5.17 (t, *J* = 7.2 Hz, 1H), 5.14 (t, *J* = 7.2 Hz, 1H), 4.87 (d, *J* = 13.0 Hz, 1H), 4.58 (d, *J* = 13.0 Hz, 1H), 4.44. (d, *J* = 13.0 Hz, 1H), 4.38 (d, *J* = 13.0 Hz, 1H), 2.67 (t, *J* = 6.8 Hz, 2H), 2.09 (s, 3H), 2.24–2.07 (m, 6H), 2.03–1.96 (m, 4H), 1.88–1.69 (m, 2H), 1.67–1.52 (m, 2H), 1.60 (s, 6H), 1.27 (s, 3H). ^13^C NMR (125 MHz, acetone-*d*_6_) δ_C_ 150.4, 148.8, 146.3, 145.6, 135.2, 134.6, 130.2, 128.2 (2C), 127.1, 126.4, 125.6, 125.2, 123.9 (2C), 121.6, 116.3, 113.3, 100.3, 75.7, 72.7, 66.6, 40.2, 40.1, 39.8, 32.0, 27.0, 25.7, 24.2, 22.9, 22.7, 16.1, 15.9, 15.7. HRMS (FAB) *m*/*z* calcd for C_34_H_43_NNaO_6_ [M + Na]^+^ 584.2983, found 584.2974.

#### (2*R*)-2-((3*E*,7*E*)-4,8-Dimethyl-11-(2-(4-nitrophenyl)-1,3-dioxan-5-ylidene)undeca-3,7-dien-1-yl)-5-(hydroxymethyl)-2,8-dimethylchroman-6-ol (**48**)

In a two-neck round-bottom flask under a nitrogen atmosphere, acetic acid (4 μL, 0.05 mmol, 0.6 equiv), boric acid (17 mg, 0.27 mmol, 3 equiv), and paraformaldehyde (48 mg, 2.62 mmol, 36 equiv) were added to a solution of the acetal **70** (50 mg, 0.09 mmol, 1 equiv) in 5 mL of dry toluene. The reaction mixture was refluxed for 12 h. After cooling at room temperature, it was diluted with toluene (20 mL). The organic layer was washed with water (20 mL) and then stirred for 30 min with a 5% aqueous solution of sodium carbonate. The organic layer was washed further with water (15 mL) and brine (15 mL), dried over anhydrous sodium sulfate, and filtered. After removal of the solvent under reduced pressure, the crude product was purified using preparative TLC eluted with a mixture of petroleum ether/acetone/dichloromethane (8:2:1), leading to **48** with 55% yield (33 mg). *R_f_* = 0.30 (petroleum ether/acetone 8:2). ^1^H NMR (400 MHz, acetone-*d*_6_) δ_H_ 8.25 (d, *J* = 8.8 Hz, 2H), 8.02 (s, 1H), 7.73 (d, *J* = 8.8 Hz, 2H), 6.48 (s, 1H), 5.82 (s, 1H), 5.43 (t, *J* = 7.2 Hz, 1H), 5.17–5.14 (m, 2H), 4.87 (d, *J* = 13.0 Hz, 1H), 4.74 (d, *J* = 4.5 Hz, 2H), 4.57 (d, *J* = 13.0 Hz, 1H), 4.44. (d, *J* = 13.0 Hz, 1H), 4.38 (d, *J* = 13.0 Hz, 1H), 4.26–4.23 (m, 1H), 2.73 (t, *J* = 6.8 Hz, 2H), 2.11 (s, 3H), 2.26–2.09 (m, 9H), 2.03–1.96 (m, 4H), 1.88–1.73 (m, 2H), 1.67–1.52 (m, 2H), 1.61 (s, 3H) 1.60 (s, 3H), 1.24 (s, 3H). ^13^C NMR (100 MHz, acetone-*d*_6_) δ_C_ 149.5, 149.0, 146.5, 145.6, 135.4, 134.8, 130.4, 128.4 (2C), 126.6, 126.4, 125.7, 125.5, 124.0 (2C), 120.2, 116.8, 100.5, 74.9, 72.9, 66.8, 58.4, 40.3 (2C), 40.0, 39.9, 32.1, 27.2, 25.8, 24.1, 22.9, 20.2, 16.3, 16.0, 15.9. HRMS (FAB) *m*/*z* calcd for C_35_H_45_NO_7_ [M]^+^ 591.3190, found 591.3177.

#### (2*R*)-2-((3*E*,7*E*)-11-(2-(3,4-Dimethoxyphenyl)-1,3-dioxan-5-ylidene)-4,8-dimethylundeca-3,7-dien-1-yl)-7-(hydroxymethyl)-2,5,8-trimethylchroman-6-ol (**46**)

To a stirred solution of **64** (152 mg, 0.25 mmol) in dry THF (20 mL) were added magnesium chloride (238 mg, 10 equiv), paraformaldehyde (249 mg, 32 equiv), and triethylamine (1.18 mL, 32 equiv) at room temperature. The mixture was stirred under reflux for 2 h. The heterogeneous mixture was cooled down to room temperature, and 1 N HCl (10 mL) and Et_2_O (30 mL) were added dropwise. The organic layer was separated, and the aqueous layer was extracted with Et_2_O (2 × 10 mL). The combined organic extracts were washed with brine (20 mL), dried over Na_2_SO_4_, and evaporated to dryness. Purification by column chromatography on silica gel, using a petroleum ether/acetone/DCM (7:2:1) mixture as the mobile phase, afforded **46** (33% yield) along with the desired formylated product **47** with 36% yield. *R_f_* = 0.68 (petroleum ether/acetone/DCM 7:2:1). Pale-yellow oil; ^1^H NMR (500 MHz, acetone-*d*_6_) δ_H_ 8.25 (s, 1H, OH), 7.02 (d, *J* = 1.8 Hz, 1H), 6.98 (dd, *J* = 1.8 Hz, 8.2 Hz, 1H), 6.89 (d, *J* = 8.2 Hz, 1H), 5.58 (s, 1H), 5.36 (t, *J* = 7.2 Hz, 1H), 5.16 (t, *J* = 7.2 Hz, 1H), 5.14 (t, *J* = 7.2 Hz, 1H), 4.92 (t, *J* = 4.5 Hz, 1H), 4.85 (d, *J* = 5.0 Hz, 2H), 4.79 (d, *J* = 13.0 Hz, 1H), 4.48 (d, *J* = 13.0 Hz, 1H), 4.33 (d, *J* = 13.0 Hz, 1H), 4.31 (d, *J* = 13.0 Hz, 1H), 3.79 (s, 3H), 3.78 (s, 3H), 2.60 (t, *J* = 6.8 Hz, 2H), 2.05 (s, 6H), 2.24–2.06 (m, 6H), 2.04–1.97 (m, 6H, H-17), 1.86–1.71 (m, 2H), 1.66–1.48 (m, 2H), 1,60 (s, 6H), 1.23 (s, 3H). ^13^C NMR (125 MHz, acetone-*d*_6_) δ_C_ 150.5, 149.9 (2C), 148.5, 145.2, 135.5, 134.8, 132.7, 131.0, 125.8, 125.6, 125.5, 122.9, 120.8, 120.8, 120.1, 119.6, 111.9, 110.8, 102.2, 74.8, 72.7, 66.6, 60.9, 56.0 (2C), 40.3, 40.0 (2C), 32.3, 27.1, 25.8, 24.1, 22.9, 21.3, 16.0, 15.9, 11.0, 10.9. HRMS (FAB) *m*/*z* calcd for C_38_H_52_O_7_ [M^+•^] 620.3713, found 620.3711.

#### 4-(((2*R*)-2-((3*E*,7*E*)-11-(2-(3,4-Dimethoxyphenyl)-1,3-dioxan-5-ylidene)-4,8-dimethylundeca-3,7-dien-1-yl)-2,8-dimethylchroman-6-yl)oxy)-4-oxobutanoic Acid (**68**)

Under a nitrogen atmosphere, succinic anhydride (175 mg, 1.79 mmol, 10.3 equiv) and triethylamine (244 μL, 1.79 mmol, 10.3 equiv) were added to a dry THF solution (4 mL) of acetal **64** (100 mg, 174 mmol, 1.0 equiv). The reaction mixture was stirred at room temperature for 20 h. It was then diluted with water and acidified to pH 4 with 1 N HCl. The aqueous layer was extracted three times with ethyl acetate (3 × 15 mL). The combined organic layers were washed once with water and brine, dried over sodium sulfate, filtered, and concentrated under reduced pressure to yield 135 mg of **68** as an oil. The product was used without further purification. *R_f_* = 0.30 (petroleum ether/acetone 3:7). ^1^H NMR (400 MHz, acetone-*d*_6_) δ_H_ 7.02 (d, *J* = 1.8 Hz, 1H), 6.97 (d, *J* = 8.2 Hz, 1H), 6.89 (d, *J* = 8.2 Hz, 1H), 6.68 (d, *J* = 3.2 Hz, 1H), 6.63 (d, *J* = 3.2 Hz, 1H), 5.58 (s, 1H), 5.37 (t, *J* = 7.2 Hz, 1H), 5.18 (t, *J* = 7.2 Hz, 1H), 5.15 (t, *J* = 7.2 Hz, 1H), 4.79 (d, *J* = 13.0 Hz, 1H), 4.49 (d, *J* = 13.0 Hz, 1H), 4.34 (d, *J* = 13.0 Hz, 1H), 4.30 (dd, *J* = 13.0 Hz, 1.6 Hz, 1H), 3.79 (s, 6H), 2.80 (t, *J* = 7.2 Hz, 2H), 2.75 (t, *J* = 7.2 Hz, 2H), 2.70 (t, *J* = 6.2 Hz, 2H), 2.25–2.08 (m, 6H), 2.13 (s, 3H), 2.03–1.96 (m, 4H), 1.90–1.72 (m, 2H), 1.68–1.52 (m, 2H), 1.61 (s, 6H), 1.29 (s, 3H). ^13^C NMR (100 MHz, acetone-*d*_6_) δ_C_ 173.8, 172.1, 150.4, 149.8 (2C), 150.2, 143.9, 135.4, 134.8, 132.7, 131.0, 127.3, 125.8, 125.6, 125.4, 122.1, 121.1, 120.2, 119.6, 111.8, 110.7, 102.2, 76.6, 72.7, 66.6, 56.0 (2C), 40.3 (2C), 40.0, 31.7, 29.5, 29.2, 27.1, 25.6, 24.4, 24.3, 22.8, 16.2, 16.1, 15.9. HRMS (FAB) *m*/*z* calcd for C_40_H_53_O_9_ [M + H]^+^ 677.3684, found 677.3674.

#### 4-(((*R*)-2-((3*E*,7*E*)-13-Hydroxy-12-(hydroxymethyl)-4,8-dimethyltrideca-3,7,11-trien-1-yl)-2,8-dimethylchroman-6-yl)oxy)-4-oxobutanoic Acid (**31**)

*para*-Toluenesulfonic acid (1.15 equiv) was added to a solution of acetal **68** (1 equiv) in THF (0.05 M). The reaction mixture was stirred at room temperature for 1 h and was diluted with EtOAc. The organic layer was washed twice with a saturated aqueous solution of NaHCO_3_, twice with water, and once with brine. It was then dried over anhydrous sodium sulfate, filtered, and concentrated under reduced pressure. The crude residue was purified by C18 flash chromatography using a water/MeOH gradient leading to **31** with 60% yield. ^1^H NMR (400 MHz, acetone-*d*_6_) δ_H_ 6.68 (d, *J* = 3.2 Hz, 1H), 6.63 (d, *J* = 3.2 Hz, 1H), 5.47 (t, *J* = 7.2 Hz, 1H), 5.18–5.14 (m, 2H), 4.17 (s, 2H), 4.09 (s, 2H), 2.85–2.72 (m, 4H), 2.71–2.68 (m, 2H), 2.21–2.08 (m, 6H), 2.13 (s, 3H), 2.03–1.96 (m, 4H), 1.90–1.75 (m, 2H), 1.68–1.52 (m, 2H), 1.61 (s, 3H), 1.59 (s, 3H), 1.29 (s, 3H). ^13^C NMR (100 MHz, acetone-*d*_6_) δ_C_ 173.6, 172.0, 150.2, 143.9, 140.1, 135.6, 135.3, 127.7, 127.3, 125.2, 122.1, 121.8, 120.2, 76.6, 65.9, 58.9, 40.5, 40.4, 40.3 (2C), 31.7, 29.7, 29.2, 27.2, 26.6, 24.4, 22.8 (2C), 16.2, 16.1, 15.9. IR (ATR) ν_max_ 3425, 2972, 2901, 1696, 1406, 1380, 1225, 1057, 835 cm^–1^. HRMS (FAB) *m*/*z* calcd for C_31_H_43_O_7_ [M – H]^−^ 527.3003, found 527.3008.

#### 2-(((*R*)-2-((3*E*,7*E*)-13-Hydroxy-12-(hydroxymethyl)-4,8-dimethyltrideca-3,7,11-trien-1-yl)-2,8-dimethylchroman-6-yl)oxy)acetamide (**32**)

**32** was obtained from **69** with 72% yield using the method described above for **31** after purification by preparative TLC using a mixture of DCM/EtOAc/MeOH (70:30:7) as the mobile phase. *R_f_* = 0.30 (cyclohexane/ethyl acetate 6:4). ^1^H NMR (500 MHz, acetone-*d*_6_) δ_H_ 7.08 (bs, 1H), 6.68 (bs, 1H), 6.63 (d, *J* = 2.8 Hz, 1H), 6.53 (d, *J* = 2.8 Hz, 1H), 5.46 (t, *J* = 7.2 Hz, 1H), 5.18 (t, *J* = 7.2 Hz, 1H), 5.14 (t, *J* = 7.2 Hz, 1H), 4.31 (s, 2H), 4.17 (d, *J* = 5.5 Hz, 2H), 4.10 (d, *J* = 5.5 Hz, 2H), 3.65 (t, *J* = 5.2 Hz, 1H), 3.57 (t, *J* = 5.2 Hz, 1H), 2.74 (t, *J* = 6.8 Hz, 2H), 2.23–2.12 (m, 4H), 2.12 (s, 3H), 2.11–2.06 (m, 2H), 2.01–1.96 (m, 4H), 1.90–1.70 (m, 2H), 1.65–1.55 (m, 2H), 1.58 (s, 3H), 1.61 (s, 3H), 1.31 (s, 3H). ^13^C NMR (125 MHz, acetone-*d*_6_) δ_C_ 171.2, 151.5, 147.4, 140.2, 135.6, 135.3, 127.6, 127.5, 125.3, 125.2, 121.9, 116.4, 113.0, 76.1, 68.5, 65.9, 58.8, 40.5, 40.4, 40.1, 32.1, 27.2, 26.6, 24.4, 23.1, 22.8, 16.4, 16.1, 15.9. HRMS (ESI) *m*/*z* calcd for C_29_H_43_NNaO_5_ [M + Na]^+^ 508.3034, found 508.3032.

### Analysis of Pan Assay Interference Compounds (PAINS)

All investigated compounds were examined for known classes of assay interference compounds using the bulk pattern checker of ZINC15 (http://zinc15.docking.org/patterns/home/). Compounds **23**, **24**, and **35**–**40** were detected as Mannich bases. They effectively inhibited 5-LOX in a cell-free assay, with a few exceptions (**24**, **40**), but were considerably less active in suppressing 5-LOX product formation in PMNL and therefore were not further investigated.

### 5-LOX Activity

Human recombinant 5-LOX was expressed in *E. coli* Bl21 (DE3) cells that were transformed with pT3–5LOX (wt 5-LOX), 5-LOX_3W (triple mutation of Trp102Ala/Trp13Ala/Trp75Ala), or 5-LOX_R (single mutant of the Arg101Asp plasmid) and purified on an ATP-agarose column (Econo-Pac, Bio-Rad, Hercules, CA) by affinity chromatography.^6,75^ In a first step, *E. coli* was lysed in 50 mM triethanolamine/HCl pH 8.0 with EDTA (5 mM), soybean trypsin inhibitor (60 μg/mL), phenylmethylsulfonyl fluoride (1 mM), dithiothreitol (1 mM), and lysozyme (1 mg/mL) and sonified three times for 15 s. The homogenate was centrifuged at 10 000*g* for 15 min, followed by centrifugation of the supernatant at 40 000*g* for 70 min at 4 °C. The supernatant was transferred to an ATP-agarose column (Sigma-Aldrich, Deisenhofen, Germany), which was sequentially washed with PBS pH 7.4, 1 mM EDTA, 50 mM phosphate buffer pH 7.4, 0.5 M NaCl, 1 mM EDTA, and finally 50 mM phosphate buffer pH 7.4 plus 1 mM EDTA. The enzyme was eluted with 50 mM phosphate buffer pH 7.4, 50 mM EDTA, and 20 mM ATP.

Purified 5-LOX (0.5 μg in PBS pH 7.4 containing 1 mM EDTA and 1 mM ATP) was preincubated with the vehicle (DMSO) or test compounds for 10 min and prewarmed at 37 °C. 5-LOX product formation was started by addition of arachidonic acid (Sigma-Aldrich; 20 μM or as indicated) and CaCl_2_ (2 mM) and stopped by an equal volume of ice-cold methanol after 10 min at 37 °C. Major 5-LOX metabolites (all-trans isomers of LTB_4_ and 5-HETE) were extracted on Sep-Pak C18 35 cc Vac Cartridges (Waters, Milford, MA), separated by RP-HPLC on a Nova-Pak C18 Radial-Pak column (4 μm, 5 mm × 100 mm, Waters) under isocratic conditions (73% methanol/27% water/0.007% trifluoroacetic acid) at a flow rate of 1.2 mL/min, and detected at 235 and 280 nm.^75^ Zileuton was used as the reference 5-LOX inhibitor. To investigate whether **27a** reversibly inhibits 5-LOX, the purified enzyme was preincubated with **27a** at 0.3 μM for 15 min. The reaction mix was then 10-fold diluted to a final compound concentration of 0.03 μM, and arachidonic acid (20 μM) and CaCl_2_ (1 mM) were added after another 5 min incubation on ice. To recognize nuisance inhibition, Triton X-100 (Sigma-Aldrich; 0.01%) was added to the reaction buffer.

### Radical Scavenging Activity

Compound **27a** was incubated with 2,2-diphenyl-1-picrylhydrazyl (DPPH, Sigma-Aldrich; 50 mM) in ethanol for 30 min, and the absorbance was measured at 520 nm using a Multiskan Spectrum Microplate Reader (Thermo Fisher Scientific).^72^ The positive control ascorbic acid (Sigma-Aldrich; 25 μM) scavenged DPPH radicals by 36 ± 11%.

### Human Whole Blood and Blood Cells

Human PMNL, PBMC, monocytes, and platelets were freshly isolated from leukocyte concentrates that we obtained together with human whole blood (containing 3.13% sodium citrate) from the Institute for Transfusion Medicine of the University Hospital Jena (Germany).^76^ Venous blood was collected in heparinized tubes (16 U heparin/mL blood), with informed consent of registered male and female healthy adult volunteers (18–65 years) who were fasted for at least 12 h. These volunteers regularly donated blood (every 8–12 weeks) and were physically inspected by a clinician. They had not taken antibiotics or anti-inflammatory drugs for more than 10 days before blood donation and were free of apparent infections, inflammatory disorders, or acute allergic reactions.

Leukocytes were concentrated by centrifugation (4000*g*/20 min/20 °C) of freshly withdrawn blood and subjected to density gradient centrifugation on a lymphocyte separation medium (LSM 1077, GE Healthcare, Freiburg, Germany). Erythrocytes were removed by dextran sedimentation and hypotonic lysis. PMNL were obtained from the cell pellet and platelets from the supernatant. The PBMC fraction was plated in cell culture flasks (Greiner, Nuertingen, Germany) for 1.5 h at 37 °C and 5% CO_2_ in an RPMI 1640 medium (Sigma-Aldrich) with 5% FCS (Sigma-Aldrich), 2 mM l-glutamine (Sigma-Aldrich), and penicillin (100 U/mL)/streptomycin (100 μg/mL, GE Healthcare) (monocyte medium) to isolate adherent monocytes. The purity of monocytes (>85%) was determined from forward and side scatter properties, and CD14 surface expression was determined by flow cytometry (BD FACS Calibur, Heidelberg, Germany).

Monocytes were differentiated to macrophages by treatment with GM-CSF (20 ng/mL) for 6 days in an RPMI 1640 medium with 10% FCS, 2 mM l-glutamine, and penicillin (100 U/mL)/streptomycin (100 μg/mL). Macrophages were polarized to the M1 phenotype by stimulation with LPS (100 ng/mL, Sigma-Aldrich) and interferon (IFN)-γ (20 ng/mL, Peprotech, Hamburg, Germany) for 48 h.^23^

Experiments with blood and blood cells were approved by the ethics committee of the Friedrich-Schiller-University Jena.

### 5-LOX Product Formation by PMNL and Blood

Freshly isolated PMNL (5 × 10^6^) suspended in PBS pH 7.4 with 1 mg/mL glucose or freshly withdrawn human whole blood were preincubated with the test compounds for 10 min (or as indicated) at 37 °C. 5-LOX product formation in PMNL was triggered by addition of arachidonic acid (20 μM) and/or Ca^2+^-ionophore A23187 (2.5 μM; Sigma-Aldrich) followed by incubation for 10 min at 37 °C. The reaction was stopped with an equal volume of methanol.

Blood was treated with Ca^2+^-ionophore A23187 (30 μM) for 10 min at 37 °C or was first primed for 30 min with LPS (1 μg/mL) and then stimulated with fMLP (1 μM; Sigma-Aldrich) for 15 min. After the reaction was stopped on ice, plasma was prepared (600*g*, 10 min, 4 °C), and aliquots were mixed with an equal volume of methanol. Proteins were precipitated at −20 °C for 2 h and removed by centrifugation (600*g*, 15 min, 4 °C).

Major 5-LOX metabolites (all-trans isomers of LTB_4_ and 5-HETE) and, for PMNL, additionally 12-HETE (major 12-LOX product) and 15-HETE (major 15-LOX product) were extracted and analyzed by RP-HPLC as described for the determination of cell-free 5-LOX activity. Zileuton (3 μM) was used as the reference 5-LOX inhibitor.

### Fluorescence Spectroscopy of 5-LOX

Fluorescence spectroscopy measurements on human recombinant 5-LOX (Abcam, Cambridge, U.K.) were conducted in PBS pH 7.4 at 25 °C using an Edinburgh Instruments FLS920 Series fluorescence spectrophotometer (Livingston, U.K.). The effect of the ligand concentration was followed by means of tryptophan fluorescence, with excitation at 295 nm and emission collected between 310 and 450 nm, using excitation and emission slits of 5 and 10 nm, respectively. The concentration range of 5-LOX used through the experiments was 0.3–0.6 μM. 5-LOX was titrated with a stock solution of the test compounds in dimethylformamide to yield an experimental compound concentration in the range of 0–10 μM. The final dimethylformamide concentration was kept under 5%. After each addition, the sample was incubated for 10 min before measurement. The fluorescence emission intensity values were corrected for dilution and background noise. All conditions were measured independently and in duplicate.

The fluorescence emission spectral shift was used to monitor ligand–protein binding since significant variations of intensity were not observed. The fluorescence spectral shifts were analyzed by monitoring the alterations in the spectral center of mass by estimating the intensity-averaged emission wavelength (⟨λ⟩), as calculated from the following equation, where *F*_i_ is the fluorescence emission intensity at wavenumber ν_i_, and the summation was performed over the range of measured *F* values
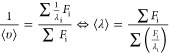
1

### Molecular Docking Simulations

The molecular docking simulation was conducted with GOLD 2020.2.0 (CCDC, Cambridge, U.K.).^77^ The built-in CHEMPLP scoring function was used to rescore the outputted poses (10 best-scored poses were kept for each compound). The stable crystallographic tridimensional structure of 5-LOX was downloaded from the Protein Data Bank (PDB entry 3O8Y).^78^ Four *in silico* mutations were inserted to return to the 5-LOX wild-type sequence: E13W, H14F, G75W, and S76L. The residues were exchanged, and the structure was energetically minimized in Discovery Studio 3.5 (Biovia, San Diego, CA).^79^ Hydrogen atoms were added with GOLD, using default settings. The binding site was constituted by 11 amino acid residues: Gln15, Arg101, Tyr81, Tyr100, Arg101, Trp102, Val110, Glu134, Asp170, Arg401, and Glu 622. Hydrogen-bond constraints were applied to Trp102-Hε1, Val110-HN, Glu134-Oε1, Asp170-Oδ1, Arg401-HH2, and Arg401-Hε with the constraint weight varying from 7 to 40 and the minimum H-bond geometry weight at 0.005. The ligands were allowed to detect internal H-bonds, flip pyramidal N, and flip amide bonds. Protein–ligand interactions of docking poses were analyzed using LigandScout 4.3 (Inteligand, Vienna, AT).^80^

### Intracellular Concentrations of **12a** and **27a**

PMNL (1 × 10^7^/mL) were suspended in PBS pH 7.4 with 1 mg/mL glucose and incubated with a vehicle (DMSO, 0.1%), **27a** or **12a** (150 nM, each) for 20 min at 37 °C. Cells were centrifuged (1200*g*, 5 min, 4 °C) and washed thrice with PBS pH 7.4 with 0.1% fatty-acid-free BSA (Sigma-Aldrich). Compounds were extracted and analyzed by UPLC-MS/MS as described below for LCMs and derivatives. The intracellular concentrations of **27a** and **12a** were calculated assuming a spherical cell shape with a diameter of 13 μm (as measured using a Vi-CELL Series Cell Counter, Beckman Coulter, Krefeld, Germany) and an equal intracellular distribution. **13d** (9 pmol) was used as the internal standard.

### Monocyte and PBMC Viability

Monocytes (4 × 10^5^/mL) or PBMC (2 × 10^6^/mL) in a monocyte medium were treated with the vehicle (0.1% DMSO) and test compounds for 2 or 24 h at 37 °C and 5% CO_2_. To measure mitochondrial dehydrogenase activity, 3-(4,5-dimethylthiazol-2-yl)-2,5-diphenyltetrazolium bromide (MTT) (20 μL, 5 mg/mL, Sigma-Aldrich) was added to PBMC. After 4 h in the darkness, the formed formazan product was solubilized (10% SDS in 20 mM HCl) and the absorbance was read at 570 nm using a Multiskan Spectrum microplate reader (Thermo Fisher Scientific).^81^ Staurosporine (3 μM; Merck Chemicals) was used as the cytotoxic reference compound.

LDH release was measured in monocytes using a CytoTox 96 nonradioactive cytotoxicity assay (Promega, Madison, WI) according to the manufacturer’s instruction using Triton X-100 (0.8%) as the maximum LDH release control.

### Intracellular Ca^2+^ Influx

PMNL (1 × 10^6^) were loaded with Fura-2/AM (2 nmol, Thermo Fisher Scientific) for 45 min at 37 °C. Washed cells in PBS pH 7.4 with 1 mg/mL glucose were preincubated with a vehicle (0.1% DMSO) or **27a** for 10 min prior to addition of 1 mM Ca^2+^ and stimulation with 1 μM fMLP for 10 min. Intracellular Ca^2+^ concentrations were measured using a NOVOstar fluorescence microplate reader (BMG Labtech, Ortenberg, Germany) at 37 °C. The emission wavelength was set to 510 nm and the excitation wavelengths to 340 nm (Ca^2+^-bound Fura-2) and 380 nm (free Fura-2).^23^ To define 100% Ca^2+^ influx, the difference between the maximal influx (cell lysis by Triton X-100) and minimal influx (subsequent chelation of Ca^2+^ by EDTA) was measured for each experiment.

### mPGES-1 Activity

Human lung carcinoma A549 cells (ATCC, Manassas, VA) were cultivated in DMEM (high glucose, 4.5 g/L; GE Healthcare) with 10% FCS and penicillin (100 U/mL)/streptomycin (100 μg/mL) at 37 °C and 5% CO_2_. Confluent cells were detached by trypsin-EDTA (GE Healthcare) and reseeded at a density of 1 × 10^4^ cells/cm^2^.

A549 cells were treated with IL-1β (2 ng/mL) at 37 °C and 5% CO_2_ for 48 h to induce mPGES-1 expression.^81^ Cell pellets of harvested cells were frozen in liquid nitrogen and then incubated on ice for 15 min and homogenized in ice-cold homogenization buffer (0.1 M potassium phosphate buffer pH 7.4, 1 mM phenylmethanesulfonyl fluoride (PMSF), 60 μg/mL soybean trypsin inhibitor (STI), 1 μg/mL leupeptin, 2.5 mM glutathione, and 250 mM sucrose) by sonication (3 × 20 s, 4 °C). Differential centrifugation at 10 000*g* for 10 min and 174 000*g* for 1 h at 4 °C yielded the microsomal fraction. The pellet was resuspended in homogenization buffer and diluted in 0.1 M potassium phosphate buffer pH 7.4 containing 2.5 mM glutathione. Microsomal membranes (adjusted to 2.5–5 μg total protein) were preincubated with **27a** for 15 min at 4 °C. The mPGES-1 substrate PGH_2_ (20 μM) was added to induce PGE_2_ formation. The reaction was stopped after 1 min by addition of an equal volume of aqueous 40 mM FeCl_2_, 80 mM citric acid, and 1 nmol 11β-PGE_2_ (Cayman Chemicals) as the internal standard. PGE_2_ was extracted on Sep-Pak C18 35 cc Vac Cartridges (Waters) and separated on a Nova-Pak C18 Radial-Pak Column (4 μm, 5 mm × 100 mm, Waters) under isocratic conditions (30% acetonitrile/70% H_2_O/0.007% trifluoroacetic acid) at a flow rate of 1 mL/min and detection at 195 nm.^82^ The mPGES-1 inhibitor MK-886 (10 μM) was used as the control.

### COX-1 and COX-2 Activity

Purified ovine COX-1 (Cayman Chemicals; 50 units) or human recombinant COX-2 (Cayman Chemicals; 20 units) was dissolved in 100 mM Tris buffer pH 8, 5 mM glutathione, 5 μM hemoglobin, and 100 μM EDTA. COX isoenzymes were preincubated with the vehicle (DMSO) or **27a** for 5 min at 4 °C and 1 min at 37 °C. Addition of arachidonic acid (COX-1: 5 μM; COX-2: 2 μM) initiated the formation of COX-derived 12(*S*)-hydroxy-5-*cis*-8,10-*trans*-heptadecatrienoic acid (12-HHT), which was extracted and analyzed by RP-HPLC according to 5-LOX products. The COX-1/2 inhibitor indomethacin (10 μM, Sigma-Aldrich) was used as the control.

### Platelet COX-1 Activity

Freshly isolated platelets (1 × 10^8^) were preincubated with vehicle (DMSO) or **27a** for 5 min at room temperature. Arachidonic acid (5 μM) was added to induce 12-HHT formation. After incubation for 5 min at 37 °C, 12-HHT was extracted and analyzed by RP-HPLC as described for 5-LOX products. The COX-1/2 inhibitor indomethacin (10 μM) was used as the reference compound.

### sEH Activity

Human recombinant sEH was expressed in Sf9 insect cells and purified by affinity chromatography.^83^ Briefly, Sf9 cells were infected with a recombinant baculovirus and lysed after 72 h in 50 mM NaHPO_4_ pH 8, 300 mM NaCl, 10% glycerol, 1 mM EDTA, 1 mM phenylmethanesulfonyl fluoride, 10 μg/mL leupeptin, and 60 μg/mL STI by sonication (3 × 10 s, 4 °C). Sequential centrifugation at 20 000*g* (10 min, 4 °C) and 100 000*g* (60 min, 4 °C) yielded a supernatant, which was subjected to benzylthio-sepharose affinity chromatography. Elution with 4-fluorochalcone oxide in PBS pH 7.4 with 1 mM dithiothreitol and 1 mM EDTA yielded sEH, which was dialyzed and concentrated.

Purified sEH (60 ng) in 25 mM Tris HCl pH 7 with 0.1 mg/mL bovine serum albumin (BSA) was preincubated with the vehicle (DMSO) or **27a** for 10 min at room temperature. The sEH substrate PHOME (50 μM, Cayman Chemicals) was added to start the enzymatic reaction, which was stopped after 60 min in darkness by addition of ZnSO_4_ (200 mM). The formation of the fluorescent product 6-methoxynaphtaldehyde was measured using a NOVOstar fluorescence microplate reader (BMG Labtech, Ortenberg, Germany), with excitation at 330 and emission at 465 nm. The selective sEH inhibitor AUDA (100 nM, Cayman Chemicals) was used as the control.

### Lipid Mediator Extraction for UPLC-MS/MS

Monocytes (1 × 10^6^/mL), which were prestimulated with LPS (1 μg/mL) for 24 h to induce COX-2 expression, or M1 macrophages (2 × 10^6^ cells/mL) were preincubated with the vehicle (0.1% DMSO), **27a**, or **12a** for 15 min. Arachidonic acid (20 μM) and A23187 (2.5 μM) were added to monocytes suspended in a monocyte medium (lacking FCS if indicated) to induce lipid mediator formation.^81^ Macrophages were stimulated in PBS pH 7.4 (containing 1 mM CaCl_2_) with *E. coli* (serotype O6:K2:H1) at a ratio of 1:50 (macrophages: *E. coli*) for 180 min at 37 °C and 5% CO_2_.^24^

Ice-cold methanol was added to monocytes, proteins were precipitated at −20 °C, and samples were acidified (pH 3.5).^81^ Lipid mediators were extracted by solid-phase extraction on Sep-Pak C18 35 cc Vac Cartridges (Waters). After washing with H_2_O twice, lipid mediators were eluted with methanol and analyzed by UPLC-MS/MS.^81^ PGB_1_ (100 ng, Cayman Chemicals) was used as the internal standard.

Fatty acids and lipid mediators were extracted from macrophage supernatants or murine peritoneal exudates (1 mL) after addition of two aliquots of ice-cold methanol.^23^*d*_8_-5*S*-HETE, *d*_4_-LTB_4_, *d*_5_-lipoxin A_4_, *d*_5_-resolvin D_2_, *d*_4_-PGE_2_ (200 nM, each, Cayman Chemicals), and *d*_8_-arachidonic acid (10 μM, Cayman Chemicals) were added as internal standards. After protein precipitation at −20 °C for ≥1 h, supernatants (1200*g*, 10 min, 4 °C) were acidified (pH 3.5) and loaded onto solid-phase cartridges (Sep-Pak Vac 6cc 500 mg/6 mL C18; Waters), which had been equilibrated with methanol and H_2_O. The columns were extensively washed with H_2_O and hexane before lipid mediators were eluted with methyl formate, evaporated to dryness, dissolved in methanol/H_2_O (50:50), and analyzed by UPLC-MS/MS.^23^

### Extraction of LCMs and Derivatives

LCMs, **27a**, and **27a** metabolites were extracted from PMNL, medium of the liver-on-chip, murine plasma, peritoneal exudate, or homogenized murine tissue as described.^6^ In brief, PBS pH 7.4, methanol, chloroform, and saline (final ratio: 14:34:35:17) were successively added together with **13d** (9 pmol) as the internal standard. The organic layer was evaporated, and the extracted LCMs were dissolved in methanol.

### Liquid Chromatography and Mass Spectrometry

Lipid mediators, LCMs, **27a**, and **27a** metabolites were separated on an Acquity UPLC BEH C18 column (1.7 μm, lipid mediators for monocytes and LCMs: 2.1 mm × 50 mm; otherwise, 2.1 mm × 100 mm, Waters) using an Acquity UPLC system (Waters), which was coupled to a QTRAP 5500 mass spectrometer (SCIEX, Framingham, MA) equipped with an electrospray ionization source.^6,23,81^ Diagnostic ion fragments were detected by (scheduled) multiple reaction monitoring (MRM) in the negative ion mode.

To analyze lipid mediators produced by monocytes, a flow rate of 0.8 mL/min and a column temperature of 45 °C were applied. Mobile phase A consisted of acetonitrile with 0.07% formic acid, and mobile phase B was acetonitrile/H_2_O (10:90) with 0.07% formic acid. Isocratic elution at A/B (30:70) for 2 min was followed by a linear gradient to A/B:70/30 within 5 min. Data were normalized to the internal standard PGB_1_, which allows the comparison of signal intensities between samples but not absolute quantification.

To quantify lipid mediators and fatty acids in macrophages, murine plasma, and murine peritoneal exudates, the mobile phase methanol/H_2_O (acidified with 0.01% acetic acid) was ramped from 42:58 to 86:14 over 12.5 min, followed by isocratic elution with methanol/H_2_O (98:2) for 3 min. Absolute quantification was based on 6 internal and 45 external standards.^23^

LCMs, **27a**, and **27a** metabolites were separated at a flow rate of 0.8 mL/min and a column temperature of 45 °C, with acetonitrile/H_2_O (10:90) as mobile phase A and acetonitrile as mobile phase B, both acidified with 0.07% formic acid. The linear gradient from A/B = 50:50 to 0:100 within 4.5 min was followed by isocratic elution with A/B = 0:100 for 1 min. MS parameters were adjusted according to Pein et al.^6^ and as detailed in Table S3. Signal intensities were normalized to the internal standard **13d**, and concentrations were calculated using external calibration curves as specified in Table S3.

Automatic peak integration was performed with Analyst 1.6 software (Sciex) using IntelliQuan default settings.

### Correlation Network Analysis

The structure-based correlation networks of LCMs and derivatives were generated with Cytoscape 3.7.2 software (Cytoscape Consortium).^84^ Tanimoto coefficients were calculated between the SMILES structures of compounds and represent the connecting edges in the correlation network, which is presented in an edge-weighted spring embedded layout. Nodes in close proximity represent structurally related compounds. The potency of the compounds to inhibit cell-free or cell-based 5-LOX activity is visualized by the node size, where bigger nodes represent compounds with higher inhibitory activity (lower IC_50_ value). The node shape illustrates the compound series, and the node color indicates the series of garcinoic acids (**13a**-**e**), amplexichromanols (**27a**–**d**), tocopherols, and tocotrienols (**1a**–**d**, **6a**–**d**).

### Multiorgan-Tissue-Flow (MOTiF) Biochips

Biochips were made from polystyrene by injection molding and were equipped with a poly(ethylene terephthalate) (PET) membrane (TRAKETCH, thickness: 12 μm; pore diameter: 8 μm; pore density: 1 × 10^5^ pores/cm^2^; Sabeu, Radeberg, Germany) that was integrated in the upper and lower parts of the biochip by heat-sealing with the bulk material. The top and bottom sides of the biochips and channels were sealed with an extruded PS bonding foil (thickness: 125 μm) by a low-temperature bonding method. The upper and lower parts of the biochips were assembled using a double-sided adhesive film, and the chip surface was hydrophilized by oxygen plasma treatment to facilitate cell adhesion and prevent air bubble formation within the chambers and channels.^85,86^

HUVECs were isolated from human umbilical cord veins and cultivated in an endothelial cell growth medium MV (Promocell, Heidelberg, Germany) with penicillin (100 U/mL)/streptomycin (100 μg/mL, GE Healthcare) up to passage 4.^87^ On reaching 95% confluence, cells were subcultured at a density of 1.5 × 10^4^ cells/cm^2^.

HepaRG cells (Biopredic International, Rennes, France) were grown in William’s medium E (Biochrom, Berlin, Germany) with 10% FCS, 5 μg/mL insulin (Sigma-Aldrich), 2 mM glutamine (Thermo Fisher Scientific), 50 μM hydrocortisone-hemisuccinate (Sigma-Aldrich), and penicillin (100 U/mL)/streptomycin (100 μg/mL, GE Healthcare) at 37 °C and 5% CO_2_ for 14 days prior to differentiation with 2% DMSO for another 14 days. The medium was changed every 3–4 days, and differentiated cells were used up to 4 weeks.

The liver-on-chip was prepared by seeding HUVECs (top: 3 × 10^5^, bottom: 1.5 × 10^5^) in an endothelial cell growth medium MV on top of the membrane in the upper chamber and on the bottom of the membrane in the lower chamber, giving the cells 3–4 h to adhere before flipping the chip upside and seeding the bottom layer. After 2 days, differentiated HepaRG cells (3 × 10^5^) were seeded on top of the lower and on the bottom of the upper membrane in a hepatocyte culture medium with hydrocortisone-hemisuccinate adjusted to 5 μM for 24 h at 37 °C and 5% CO_2_. The medium between the two membranes was renewed, and the vehicle (DMSO), **12a**, or **27a** were added. After incubation for 48 h at 37 °C and 5% CO_2,_ the medium was collected and the system was washed with 500 μL of methanol. **12a**, **27a**, and their metabolites were extracted from the combined fractions and analyzed by UPLC-MS/MS.^6^

### CYP3A4 Expression in Hepatocytes

HepaRG cells were seeded at a density of 2.6 × 10^4^ cells/cm^2^ in Williams’ E medium supplemented with 2 mM GlutaMAX (Thermo Fisher Scientific, Waltham, MA), 100 U/mL penicillin, 100 μg/mL streptomycin, 10% HyClone FCS (Thermo Fisher Scientific), 5 μg/mL insulin, and 50 mM hydrocortisone-hemisuccinate. At confluence after two weeks, HepaRG cells were shifted to a medium supplemented with 1.7% DMSO for two additional weeks to obtain confluent differentiated cultures containing equal proportions of hepatocyte-like and progenitors/primitive biliary-like cells. These differentiated hepatic cell cultures were exposed to the compounds in Williams’ E medium supplemented with 2% FCS plus 0.2% DMSO for 48 h.

For immunolocalization of cytochrome CYP3A4 and F-actin, cells were fixed at 4 °C with 4% paraformaldehyde for 20 min and permeabilized for 30 min with 0.1% saponin in PBS pH 7.4 containing 5% donkey serum. Cells were washed with PBS and incubated for 90 min with the CYP3A4 primary antibody (AB1254, Chemicon International, Temecula, CA) diluted at 1/800 in PBS containing 0.1% saponin and, then, incubated for 45 min with an FITC-labeled secondary antibody (Jackson ImmunoResearch, West Grove, PA) diluted at 1/400 in 0.1% saponin. F-actin was labeled by the phalloidin fluoroprobe SR101 (200 U/mL) (Interchim, Montluçon, France) diluted at 1/100 for 20 min.

The assay plate was imaged and analyzed using the Cell Health Profiling BioApplications (HCS Studio Cellomics software Version x.6.4.3 (Build 7207)) on the ArrayScan VTI HCS Reader (Thermo Fisher Scientific—Cellomics, Pittsburgh, PA) using the 20× objective (Zeiss, LD Plan Neofluar 20×/0.4 korr). Images were acquired with the Photometrics X1 CCD camera with a 14-bit dynamic range, 2208 × 2208 pixel array, and 4.54 μm pixel size.

The CYP3A4 expression was quantified using an arbitrary unit of fluorescent mass representing the combination of intensity and area adjusted to cell number calculated by nuclei counting (Hoechst).

### Experimental Atopic Dermatitis

Compound **27a** was evaluated in an inflammatory model of RHE by Synelvia S.A.S. (Labège, France) according to the company’s protocol.^88^ In brief, primary female human keratinocytes were used to generate RHE on polycarbonate filters as described.^88^ RHE was allowed to acclimate for 6 days (5% CO_2_, 37 °C) prior to incubation with **27a** (5, 10, 25 μM), dexamethasone (1 μM), or vehicle (0.05% DMSO), and a proinflammatory cytokine cocktail for 4 days at 5% CO_2_ and 37 °C. The culture medium including compounds and cytokines was changed on days 6, 8, and 10. Each culture condition was repeated three times. Acute cytotoxicity of **27a** was excluded at the concentrations studied within 24 h by measuring the mitochondrial dehydrogenase activity (MTT assay).

Morphological changes (*i.e*., spongiosis and the formation of keratohyalin granules) were evaluated for sections of paraffin-embedded RHEs after hematoxylin-eosin staining.^89^ The permeability of the skin barrier was estimated from the transepidermal water loss at the RHE surface.^90^ The surface pH was determined with a pH electrode as reported.^89^ The permeability of the stratum corneum was addressed by applying Lucifer yellow onto the RHE surface and inspecting the penetration by fluorescence microscopy.^88^ TSLP and IL-8 levels were analyzed by ELISA (according to the specifications of Synelvia S.A.S.) in the culture medium on day 2 and day 4 after addition of cytokines, respectively.

### Animals

Male CD-1 mice (33–39 g, 6–8 weeks, Charles River Laboratories; Calco, Italy) and female BALB/c mice (20 g, 8 weeks, Charles River Laboratories) were fed with standard rodent chow and water and acclimated for 4 days at a 12 h light and 12 h dark schedule in a constant air-conditioned environment (21 ± 2 °C). Mice were randomly assigned to groups, and experiments were carried out during the light phase. Experimental procedures were conducted in conformity with Italian (DL 26/2014) and European (directive 2010/63/EU) regulations on the protection of animals used for scientific purposes and approved by the Italian Ministry.

### Zymosan-Induced Peritonitis in Mice

CD-1 mice received **27a** or zileuton, with DMSO (2%) in saline (0.5 mL) as a vehicle for i.p. administration and carboxymethylcellulose (0.5%) in 10% Tween 20 (0.5 mL) as a vehicle for p.o. administration. Zymosan (2 mg/mL in saline, i.p., 0.5 mL, Sigma-Aldrich) was injected at 30 min (i.p.) or 60 min (p.o.) post compound administration. Mice were sacrificed by inhalation of CO_2_ after another 30 min to determine LTC_4_ levels, lipid mediator profiles, metabolites, and vascular permeability and after 4 h to analyze LTB_4_ levels and cell infiltration.^72^ Plasma and peritoneal exudates were collected, and cells were counted in exudates after trypan blue staining. Vascular permeability was measured by injection of Evans blue dye (40 mg/kg in saline, 0.3 mL, Sigma-Aldrich) into the tail vain directly before peritonitis induction.^72^ The exudate was centrifuged (3000*g*, 5 min), and the absorbance was measured at 610 nm (DU730 spectrophotometer, Beckman Coulter, Krefeld, Germany). Levels of LTB_4_ and cysteinyl-LTs (dominated by LTC_4_) were quantified in the exudate by ELISA (Enzo Life Sciences, Lörrach, Germany) according to the manufacturer’s instructions. Compound **27a**, its metabolites, and lipid mediators were extracted and analyzed by UPLC-MS/MS.

### Experimental Model of Murine Asthma

BALB/c mice received **27a** (p.o.) or vehicle (0.5% carboxymethylcellulose in 10% Tween 20; 0.5 mL) on days 0 and 7, 1 h (p.o) before being sensitized to ovalbumin (100 μg adsorbed to 3.3 mg of aluminum hydroxide gel, s.c., Sigma-Aldrich). Mice were sacrificed on days 1, 3, 8, 10, or 21 to collect lung and plasma. Bronchi were cut in rings of 1 to 2 mm length, placed in organ baths, and fixed to an isometric force transducer 7006 connected to a Powerlab 800 (AD Instruments, Ugo Basile, Comerio, Italy). After stretching the rings to a resting tension of 0.5*g* and equilibration for at least 30 min, the rings were challenged with carbachol (1 μM) until a reproducible response was observed. To assess bronchial reactivity, the cumulative response to carbachol (0.001 to 3.16 μM) was measured. Bronchial relaxation was determined from the cumulative response of precontracted bronchial tissues to salbutamol. Compound **27a**, LTB_4_, and LTB_4_ isomers were analyzed in plasma and lung homogenates by UPLC-MS/MS. Lung tissue (100 mg/mL) was homogenized in PBS pH 7.4 at 4 °C for 1–2 min using an Omni tissue homogenizer (Omni, Kennesaw, GA).

### Statistics

Data are presented as mean with single values or mean ± SEM of *n* observations, where *n* represents the number of experiments or the number of animals, as indicated. The compound library was blinded for screening purposes but not for further biological evaluation, and samples from mouse peritoneum were blinded for counting infiltrated cells. Outliers were identified using a Grubb’s test. Different groups were compared by one-way or two-way ANOVA for independent or correlated samples followed by Tukey’s or Bonferroni’s HSD *post hoc* test or by the two-tailed Student’s *t*-test for paired or unpaired samples. Tests were conducted using a two-sided α level of 0.05. *P* values <0.05 were considered statistically significant. Statistical calculations were performed using GraphPad Prism 9.1 (GraphPad Software, La Jolla, CA). IC_50_ values were determined by graphical analysis using SigmaPlot 14.0 (Systat Software Inc., San Jose, CA).

## Supporting Information Available

Molecular formula strings of all compounds (210705_SMILES (CSV))
Inhibition of human isolated 5-LOX and 5-LOX product formation in activated PMNL by natural vitamin E forms and derivatives (**1a**–**11**) (Table S1); nomenclature proposed to name natural vitamin E forms and ω-oxidized derivatives (Table S2); conditions for the quantification of **27a** and its metabolites by UPLC-MS/MS (Table S3); SARs on cell-free 5-LOX inhibition^a^ (Scheme S1); SARs on the inhibition of 5-LOX product formation in PMNL^a^ (Scheme S2); correlation network of the compound library for the inhibition of cell-free 5-LOX (Figure S1); molecular docking simulation of 5-LOX (Figure S2); fluorescence spectroscopic analysis of 5-LOX ligand interactions (Figure S3); effect of **27a** on human monocyte and PBMC viability (Figure S4); compound **27a** selectively inhibits 5-LOX product formation in activated monocytes (Figure S5); compound **27a** attenuates cytokine-triggered defects in reconstructed human epidermis (RHE) (Figure S6); effect of **27a** on resolvin (Rv)E3 and systemic LTB_4_ levels in mice with acute peritonitis (Figure S7); ^1^H and ^13^C NMR spectra of **49**, **50**, **13e**, **51**, **2**, **4**, **53**, **19b**, **19a**, **20**, **21**, **22**, **55**, **15b**, **15a**, **25**, **58**, **14**, **59**, **16**, **60, 17**, **61**, **18**, **65**, **66**, **26**, **41**, **40**, **35**, **36**, **37**, **38**, **39**, **42**, **43**, **67**, **31**, **70**, **48**, **46**, **68**, **31**, **69**, **32**, **62**, **28, 63**, and ****29****; and HPLC-ELSD spectra of **13a**, **13d**, **27a**, and **27d** (Figures S8−S60) (PDF)

## Abbreviations Used

COX: cyclooxygenase
cPLA_2_: cytosolic phospholipase A_2_
DPPH: 2,2-diphenyl-1-picrylhydrazyl
FLAP: 5-lipoxygenase-activating protein
fMLP: *N*-formyl-methionyl-leucyl-phenylalanine
H(P)ETE: hydro(pero)xyeicosatetraenoic acid
HDHA: hydroxydocosahexaenoic acid
HEPE: hydroxyeicosapentaenoic acid
HETE: hydroxyeicosatetraenoic acid
HUVEC: human umbilical vein endothelial cells
IL: interleukin
iNOS: inducible nitric oxide synthase
LCM: long-chain vitamin E metabolite
LDH: lactate dehydrogenase
LOX: lipoxygenase
LPS: lipopolysaccharide
LT: leukotriene
mPGES-1: microsomal prostaglandin E_2_ synthase-1
MTT: 3-(4,5-dimethylthiazol-2-yl)-2,5-diphenyltetrazolium bromide
NAFLD: nonalcoholic fatty liver disease
PBMC: peripheral blood mononuclear cells
PG: prostaglandin
PMNL: polymorphonuclear leukocytes
RHE: reconstructed human epidermis
sEH: soluble expoxide hydrolase
TSLP: thymic stromal lymphopoietin
