# Supplementary material for: Exploration of Long-Chain Vitamin E Metabolites for the Discovery of a Highly Potent, Orally Effective, and Metabolically Stable 5-LOX Inhibitor that Limits Inflammation
Source: J Med Chem. 2021 Jul 19;64(15):11496–526. doi: 10.1021/acs.jmedchem.1c00806 (PMC8365602; doi:10.1021/acs.jmedchem.1c00806)
Supplement: Supplementary file 1 — jm1c00806_si_001.pdf [file jm1c00806_si_001.pdf]

SUPPORTING INFORMATION

Exploration of long-chain vitamin E metabolites for the discovery of a highly potent, orally effective and metabolically stable 5-LOX inhibitor that limits inflammation

Konstantin Neukirch[1,2 ‡], Khaled Alsabil[3 ‡], Chau-Phi Dinh[3 †], Rossella Bilancia[4], Martin Raasch[5], Alexia Ville[3 †], Ida Cerqua[4], Guillaume Viault[3], Dimitri Bréard[3], Simona Pace[2], Veronika Temml[6], Elena Brunner[2], Paul M. Jordan[2], Marta C. Marques[7], Konstantin Loeser[2], André Gollowitzer[1,2], Stephan Permann[1], Jana Gerstmeier[2], Stefan Lorkowski[8], Hermann Stuppner[9], Ulrike Garscha[10], Tiago Rodrigues[7], Gonçalo J. L. Bernardes[7,11], Daniela Schuster[6], Denis Séraphin[3], Pascal Richomme[3], Antonietta Rossi[4], Alexander S. Mosig[5], Fiorentina Roviezzo[4], Oliver Werz[2 *], Jean-Jacques Helesbeux[3 *], Andreas Koeberle[1,2 *]

[1]Michael Popp Institute and Center for Molecular Biosciences Innsbruck (CMBI), University of Innsbruck, 6020 Innsbruck, Austria

[2]Department of Pharmaceutical/Medicinal Chemistry, Institute of Pharmacy, Friedrich Schiller University Jena, 07743 Jena, Germany

[3]Univ Angers, SONAS, SFR QUASAV, F-49000 Angers, France

[4]Department of Pharmacy, School of Medicine and Surgery, University of Naples Federico II, 80131 Naples, Italy

[5]Institute of Biochemistry II, Jena University Hospital, 07747 Jena, Germany

[6]Department of Pharmaceutical and Medicinal Chemistry, Paracelsus Medical University Salzburg, 5020 Salzburg, Austria

[7]Instituto de Medicina Molecular João Lobo Antunes, Faculdade de Medicina, Universidade de Lisboa, 1649-028 Lisboa, Portugal

[8]Department of Nutritional Biochemistry and Physiology, Institute of Nutritional Science and Competence Cluster for Nutrition and Cardiovascular Health (nutriCARD), Halle-Jena-Leipzig, Friedrich Schiller University Jena, 07743 Jena, Germany

[9]Institute of Pharmacy/Pharmacognosy and Center for Molecular Biosciences Innsbruck (CMBI), University of Innsbruck, 6020 Innsbruck, Austria

[10]Department of Pharmaceutical/Medicinal Chemistry, Institute of Pharmacy, University of Greifswald, 17489 Greifswald, Germany

[11]Department of Chemistry, University of Cambridge, CB2 1EW, Cambridge, UK

* Andreas Koeberle – Michael Popp Institute and Center for Molecular Biosciences Innsbruck (CMBI), University of Innsbruck, A-6020 Innsbruck, Austria; orcid.org/0000-0001-6269-5088; Tel.: +43 512 507-57903; E-mail: andreas.koeberle@uibk.ac.at

Jean-Jacques Helesbeux – SONAS, EA921, UNIV Angers, SFR QUASAV, Faculty of Health Sciences, Department of Pharmacy, 49045 Angers Cedex 01, France; orcid.org/0000-0002-1894-8911; Tel.: +33 249 180 441; E-mail: jean-jacques.helesbeux@univ-angers.fr

Oliver Werz – Department of Pharmaceutical/Medicinal Chemistry, Institute of Pharmacy, Friedrich-Schiller-University Jena, 07743 Jena, Germany; orcid.org/0000-0002-5064-4379; Tel.: +49 3641 9-49801; Fax: +49 3641 9-49802; E-mail: oliver.werz@uni-jena.de

SUPPORTING INFORMATION

Table of Contents

SI TABLES S3

Table S1. Inhibition of human isolated 5-LOX and 5-LOX product formation in activated PMNL by natural vitamin E forms and derivatives (**1a-11**) S3

Table S2. Nomenclature proposed to name natural vitamin E forms and ω-oxidized derivatives S6

Table S3. Conditions for the quantification of **27a** and its metabolites by UPLC-MS/MS S7

SI SCHEMES S8

Scheme S1. SARs on cell-free 5-LOX inhibition[a] S8

Scheme S2. SARs on the inhibition of 5-LOX product formation in PMNL[a] S9

SI FIGURES S10

Figure S1. Correlation network of the compound library for inhibition of cell-free 5-LOX. S10

Figure S2. Molecular docking simulation of 5-LOX. S11

Figure S3. Fluorescence spectroscopic analysis of 5-LOX ligand interactions S12

Figure S4. Effect of **27a** on human monocyte and PBMC viability. S13

Figure S5. Compound **27a** selectively inhibits 5-LOX product formation in activated monocytes. S14

Figure S6. Compound **27a** attenuates cytokine-triggered defects in reconstructed human epidermis (RHE) S15

Figure S7. Effect of **27a** on resolvin (Rv)E3 and systemic LTB_4 levels in mice with acute peritonitis. S16

Figure S8. ^{1}H and ^{13}C NMR spectra of **49** in acetone-d_6 .... S17

Figure S9. ^{1}H and ^{13}C NMR spectra of **50** in $CDCl_3$ S18

Figure S10. ^{1}H and ^{13}C NMR spectra of **13e** in $CDCl_3$ S19

Figure S11. ^{1}H and ^{13}C NMR spectra of **51** in $CDCl_3$ S20

Figure S12. ^{1}H and ^{13}C NMR spectra of **2** in acetone-d_6 .... S21

Figure S13. ^{1}H and ^{13}C NMR spectra of **4** in acetone-d_6 .... S22

Figure S14. ^{1}H and ^{13}C NMR spectra of **53** in $CDCl_3$ S23

Figure S15. ^{1}H and ^{13}C NMR spectra of **19b** in $CDCl_3$ S24

Figure S16. ^{1}H and ^{13}C NMR spectra of **19a** in $CDCl_3$ S25

Figure S17. ^{1}H and ^{13}C NMR spectra of **20** in $CDCl_3$ S26

Figure S18. ^{1}H and ^{13}C NMR spectra of **21** in $CDCl_3$ S27

Figure S19. ^{1}H and ^{13}C NMR spectra of **22** in $CDCl_3$ S28

Figure S20: ^{1}H and ^{13}C NMR spectra of **55** in $CDCl_3$ S29

Figure S21. ^{1}H and ^{13}C NMR spectra of **15b** in $CDCl_3$ S30

Figure S22. ^{1}H and ^{13}C NMR spectra of **15a** in $CDCl_3$ S31

Figure S23. ^{1}H and ^{13}C NMR spectra of **25** in $CDCl_3$ S32

Figure S24. ^{1}H and ^{13}C NMR spectra of **58** in $CDCl_3$ S33

Figure S25. ^{1}H and ^{13}C NMR spectra of **14** in $CDCl_3$ S34

Figure S26. ^{1}H and ^{13}C NMR spectra of **59** in $CDCl_3$ S35

Figure S27. ^{1}H and ^{13}C NMR spectra of **16** in $CDCl_3$ S36

Figure S28. ^{1}H and ^{13}C NMR spectra of **60** in $CDCl_3$ S37

Figure S29. ^{1}H and ^{13}C NMR spectra of **17** in $CDCl_3$ S38

Figure S30. ^{1}H and ^{13}C NMR spectra of **61** in $CDCl_3$ S39

Figure S31. ^{1}H and ^{13}C NMR spectra of **18** in $CDCl_3$ S40

Figure S32. ^{1}H and ^{13}C NMR spectra of **65** in $CDCl_3$ S41

Figure S33. ^{1}H and ^{13}C NMR spectra of **66** in $CDCl_3$ S42

Figure S34. ^{1}H and ^{13}C NMR spectra of **26** in $CDCl_3$ S43

Figure S35. ^{1}H and ^{13}C NMR spectra of **41** in acetone-d_6 .... S44

Figure S36. ^{1}H and ^{13}C NMR spectra of **40** in methanol-d_4 .... S45

Figure S37. ^{1}H and ^{13}C NMR spectra of **35** in acetone-d_6 .... S46

Figure S38. ^{1}H and ^{13}C NMR spectra of **36** in acetone-d_6 .... S47

Figure S39. ^{1}H and ^{13}C NMR spectra of **37** in acetone-d_6 .... S48

Figure S40. ^{1}H and ^{13}C NMR spectra of **38** in acetone-d_6 .... S49

Figure S41. ^{1}H and ^{13}C NMR spectra of **39** in acetone-d_6 .... S50

Figure S42. ^{1}H and ^{13}C NMR spectra of **42** in acetone-d_6 .... S51

Figure S43. ^{1}H and ^{13}C NMR spectra of **43** in acetone-d_6 .... S52

Figure S44. ^{1}H and ^{13}C NMR spectra of **67** in acetone-d_6 .... S53

Figure S45. ^{1}H and ^{13}C NMR spectra of **31** in acetone-d_6 .... S54

Figure S46. ^{1}H and ^{13}C NMR spectra of **70** in acetone-d_6 .... S55

Figure S47. ^{1}H and ^{13}C NMR spectra of **48** in acetone-d_6 .... S56

Figure S48. ^{1}H and ^{13}C NMR spectra of **46** in acetone-d_6 .... S57

Figure S49. ^{1}H and ^{13}C NMR spectra of **68** in acetone-d_6 .... S58

Figure S50. ^{1}H and ^{13}C NMR spectra of **31** in acetone-d_6 .... S59

Figure S51. ^{1}H and ^{13}C NMR spectra of **69** in acetone-d_6 .... S60

Figure S52. ^{1}H and ^{13}C NMR spectra of **32** in acetone-d_6 .... S61

Figure S53. ^{1}H and ^{13}C NMR spectra of **62** in $CDCl_3$ S62

Figure S54. ^{1}H and ^{13}C NMR spectra of **28** in $CDCl_3$ S63

Figure S55. ^{1}H and ^{13}C NMR spectra of **63** in $CDCl_3$ S64

Figure S56. ^{1}H and ^{13}C NMR spectra of **29** in $CDCl_3$ S65

Figure S57. HPLC-ELSD spectrum of **13a** S66

Figure S58. HPLC-ELSD spectrum of **13d** S66

Figure S59. HPLC-ELSD spectrum of **27a** S67

Figure S60. HPLC-ELSD spectrum of **27d** S67

SI TABLES

Table S1. Inhibition of human isolated 5-LOX and 5-LOX product formation in activated PMNL by natural vitamin E forms and derivatives (1a-11)

Compound	Structure 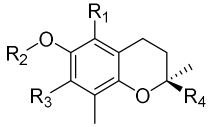				5-LOX enzyme		5-LOX PMNL	
	**R1**	**R2**	**R3**	**R4**	**IC_{50} [µM][a]**	**at 1 µM [%][b]**	**IC_{50} [µM][a]**	**at 3 µM [%][c]**
**1a**	CH_3	H	CH_3	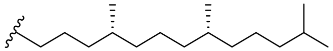	> 1[d]	86.1 ± 8.3	> 3[d]	88.6 ± 10.5
**1b**	CH_3	H	H	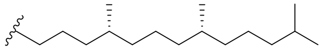	0.75 ± 0.15[d]	32.8 ± 9.0	> 3[d]	79.6 ± 6.1
**1c**	H	H	CH_3	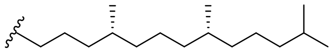	0.91 ± 0.15[d]	47.0 ± 4.5	> 3[d]	78.5 ± 13.6
**1d**	H	H	H	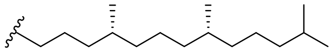	0.60 ± 0.25	43.3 ± 7.2	> 3[d]	107.4 ± 3.2
**2**	Cl	H	CHO	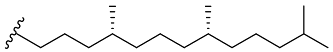	> 1	72.9 ± 5.6	> 3	109.8 ± 10.7
**3**	CHO	H	H	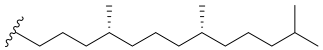	> 1	94.6 ± 3.5	> 3	92.2 ± 6.3
**4**	CHO	CH_2OCH_3	H	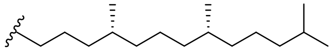	> 1	77.3 ± 17.2	> 3	91.9 ± 5.3
**5**	CO_2H	H	H	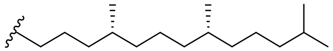	> 1	54.6 ± 5.8	> 3	80.8 ± 0.6
**6a**	CH_3	H	CH_3	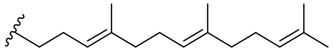	0.33 ± 0.08[d]	16.9 ± 6.6	> 3[d]	75.5 ± 4.5

**6b**	CH_3	H	H	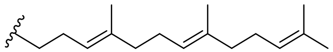	0.19 ± 0.03[d]	6.4 ± 3.2	2.11 ± 0.36[d]	41.4 ± 8.0
**6c**	H	H	CH_3	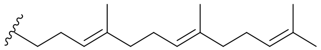	0.20 ± 0.06[d]	6.4 ± 3.2	> 3[d]	73.5 ± 1.2
**6d**	H	H	H	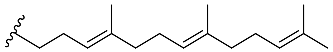	0.17 ± 0.10[d]	6.7 ± 3.1	> 3[d]	74.9 ± 8.0
**7**	CHO	H	H	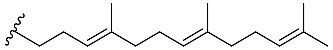	> 1	74.5 ± 4.3	> 3	87.1 ± 8.0
**8**	CHO	H	Br	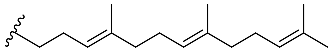	> 1	59.0 ± 5.9	> 3	79.2 ± 16.7
**9a**	CH_3	H	CH_3	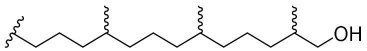	0.35 ± 0.04[d]	1.5 ± 0.4	0.19 ± 0.05[d]	21.6 ± 2.5
**9b**	H	H	H	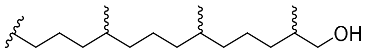	0.12 ± 0.04[d]	2.1 ± 0.3	0.54 ± 0.18[d]	22.6 ± 2.4
**10a**	CH_3	H	CH_3	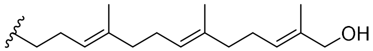	0.11 ± 0.01[d]	0.2 ± 0.1	0.27 ± 0.10[d]	13.2 ± 2.0
**10b**	CH_3	H	H	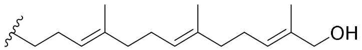	0.09 ± 0.03[d]	0.2 ± 0.1	0.38 ± 0.09[d]	7.7 ± 1.3
**10c**	H	H	H	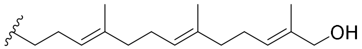	0.15 ± 0.05[d]	2.7 ± 1.5	1.26 ± 0.33[d]	8.8 ± 1.9
**10d**	H	H	CH_3	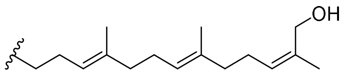	0.12 ± 0.03[d]	0.0 ± 0.0	0.14 ± 0.02[d]	1.1 ± 1.1
**10e**	H	H	H	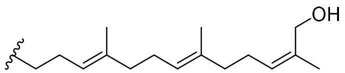	0.14 ± 0.03[d]	0.3 ± 0.2	0.22 ± 0.04[d]	2.0 ± 0.2
**11**	H	H	H	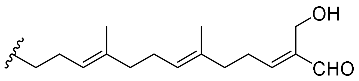	0.12 ± 0.05	0.4 ± 0.3	0.57 ± 0.09	17.6 ± 1.3[b]

**zil** (zileuton)	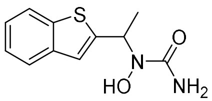	0.69 ± 0.24	34.6 ± 14.1	3.57 ± 0.55	55.6 ± 5.6

[a]IC$_{50}$ values (μM) and residual activities (% control) at [b]1 or [c]3 μM compound concentration given as mean ± SEM of single determinations obtained in 3 to 4 independent experiments. [d]Highlighted data (grey) originates from Pein et al.[4].

Table S2. Nomenclature proposed to name natural vitamin E forms and ω-oxidized derivatives

		T - tocopherol	TE - tocotrienol
**Scaffold**		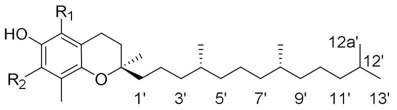	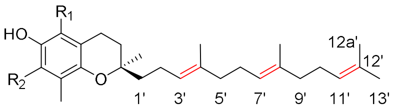
		**R_1**	**R_2**
**Chromanol substituents**	α-	CH_3	CH_3
	β-	CH_3	H
	γ-	H	CH_3
	δ-	H	H
**Exemplary side chain modifications**[a]		α-T-13‘-CH_2OH (**9a**) 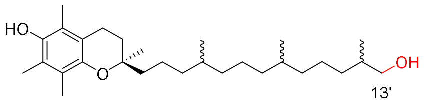	δ-TE-13‘-COOH (**13d**) 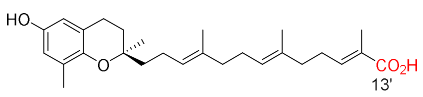
		α-T-13‘-COOH (**12a**) 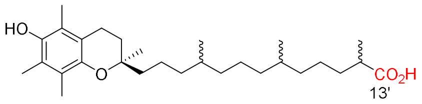	α-TE-12a‘,13‘-diCH_2OH (**27a**) 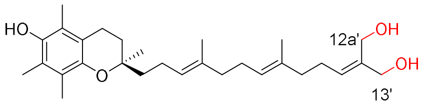
		α-T-11‘-COOH 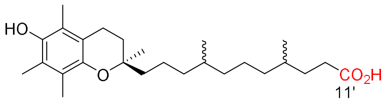	α-TE-12a‘/13‘-CH_2OH/COOH 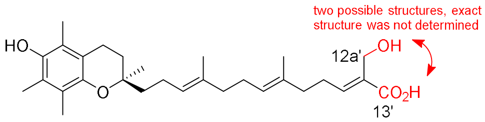
		α-T-5‘-COOH 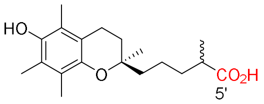	α-TE-11‘-COOH 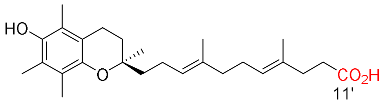

[a]The nomenclature of structurally related compounds follows this principle.

Table S3. Conditions for the quantification of 27a and its metabolites by UPLC-MS/MS

Metabolite	Transition	Collision energy [eV]	External standard	Lower limit of quantitation [nM][a]
α-TE-12a',13'-diCH_2OH (**27a**)	455 → 135	-55	α-TE-12a',13'-diCH_2OH (**27a**)	0.2
	455 → 163[b]	-45		
	455 → 438	-35		
α-TE-12a',13'-diCH_2OH (sulfate)	535 → 163[b]	-55	α-TE-12a',13'-diCH_2OH (**27a**)	/
	535 → 243	-45		
α-TE-12a'/13'-CH_2OH/COOH	469 → 163	-55	α-TE-12a',13'-diCH_2OH (**27a**)	/
α-TE-11'-COOH	413 → 163	-55	α-TE-12a',13'-diCH_2OH (**27a**)	/
α-TE-9'-COOH	385 → 163	-55	α-TE-12a',13'-diCH_2OH (**27a**)	/
α-TE-7'-COOH	345 → 163	-38	α-TE-12a',13'-diCH_2OH (**27a**)	/
α-TE-5'-COOH	317 → 163	-38	α-TE-12a',13'-diCH_2OH (**27a**)	/
α-T-13'-COOH (sulfate)	539 → 163	-46	α-T-13'-COOH (**12a**)[c]	1[c]

[a]signal-to-noise ratio ≥ 3. [b]transition used for quantitation. [c]analyzed according to Pein et al.[4]

SI SCHEMES

Scheme S1. SARs on cell-free 5-LOX inhibition[a]

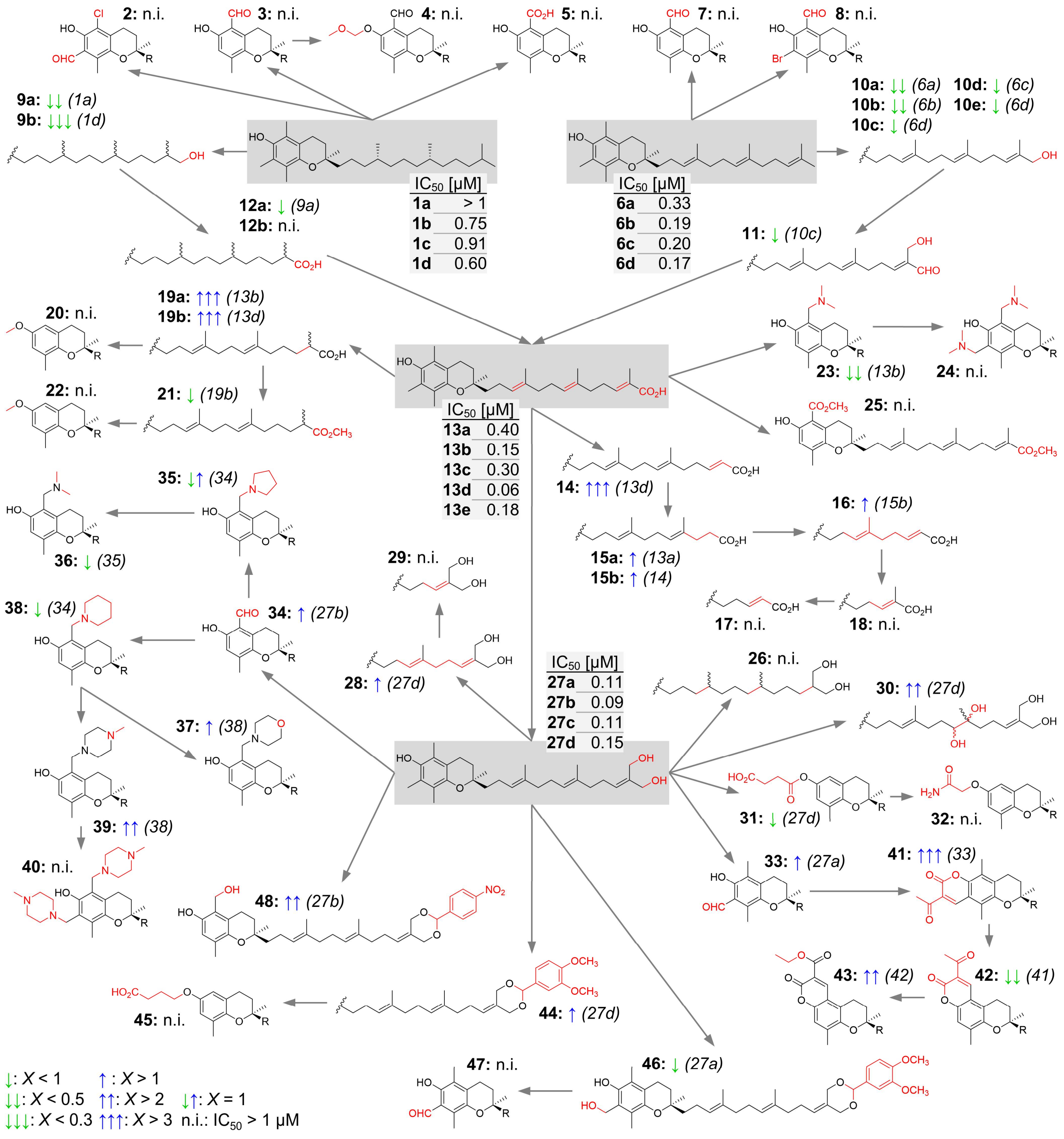

	IC50 [µM]
1a	> 1
1b	0.75
1c	0.91
1d	0.60

	IC50 [µM]
6a	0.33
6b	0.19
6c	0.20
6d	0.17

	IC50 [µM]
13a	0.40
13b	0.15
13c	0.30
13d	0.06
13e	0.18

	IC50 [µM]
27a	0.11
27b	0.09
27c	0.11
27d	0.15

[a]Fold-changes in IC_{50} values compared to the structurally parental compound (indicated in brackets) are visualized in the scheme by green downward (decreased IC_{50}) and blue upward arrows (increased IC_{50}) as indicated in the legend. R indicates that the side-chain is identical between parental and daughter compounds that are connected by an arrow. n.i., no inhibition.

Scheme S2. SARs on the inhibition of 5-LOX product formation in PMNL[a]

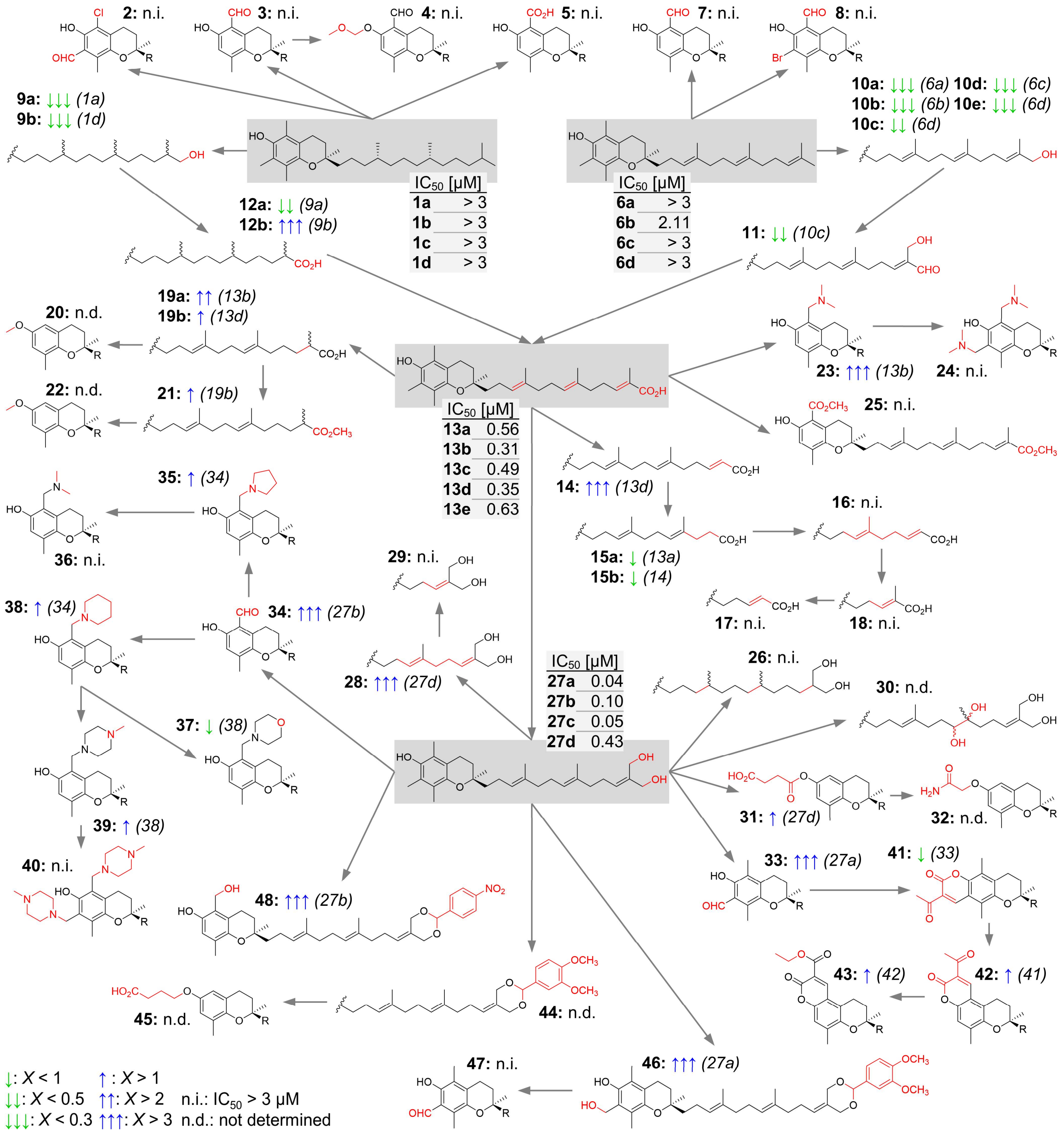

[a]Fold-changes in IC_{50} values compared to the structurally parental compound (indicated in brackets) are visualized in the scheme by green downward (decreased IC_{50}) and blue upward arrows (increased IC_{50}) as indicated in the legend. R indicates that the side-chain is identical between parental and daughter compounds that are connected by an arrow. n.i., no inhibition; n.d., not determined.

SI FIGURES

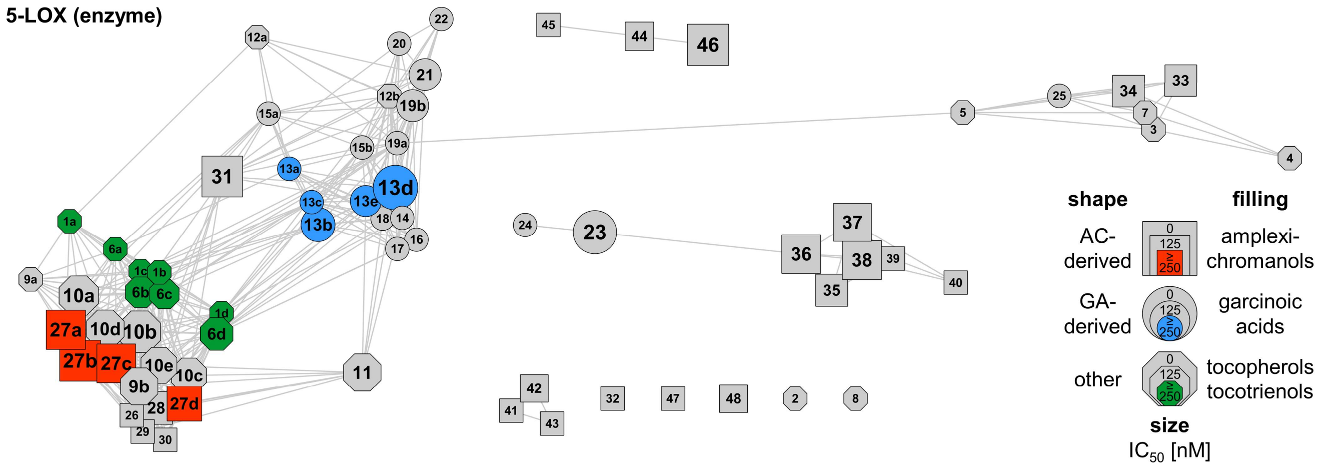

Figure S1. Correlation network of the compound library for inhibition of cell-free 5-LOX.

The network visualizes structural similarity between compounds calculated using Tanimoto similarity. Nodes represent individual compounds and connecting edges represent Tanimoto coefficients > 0.9. The node shape differentiates between derivatives derived from amplexichromanols (AC), garcinoic acids (GA), or other leads, and the filling highlights the parental series, i.e. amplexichromanol (red), garcinoic acid (blue), tocopherol and tocotrienol (green). The node size reflects the potency (IC_{50} values) of the compound to inhibit 5-LOX product formation in cell-free assays.

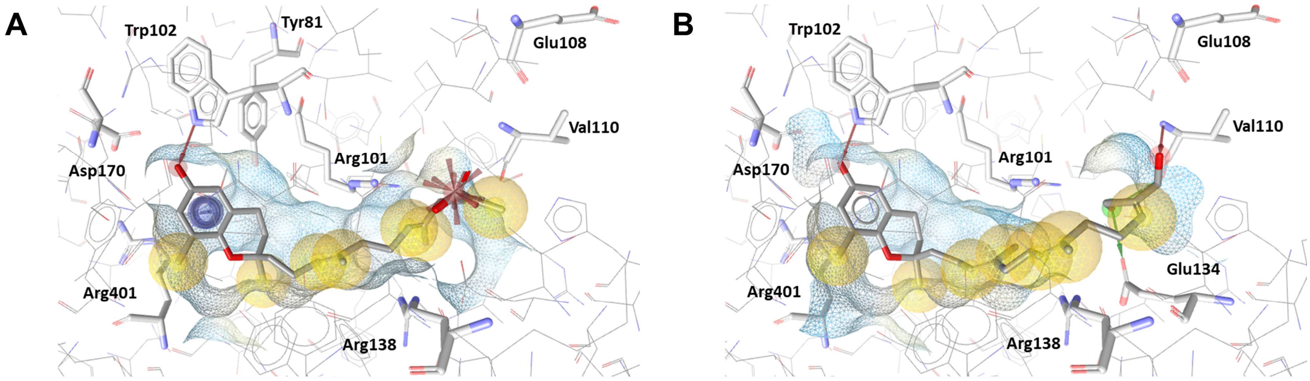

Figure S2. Molecular docking simulation of 5-LOX.

(A-B) Proposed interaction of **13d** (A) and **27d** (B) with 5-LOX at the interface of the catalytic and regulatory C2-like domain.

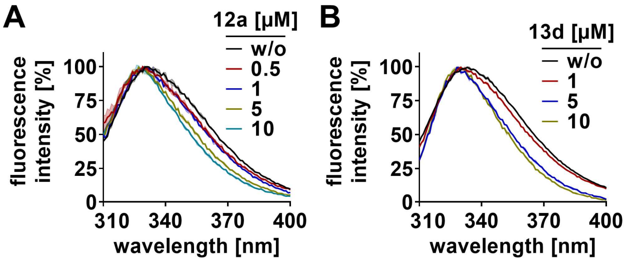

Figure S3. Fluorescence spectroscopic analysis of 5-LOX ligand interactions.

(A, B) Fluorescence excitation spectra as percentage of maximum fluorescence intensity shown for 5-LOX titrated with **12a** (A) and **13d** (B). Data are expressed as mean ± SEM (transparent area) from n = 2 independent experiments.

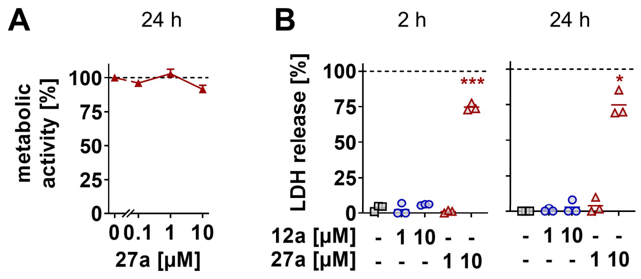

Figure S4. Effect of 27a on human monocyte and PBMC viability.

PBMC (A) or monocytes (B) were treated with **27a** or **12a** for 24 h (A, B) or 2 h (B). (A) Mitochondrial dehydrogenase activity analyzed by MTT assay. (B) Membrane integrity measured as LDH release into the culture medium. Data are expressed as mean + SEM (A) or mean with single values (B) from n = 4 (A), n = 3 (B) independent experiments. *$p < 0.05$, ***$p < 0.001$ vs. control; RM one-way ANOVA + Tukey *post hoc* test.

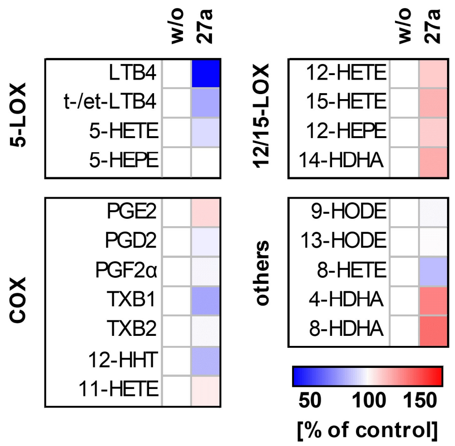

Figure S5. Compound 27a selectively inhibits 5-LOX product formation in activated monocytes.

Heatmap showing the effect of **27a** (1 µM) on the lipid mediator profile in A23187/AA-treated monocytes that were pre-activated with LPS. HODE, hydroxyoctadecadienoic acid; t-/et-LTB_4, LTB_4 isomers; TX, thromboxane. Data are expressed as percentage change to vehicle control and are given as mean from n = 3 independent experiments.

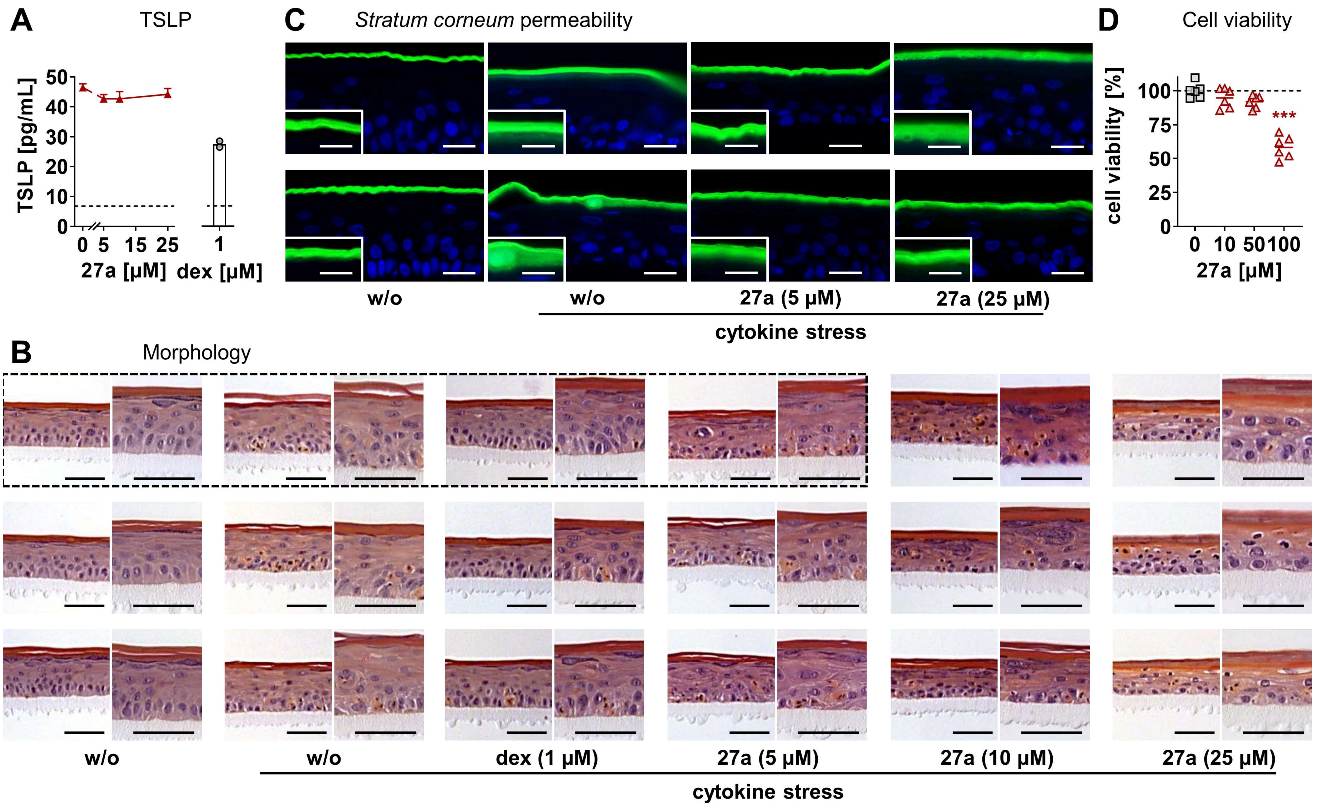

Figure S6. Compound 27a attenuates cytokine-triggered defects in reconstructed human epidermis (RHE).

RHE exposed to **27a** or dexamethasone (dex) was treated with a cytokine cocktail for 2 days (A) or 4 days (B-D) to trigger the inflammatory reaction. (A) Concentration of thymic stromal lymphopoietin (TSLP) in the growth medium. The dotted line indicates basal levels without cytokine stress. (B) Morphological changes visualized by hematoxylin and eosin staining (scale bar: 50 µm). Images in the dotted box are shown in Fig. 5B. (C) Impermeability of the *stratum corneum*. The *stratum corneum* of cytokine-stressed RHE becomes permeable for Lucifer yellow (green) that diffuses into the viable cell layers, as shown in the inserts in higher magnification (scale bar outer box: 20 µm, scale bar insert: 10 µm; exemplary images from three independent experiments that are not shown in Fig. 5E). (D) Mitochondrial dehydrogenase activity analyzed by MTT assay. Data are expressed as mean + SEM (A, **27a**) with single values (A, dex) or mean with single values (D) from n = 2 (A), n = 3 (B, C) independent experiments or n = 6 based on three independent experiments in biological duplicates (D). ***$p < 0.001$ vs. control; ordinary one-way ANOVA + Tukey *post hoc* test.

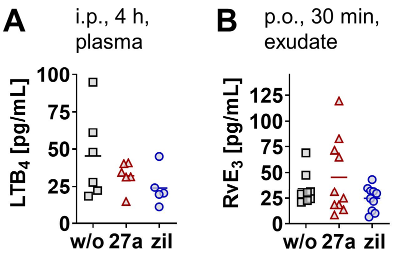

Figure S7. Effect of 27a on resolvin (Rv)E3 and systemic LTB_4 levels in mice with acute peritonitis.

Mice received **27a** (10 mg/kg, A: i.p., B: p.o.) or zileuton (zil; 10 mg/kg, A: i.p., B: p.o.) and were sacrificed 4 h (A) or 30 min (B) post zymosan injection. (A) LTB_4 levels in plasma analyzed by ELISA. (B) RvE3 levels in the exudate analyzed by UPLC-MS/MS. Data are expressed as mean with single values from n = 6 (A, w/o and **27a**), n = 5 (A, zil), n = 9 (B, w/o), n = 10 (B, **27a** and zil) mice. Two-tailed unpaired *t*-test of log data.

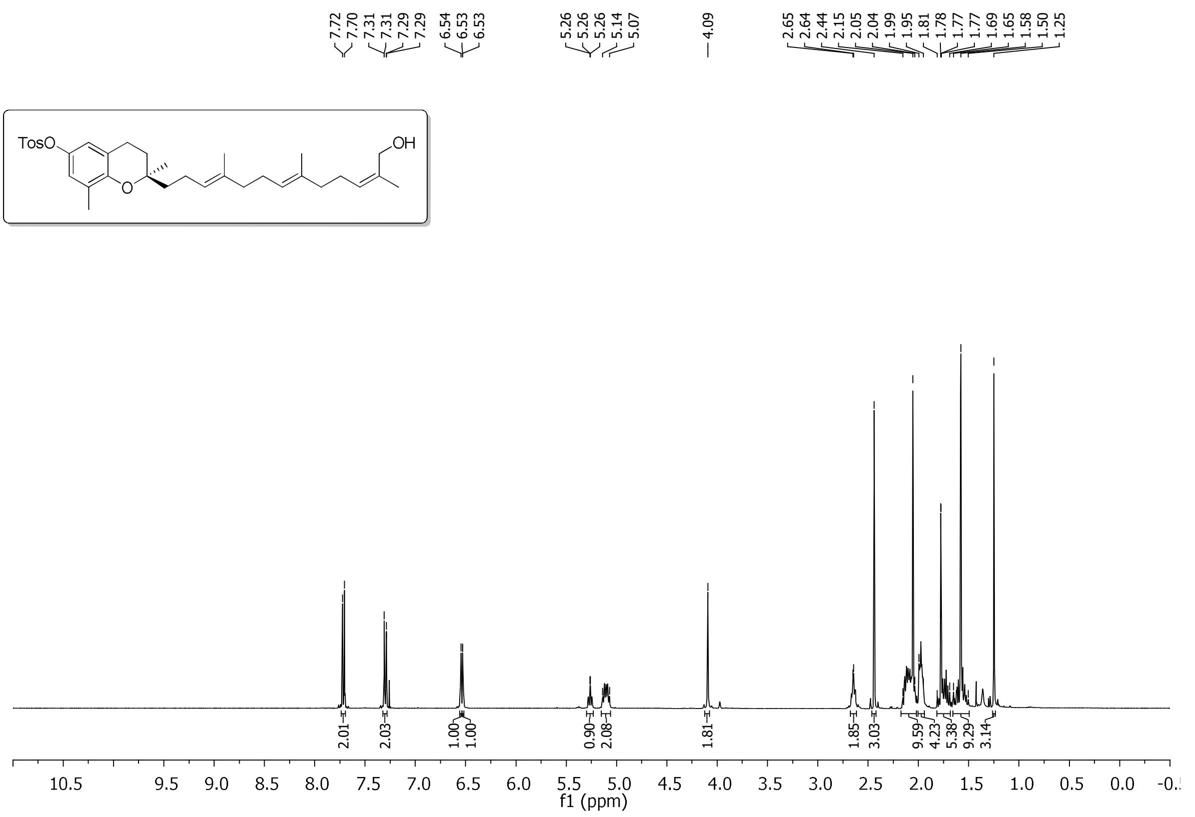

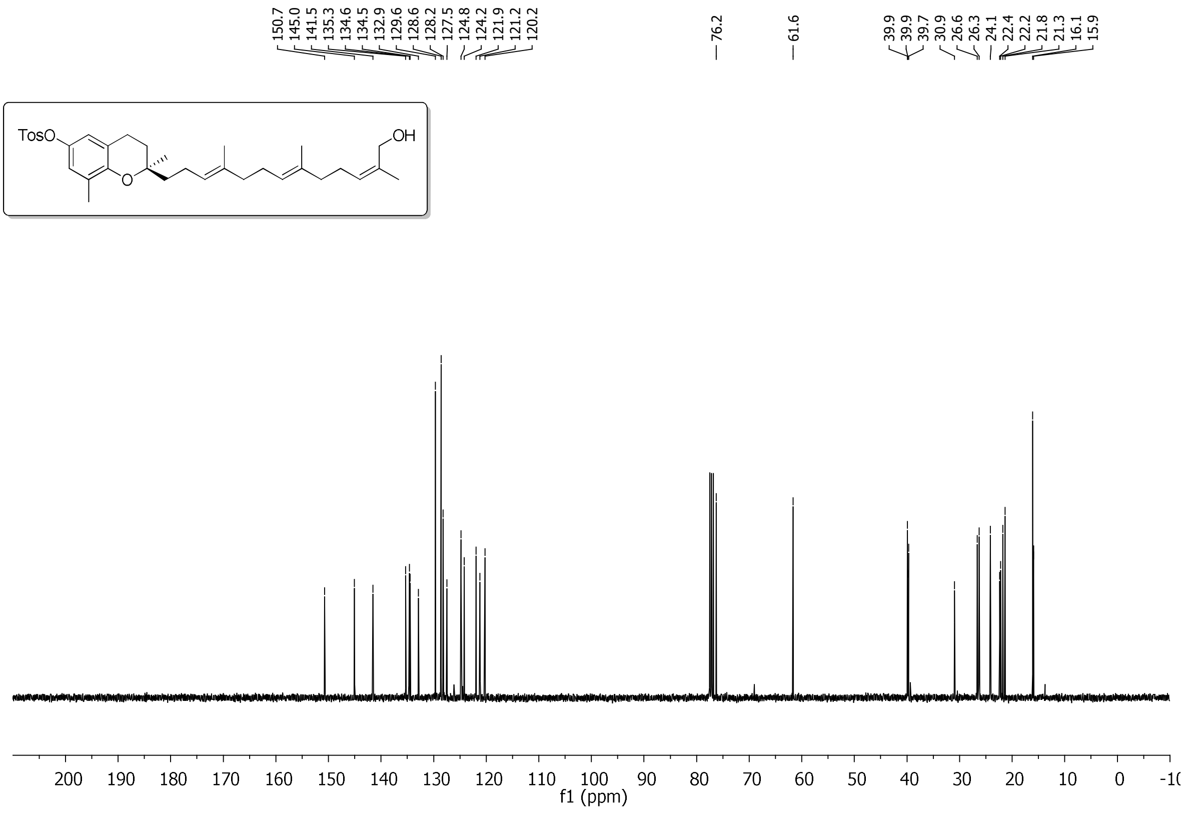

Figure S8. ^{1}H **and** ^{13}C **NMR spectra of 49 in acetone-*d*$_6$**

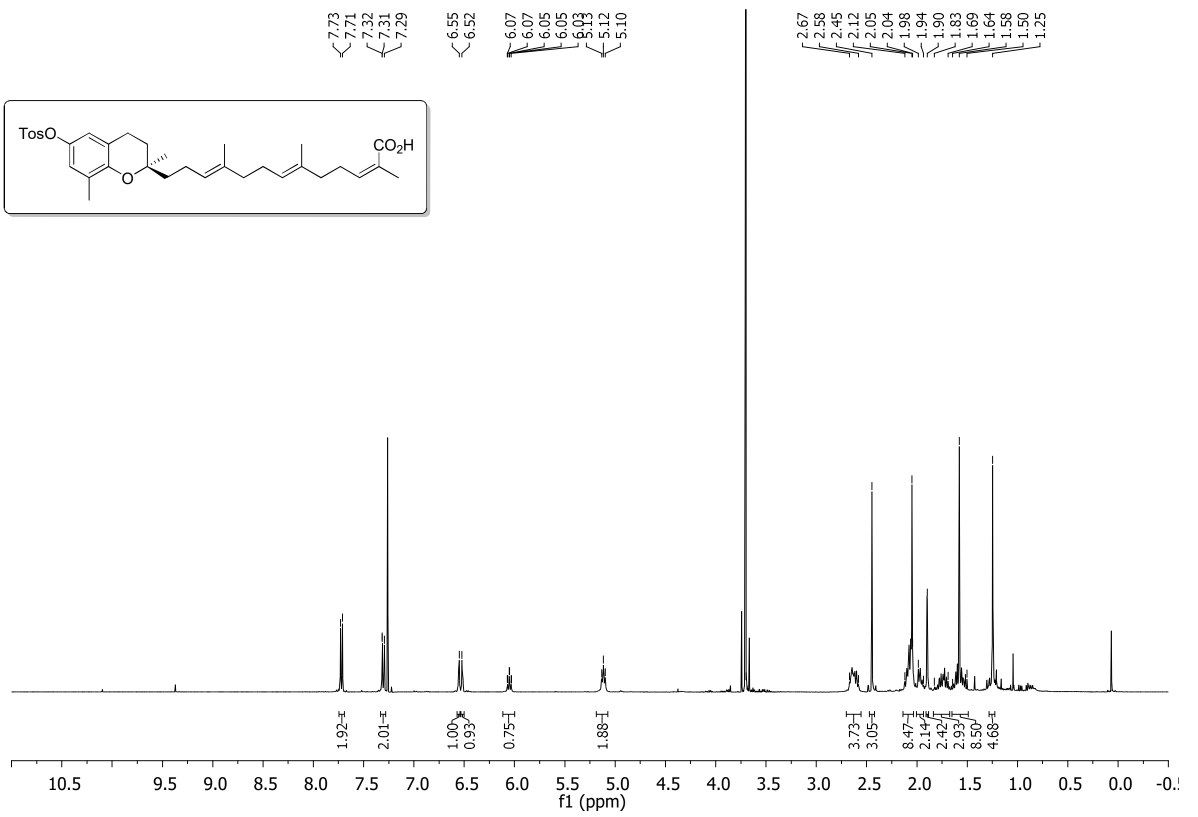

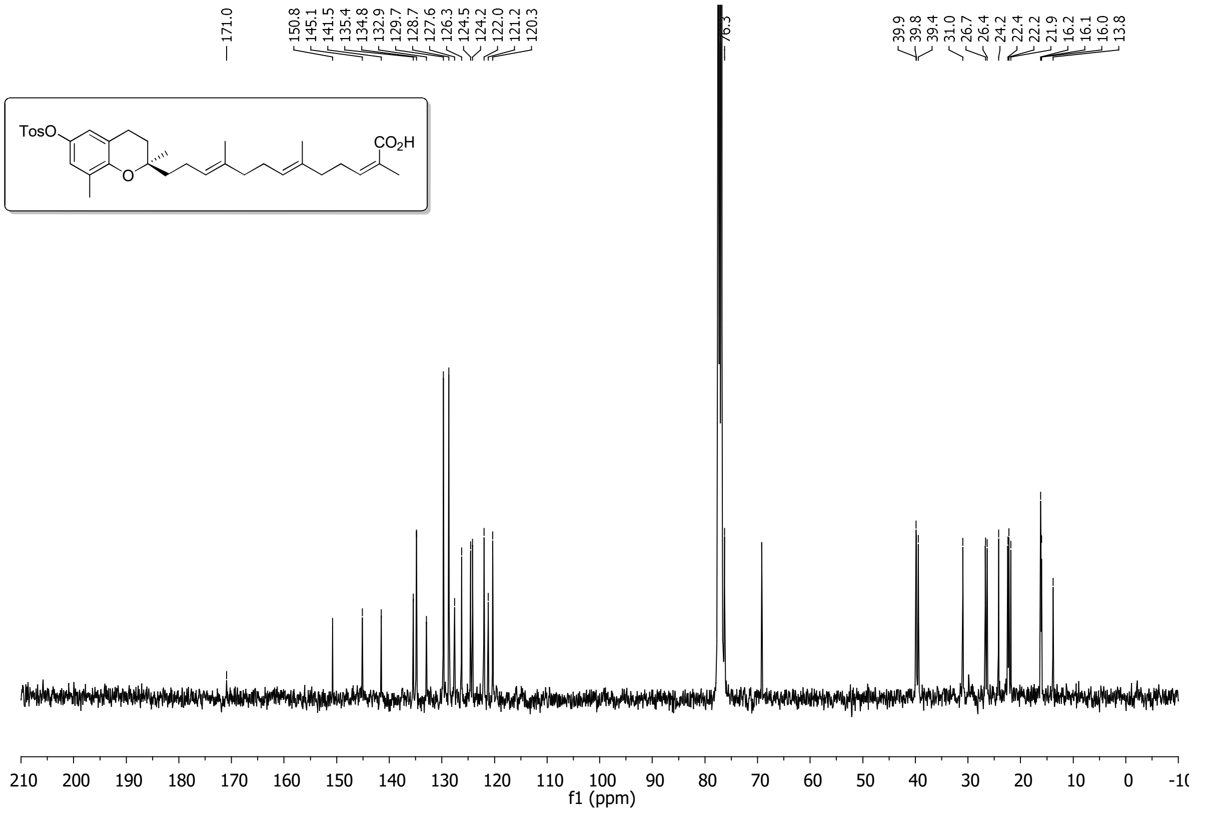

Figure S9. ^{1}**H and** ^{13}**C NMR spectra of 50 in CDCl**$_3$

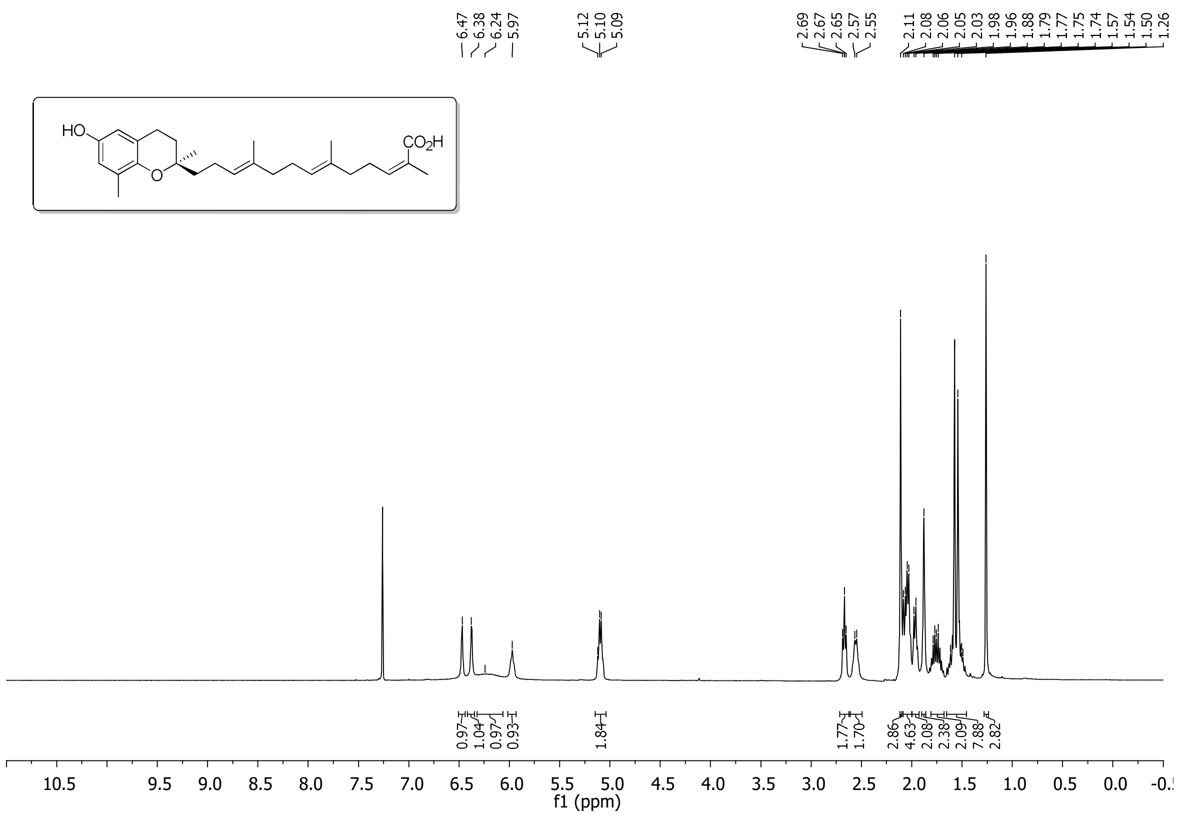

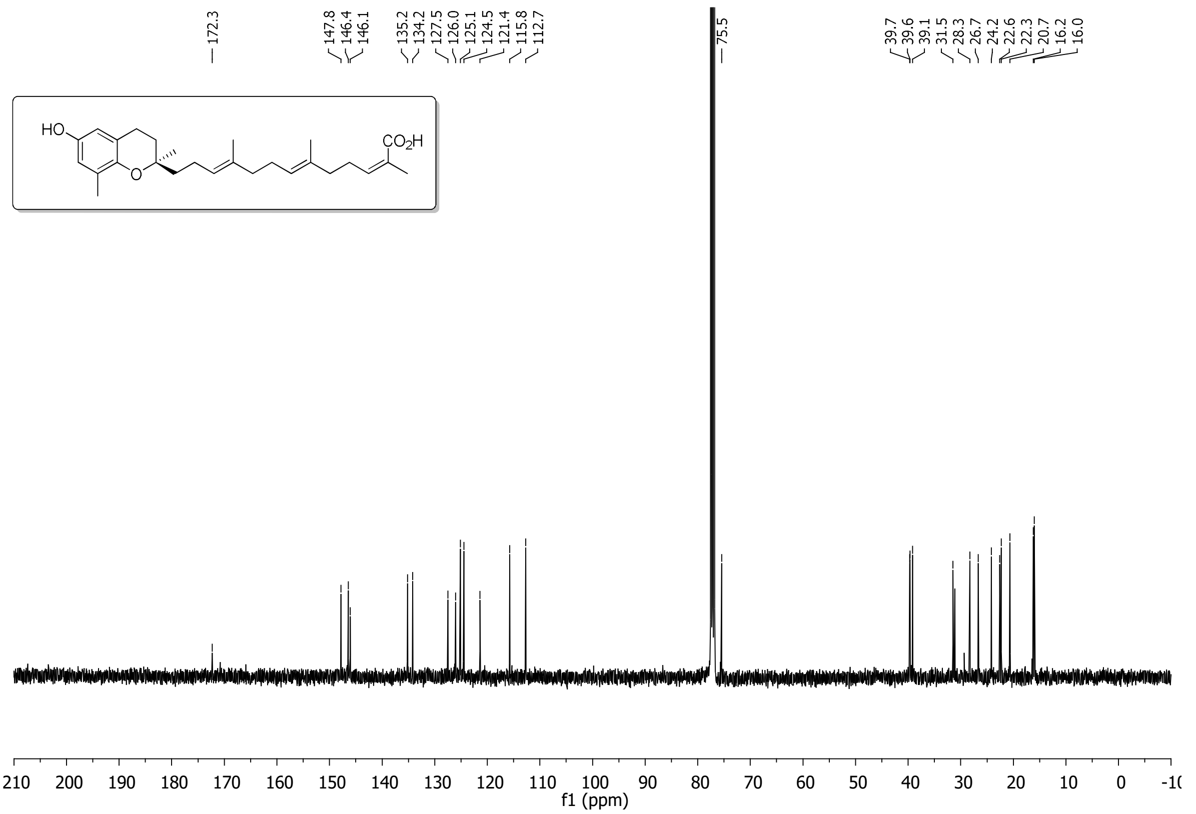

Figure S10. ^{1}H **and** ^{13}C **NMR spectra of 13e in** $CDCl_3$

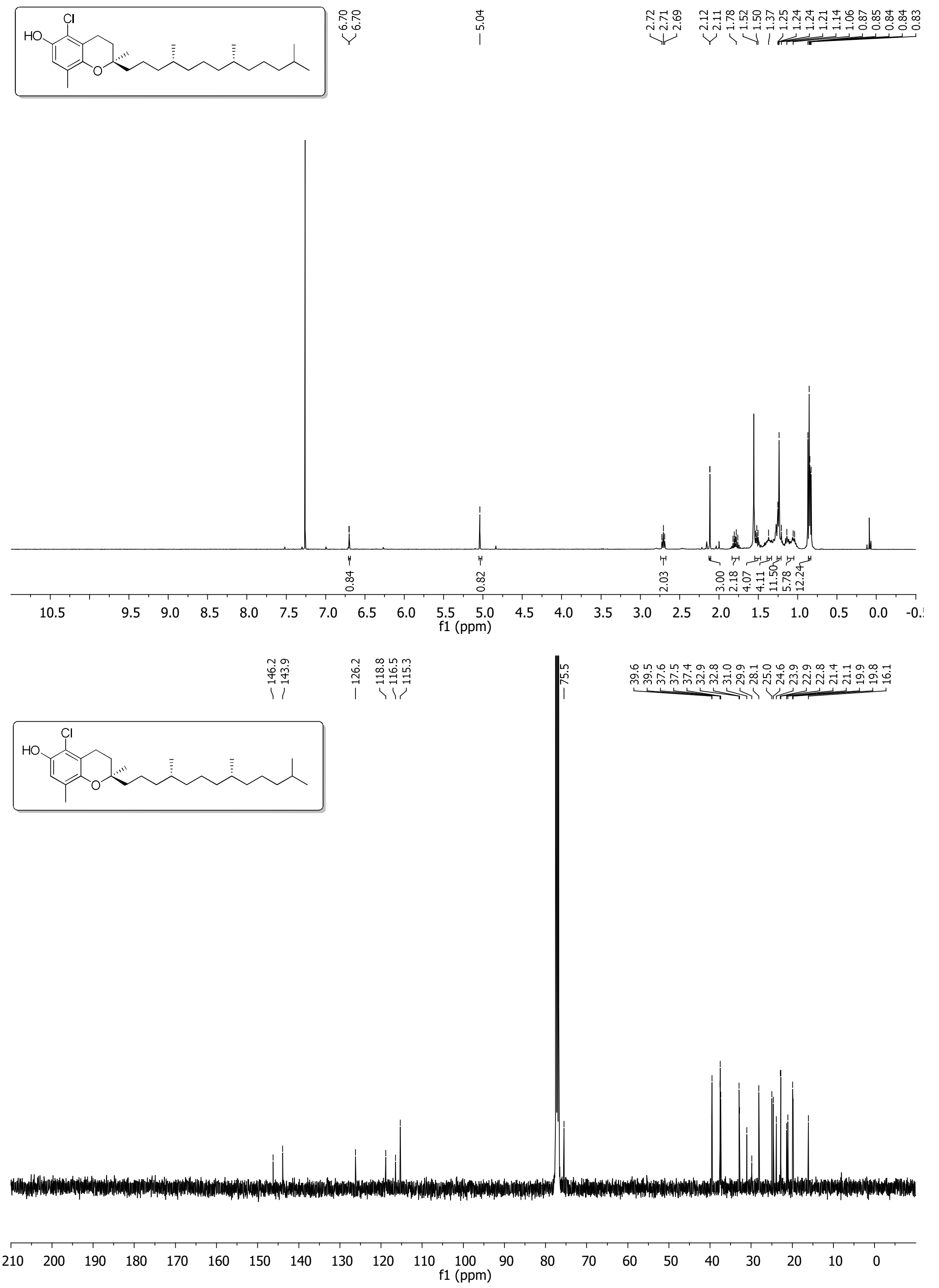

Figure S11. ^{1}H and ^{13}C NMR spectra of **51** in $CDCl_3$

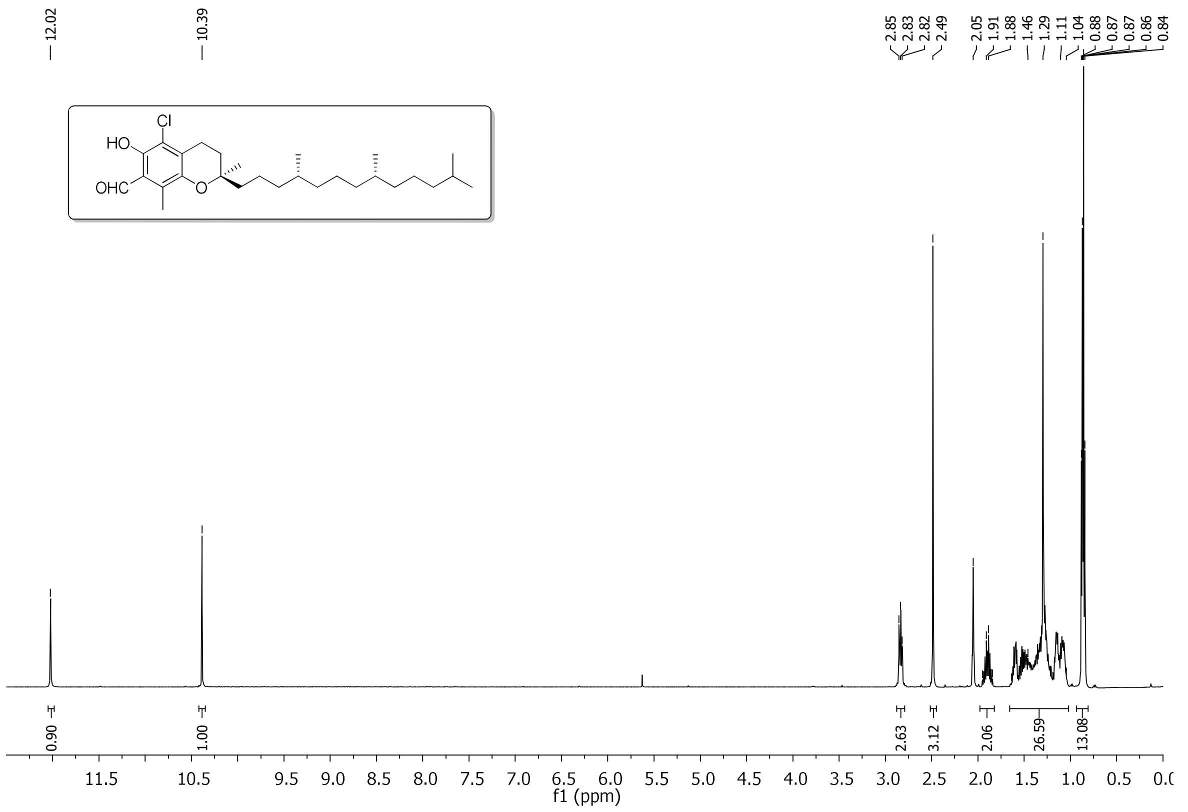

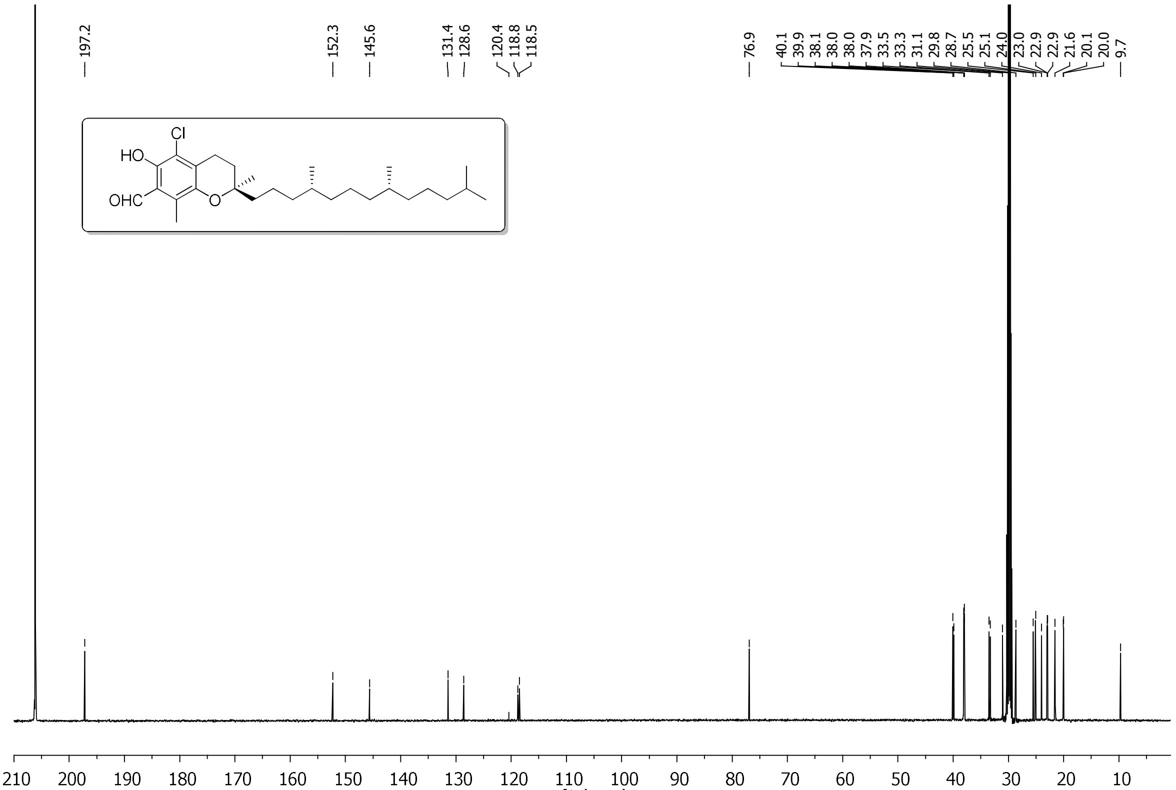

Figure S12. ^{1}H and ^{13}C NMR spectra of 2 in acetone-d_6

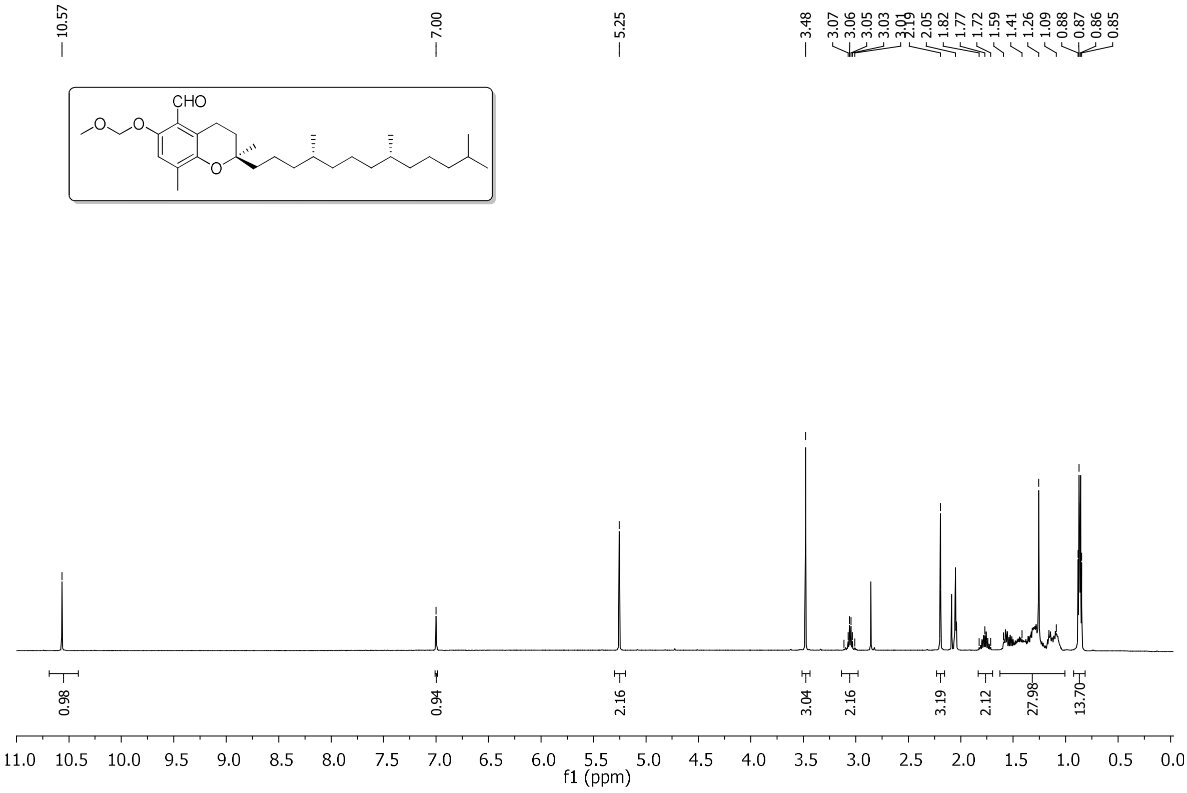

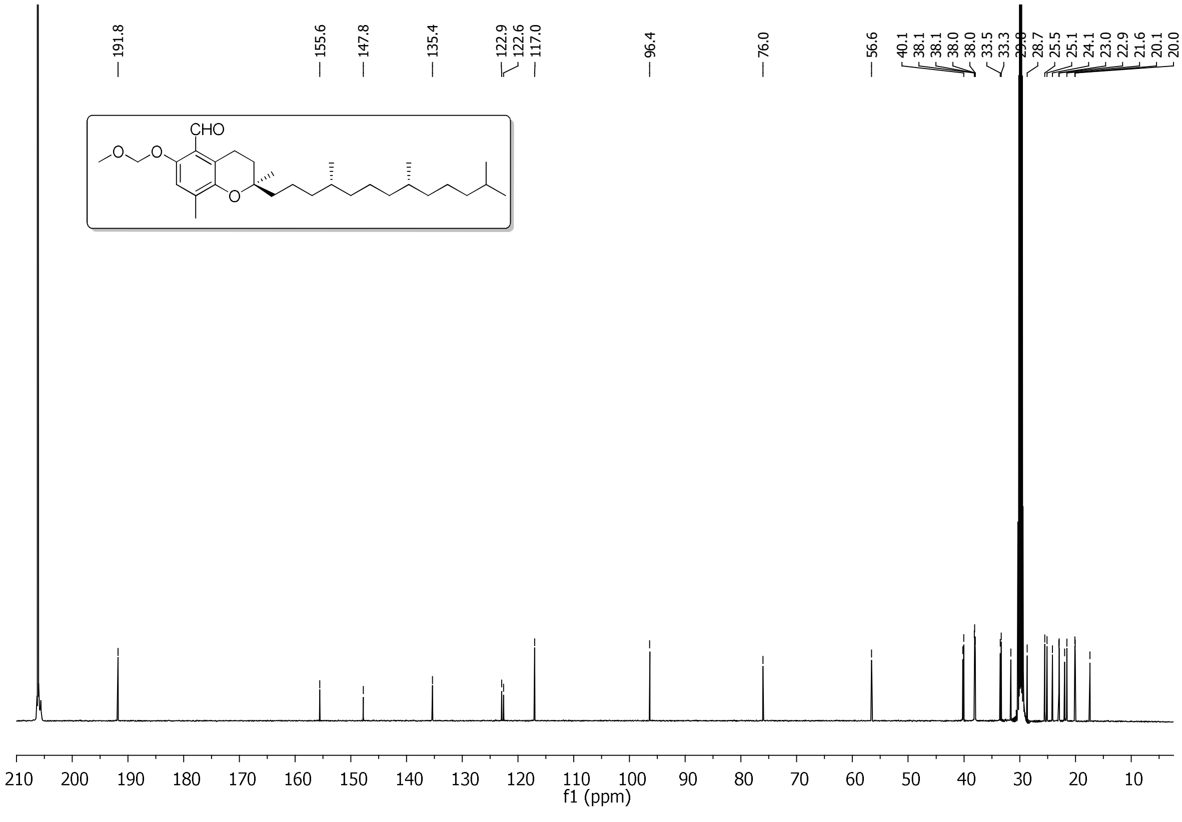

Figure S13. ^{1}H and ^{13}C NMR spectra of 4 in acetone-d_6

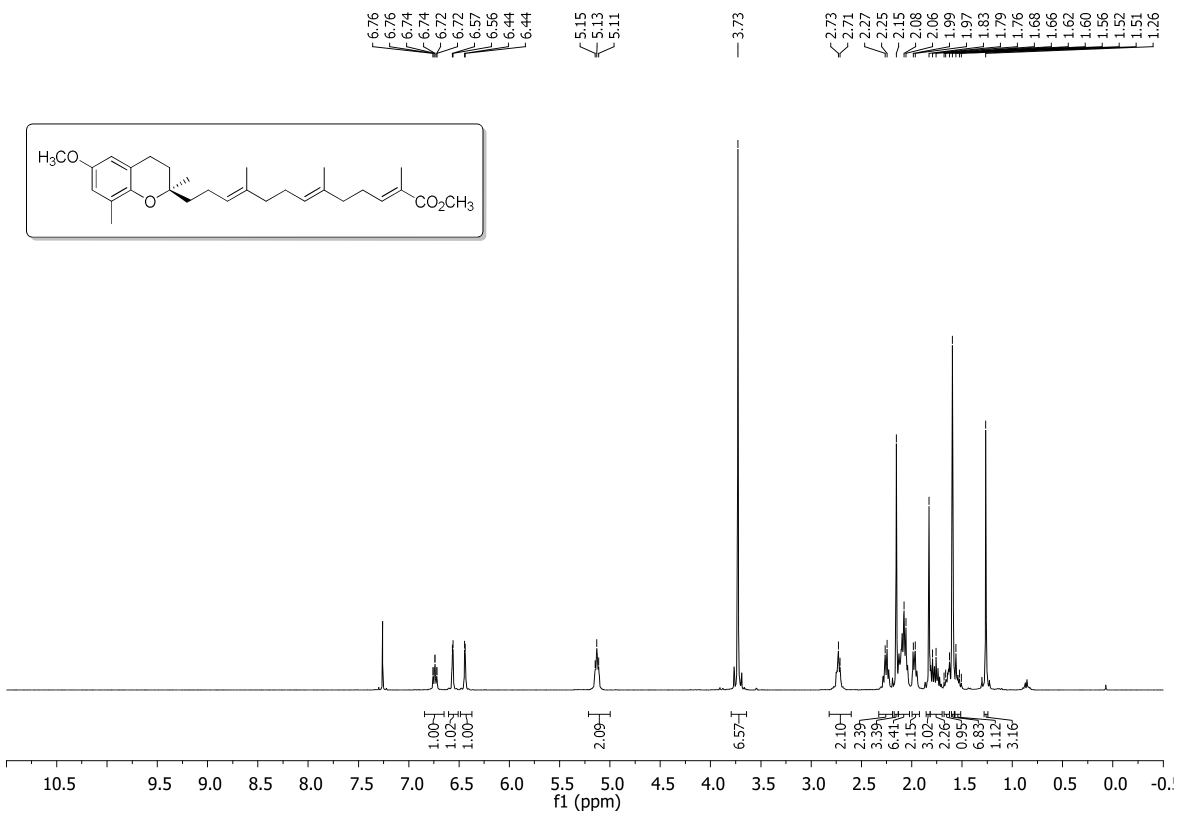

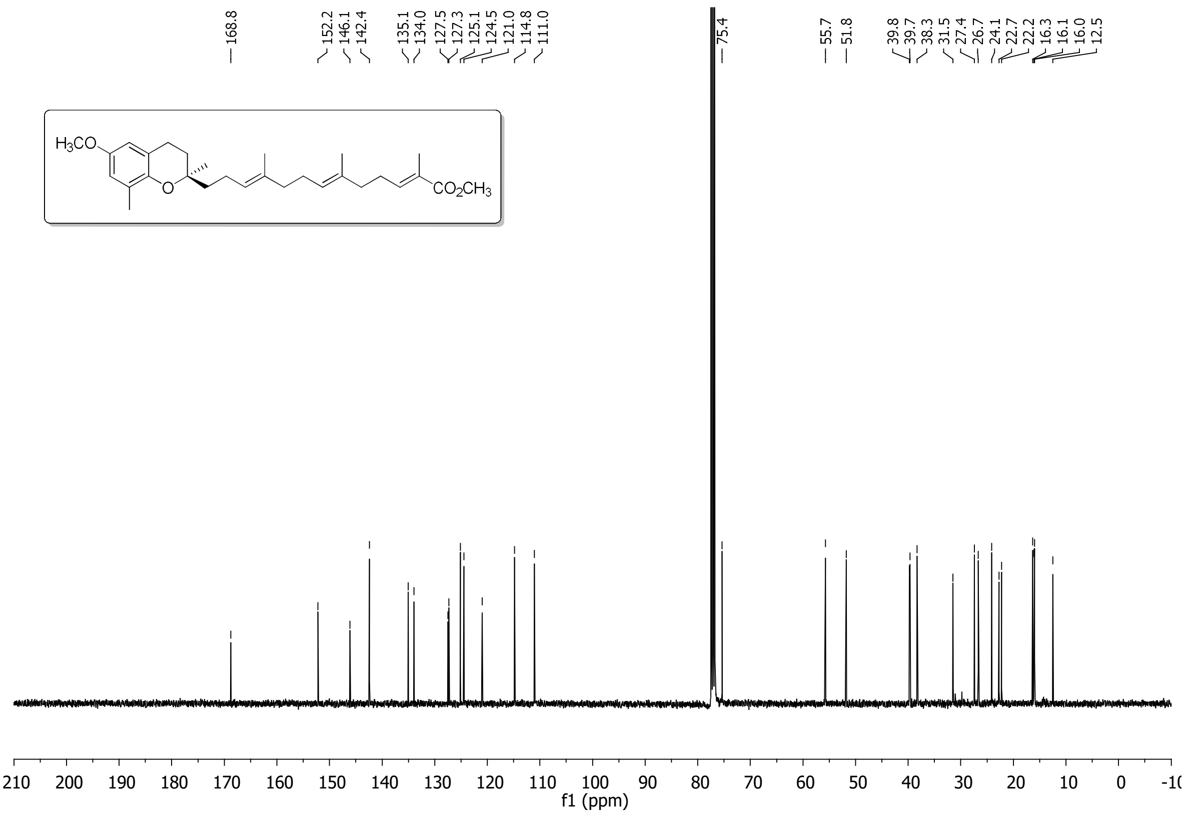

Figure S14. ^{1}H **and** ^{13}C **NMR spectra of 53 in** $CDCl_3$

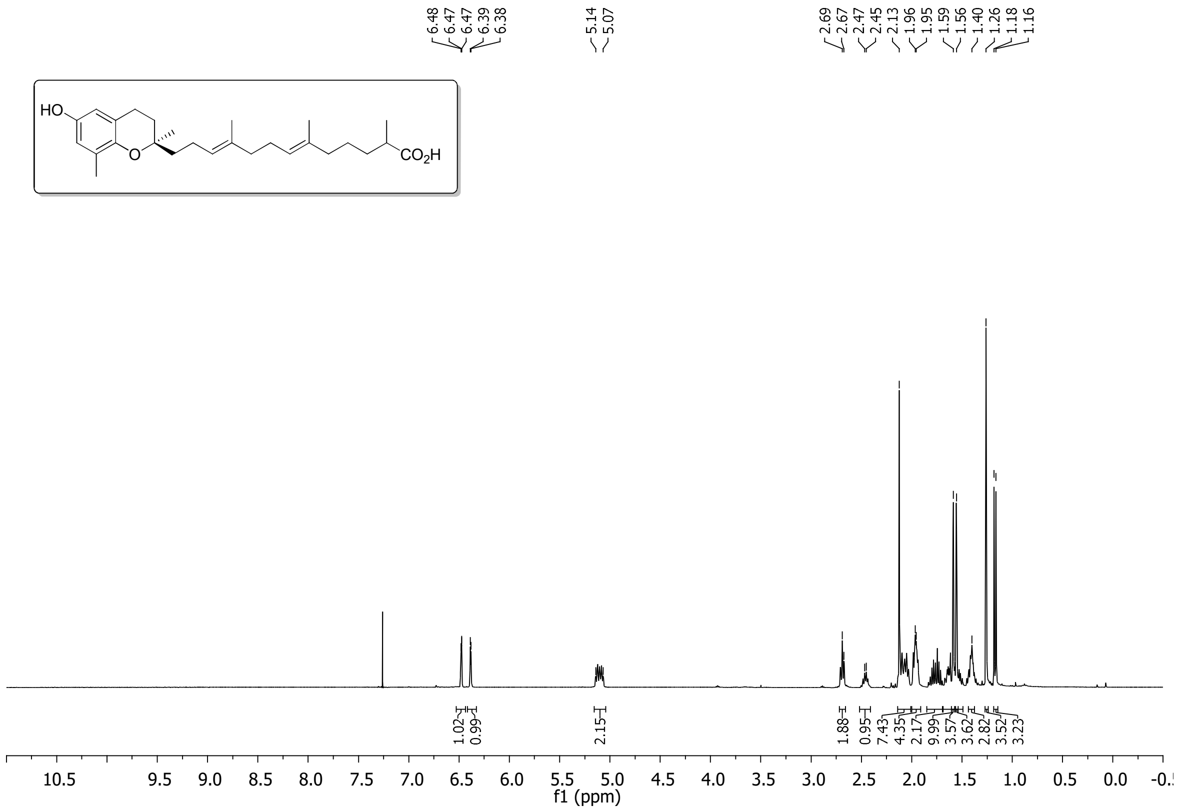

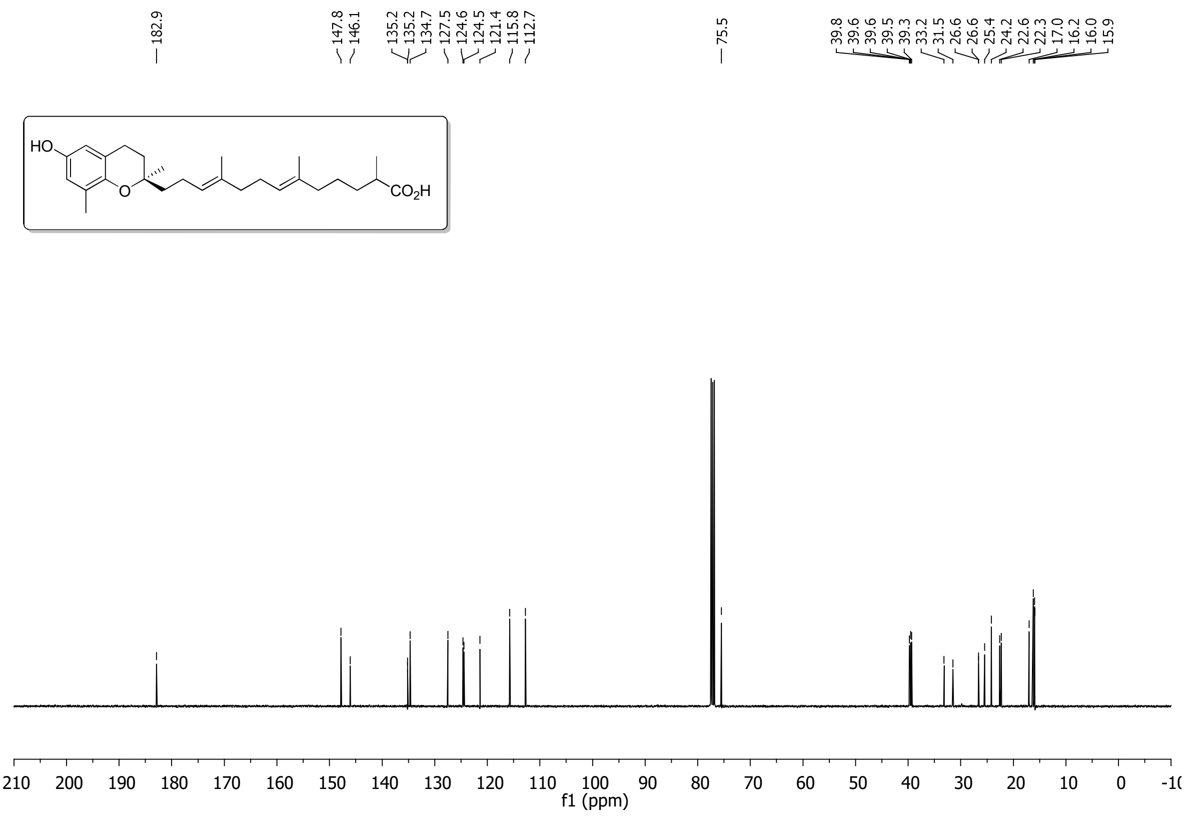

Figure S15. ^{1}H **and** ^{13}C **NMR spectra of 19b in** $CDCl_3$

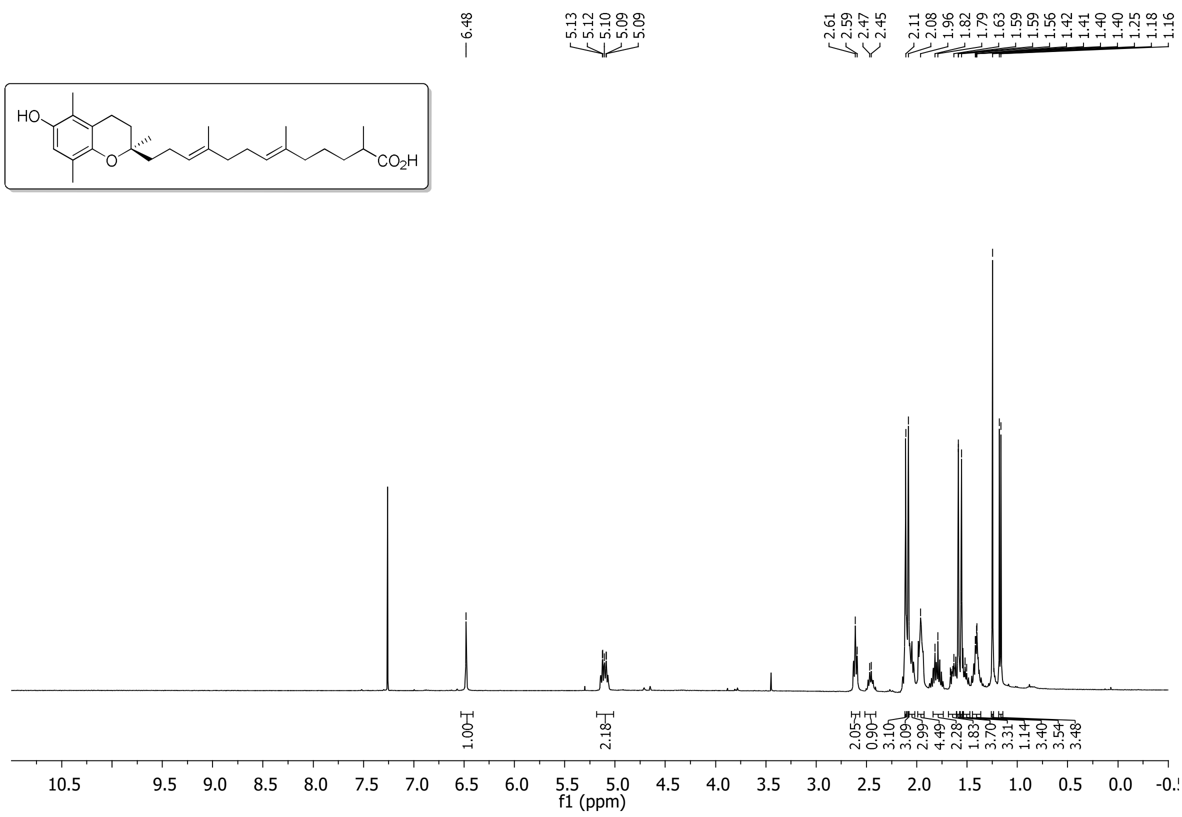

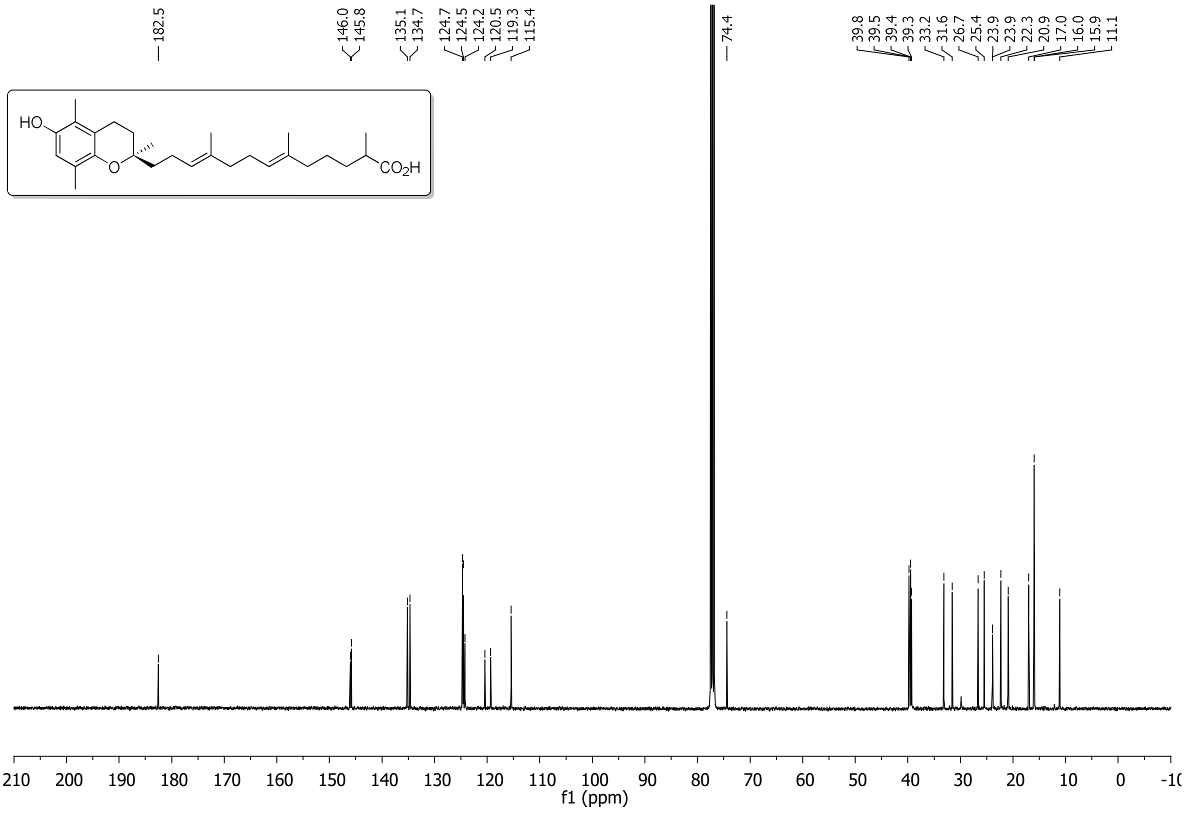

Figure S16. ^{1}H and ^{13}C NMR spectra of 19a in $CDCl_3$

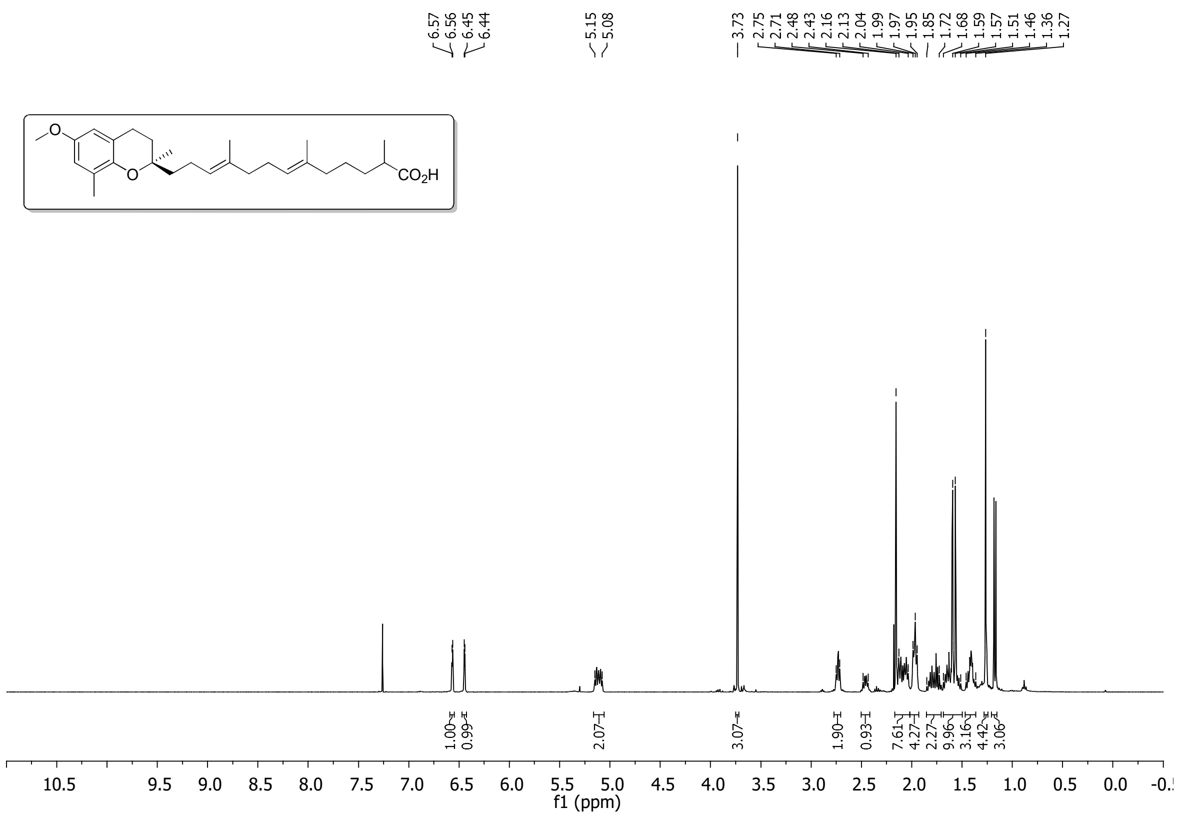

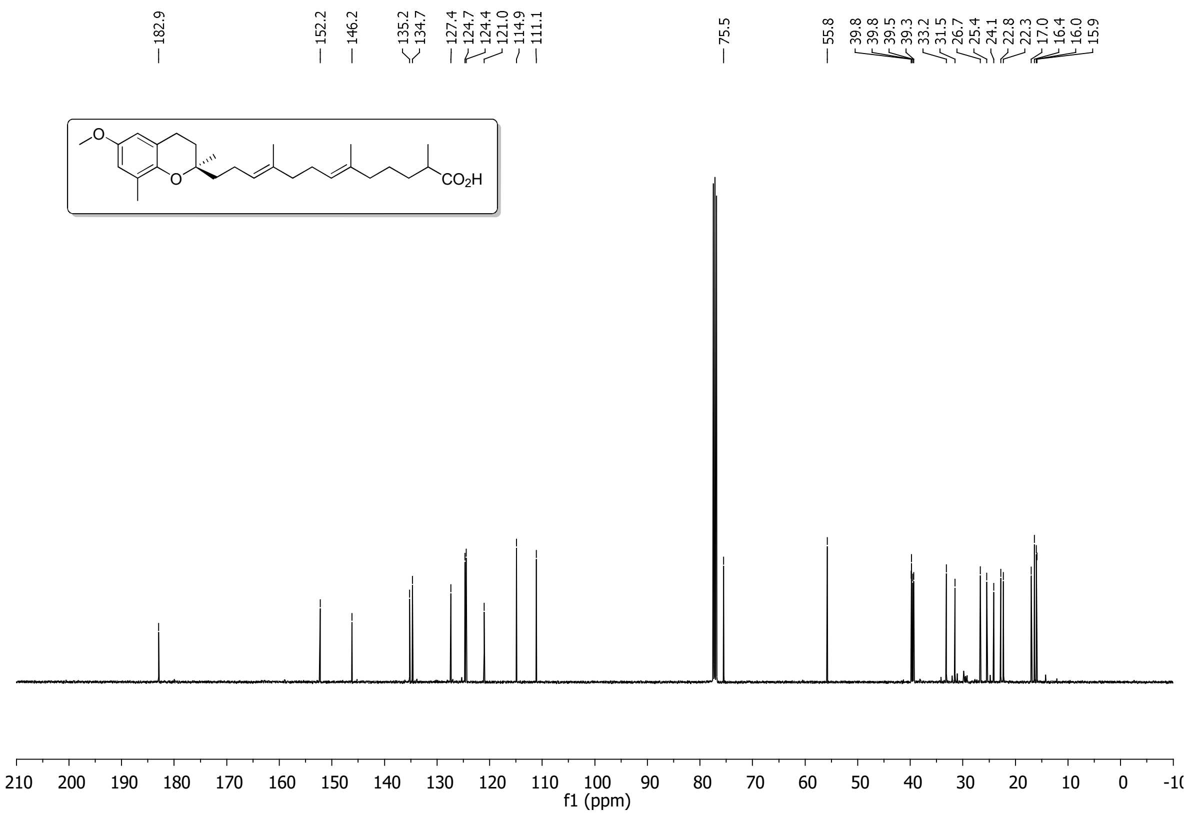

Figure S17. ^{1}H **and** ^{13}C **NMR spectra of 20 in** $CDCl_3$

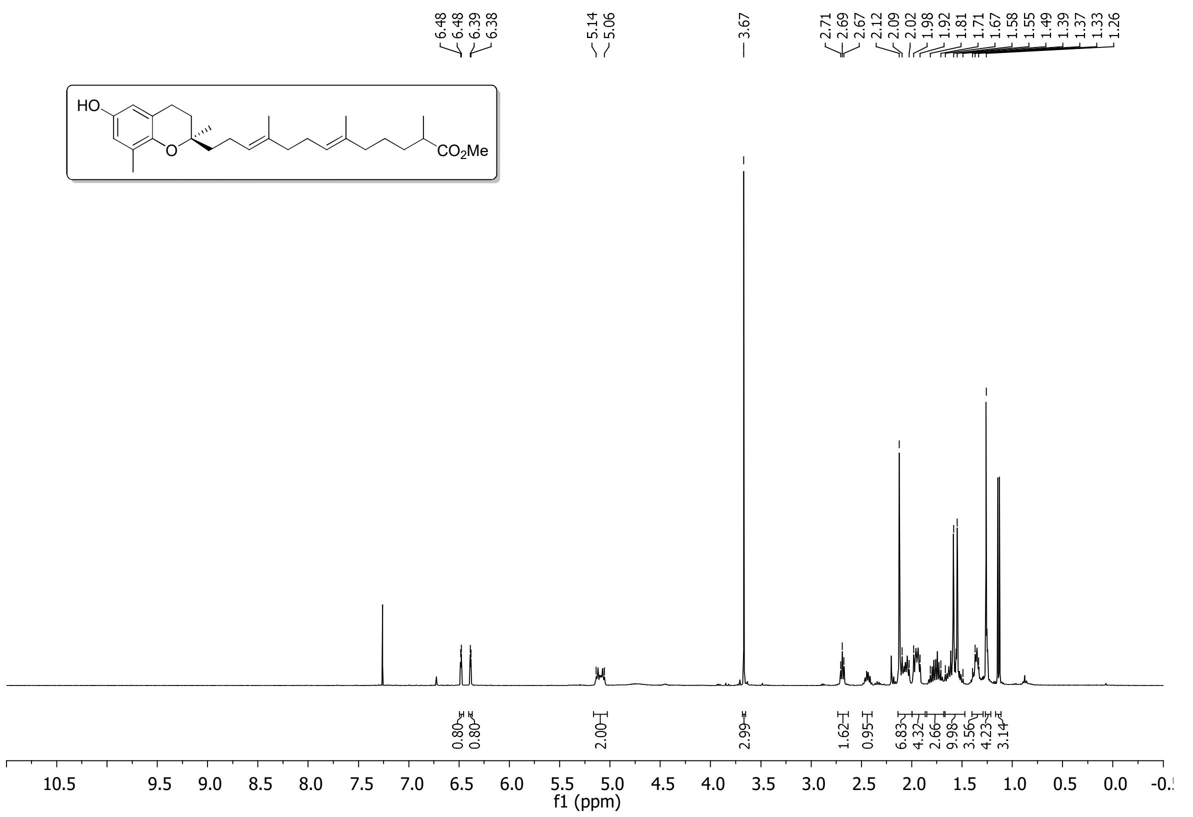

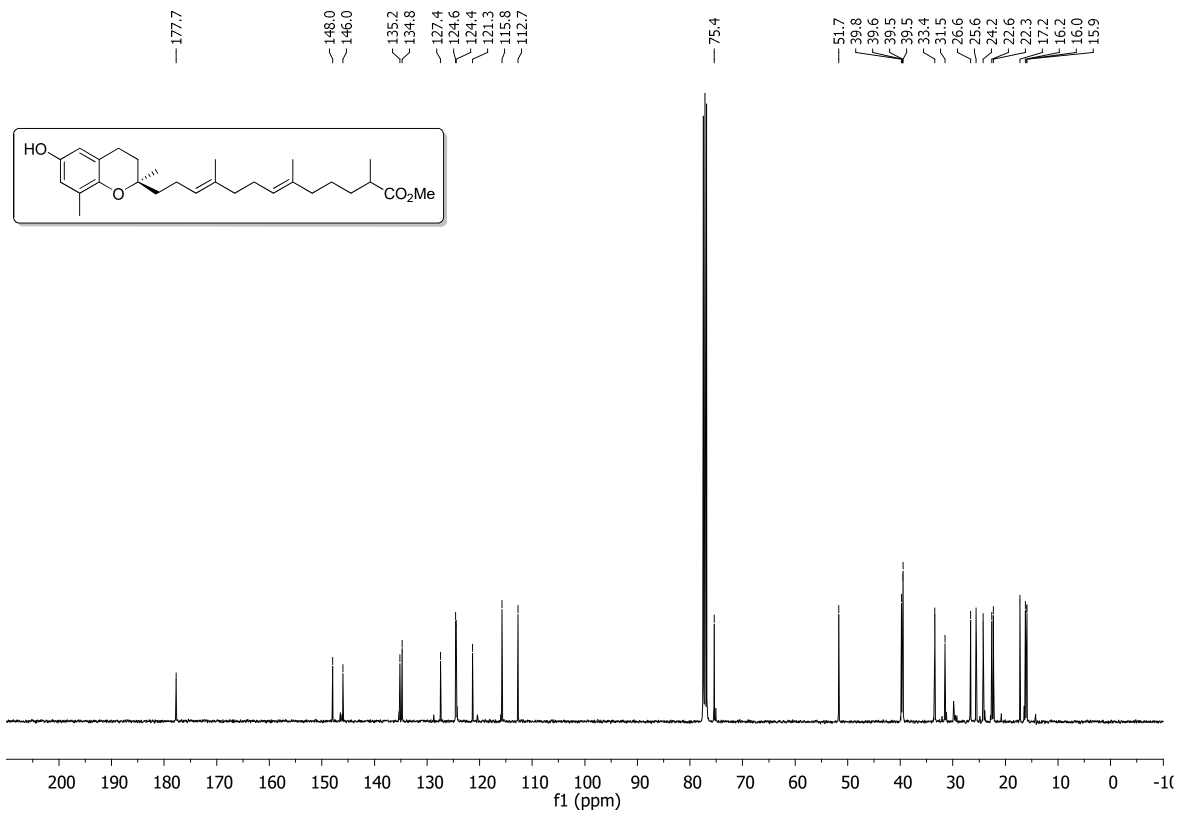

Figure S18. ^{1}H and ^{13}C NMR spectra of 21 in $CDCl_3$

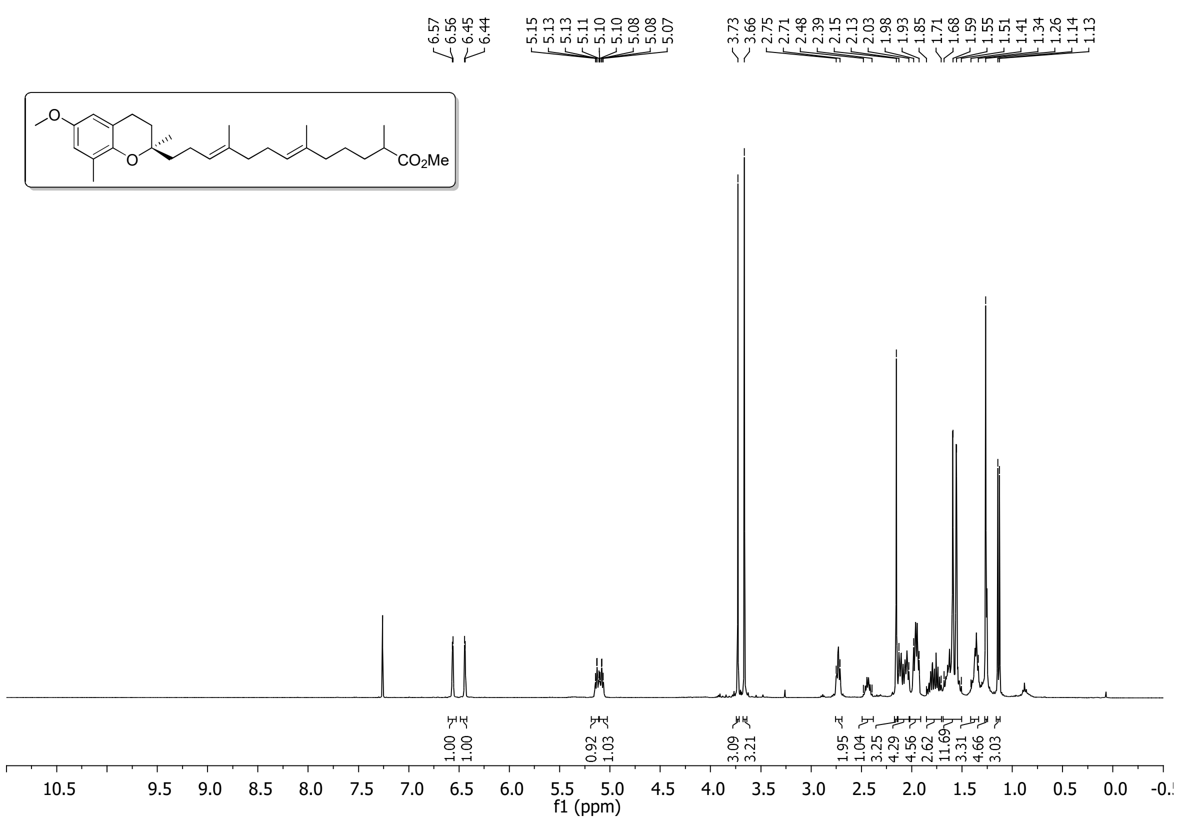

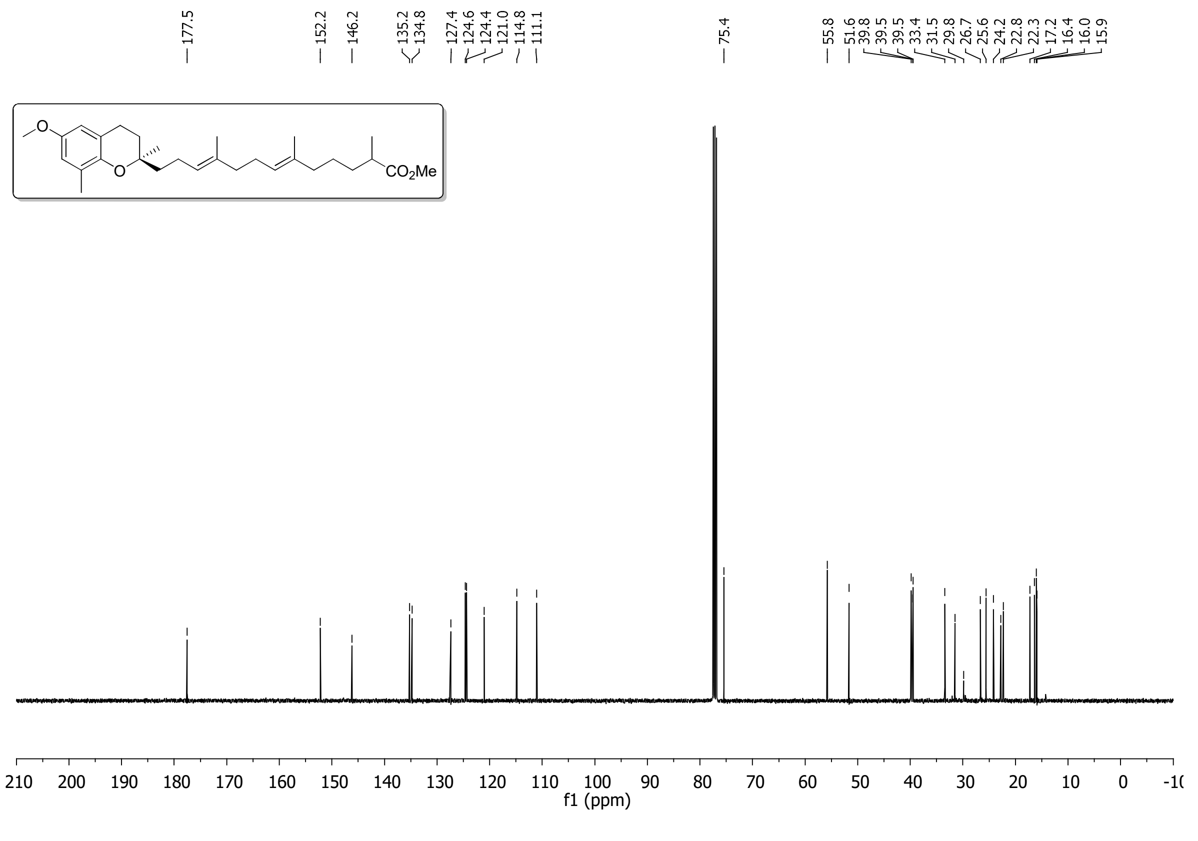

Figure S19. ^1H and ^{13}C NMR spectra of 22 in $CDCl_3$

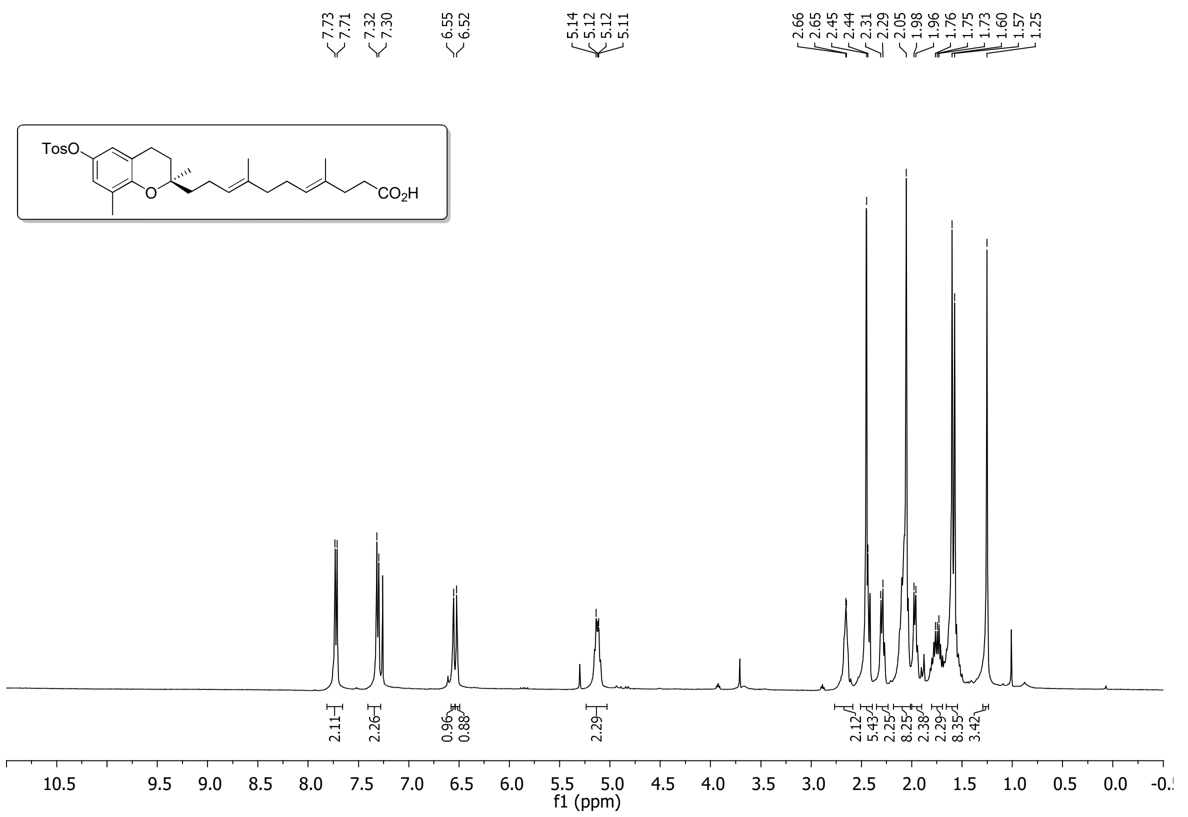

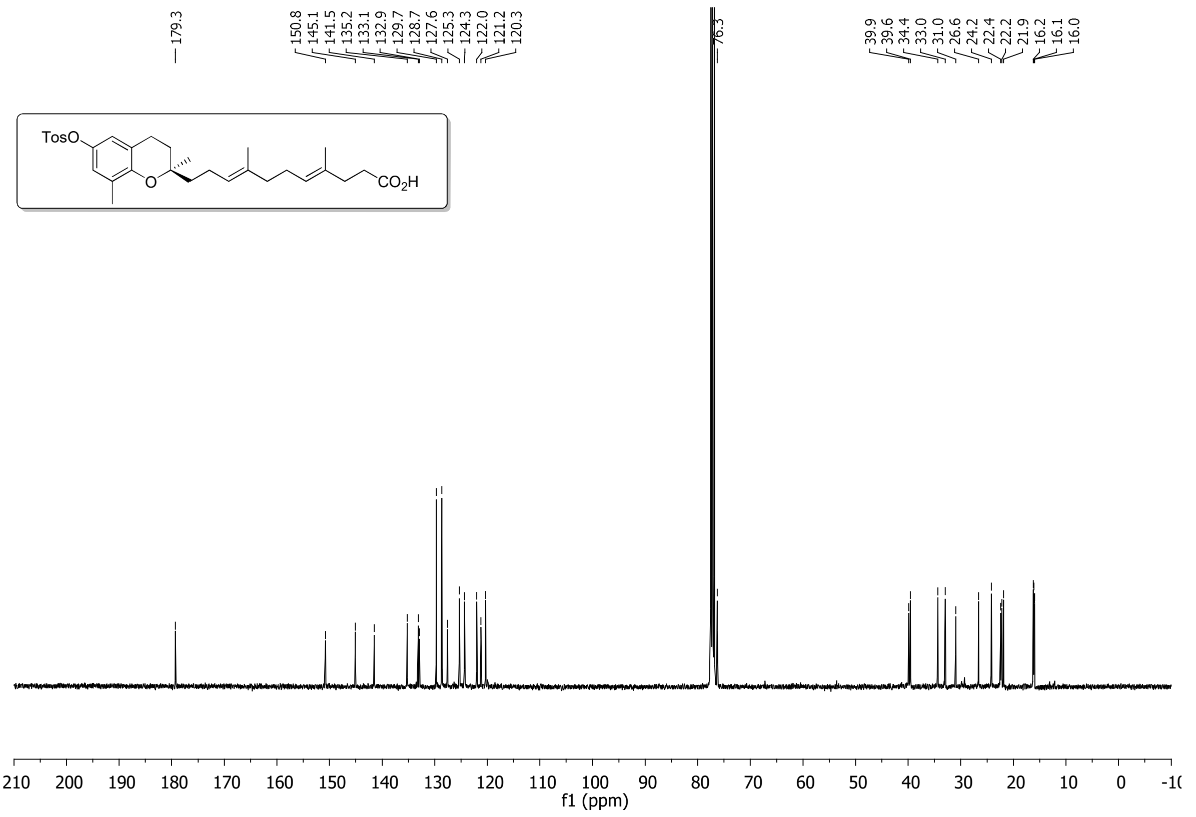

Figure S20: ^{1}H and ^{13}C NMR spectra of 55 in $CDCl_3$

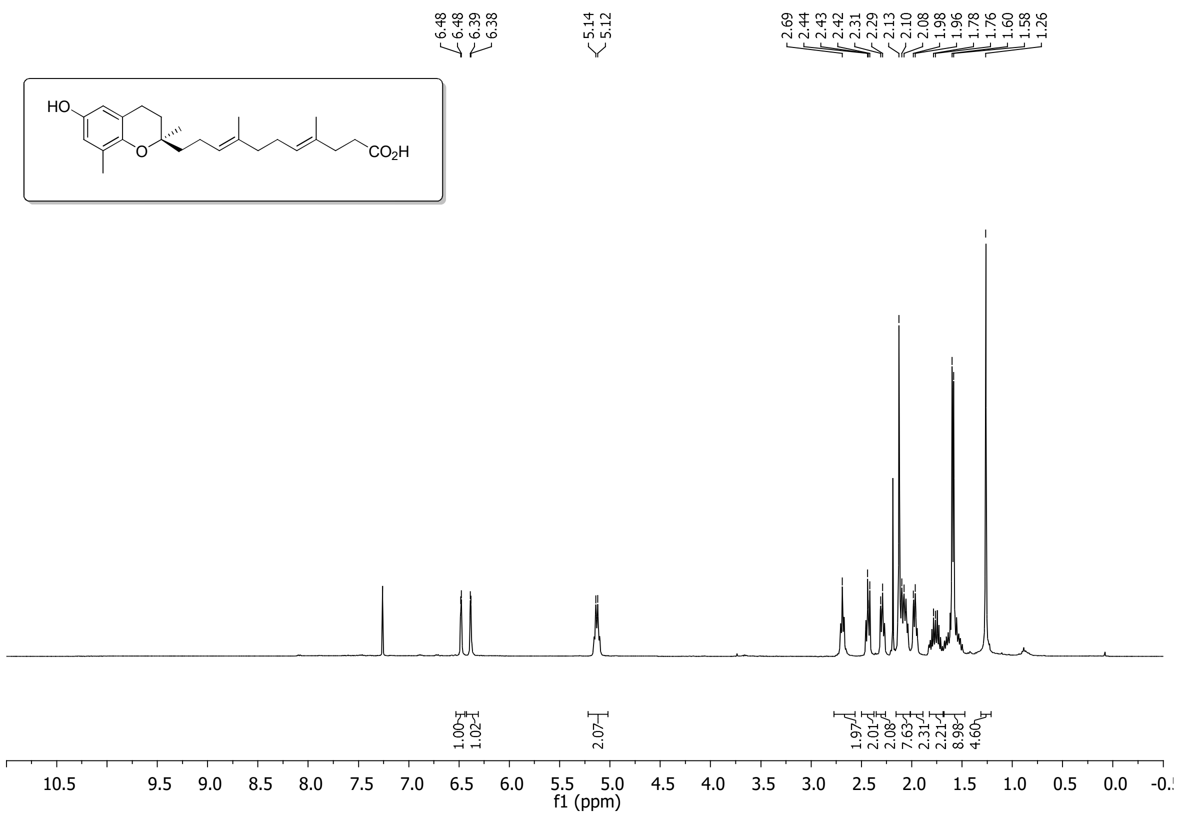

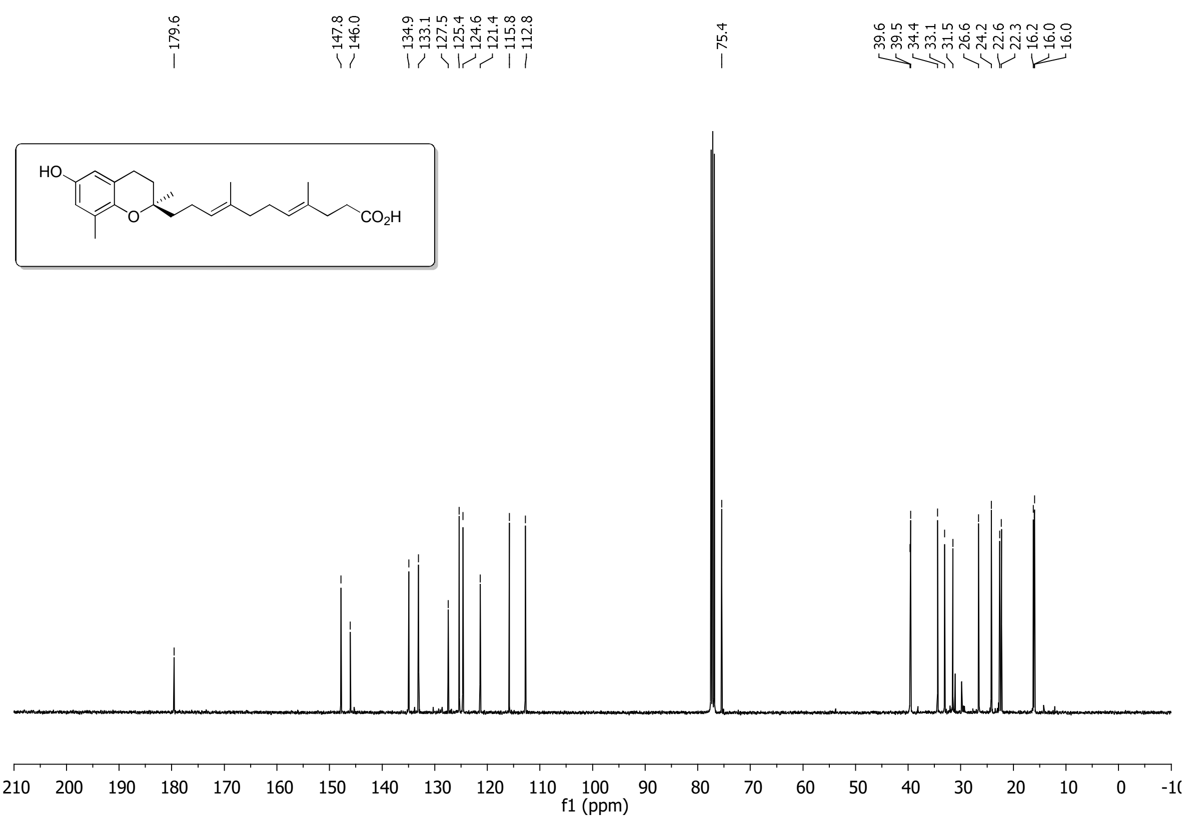

Figure S21. ^{1}H and ^{13}C NMR spectra of 15b in $CDCl_3$

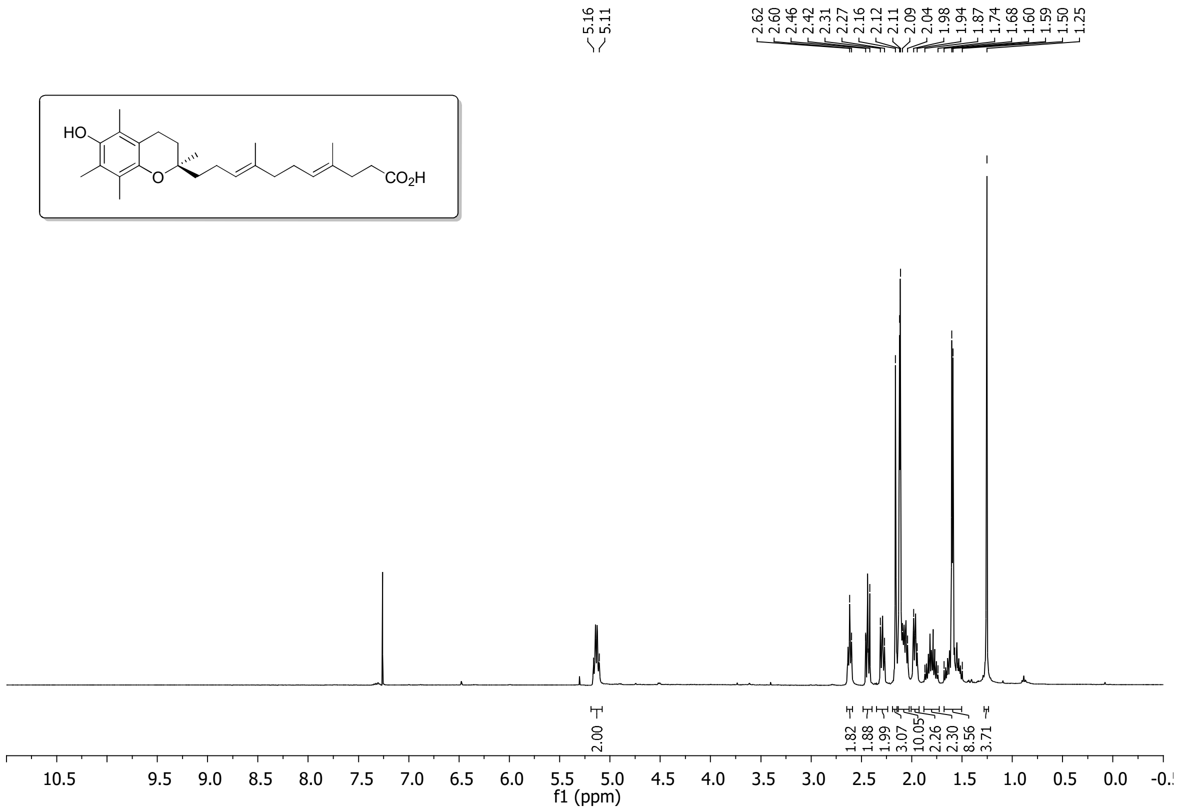

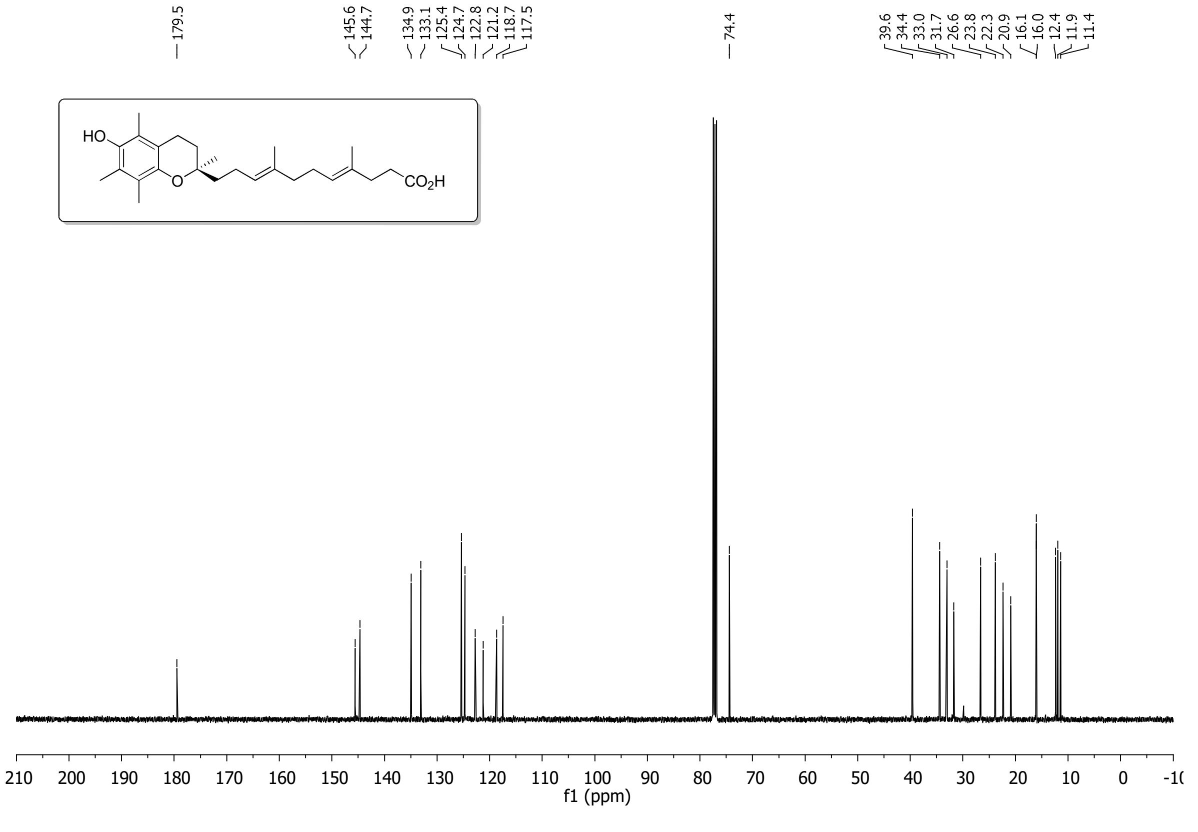

Figure S22. **^{1}H and ^{13}C NMR spectra of 15a in $CDCl_3$**

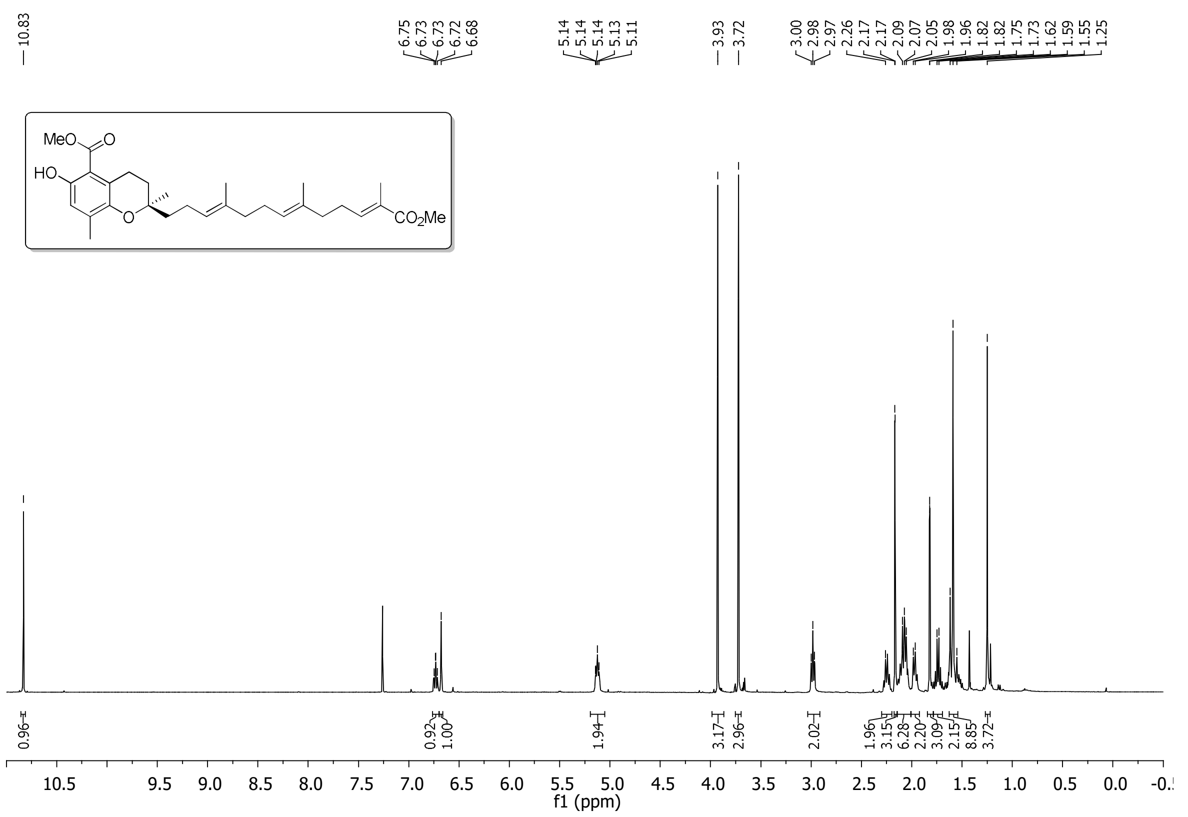

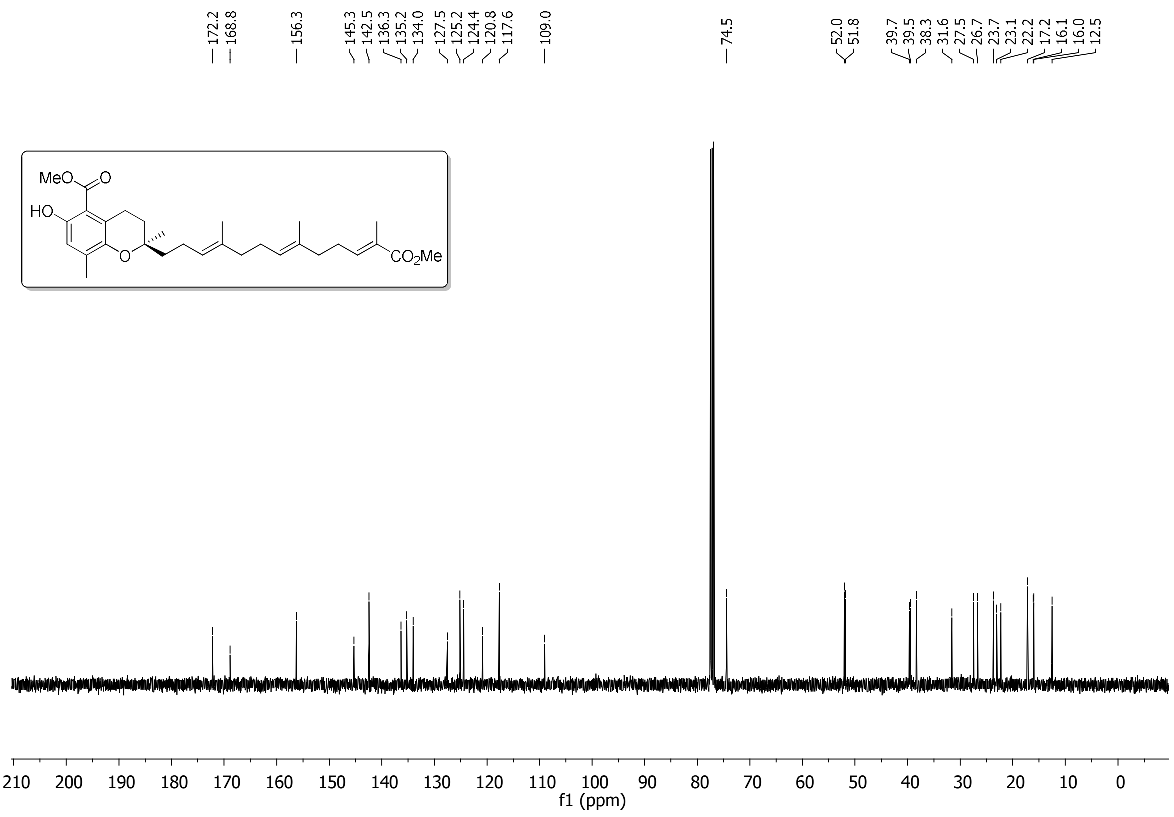

Figure S23. ^{1}H **and** ^{13}C **NMR spectra of 25 in** $CDCl_3$

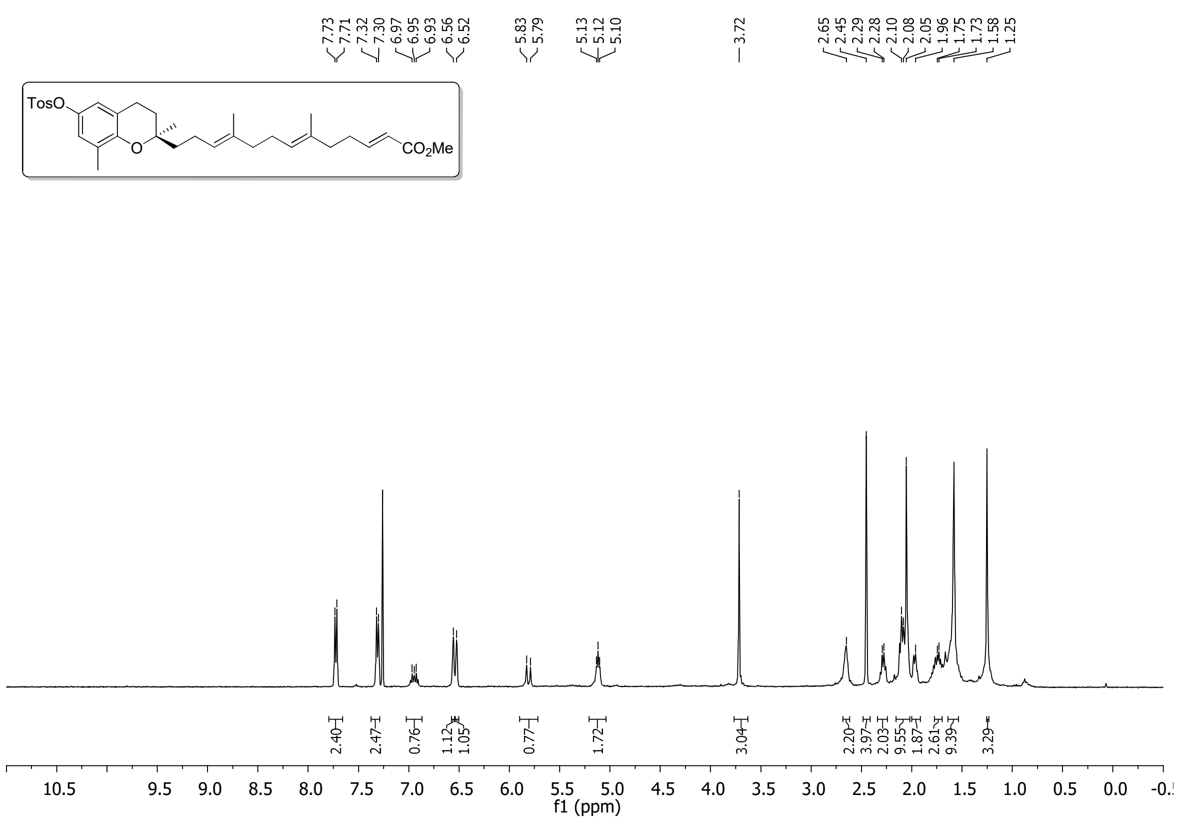

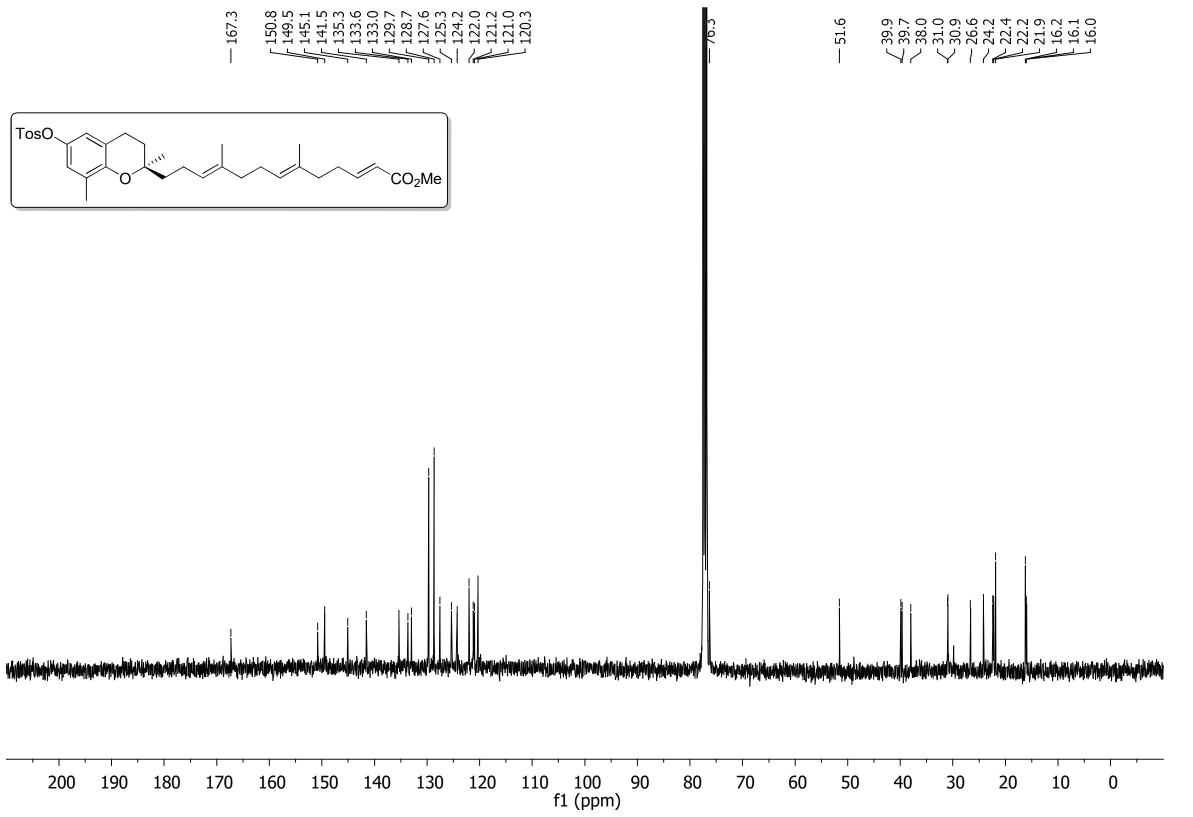

Figure S24. ^{1}H and ^{13}C NMR spectra of **58** in $CDCl_3$

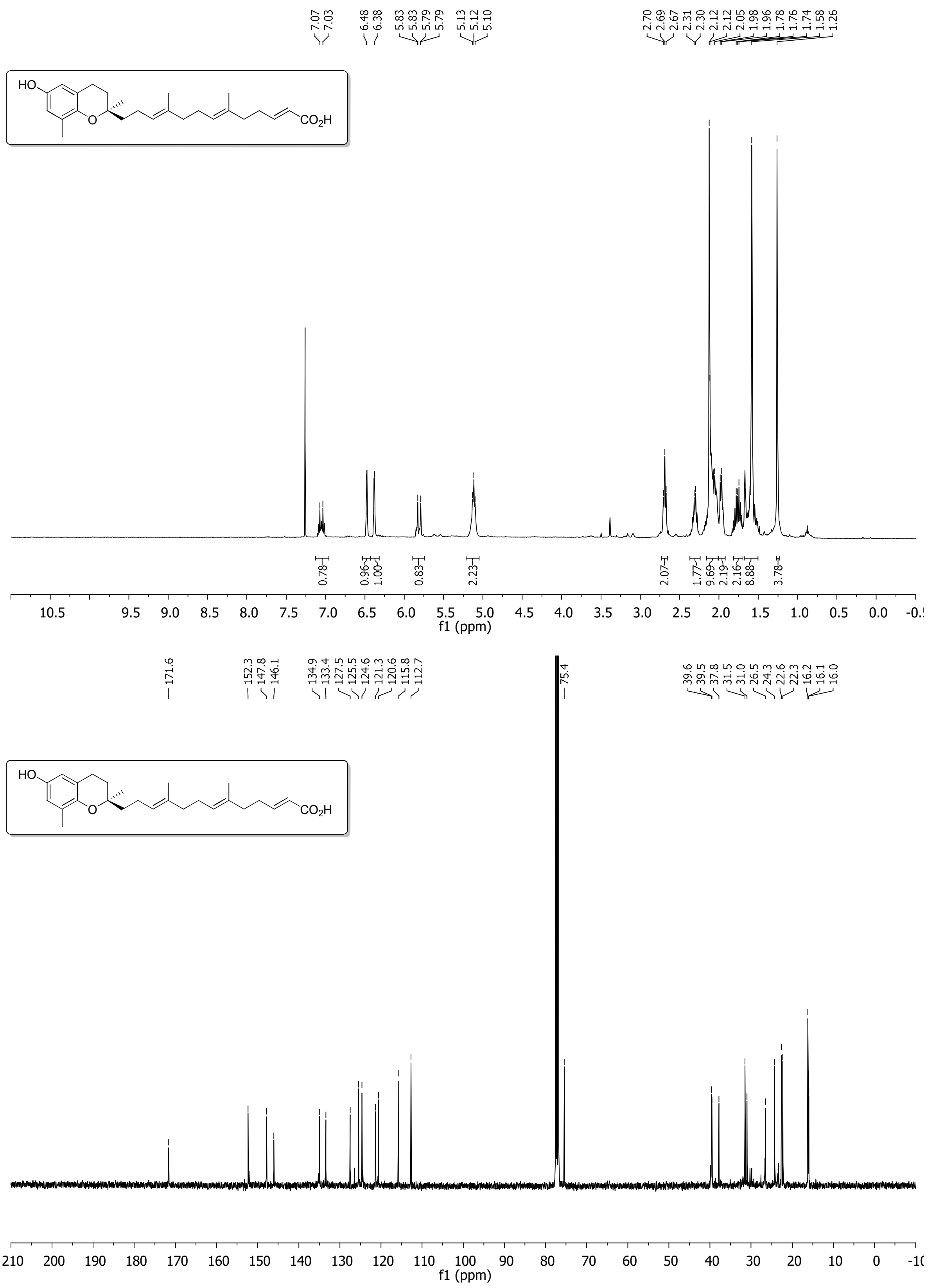

Figure S25. **^{1}H and ^{13}C NMR spectra of 14 in $CDCl_3$**

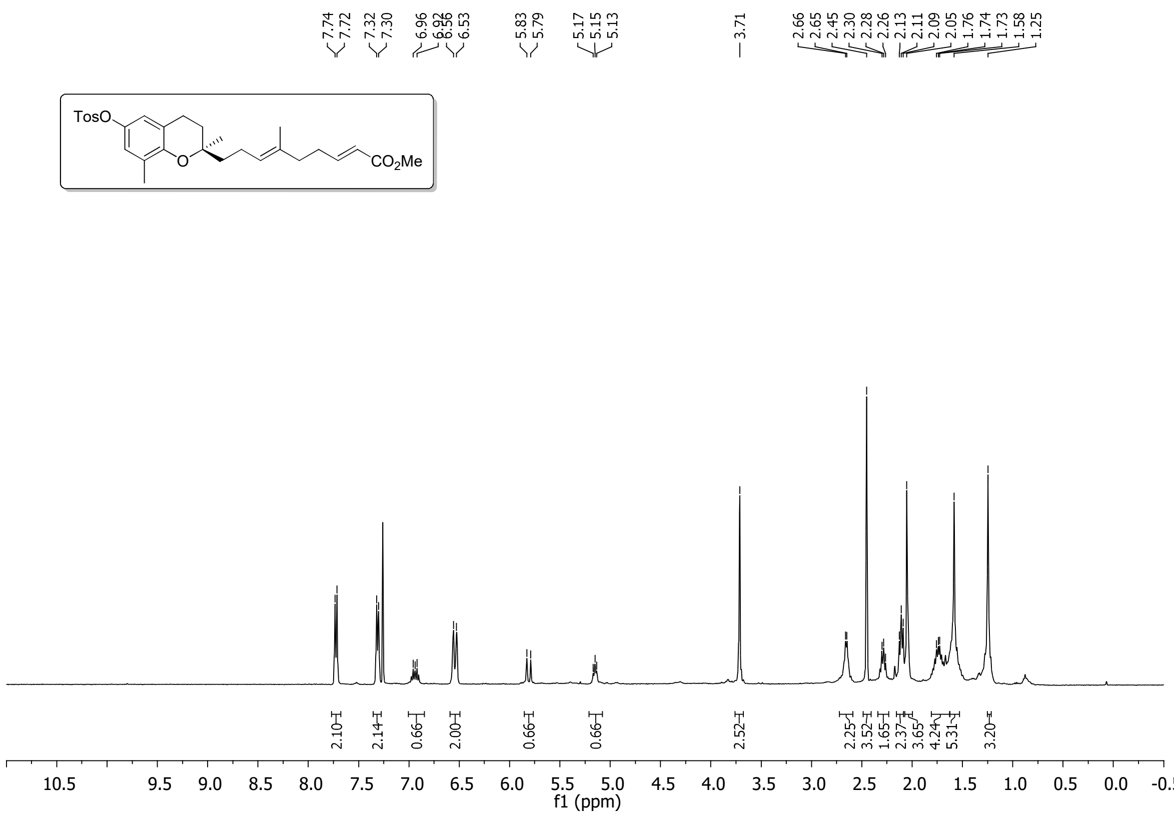

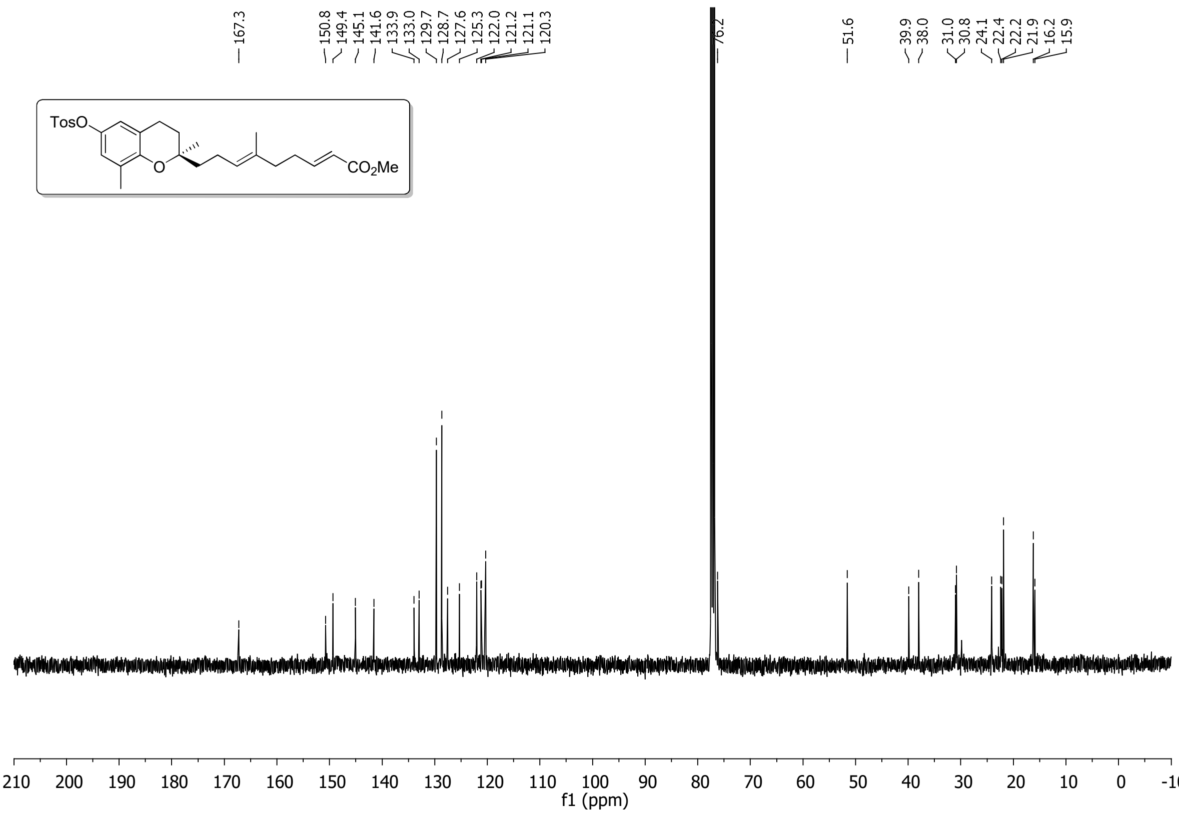

Figure S26. ^1H **and** ^{13}C **NMR spectra of 59 in** $CDCl_3$

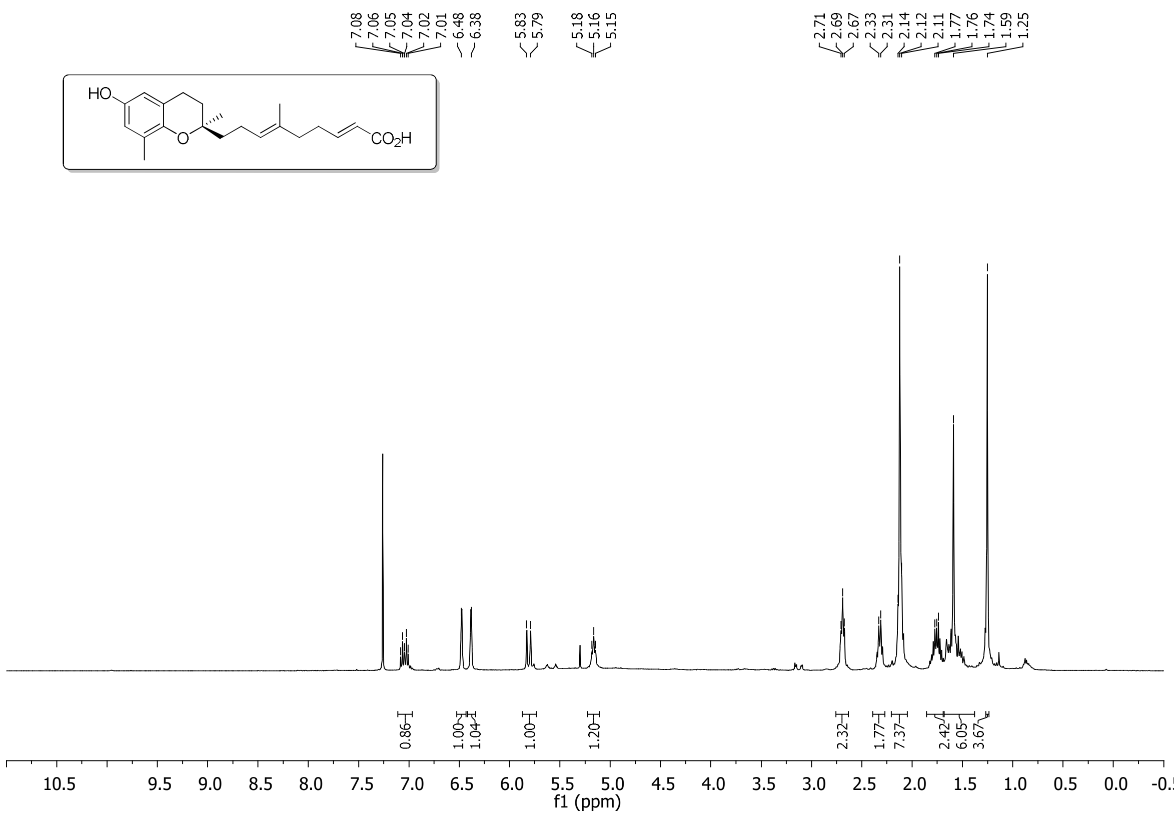

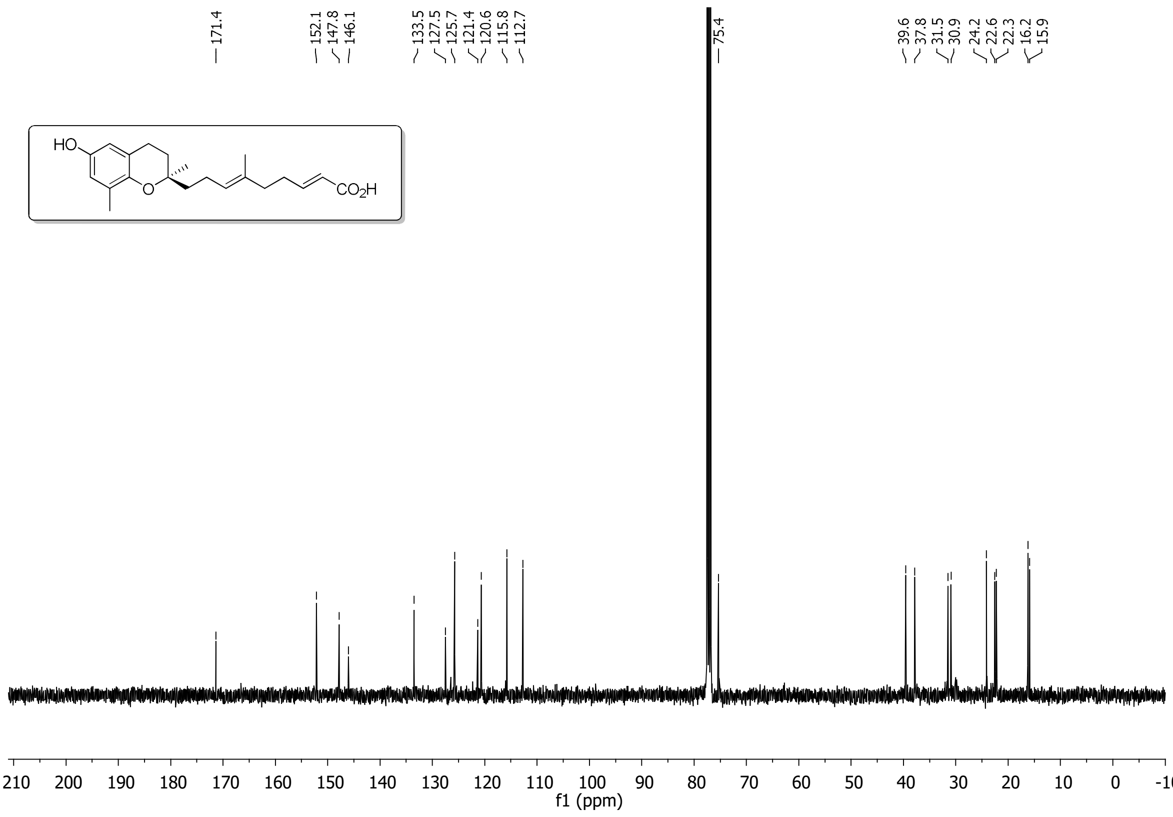

Figure S27. ^{1}H **and** ^{13}C **NMR spectra of 16 in** $CDCl_3$

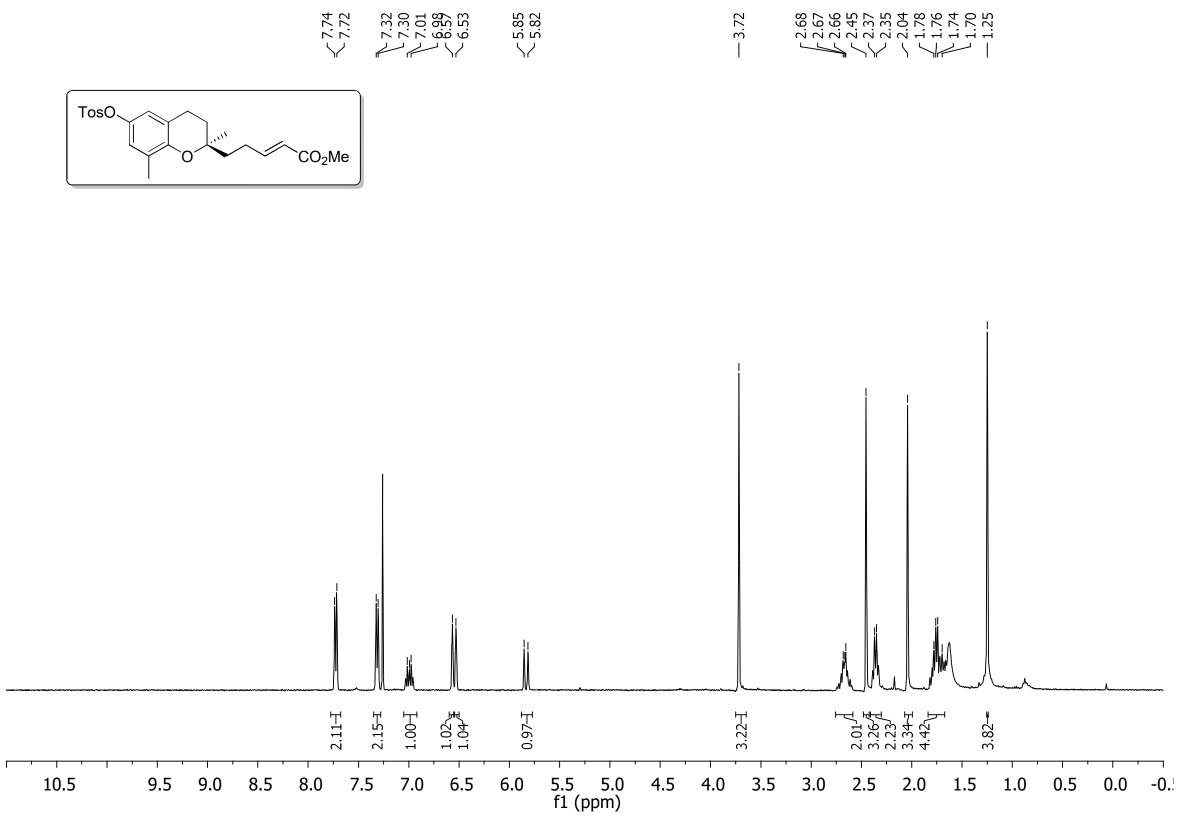

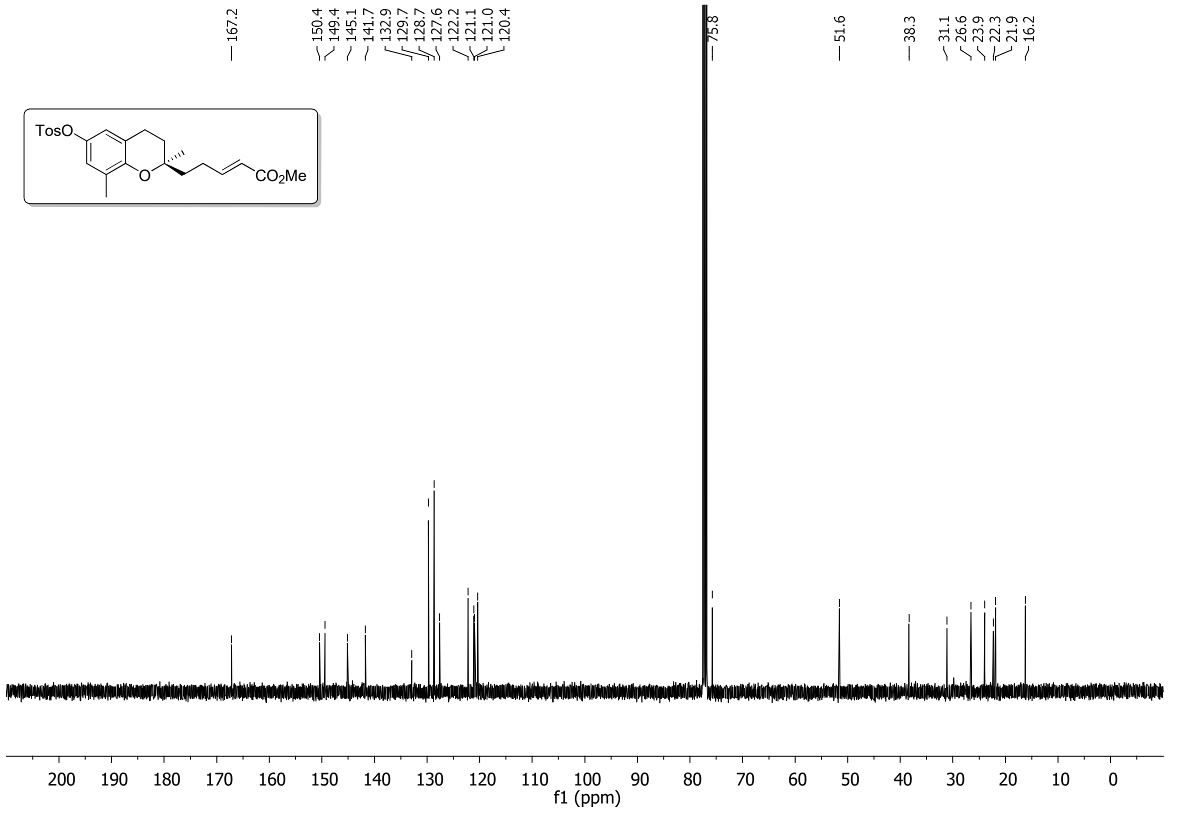

Figure S28. ^{1}H and ^{13}C NMR spectra of 60 in $CDCl_3$

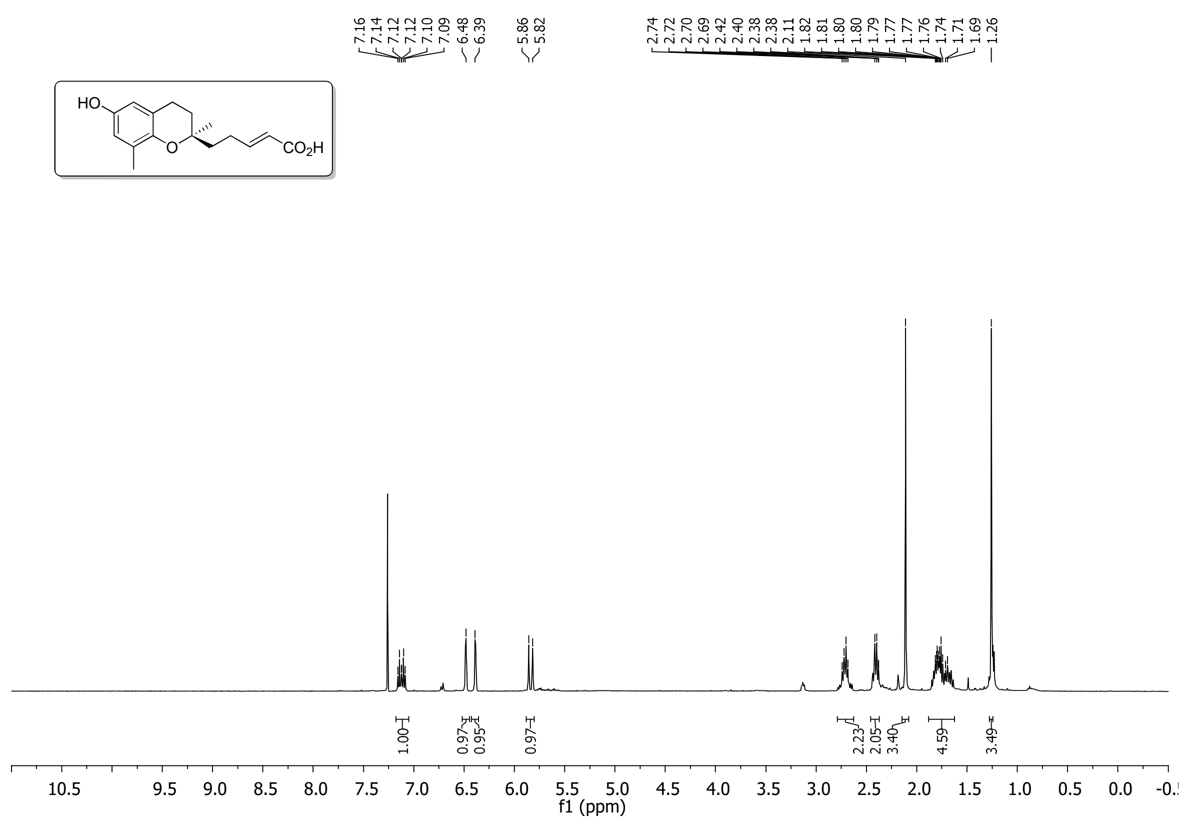

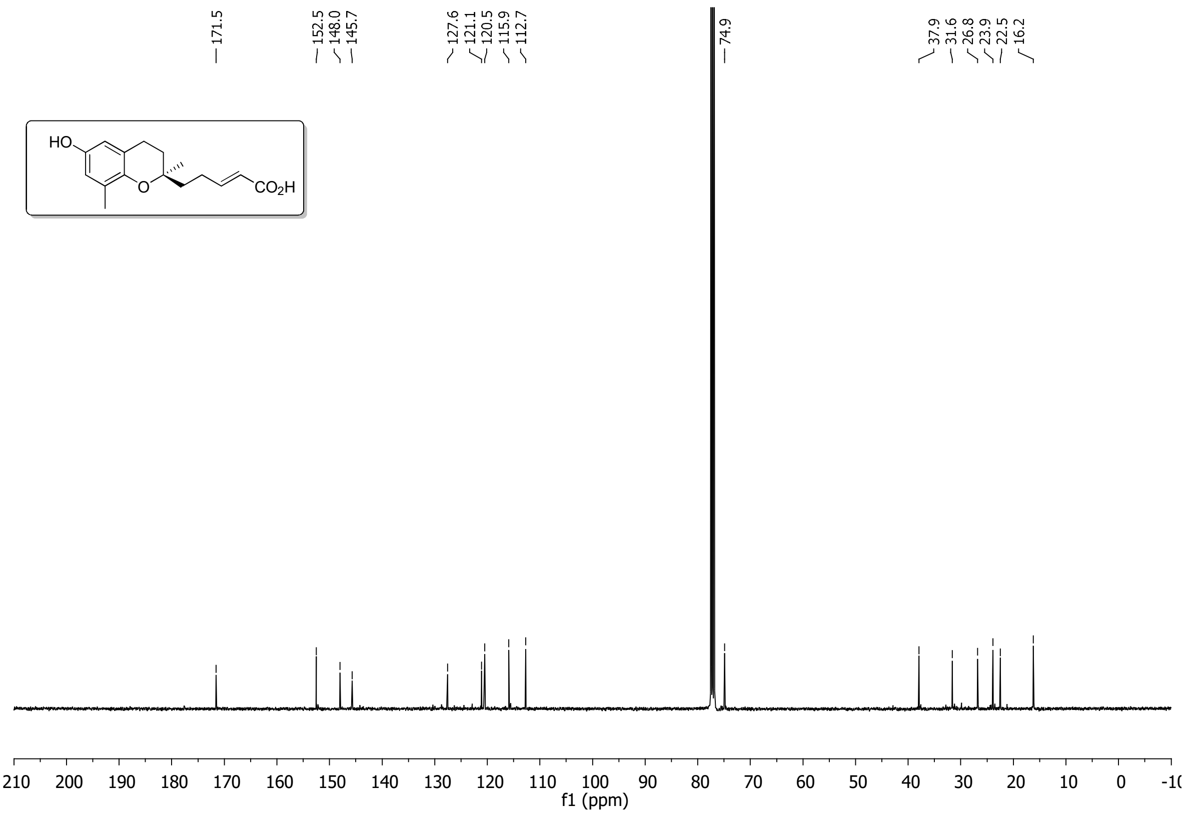

Figure S29. ^{1}H **and** ^{13}C **NMR spectra of 17 in** $CDCl_3$

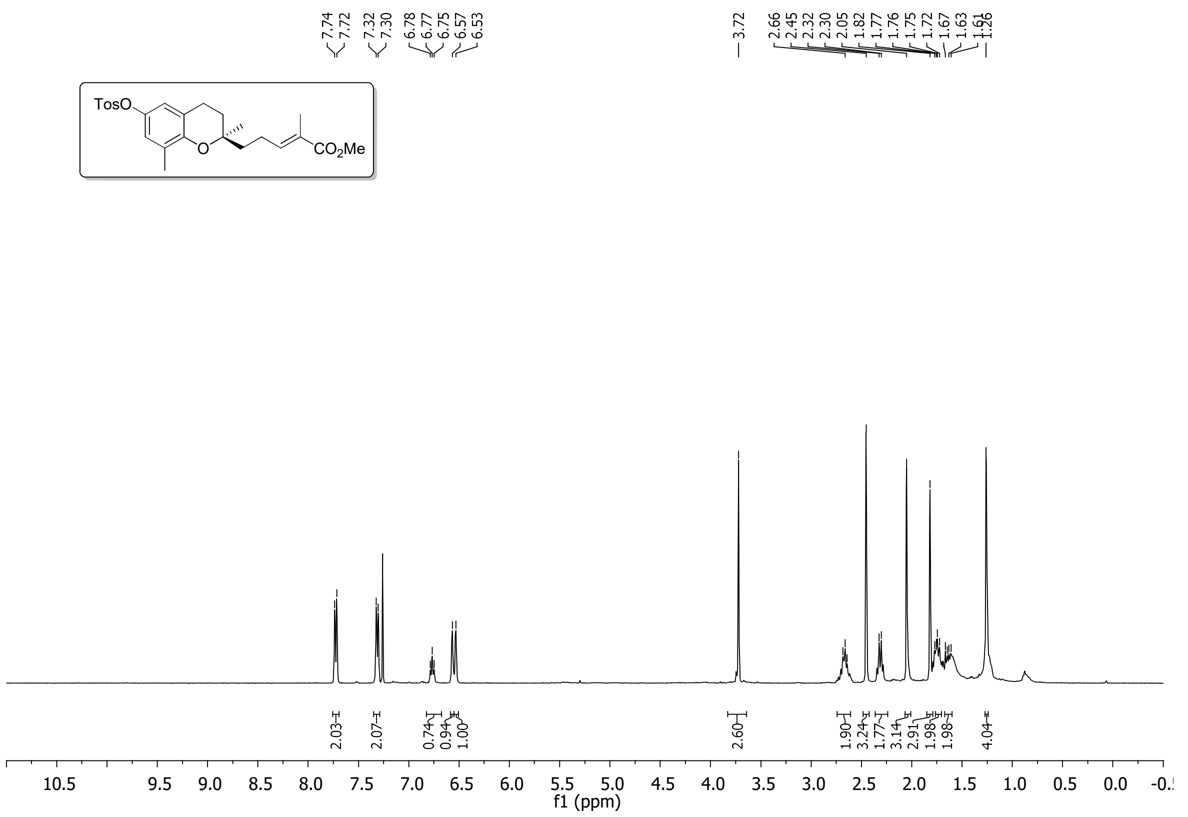

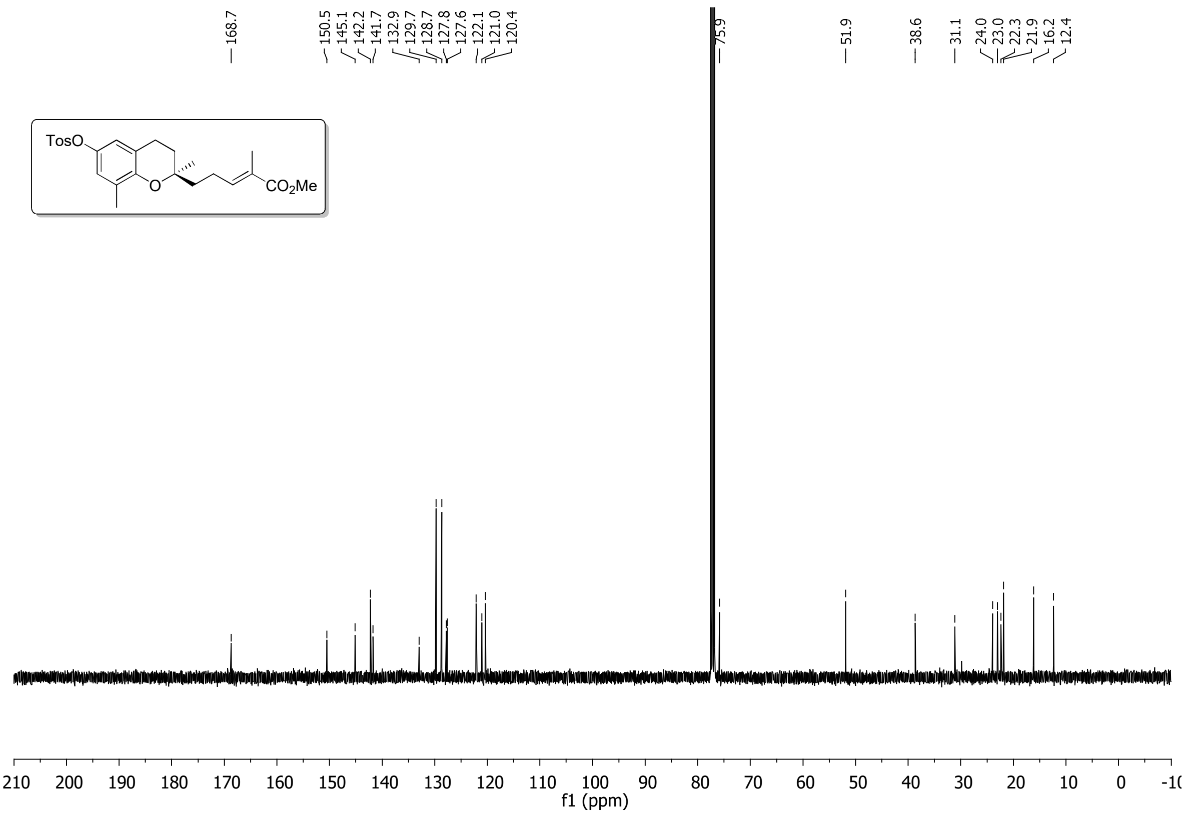

Figure S30. ^{1}H **and** ^{13}C **NMR spectra of 61 in** $CDCl_3$

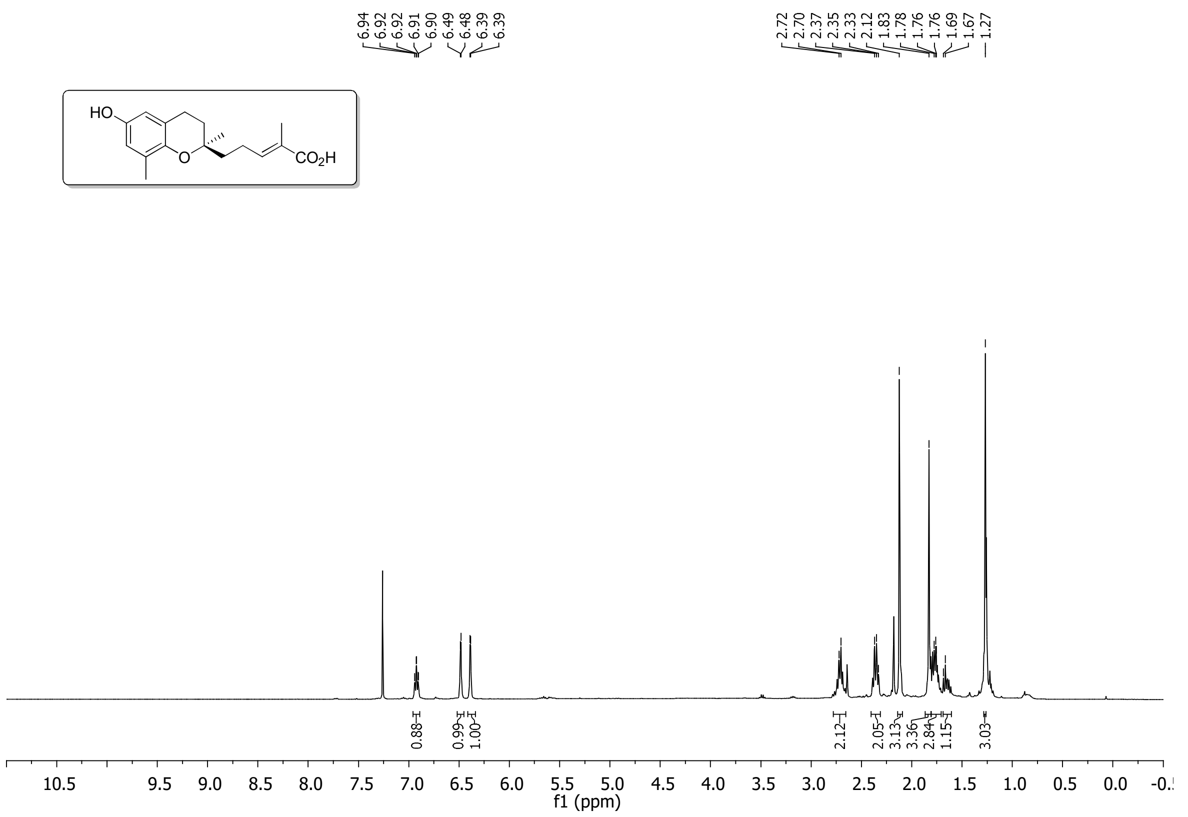

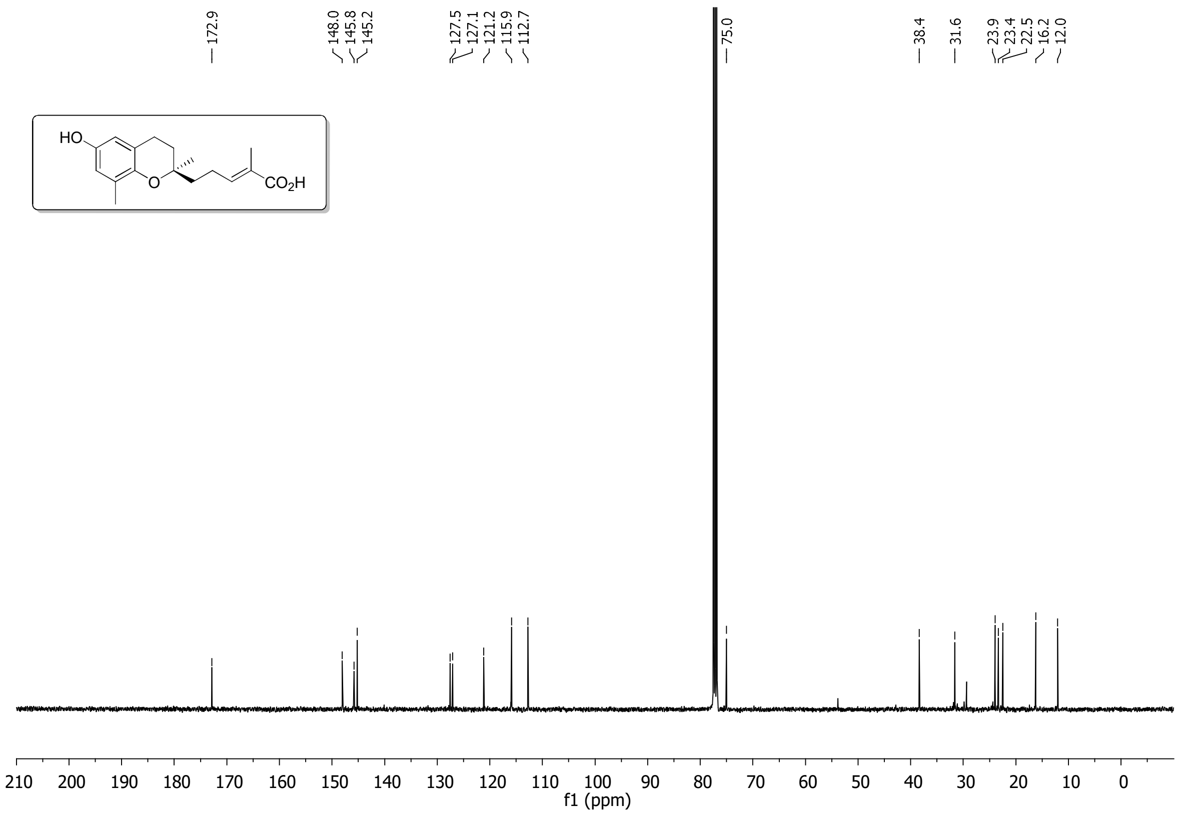

Figure S31. ^{1}H and ^{13}C NMR spectra of **18** in $CDCl_3$

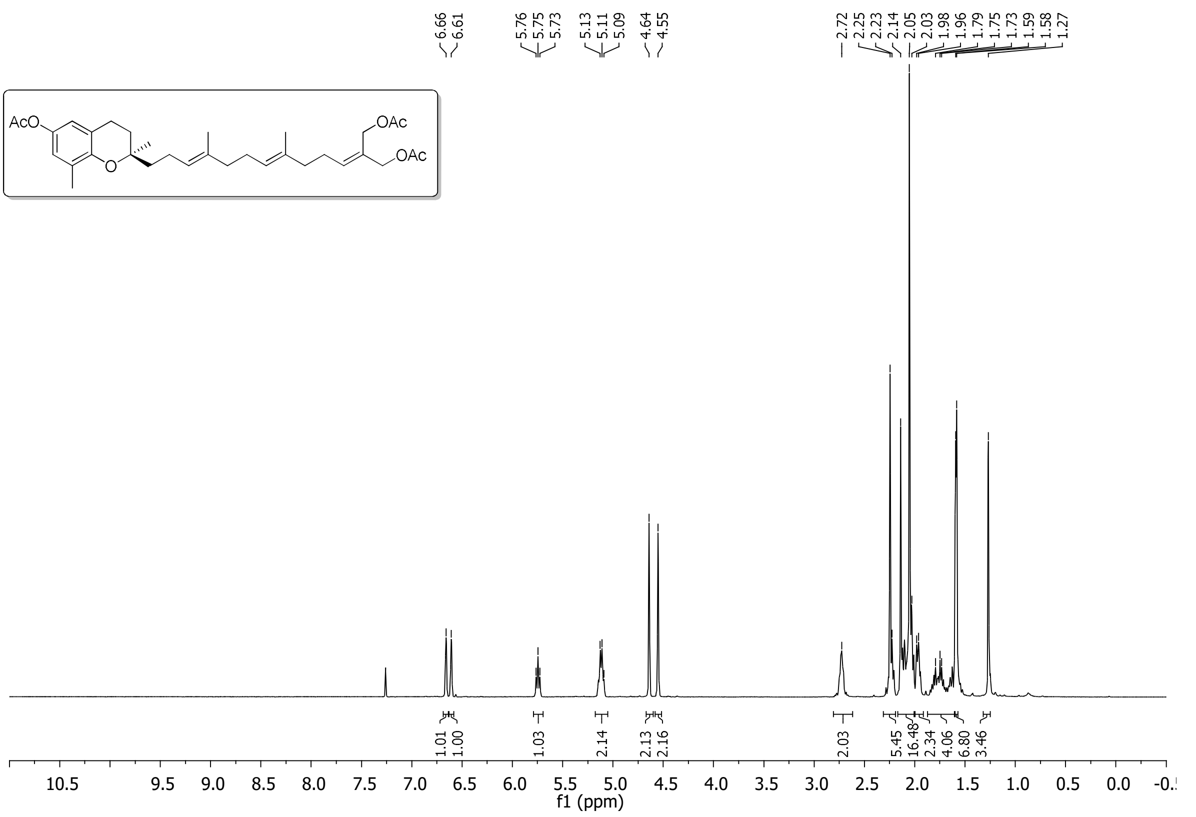

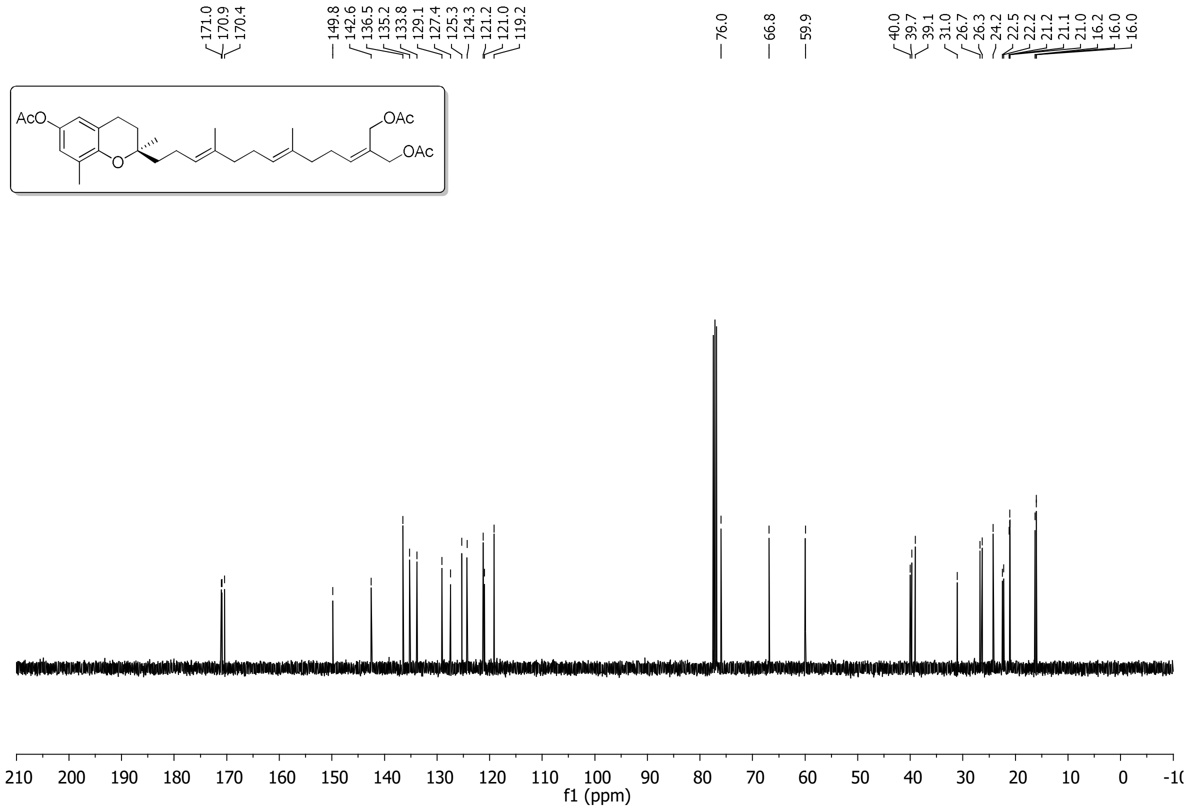

Figure S32. ^{1}H **and** ^{13}C **NMR spectra of 65 in** $CDCl_3$

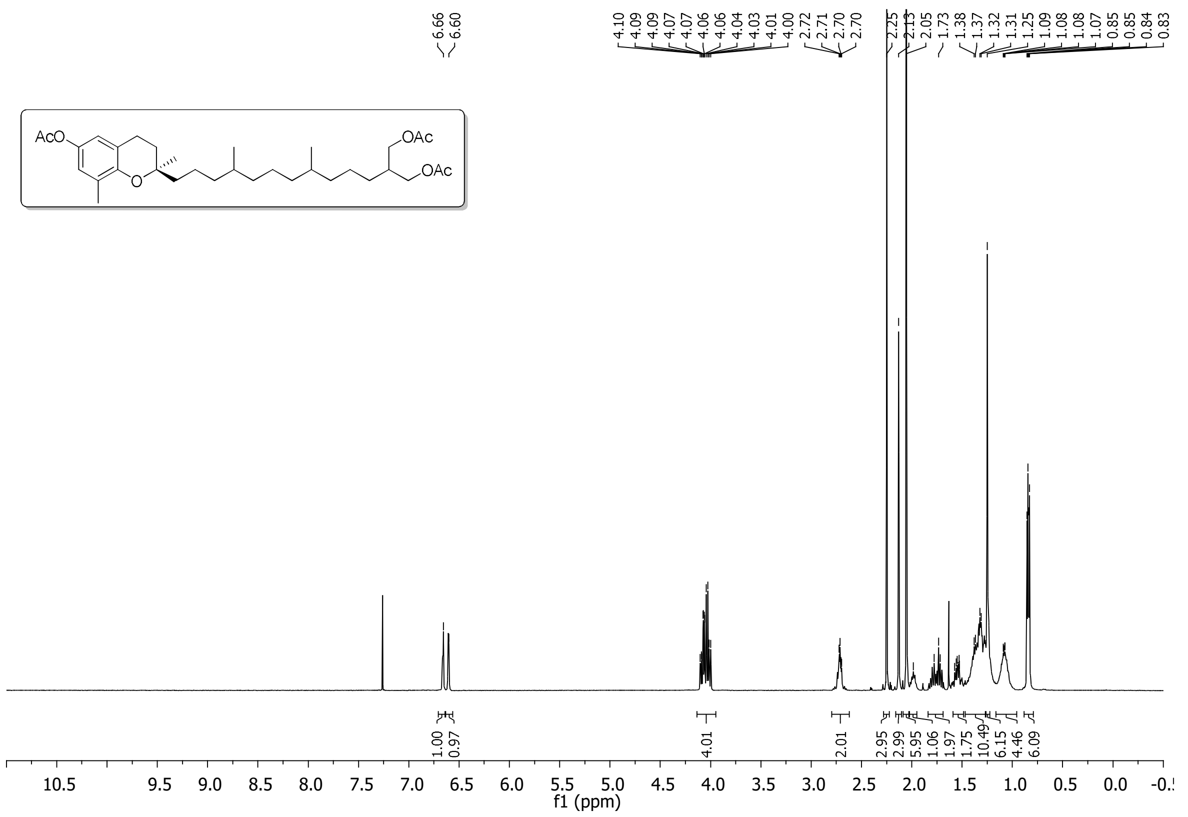

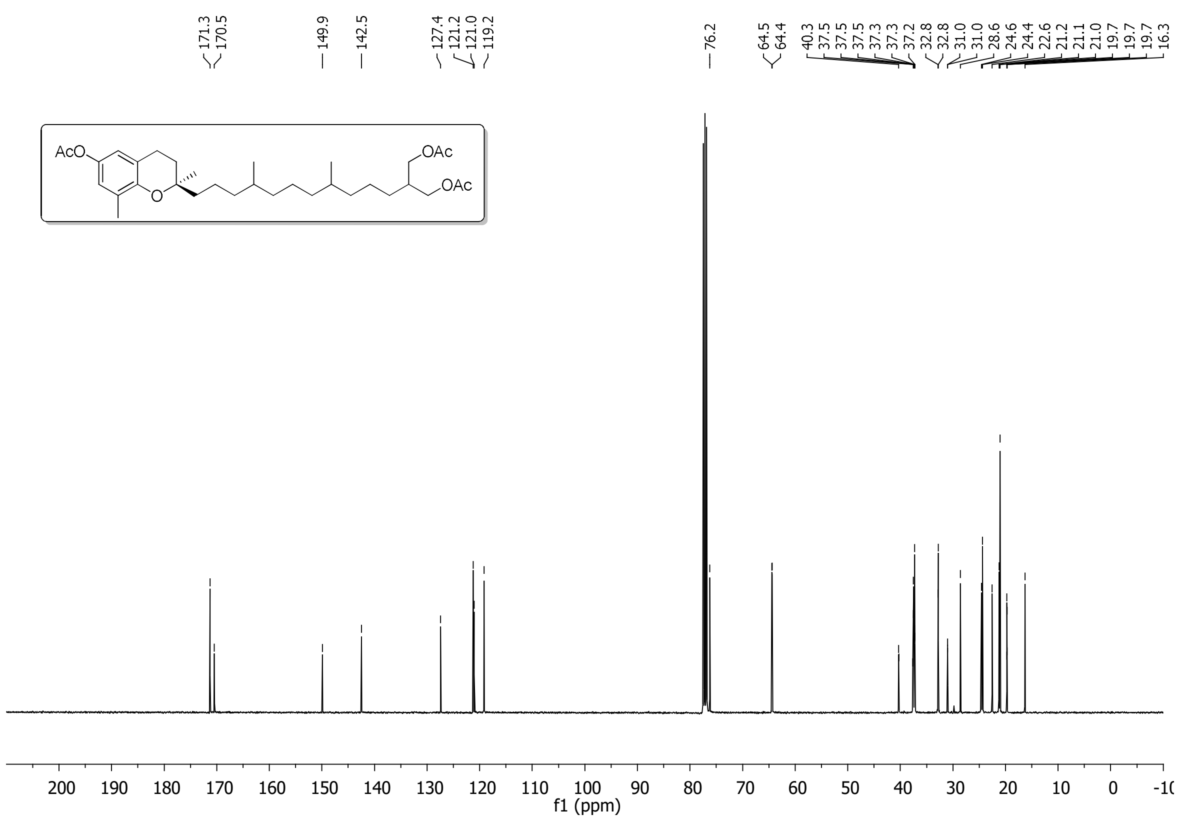

Figure S33. ^{1}H and ^{13}C NMR spectra of 66 in $CDCl_3$

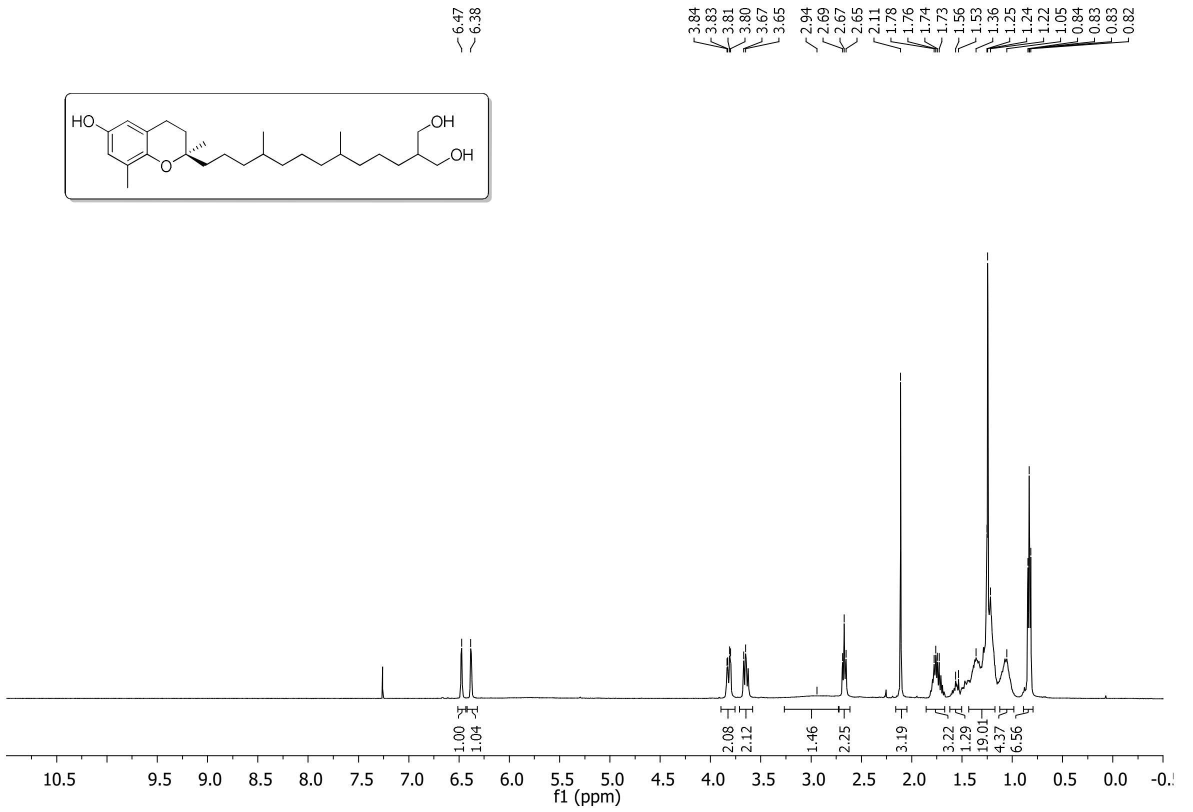

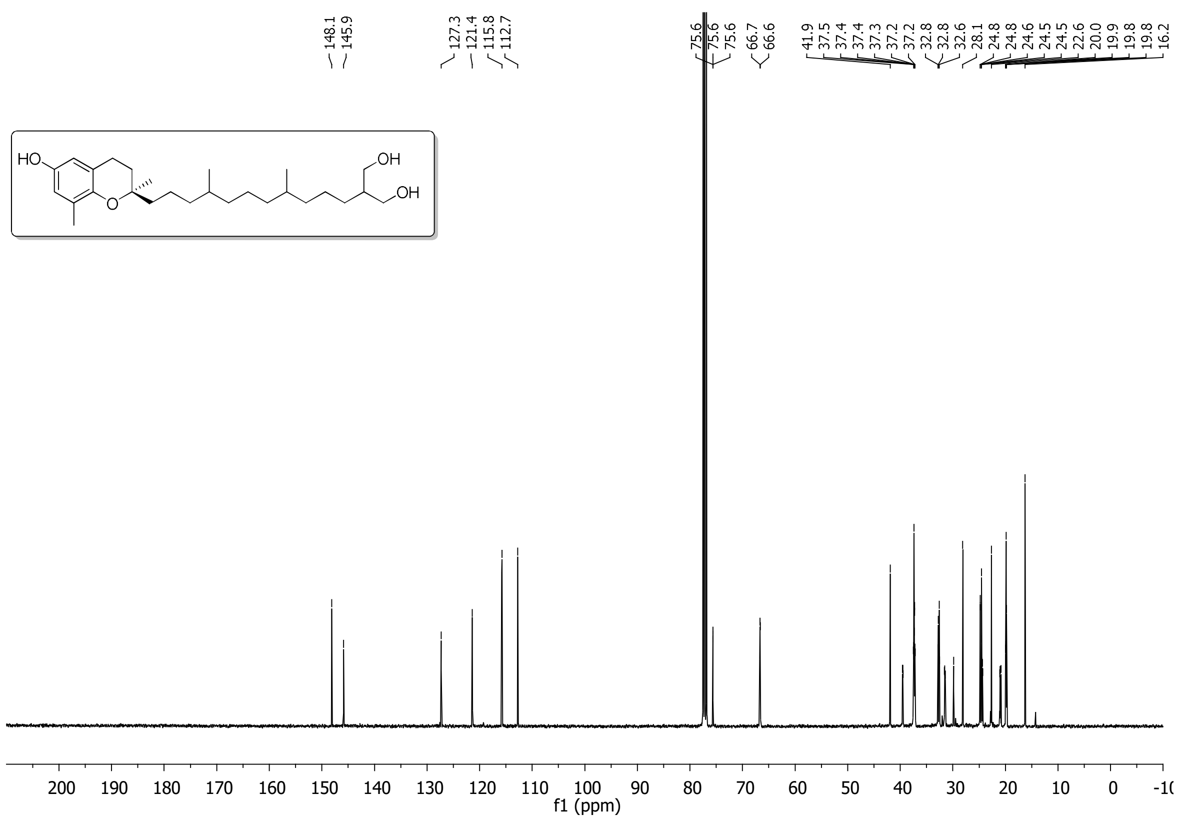

Figure S34. ^{1}H and ^{13}C NMR spectra of 26 in $CDCl_3$

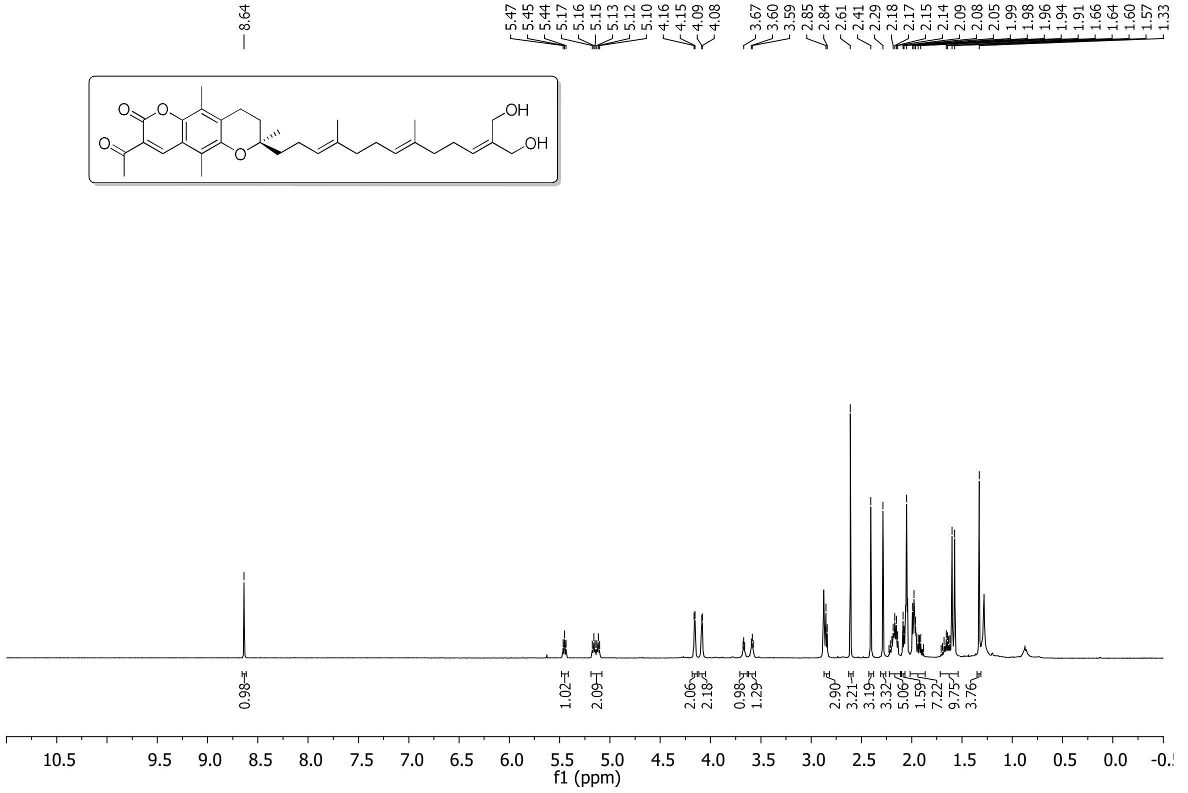

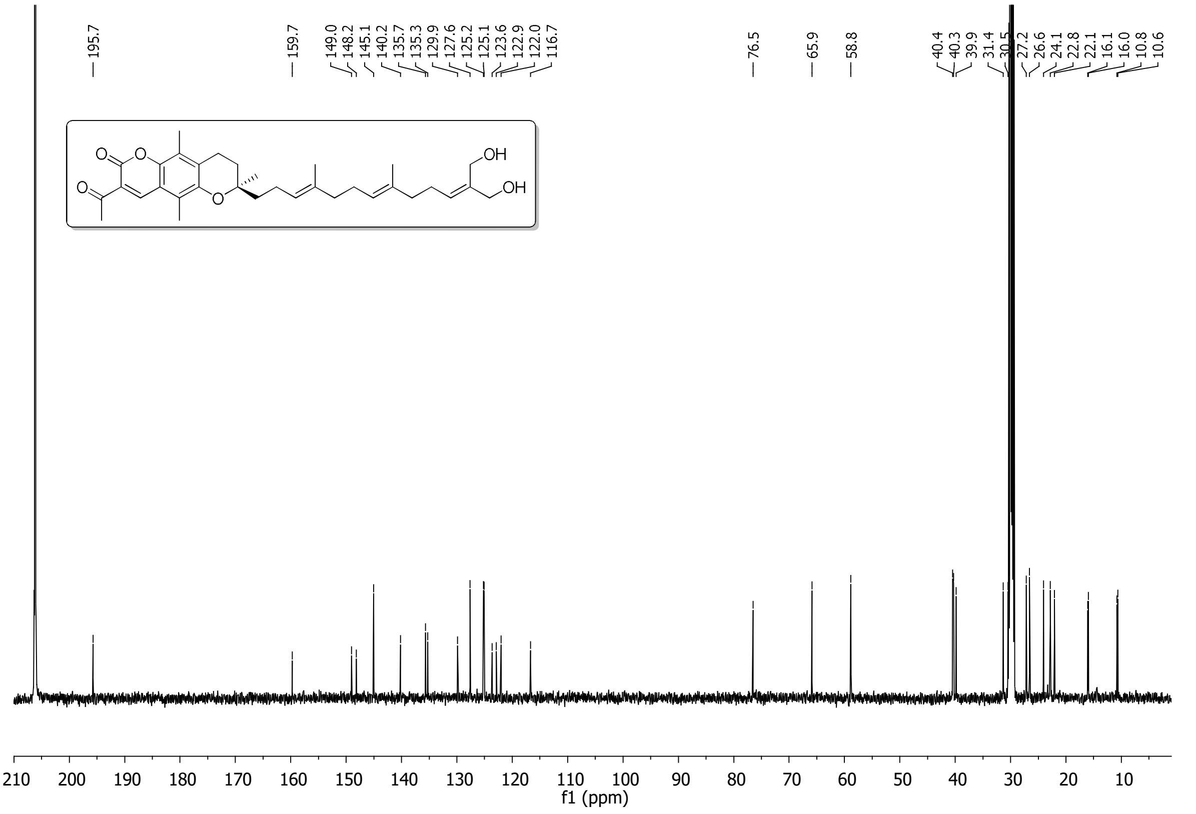

Figure S35. ^{1}H **and** ^{13}C **NMR spectra of 41 in acetone-*d*$_6$**

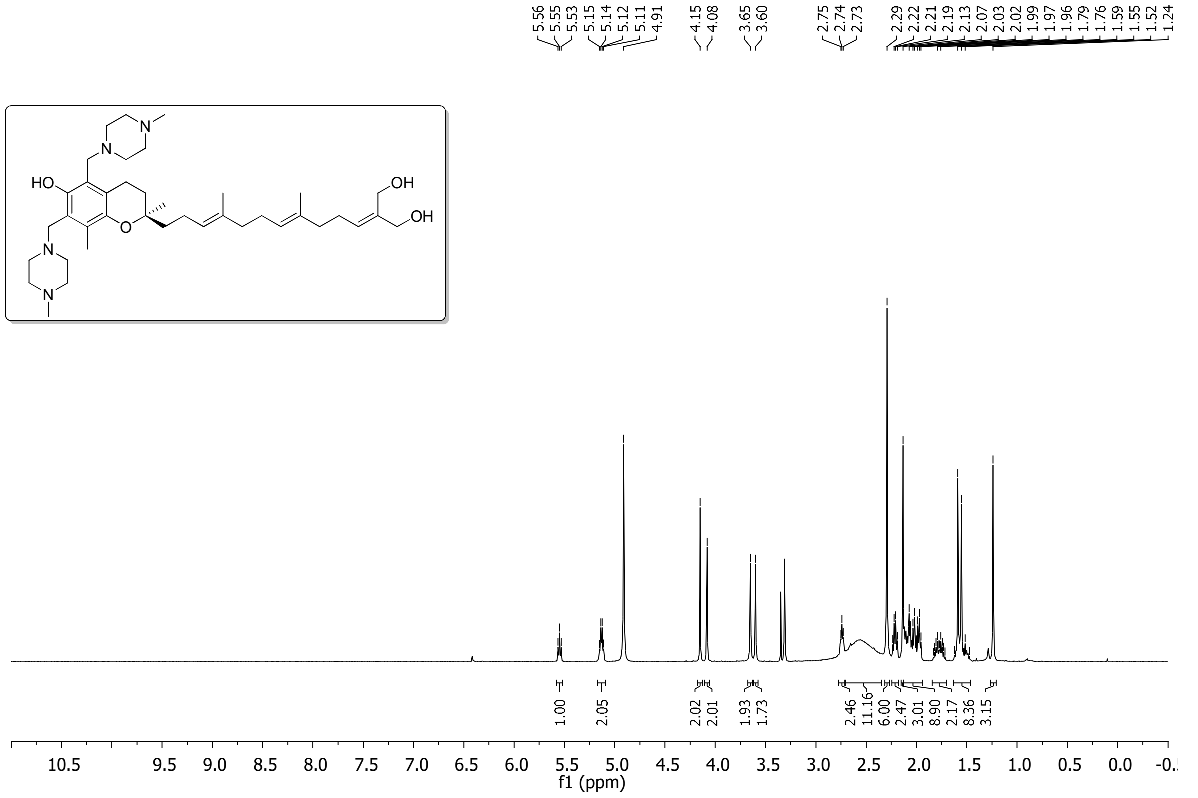

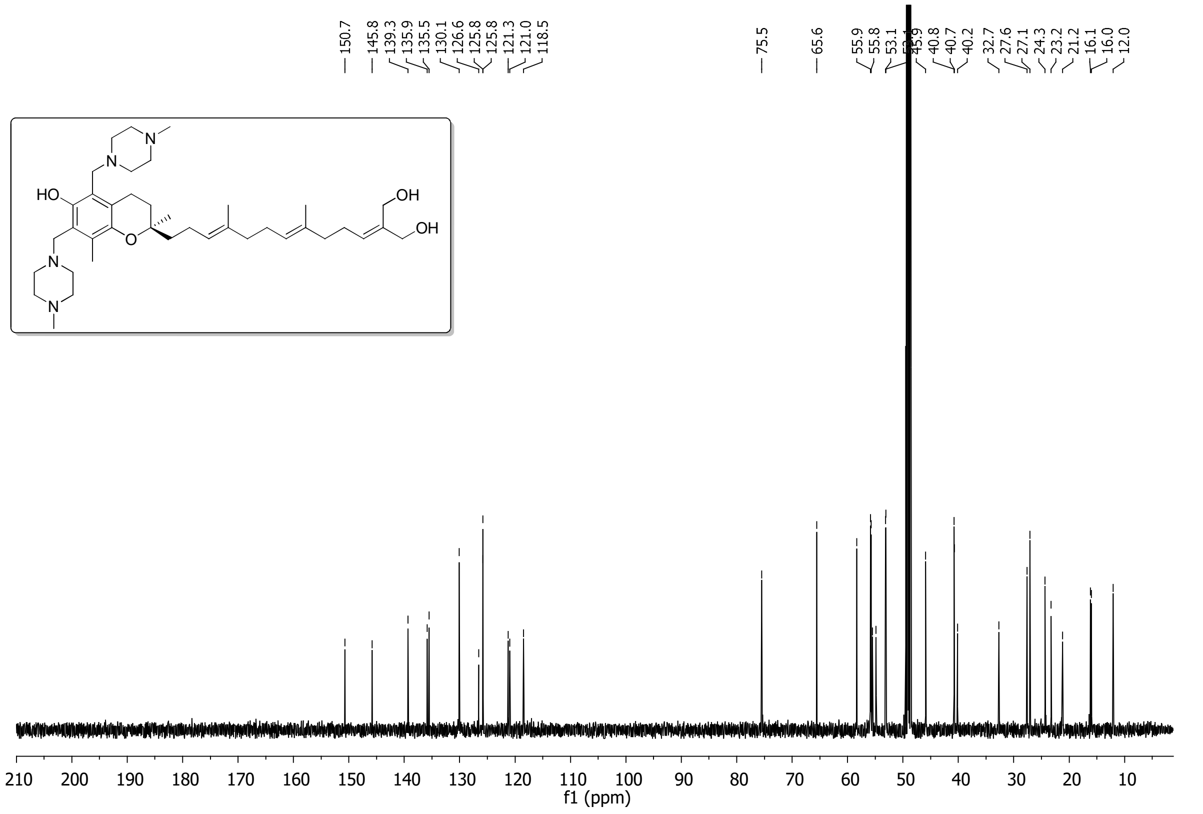

Figure S36. ^{1}H and ^{13}C NMR spectra of 40 in methanol-d_4

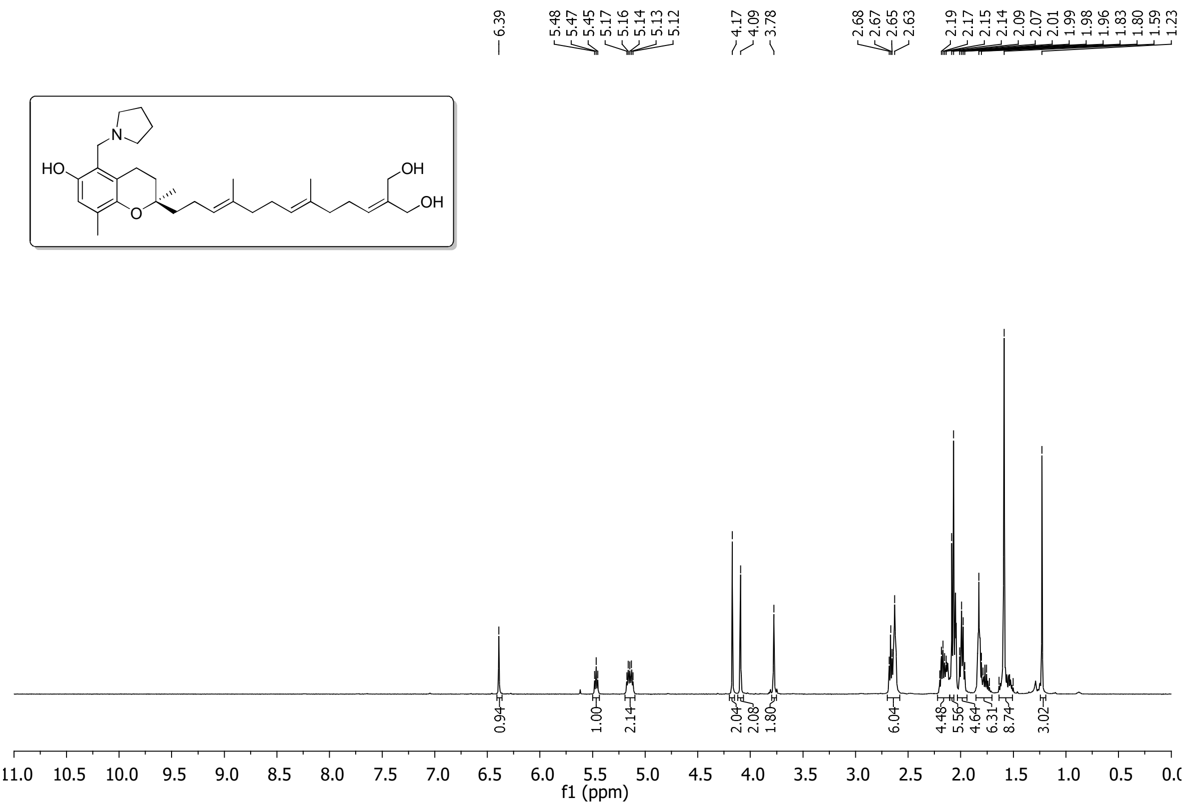

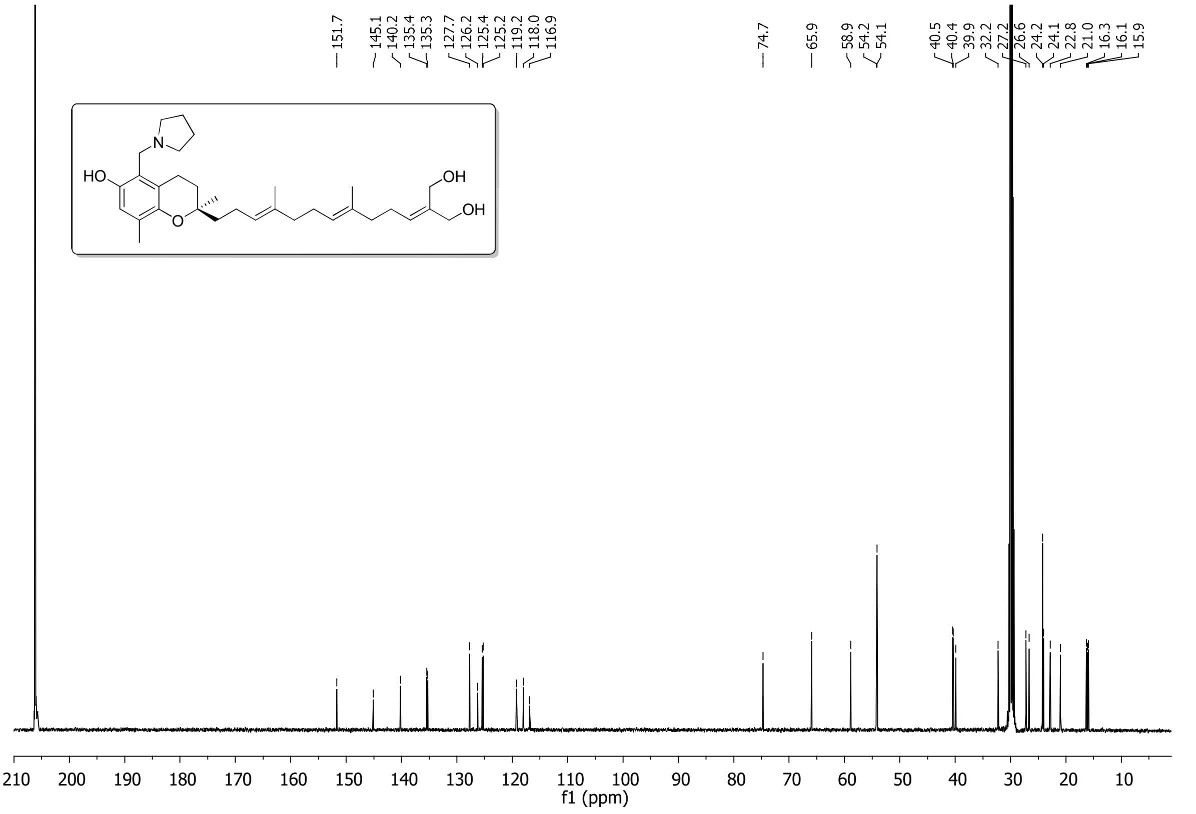

Figure S37. ^{1}H and ^{13}C NMR spectra of 35 in acetone-*d*₆

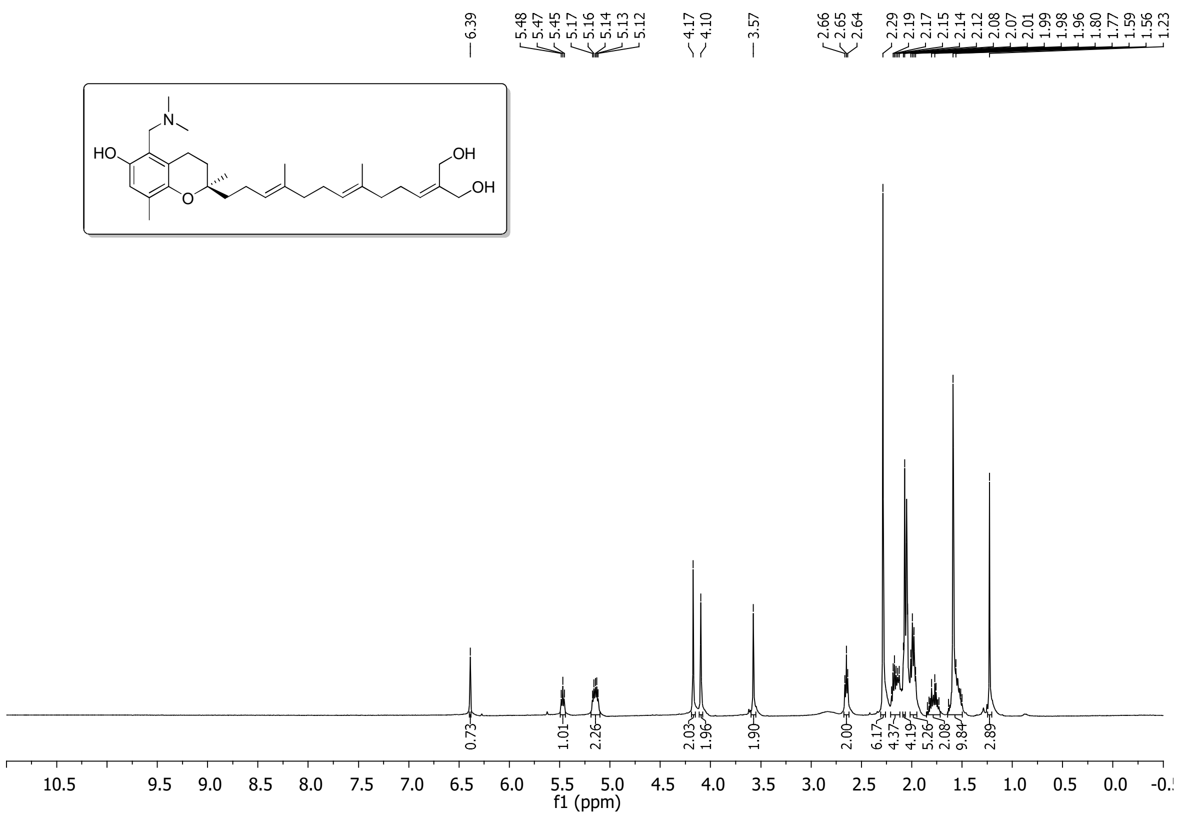

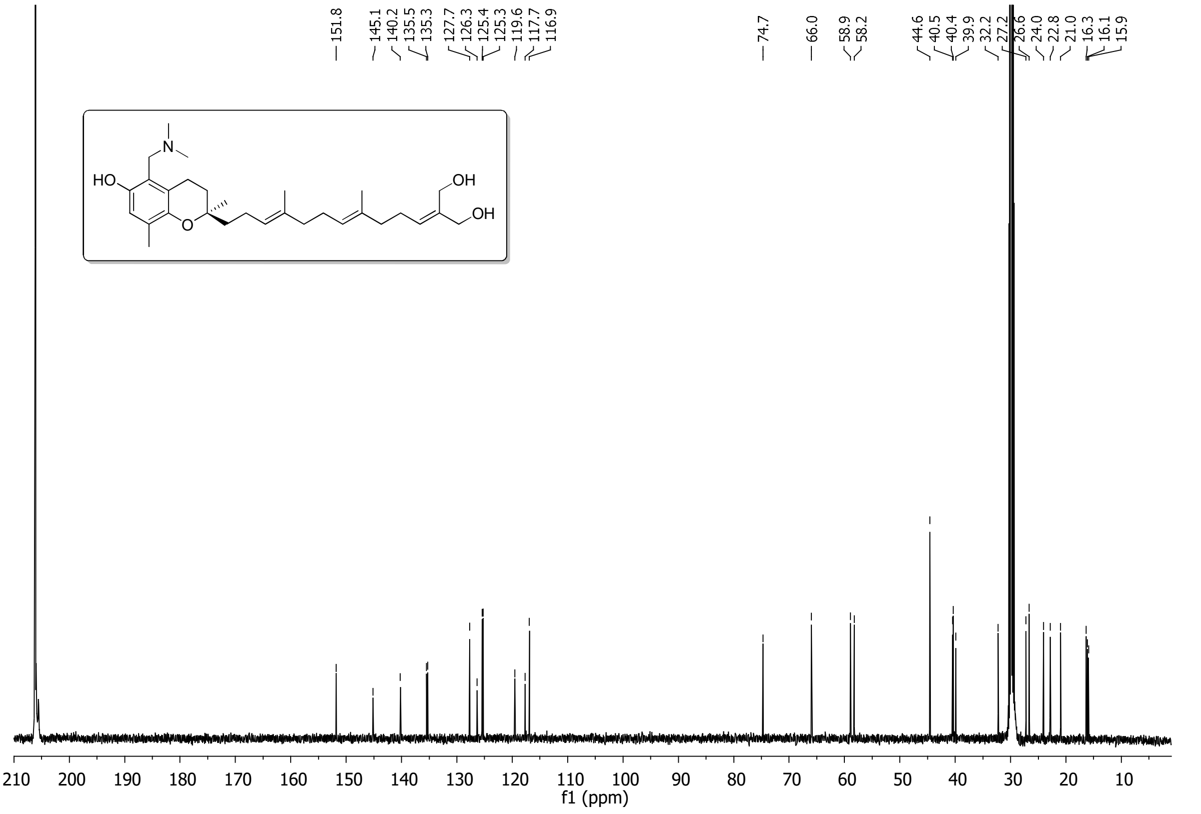

Figure S38. ^{1}H **and** ^{13}C **NMR spectra of 36 in acetone-*d*₆**

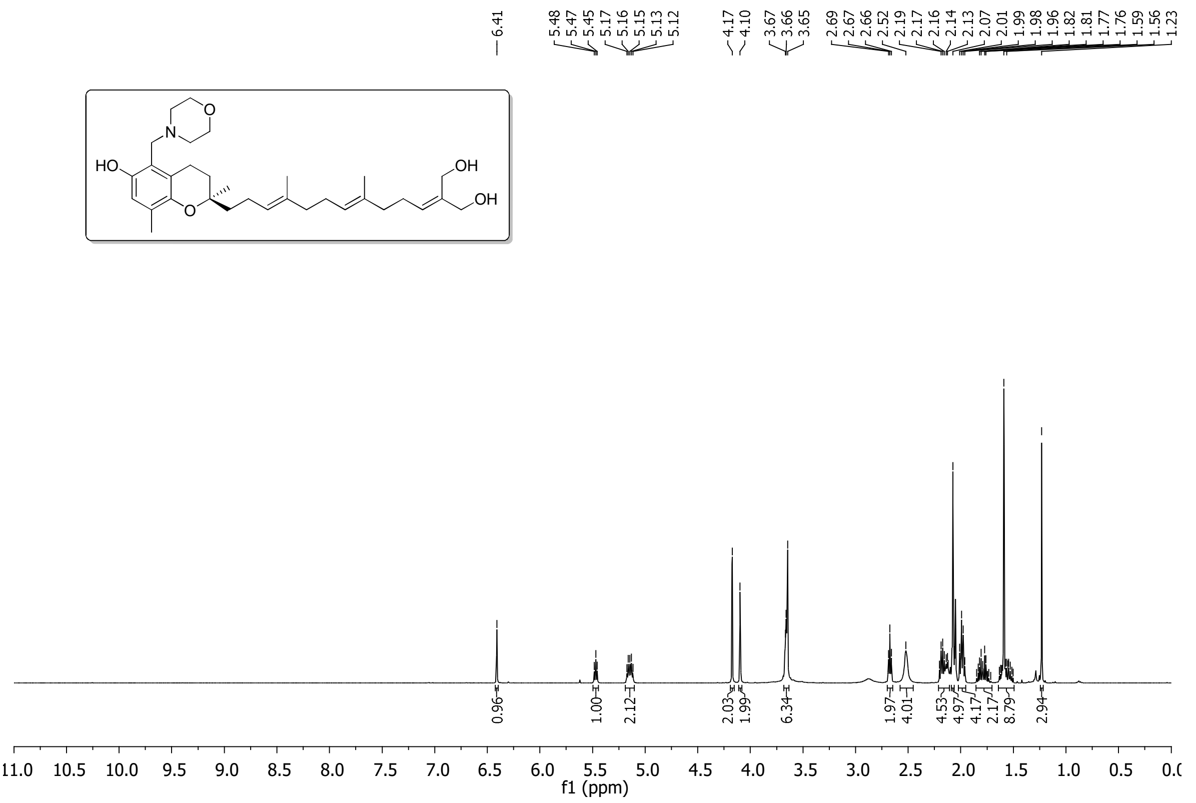

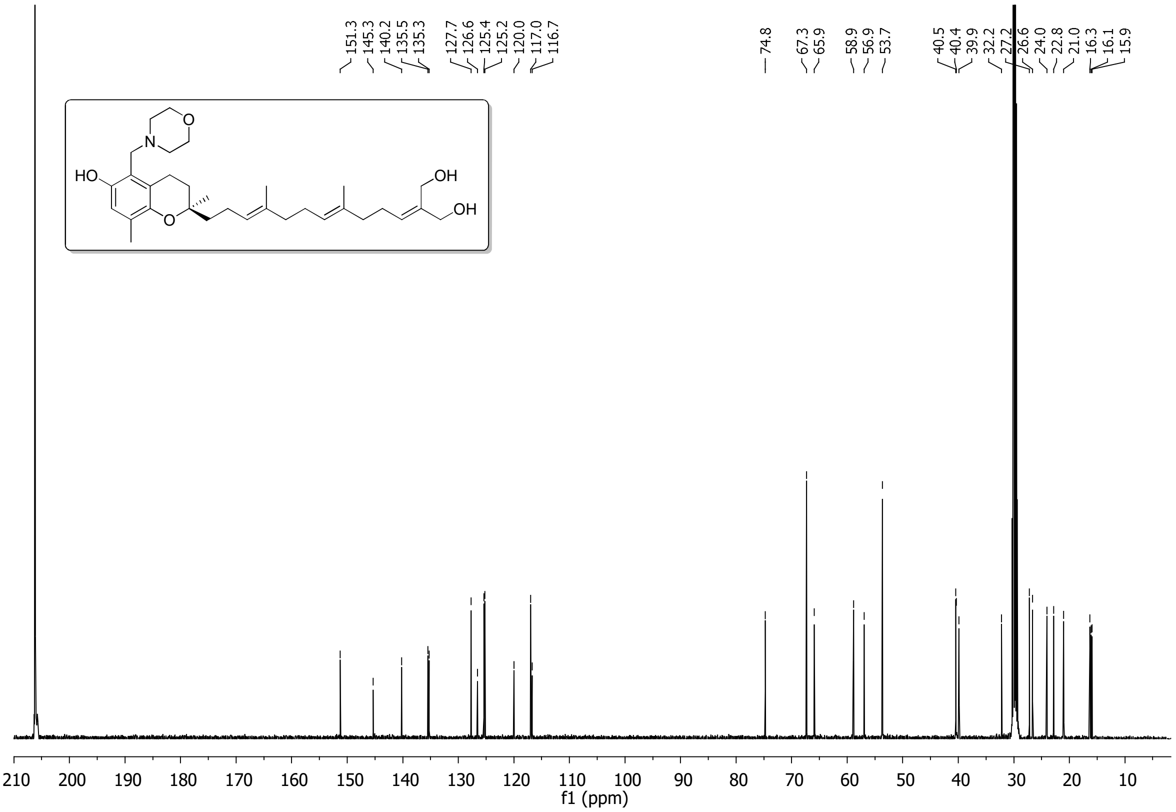

Figure S39. ^{1}H and ^{13}C NMR spectra of 37 in acetone-d_6

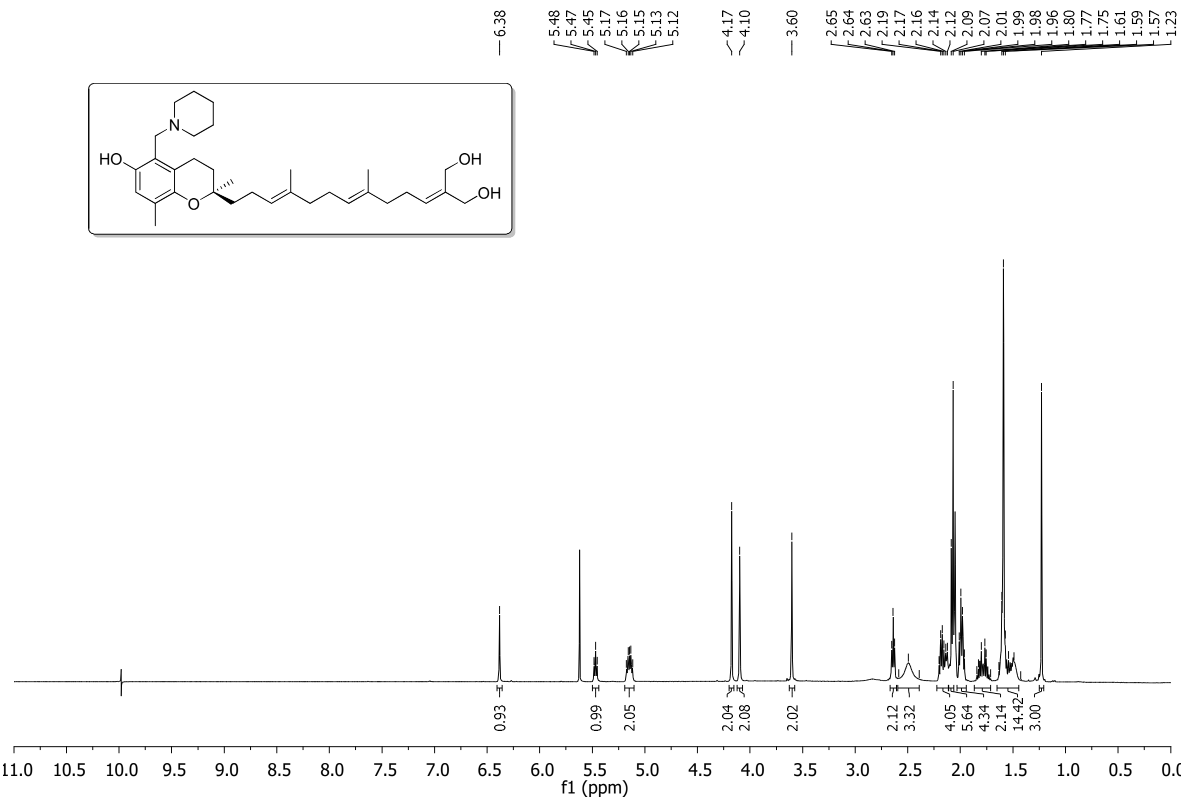

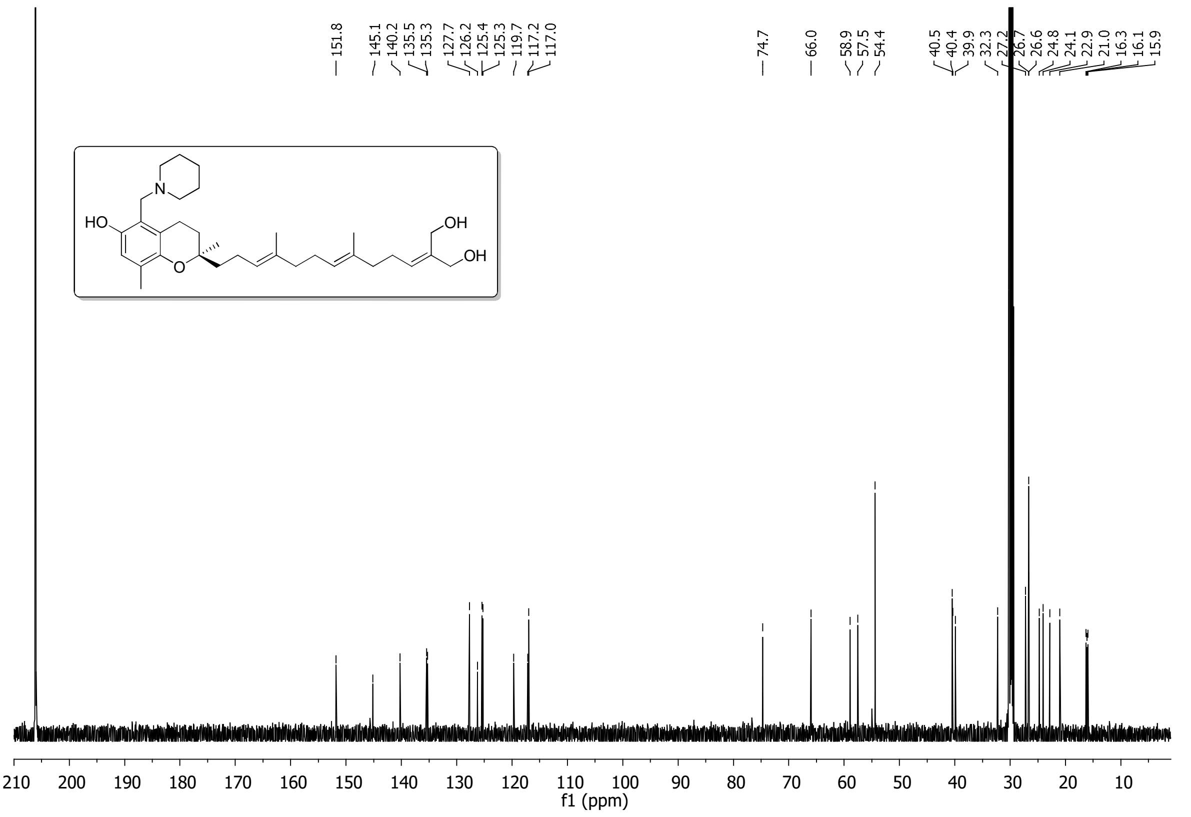

Figure S40. ^{1}H and ^{13}C NMR spectra of 38 in acetone-d_6

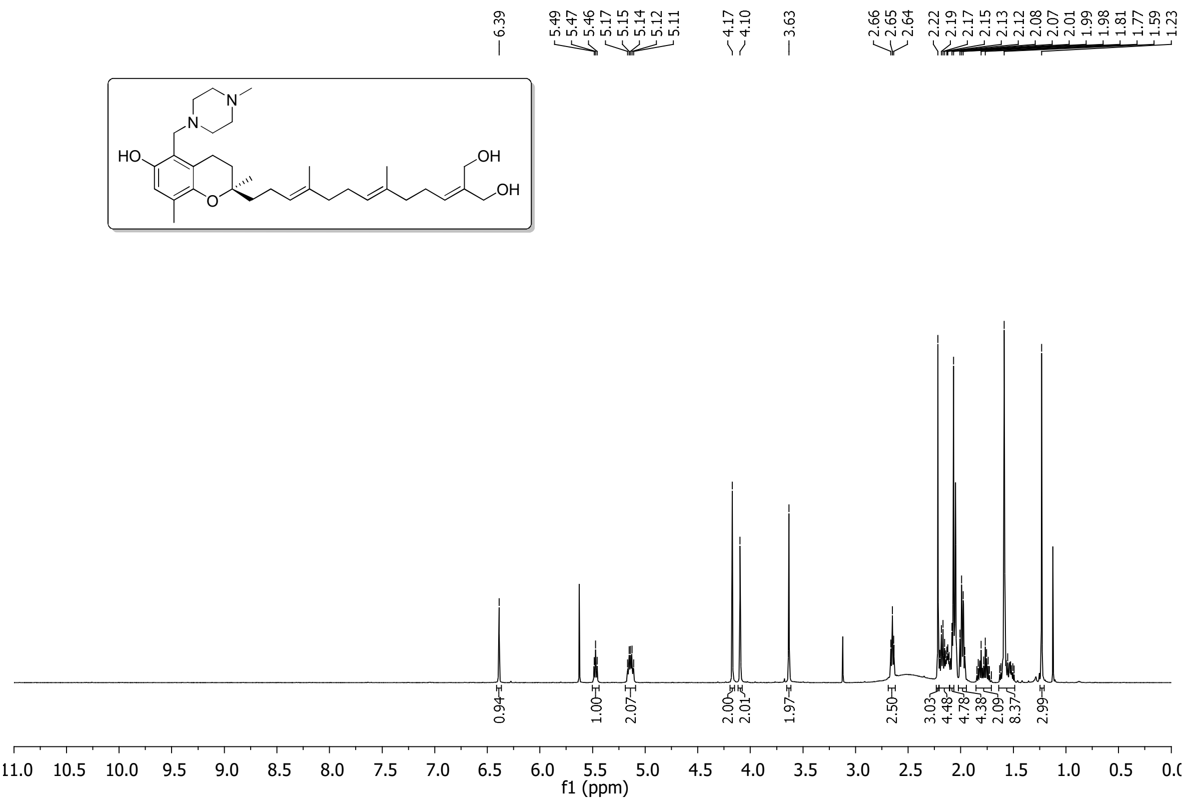

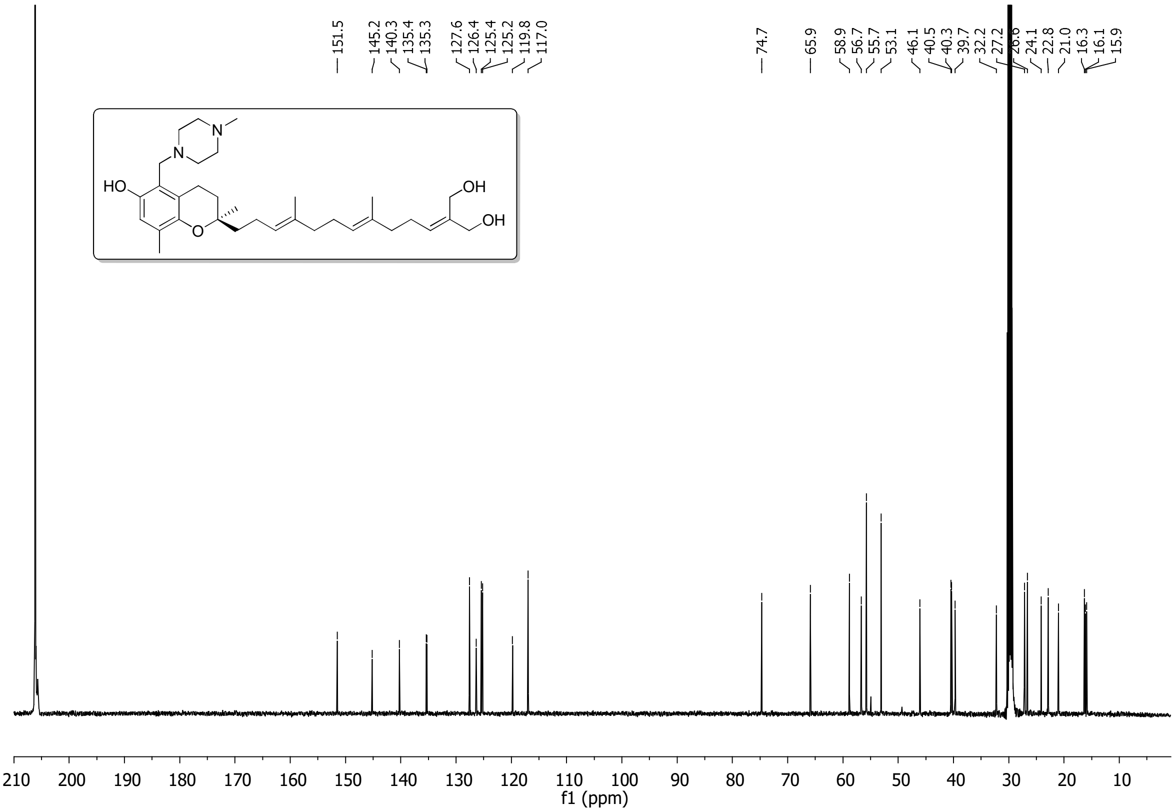

Figure S41. ^{1}H **and** ^{13}C **NMR spectra of 39 in acetone-*d_6***

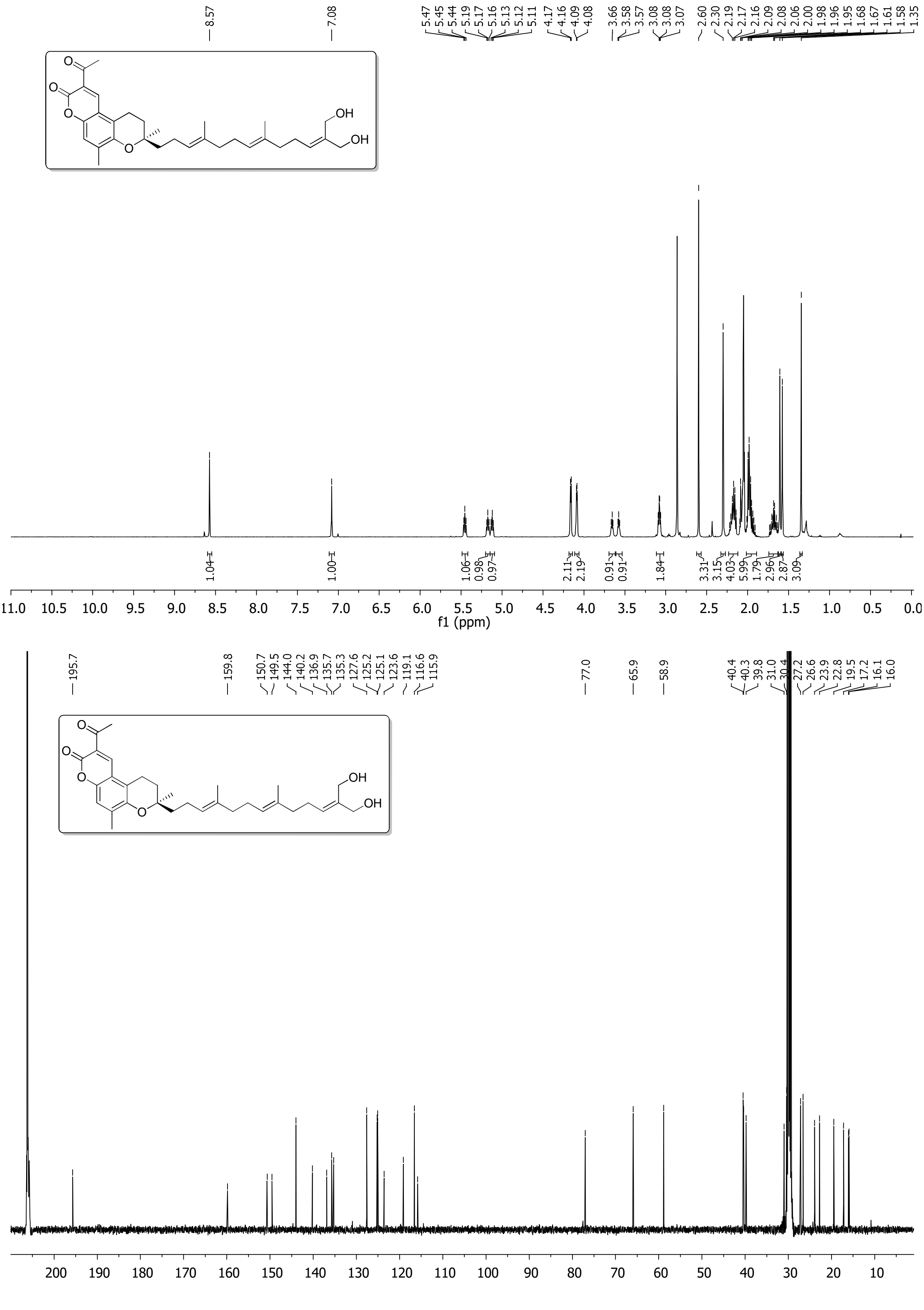

Figure S42. ^1H and ^{13}C NMR spectra of 42 in acetone-d_6

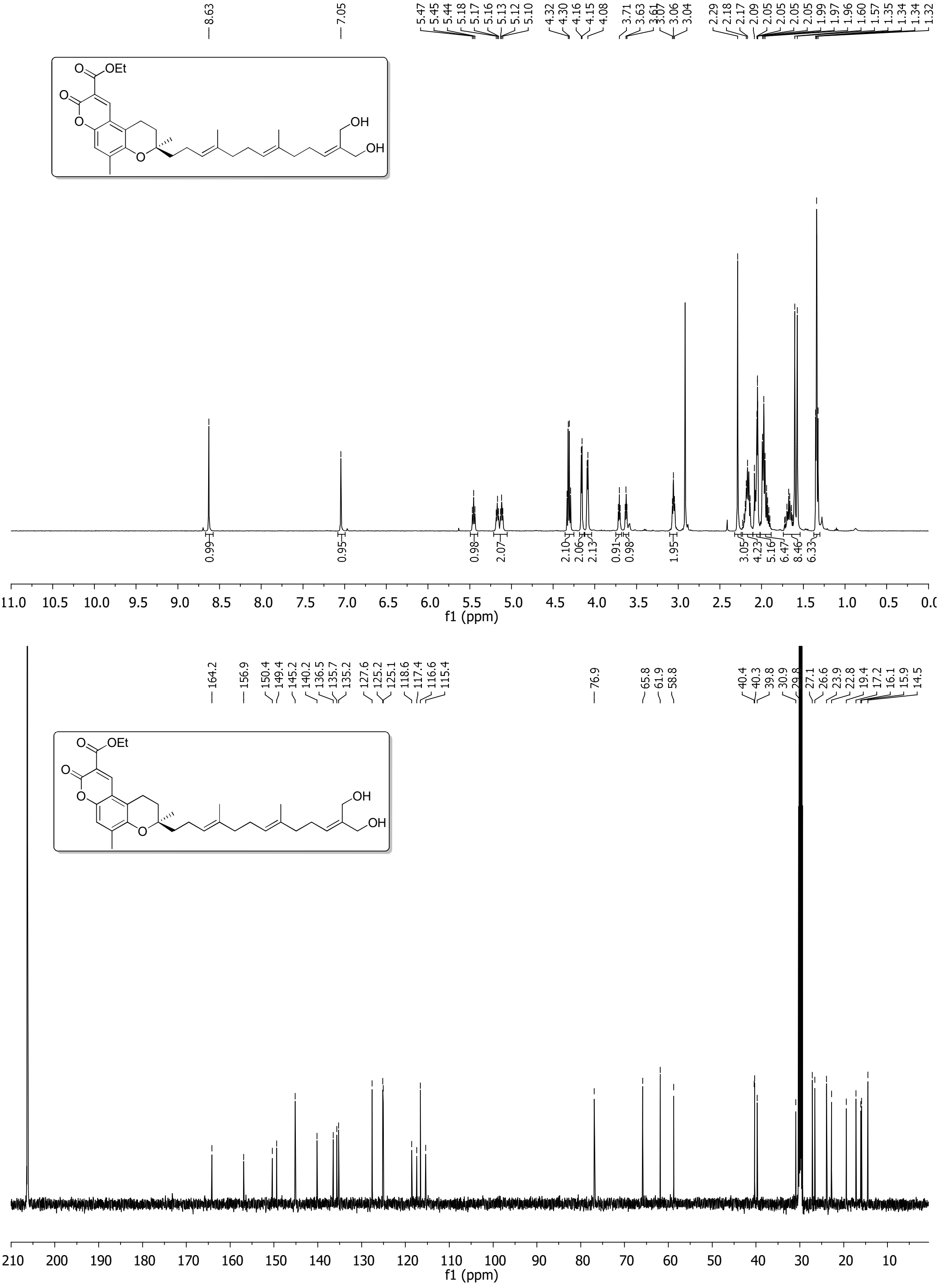

Figure S43. ^{1}H **and** ^{13}C **NMR spectra of 43 in acetone-d_6**

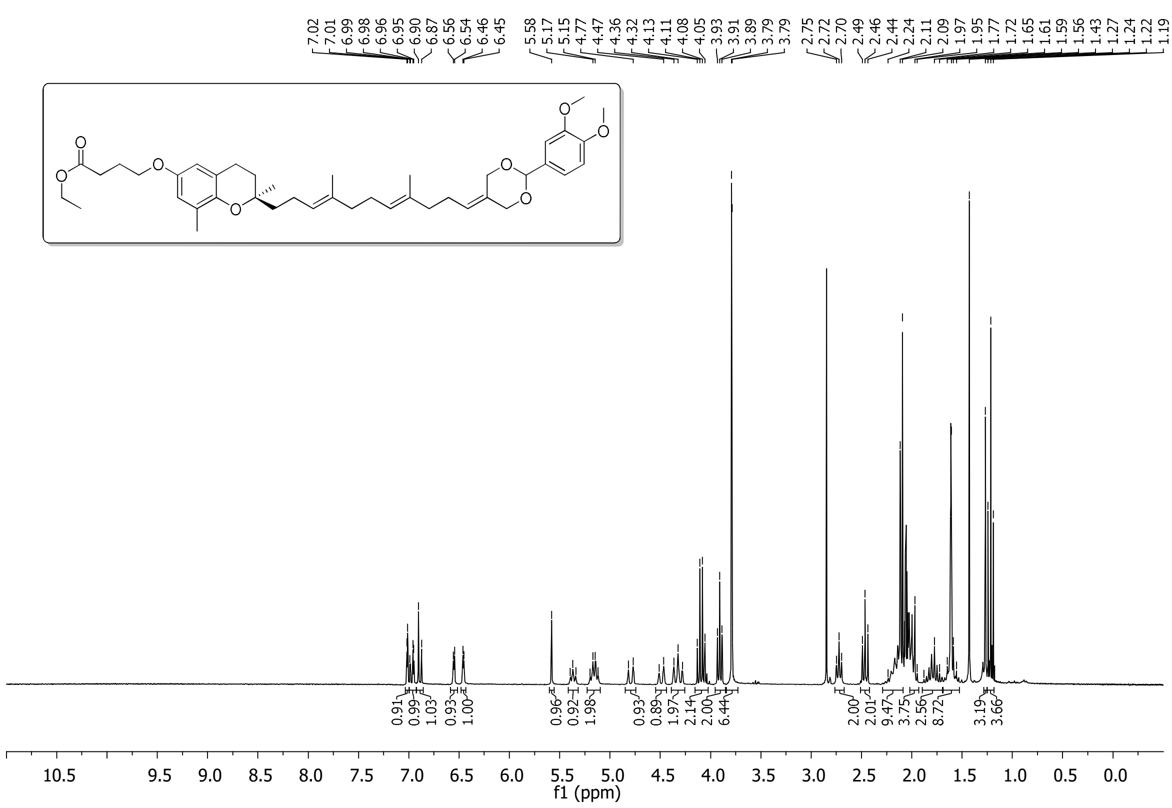

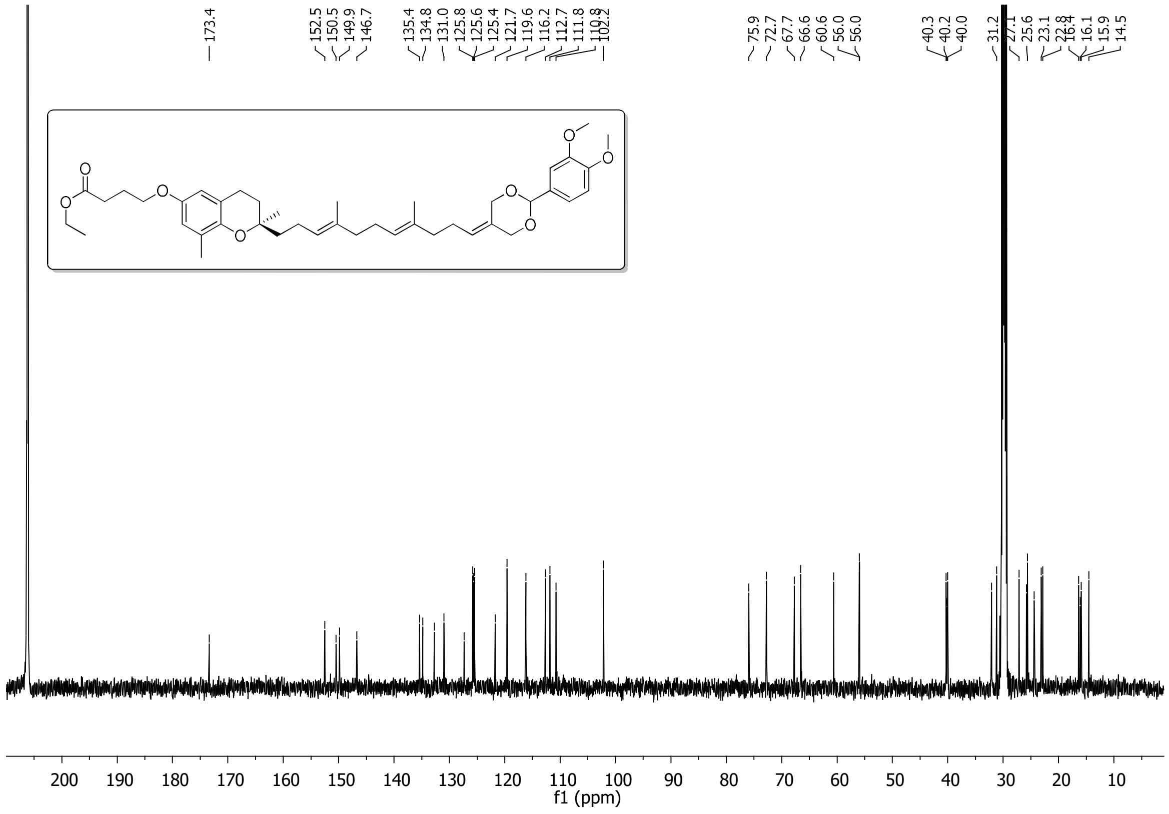

Figure S44. ^{1}H **and** ^{13}C **NMR spectra of 67 in acetone-*d*₆**

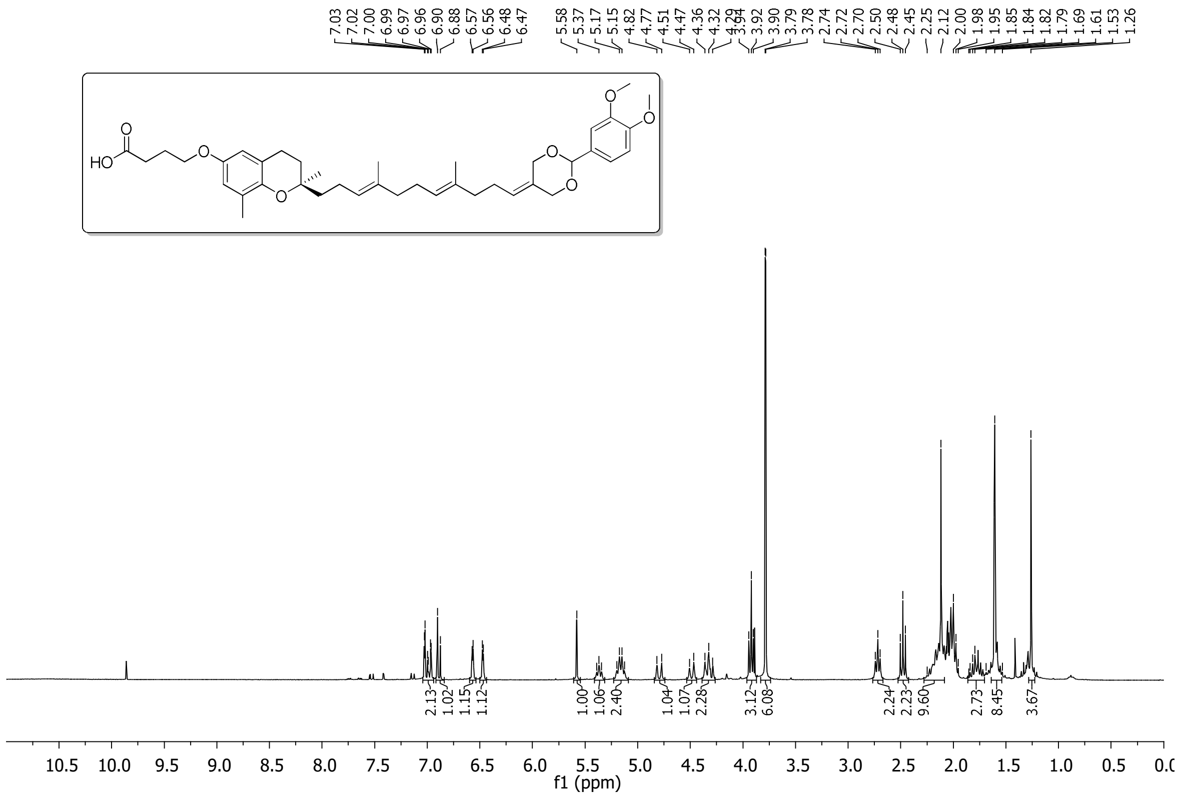

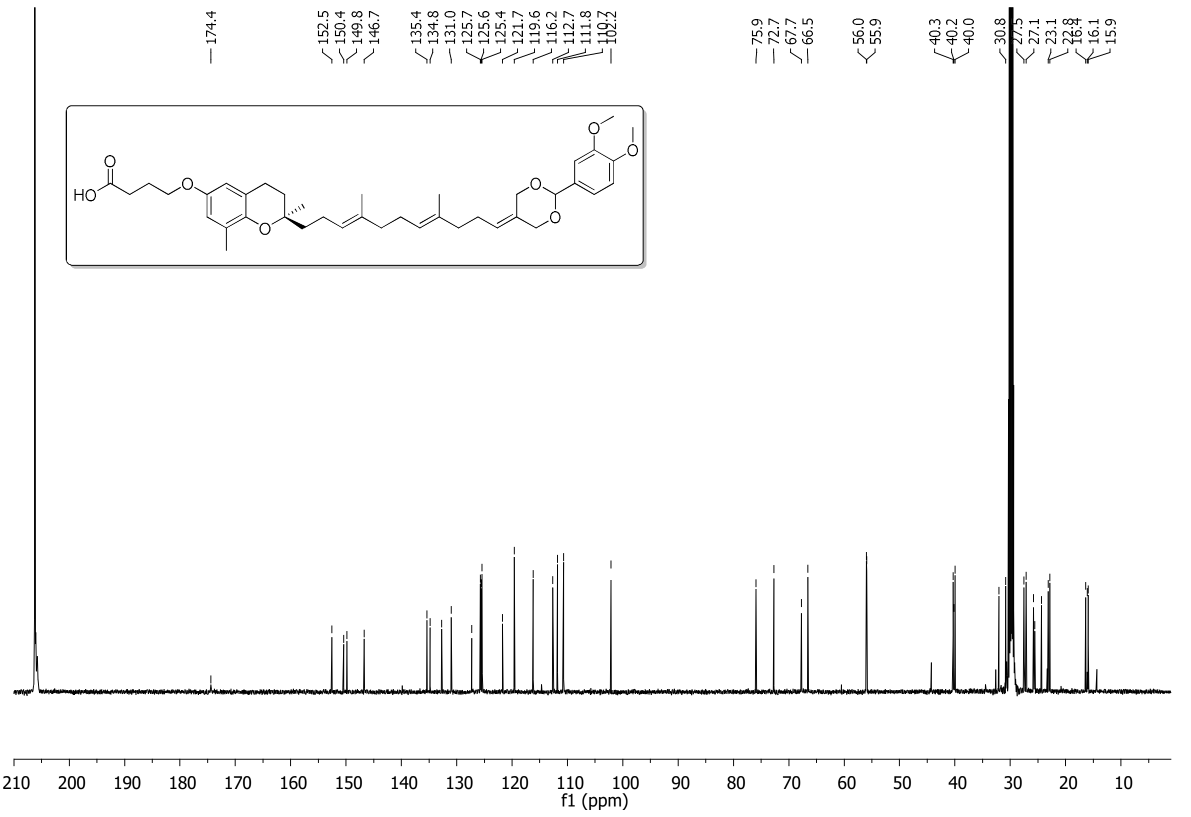

Figure S45. ^{1}H **and** ^{13}C **NMR spectra of 31 in acetone-*****d*****$_6$**

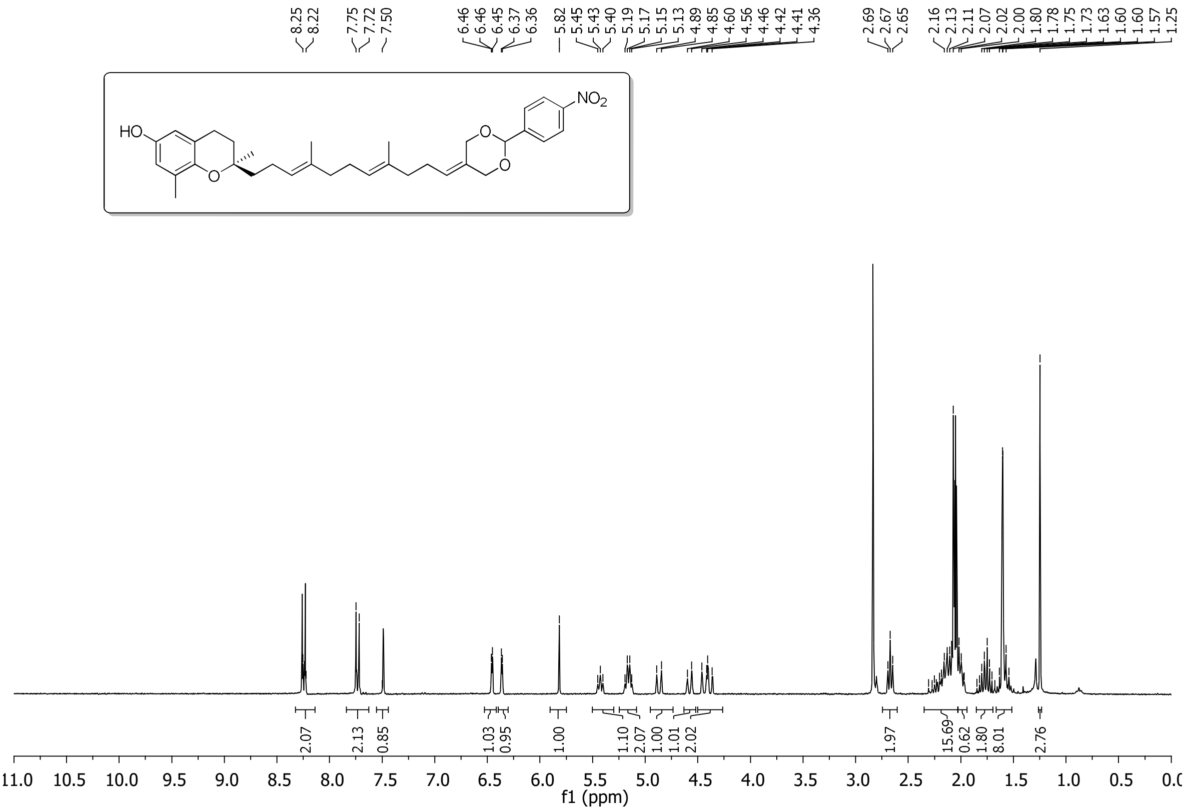

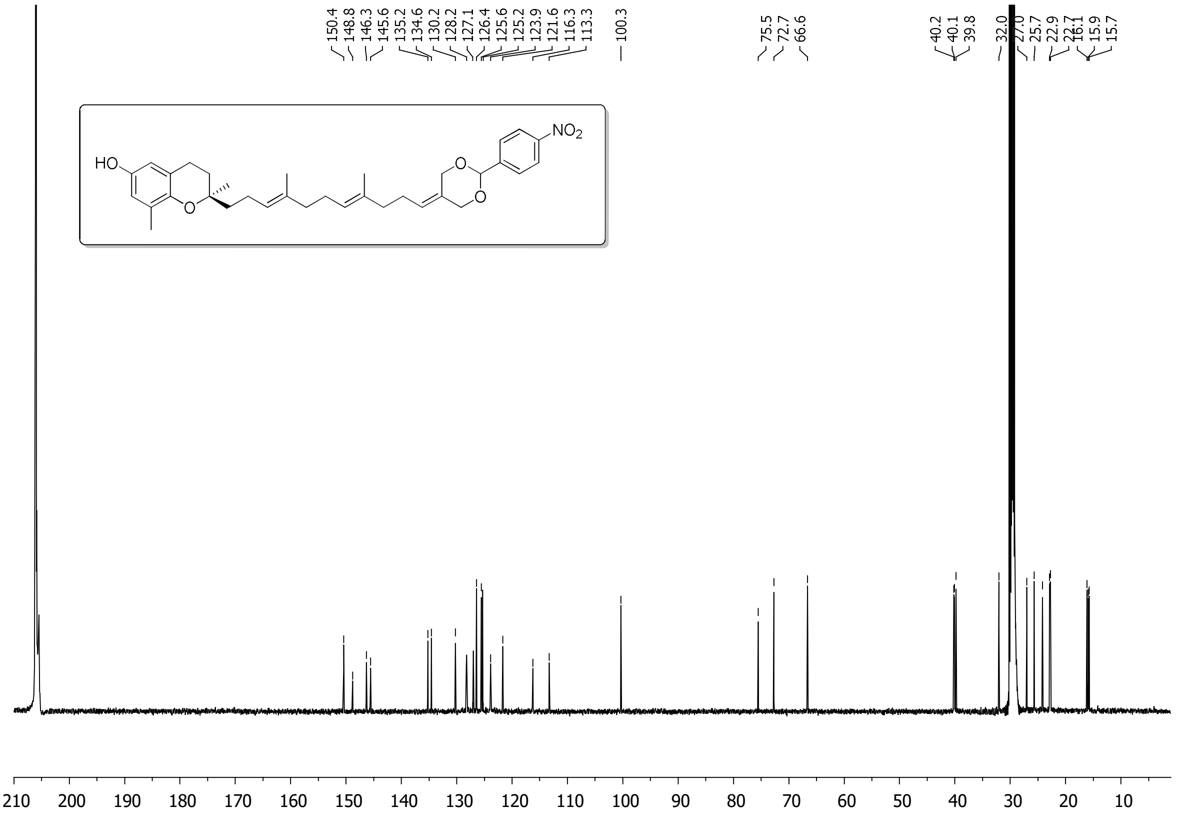

Figure S46. ^{1}H **and** ^{13}C **NMR spectra of 70 in acetone-*d*$_6$**

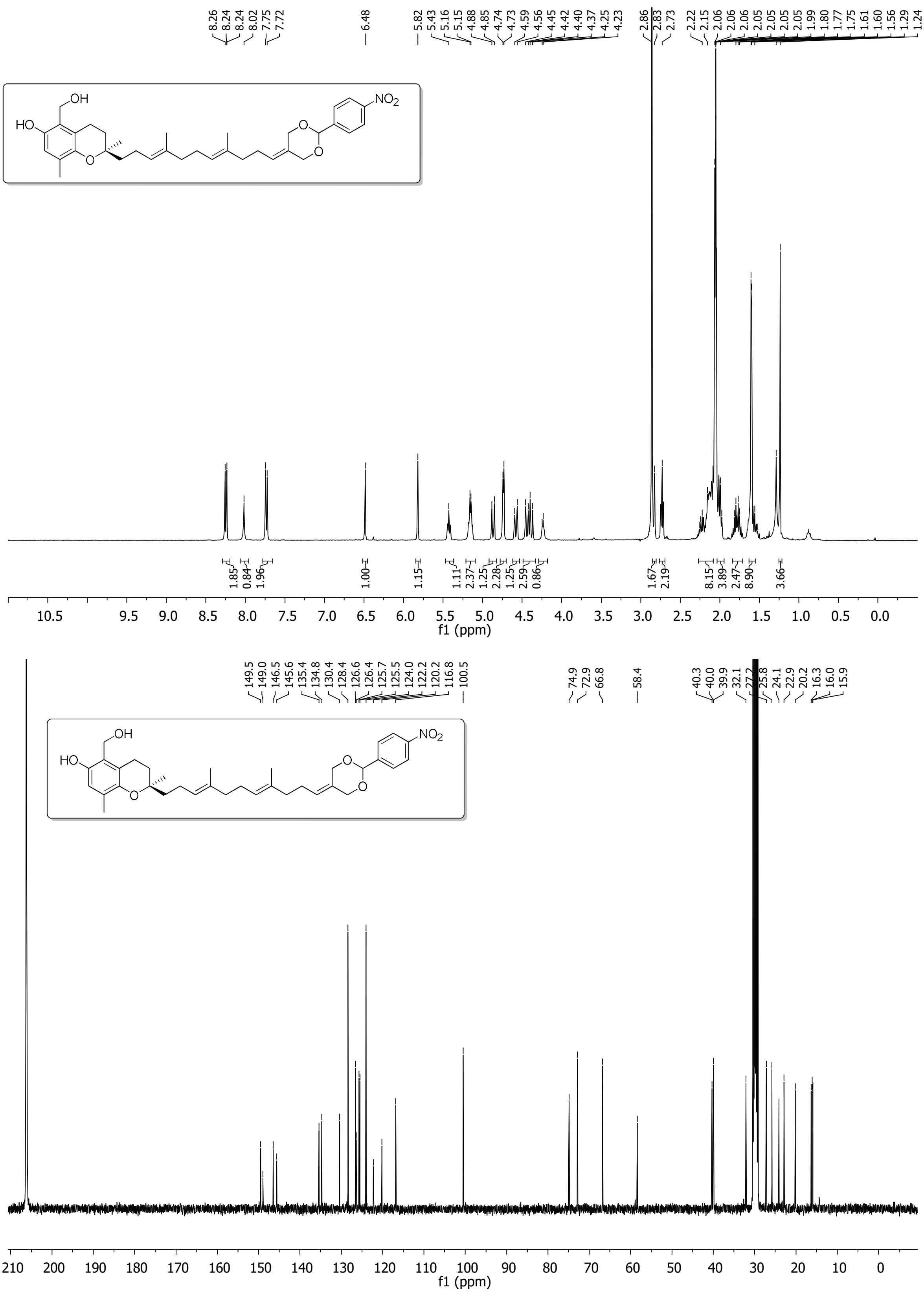

Figure S47. ^{1}H and ^{13}C NMR spectra of 48 in acetone-d_6

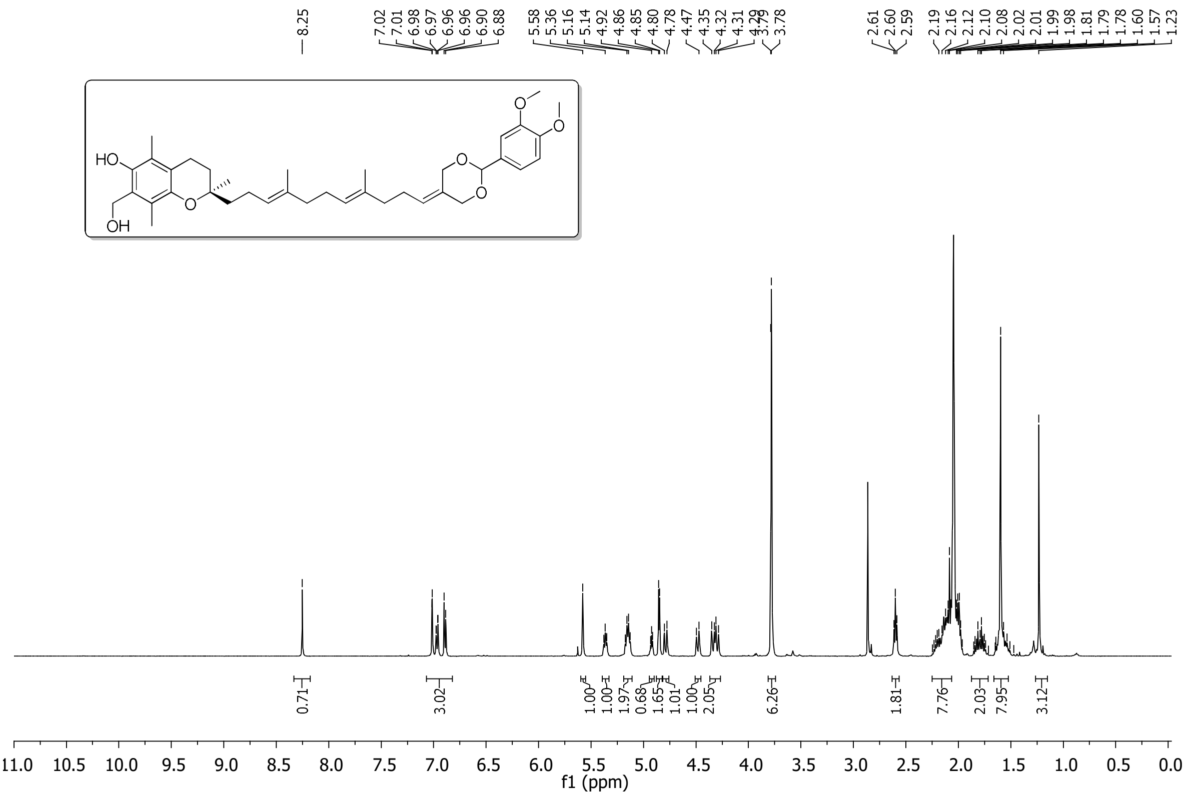

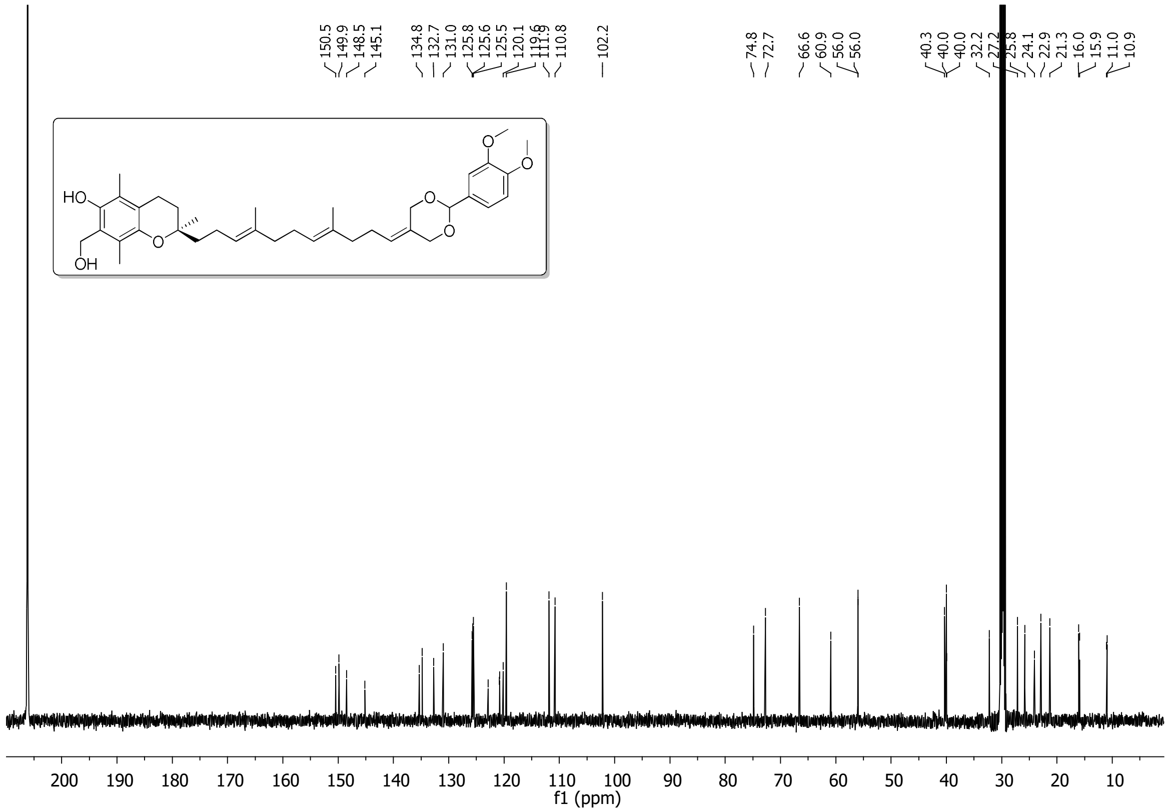

Figure S48. ^{1}H and ^{13}C NMR spectra of 46 in acetone-d_6

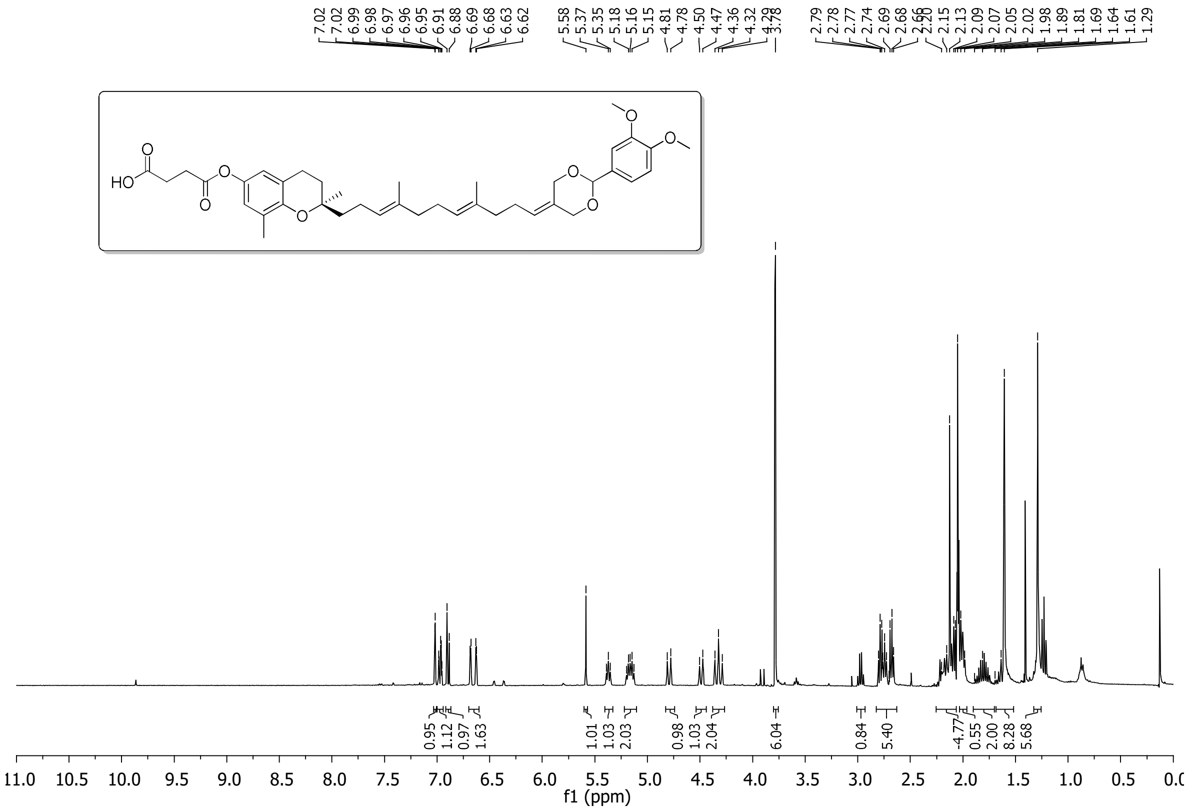

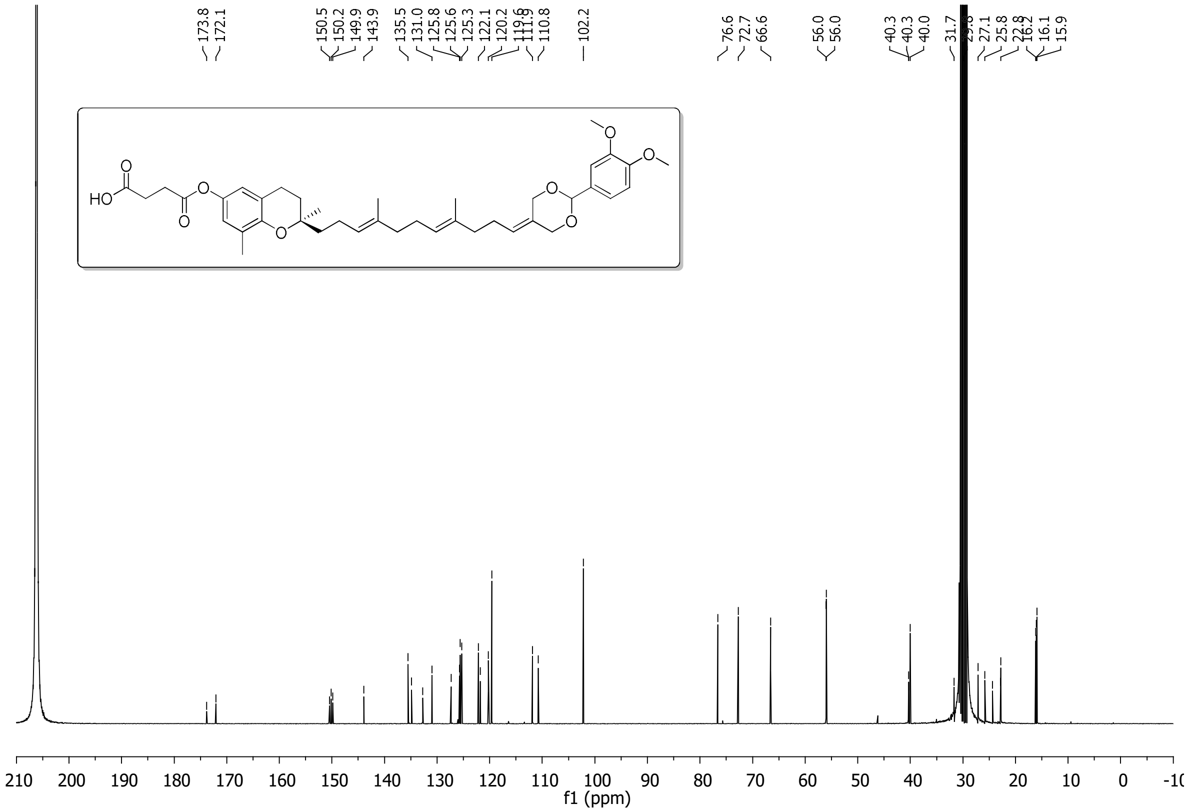

Figure S49. ^{1}H and ^{13}C NMR spectra of 68 in acetone-d_6

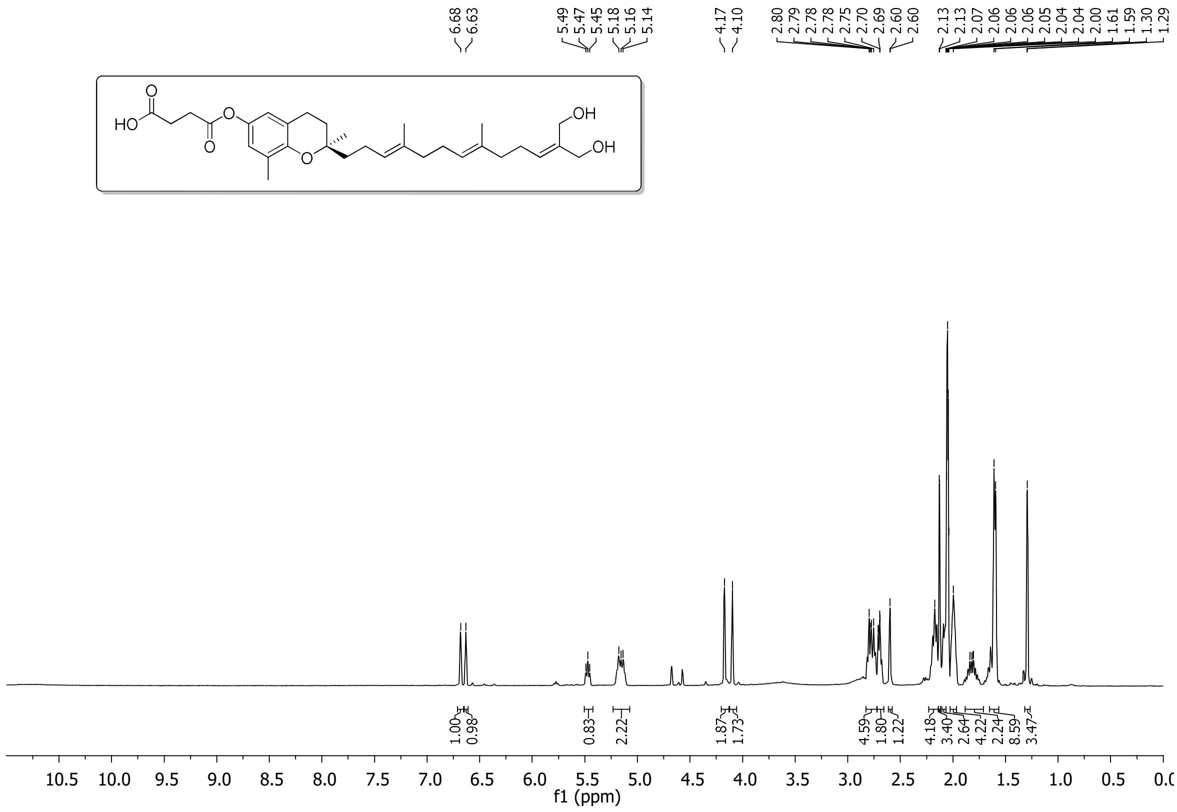

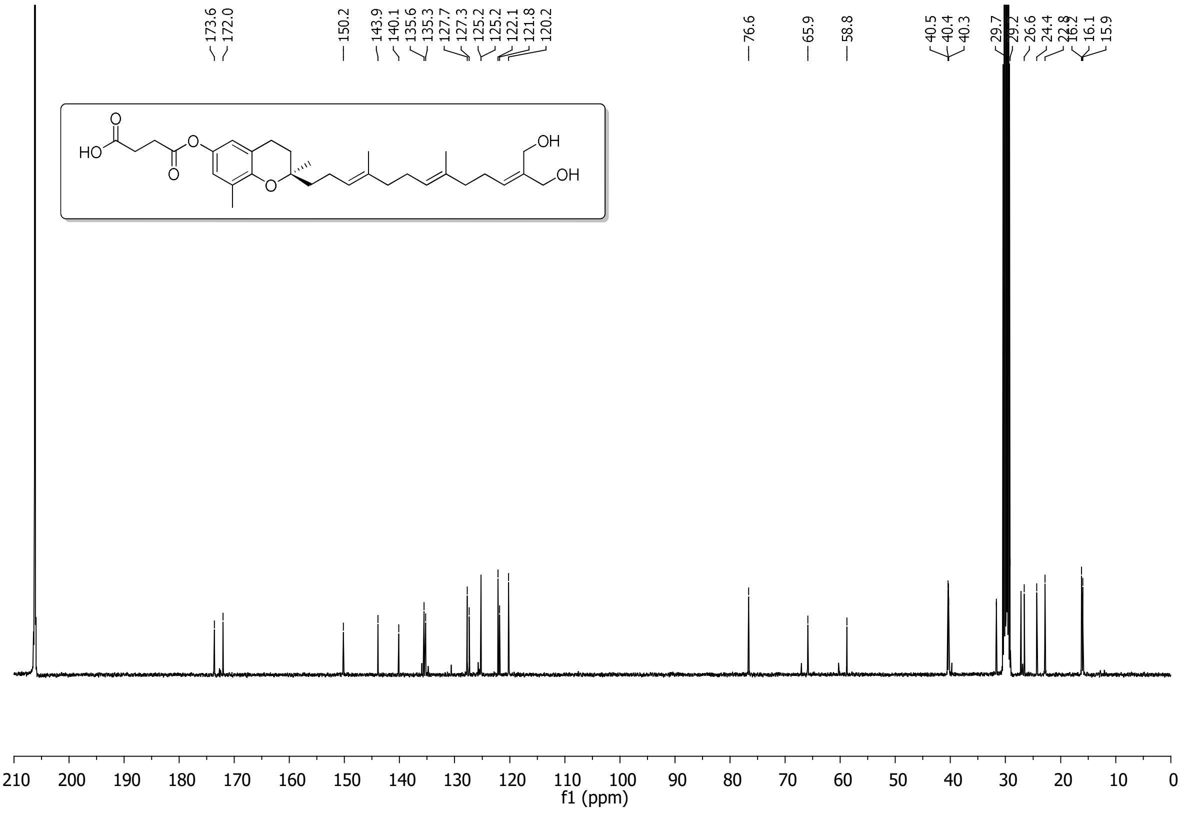

Figure S50. **^{1}H and ^{13}C NMR spectra of 31 in acetone-d_6**

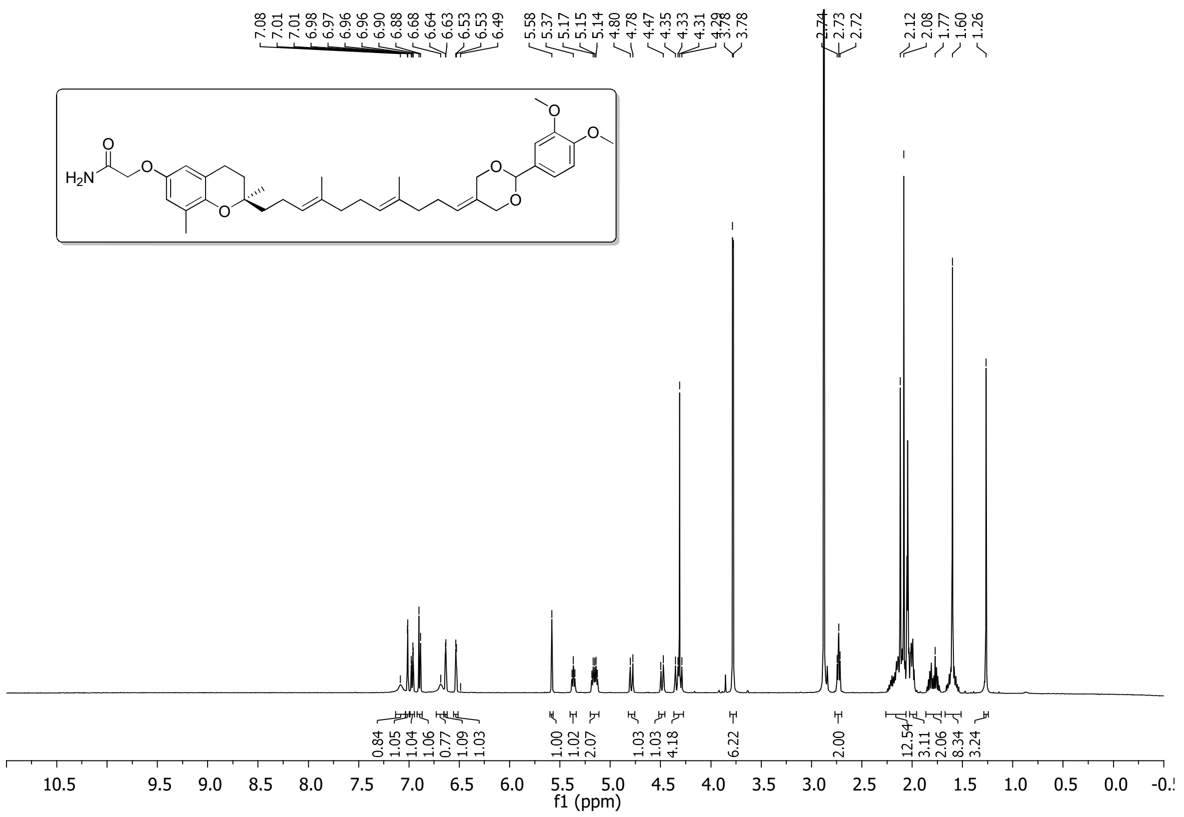

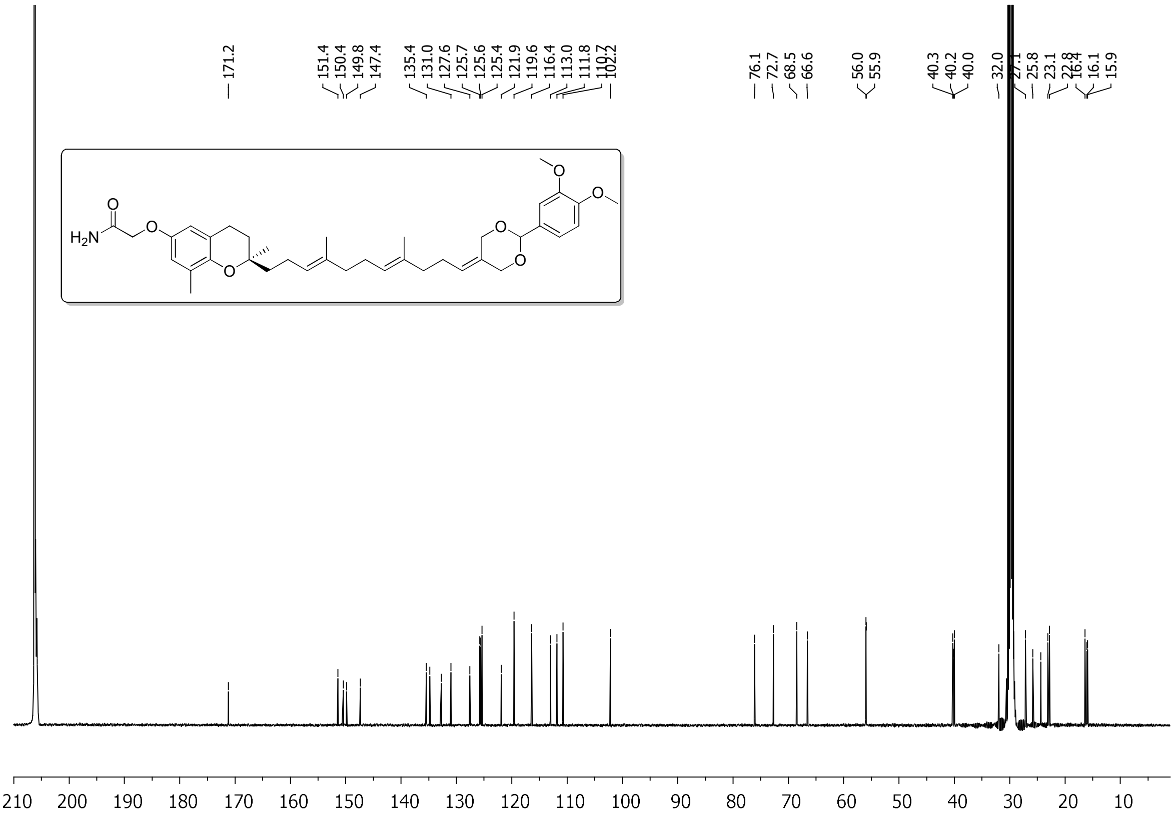

Figure S51. ^{1}H and ^{13}C **NMR spectra of 69 in acetone-d_6**

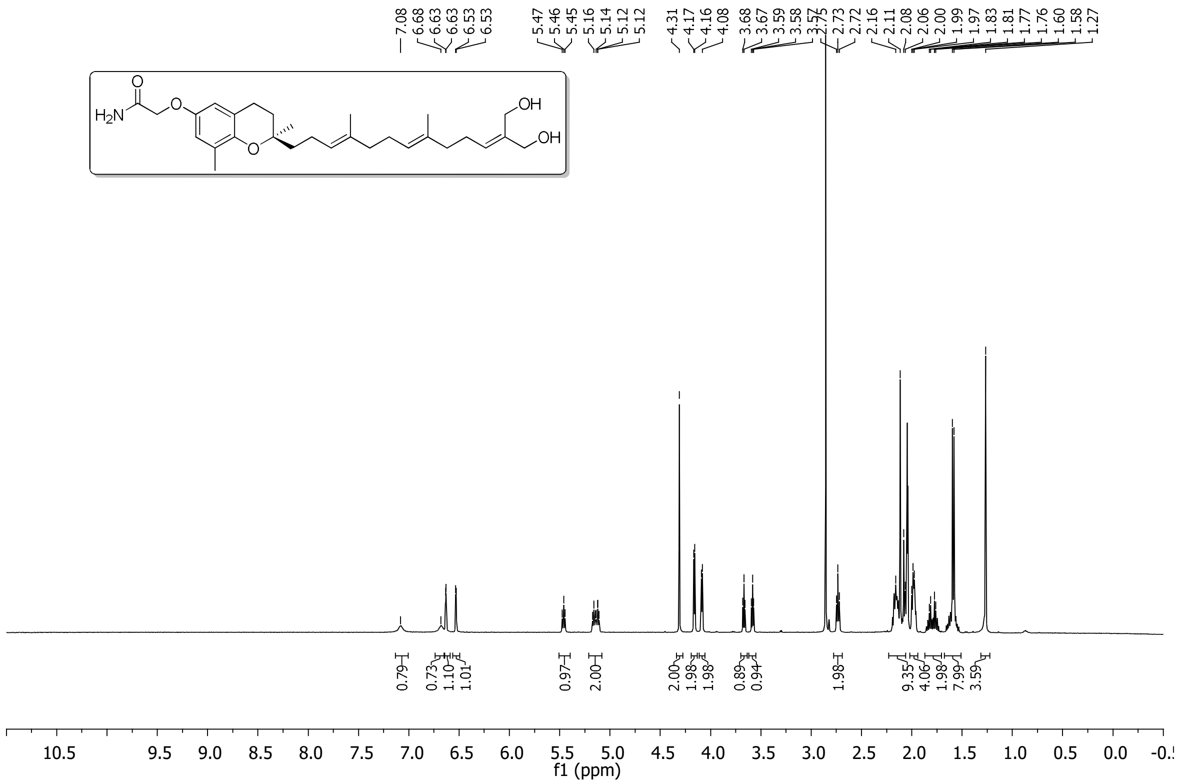

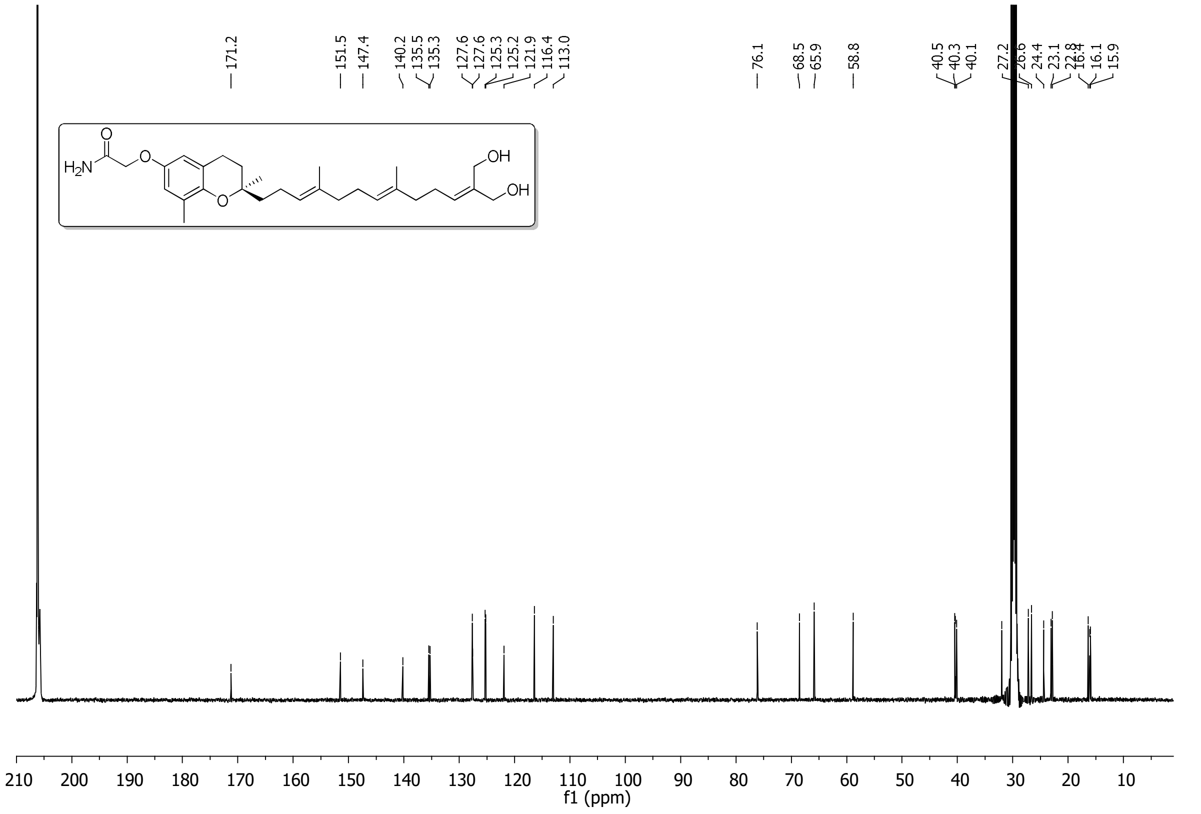

Figure S52. ^{1}**H and** ^{13}**C NMR spectra of 32 in acetone-*****d*****$_6$**

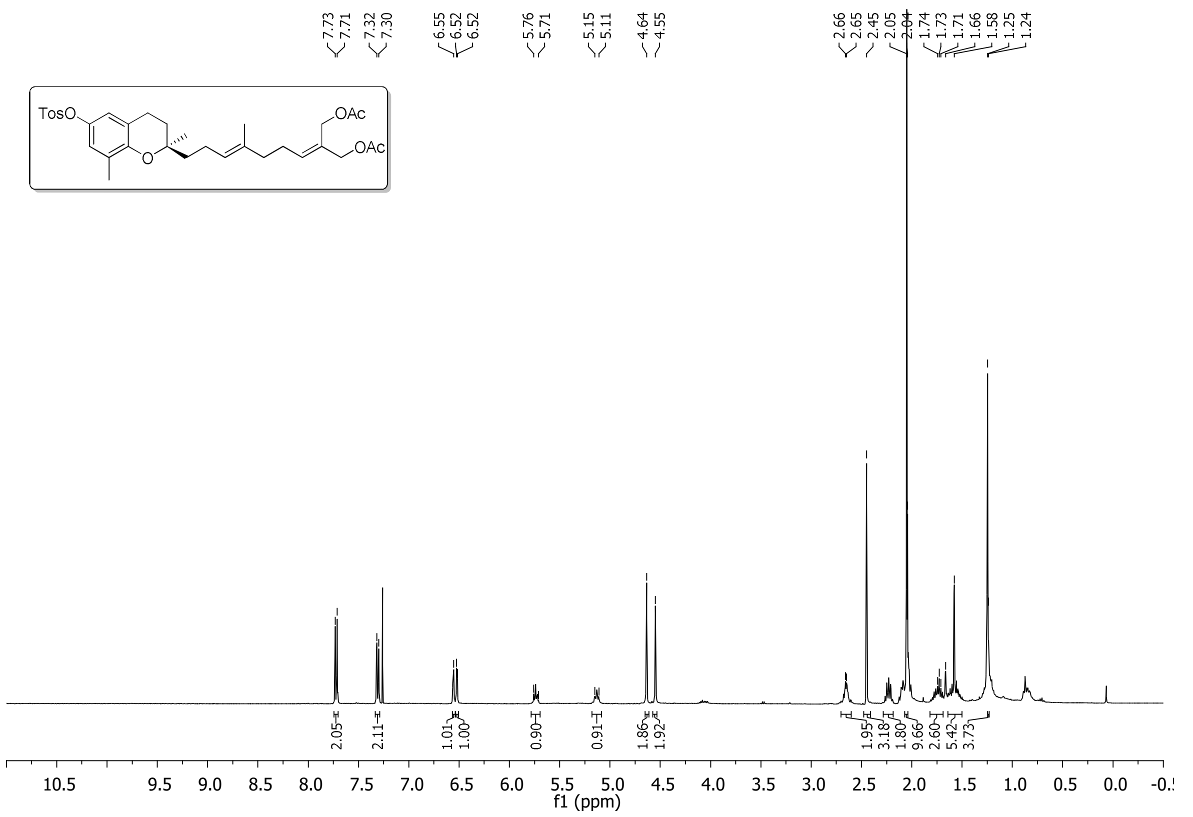

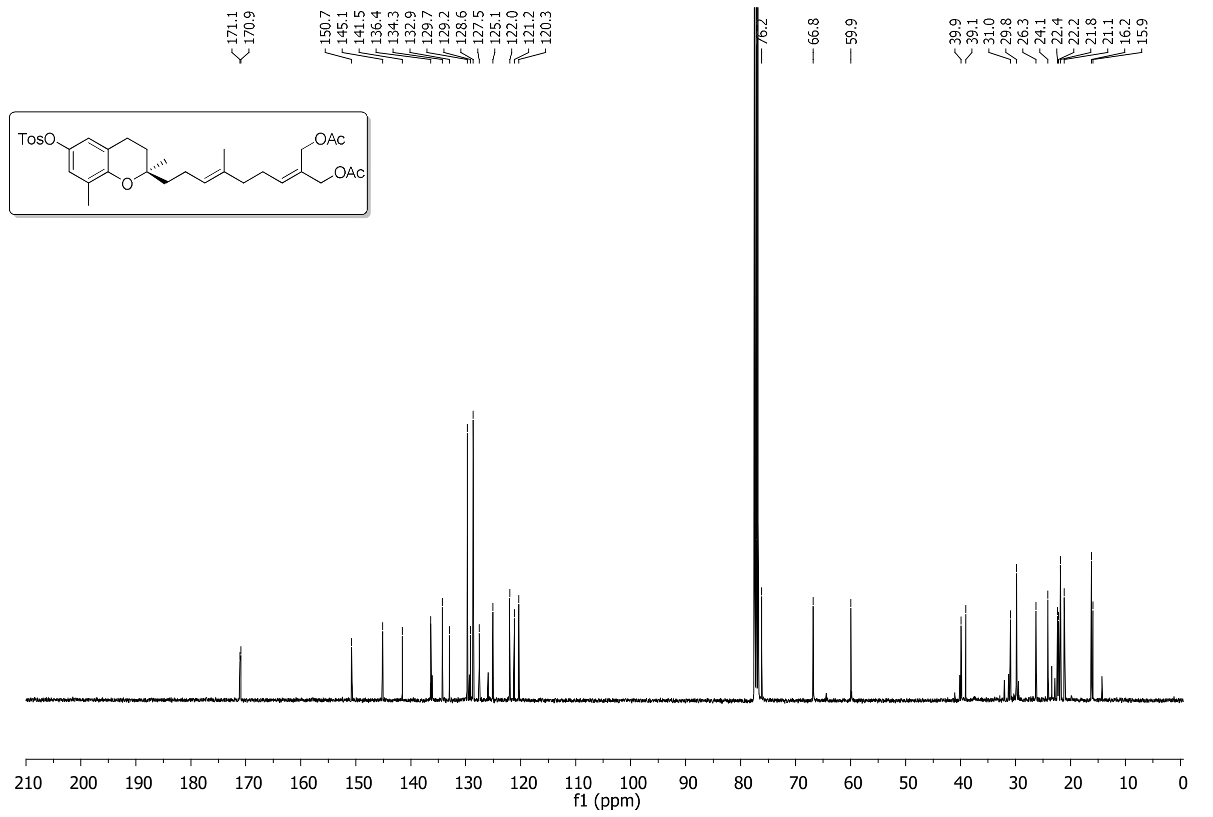

Figure S53. ^{1}H **and** ^{13}C **NMR spectra of 62 in** $CDCl_3$

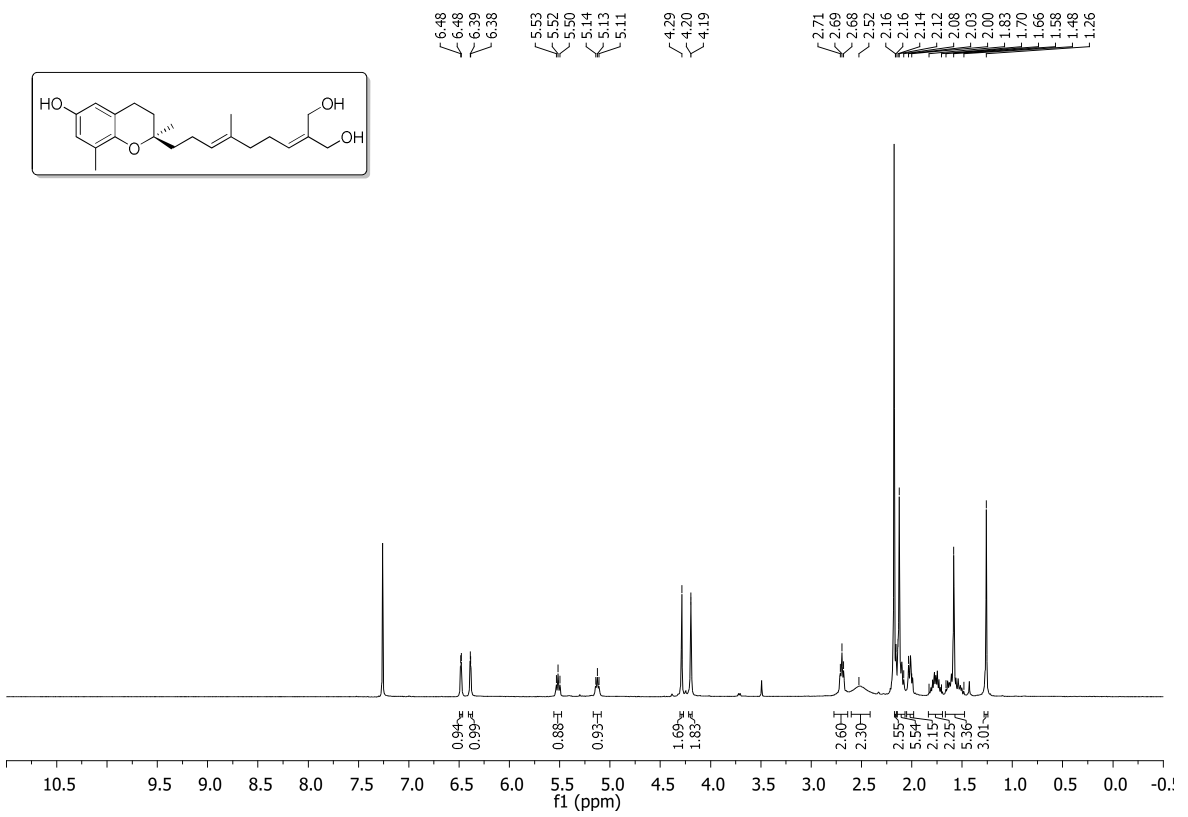

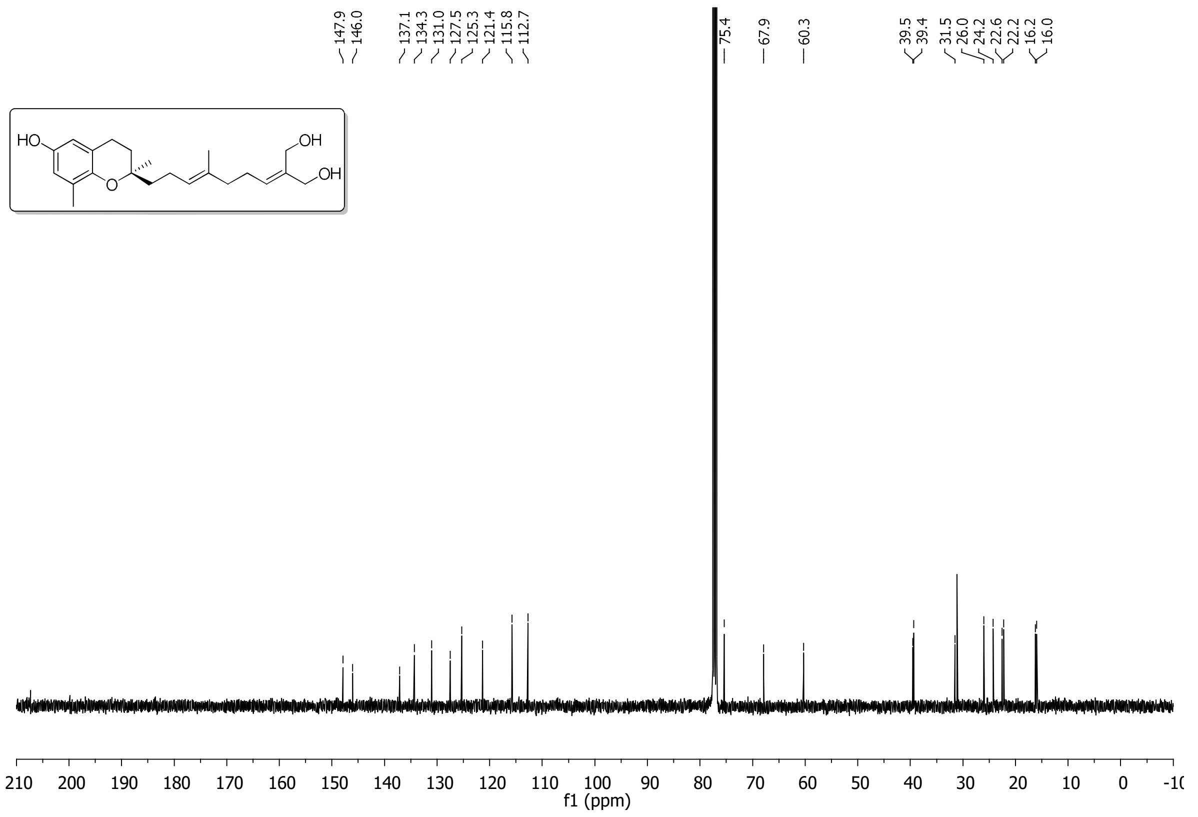

Figure S54. ^{1}H and ^{13}C NMR spectra of 28 in $CDCl_3$

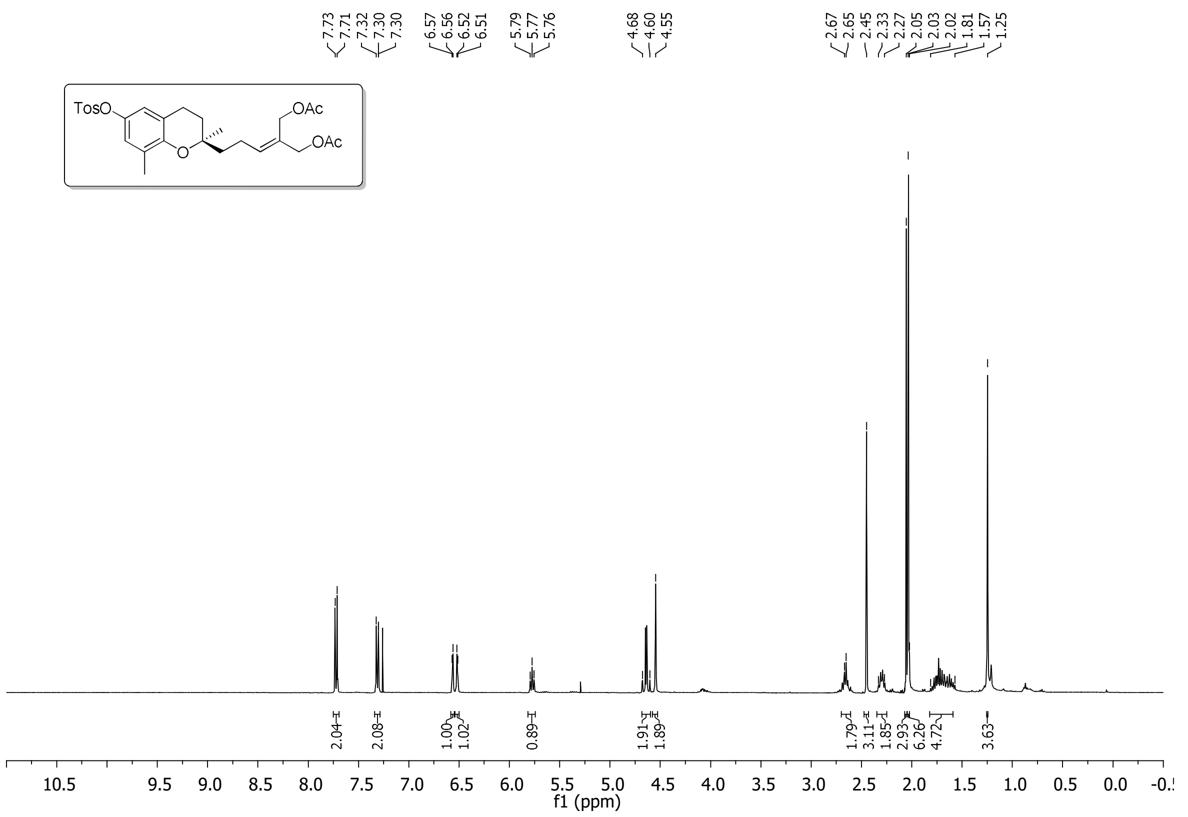

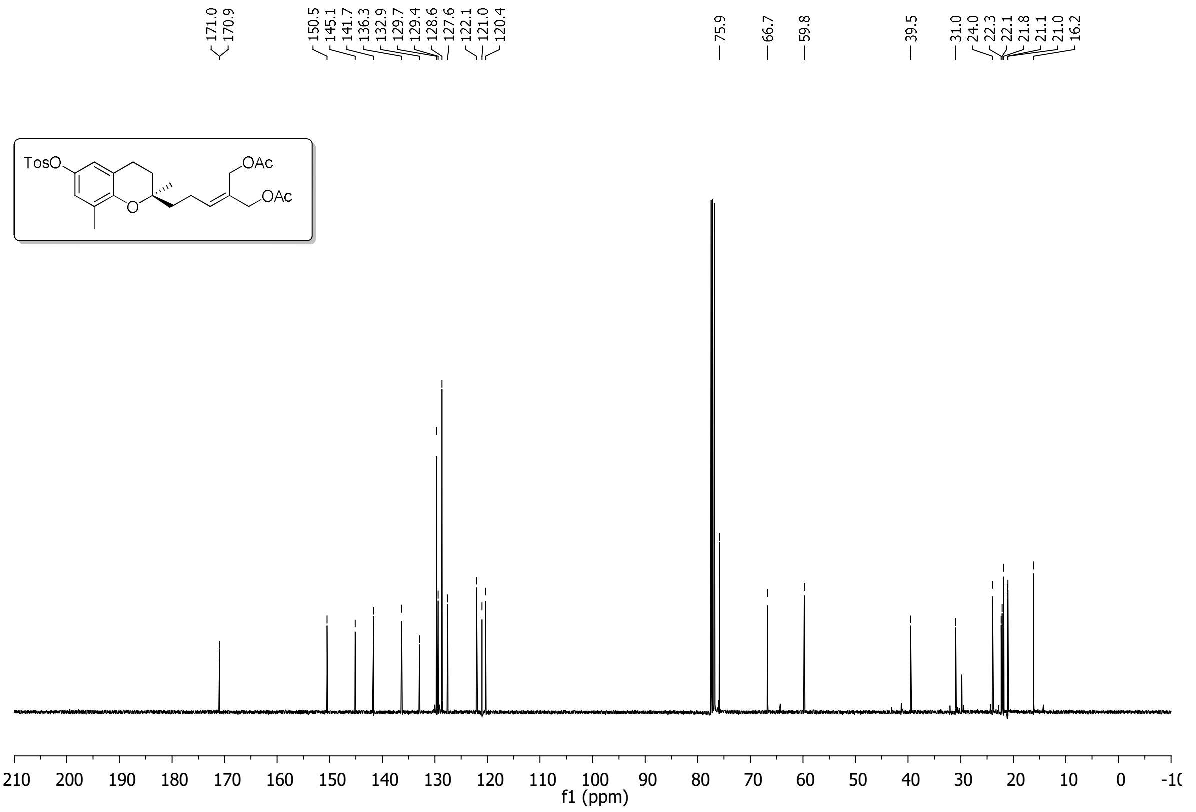

Figure S55. ^{1}H and ^{13}C NMR spectra of 63 in $CDCl_3$

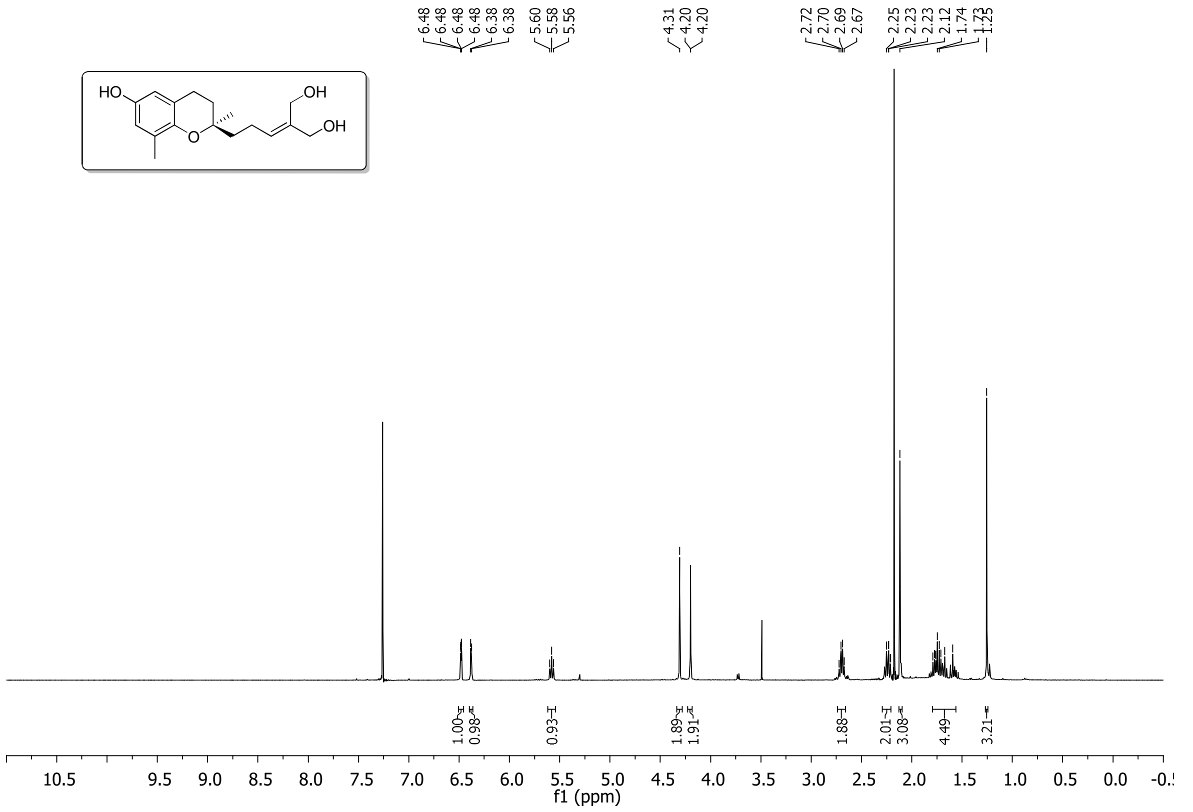

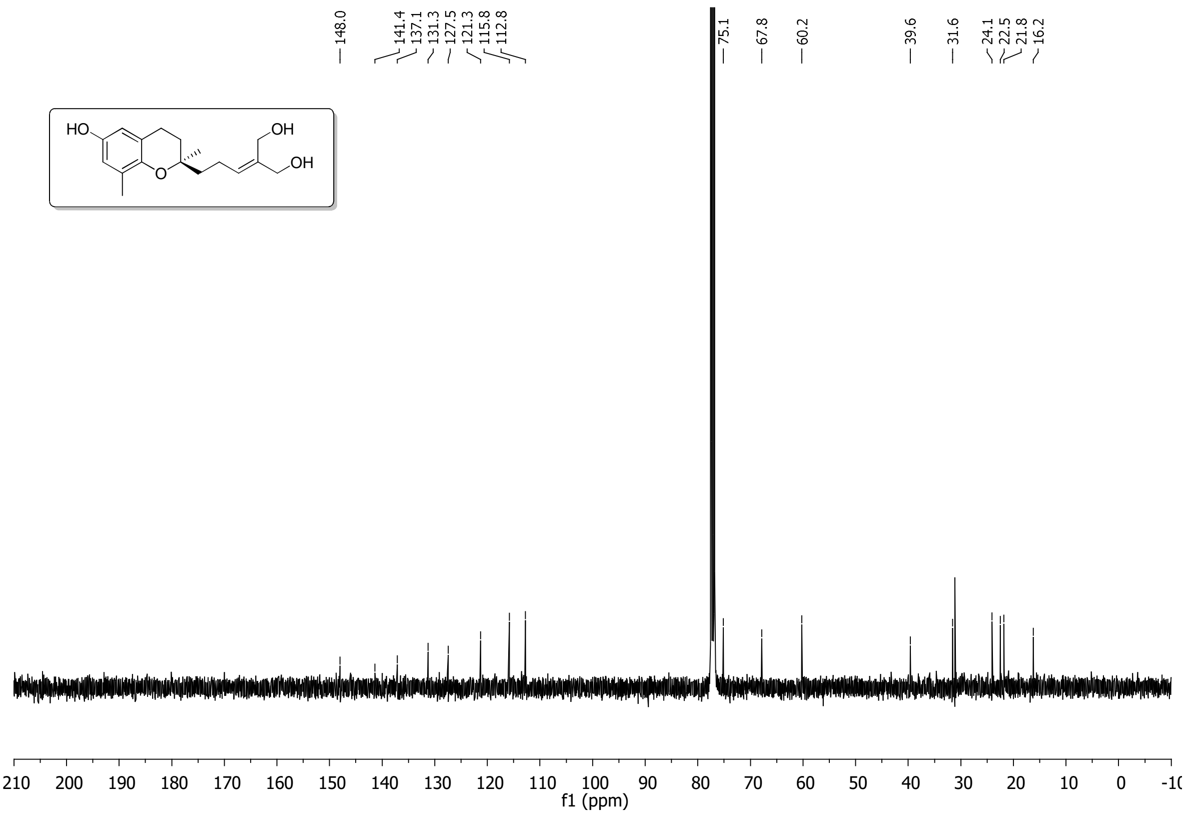

Figure S56. ^{1}H and ^{13}C NMR spectra of 29 in $CDCl_3$

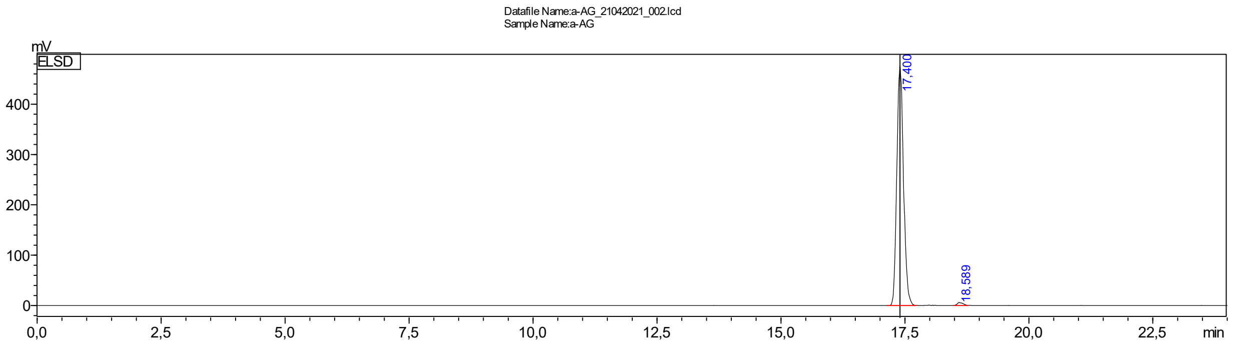

Peak #	Retention time	Area	Area [%]
1	17.4	4484067	98.891
2	18.589	50276	1.109
Total		4534343	100.0

Figure S57. HPLC-ELSD spectrum of 13a

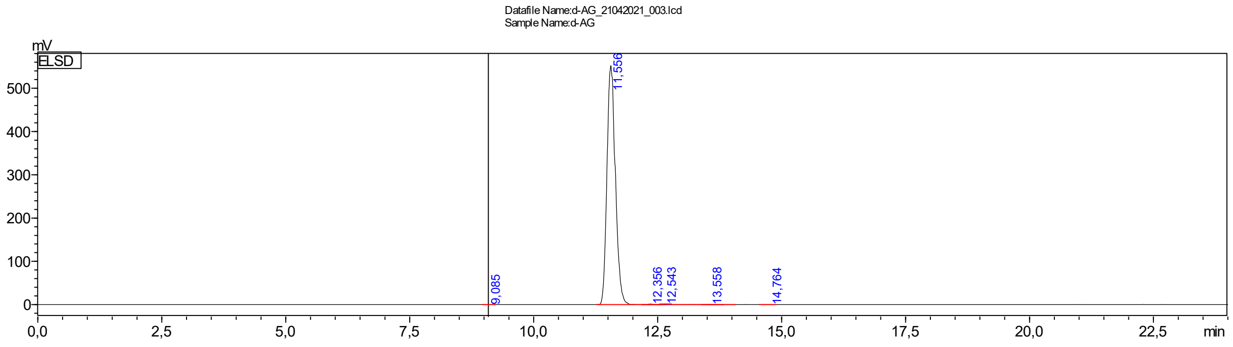

Peak #	Retention time	Area	Area [%]
1	9.085	2041	0.031
2	11.556	6517329	99.552
3	12.356	12010	0.183
4	12.543	2114	0.032
5	13.558	10641	0.163
6	14.764	2495	0.038
Total		6546630	100.0

Figure S58. HPLC-ELSD spectrum of 13d

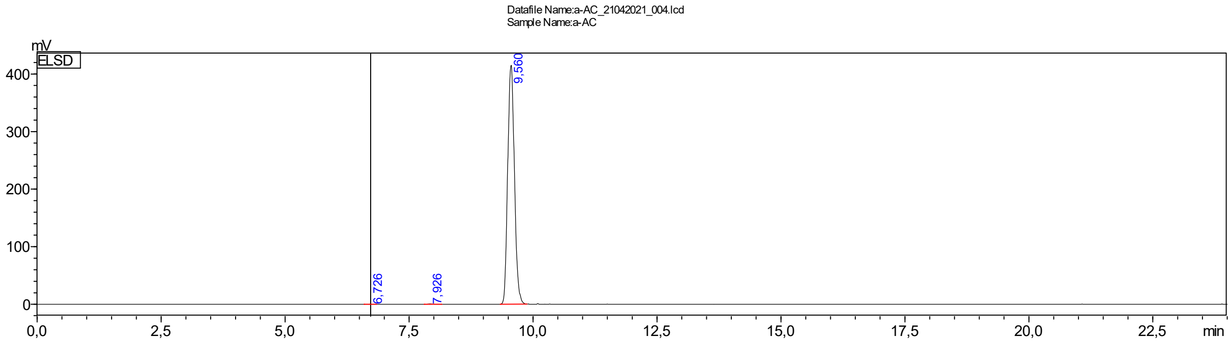

Peak #	Retention time	Area	Area [%]
1	6.726	1919	0.049
2	7.926	4813	0.123
3	9.560	3912194	99.828
Total		3918926	100.0

Figure S59. HPLC-ELSD spectrum of 27a

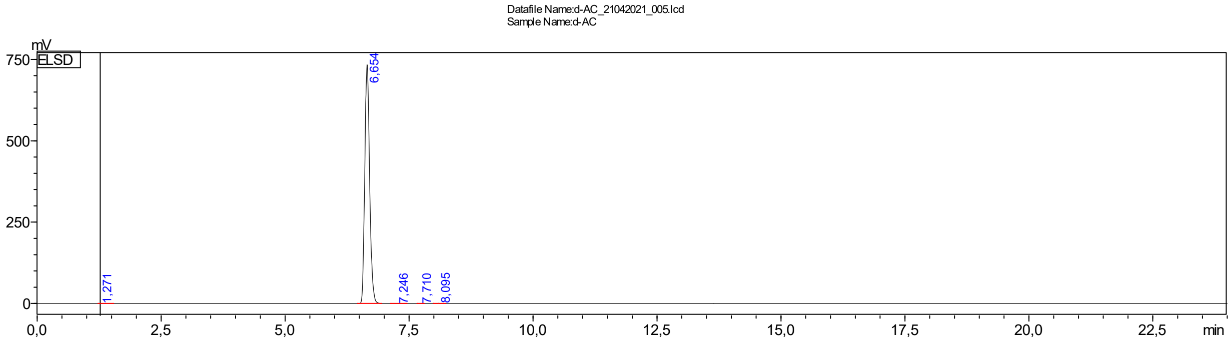

Peak #	Retention time	Area	Area [%]
1	1.271	1993	0.040
2	6.654	4995475	99.916
3	7.246	576	0.012
4	7.710	446	0.009
5	8.095	1184	0.024
Total		4999674	100.0

Figure S60. HPLC-ELSD spectrum of 27d
